# Identification of Signature Genes in the PD-1 Relative Gastric Cancer Using a Combined Analysis of Gene Expression and Methylation Data

**DOI:** 10.1155/2022/4994815

**Published:** 2022-12-15

**Authors:** Han Yu, En Li, Sha Liu, ZuGuang Wu, FenFei Gao

**Affiliations:** ^1^Department of Gastrointestinal Surgery, Meizhou People's Hospital, Huangtang Road, Meijiang District, Meizhou 514031, Guangdong Province, China; ^2^Department of Pharmacology, Shantou University Medical College, 22 Xinling Road, Shantou 515041, Guangdong Province, China

## Abstract

**Background:**

The morbidity and mortality rates for gastric cancer (GC) rank second among all cancers, indicating the serious threat it poses to human health, as well as human life. This study aims to identify the pathways and genes as well as investigate the molecular mechanisms of tumor-related genes in gastric cancer (GC).

**Method:**

We compared differentially expressed genes (DEGs) and differentially methylated genes (DMGs) in gastric cancer and normal tissue samples using The Cancer Genome Atlas (TCGA) data. The Kyoto Encyclopedia of Gene and Genome (KEGG) and the Gene Ontology (GO) enrichment analysis' pathway annotations were conducted on DMGs and DEGs using a clusterProfiler R package to identify the important functions, as well as the biological processes and pathways involved. The intersection of the two was chosen and defined as differentially methylated and expressed genes (DMEGs). For DMEGs, we used the principal component analysis (PCA) to differentiate gastric cancer from adjacent samples. The linear discriminant analysis method was applied to categorize the samples using DMEGs methylation data and DMEGs expression profiles data and was validated using the leave-one-out cross-validation (LOOCV) method. We plotted the ROC curve for the classification and calculated the AUC (area under the ROC curve) value for a more intuitive view of the classification effect. We also used the NetworkAnalyst 3.0 tool to analyze DMEGs, using DrugBank to acquire information on protein-drug interactions and generate a network map of gene-drug interactions.

**Results:**

We identified a total of 971 DMGs in 188 PD-1 negative and 187 PD-1 positive gastric cancer samples obtained from TCGA. The KEGG and GO enrichment analysis showed the involvement of the regulation of ion transmembrane transport, collagen-containing extracellular matrix, cell-cell junction, and peptidase regulator activity. We simultaneously obtained 1,189 DEGs, out of which 986 were downregulated, while 203 were upregulated in tumors. The enriched analysis of the GO's and KEGG's pathways indicated that the most significant pathways included an intestinal immune network for IgA production, *Staphylococcus aureus* infection, cytokine-cytokine receptor interaction, and viral protein interaction with cytokine and cytokine receptor, which have previously been linked with gastric cancer. The compound DB01830 can bind well to the active site of the LCK protein and shows good stability, thus making it a potential inhibitor of the LCK protein. To observe the relationship between DMEGs' expression and prognosis, we observed 10 genes, among which were TRIM29, TSPAN8, EOMES, PPP1R16B, SELL, PCED1B, IYD, JPH1, CEACAM5, and RP11-44K6.2. Their high expressions were related to high risks. Besides, those genes were validated in different internal and external validation sets.

**Conclusion:**

These results may provide potential molecular biological therapy for PD-1 negative gastric cancer.

## 1. Introduction

Gastric cancer (GC), also referred to as stomach cancer, occurs when malignant tumors grow within the inner lining of the stomach. It is the most common type of cancer in the world to affect the gastrointestinal system. The morbidity and mortality rates for this form of cancer rank second among all cancers, indicating the serious threat it poses to human health, as well as human life [1]. Gastric cancer is also the second most diagnosed cancer in China, with approximately 403,000 new cases (281,000 males and 122,000 females) and 291,000 deaths in 2015. It is also the third leading cause of death in relation to all types of cancer [2, 3]. Currently, commonly used therapies to treat gastric cancer include chemotherapy, radiotherapy, and surgery. However, many patients suffering from stomach cancer are often first diagnosed when the disease has reached an advanced stage. Despite the available therapy options, the overall response to treatment remains dismal, with a 5-year survival rate of less than 30% [4]. As the molecular biology of this stomach cancer became clearer, immunotherapy was introduced as a new therapeutic strategy. Indeed, immune checkpoint inhibitors can improve the therapeutic efficacy of gastric cancer patients by activating the patients' immune systems and enhancing their antitumor immune response [5]. The tissues affected by gastric cancer often contain several infiltrating immune cells, such as natural killer (NK) cells or T and B cells. The occurrences of these activated immune cells, along with the associated effector molecules at elevated levels, indicate a longer survival [6]. Besides, the overexpression of PD-L1 also indicates a dismal survival in people with gastric cancer [7]. However, we still lack effective biomarkers for the prognoses of patients with PD-1 negative gastric cancer. Therefore, the discovery and application of new biomarkers were crucial for improving the therapeutic effects and predicting the prognoses of patients with PD-1 negative gastric cancer.

Gastric cancer is considered a heterogeneous disease, which involves a multistep and multifactor process. Multiple cumulative epigenetic and genetic changes—such as tumor suppressor gene mutations and hypermethylation—turn normal cells into tumor cells. This results in tumorigenesis and the development of stomach cancer and affects the disease's biological behavior [8]. Over the past few years, research focusing on gastric cancer has increasingly been paying attention to the epigenetic mechanisms controlling this type of cancer, including long non-coding RNAs (non-coding ribonucleic acid), microRNAs, histone modification, and DNA methylation [9]. DNA methylation is a biochemical process and a considerably important epigenetic factor in tumorigenesis. It has been previously reported that methylation of CpG islands may induce tumorigenesis and aberrant methylation of CpG islands may affect the function of the tumor suppressor gene by changing the expression level of CpG islands [10, 11]. Because of its stable and easy-to-detect properties, DNA methylation can also be used as a good biomarker and may become a meaningful target for cancer diagnosis and treatment. Gastric cancer has shown high rates of DNA hypermethylation [12]. However, previous studies found that various aberrant methylation can lead to gene inactivation and gene silencing and promote the development of gastric cancer [13, 14]. In Epstein-Barr virus (EBV) positive gastric cancer, methylation patterns of several tumor suppressor genes, including CHD1 and P16, have been altered and were considered essential tumorigenesis mechanisms [15]. Meanwhile, *Helicobacter pylori* infection and gene promoter hypermethylation was involved in multiple steps of carcinogenesis. *Helicobacter pylori* infection may result in methylation of the trefoil factor (TFF) family 2 and E-cadherin promoters. However, and despite extensive research, the clinical impact of these studies remains limited. The exact mechanism by which DNA methylation induces gastric cancer remains unclear, and the drugs targeting these potential biomarkers were lacking.

We used numerous analyses of aberrant methylation and gene expression for differential expression genes (DEGs) and differential methylation genes (DMGs) [16–18]. We applied bioinformatics to recognize candidate genes and try to understand the genetic foundation of the disease. Therefore, we must use a combined approach when analyzing the gene expression profiles chip and the gene methylation chip in gastric cancer. Using TCGA data, this study compared DNA methylation and DEGs in normal tissues and tissues suffering from gastric cancer. Through enrichment analyses, we screened the determining genes and pathways in stomach cancer that might affect the development of this type of cancer. We also screened DrugBank to identify the potential drugs that targeted upregulated DMEGs. We identified and confirmed 10 DMEG genes in different internal and external validation sets. Our study showed that gastric cancer cells contained 114 dysregulated DMEGs, which might be used as molecular biomarkers to aid in the early recognition of PD-1 negative gastric cancer. These results may provide potential molecular biological therapy for gastric cancer.

## 2. Methods

### 2.1. Data Source and Preprocessing

We acquired the most recent stomach adenocarcinoma (STAD) expression profiles, methylation data, and information on clinical follow-up through the TCGA GDC API on October 2, 2020. We also downloaded the expression profiles data and survival information of the GSE30219 data set from GEO.

The TCGA data were processed following these methods:

1. Only normal samples and primary tumor samples were retained
2. Based on the PD-1 gene expression level, tumor samples whose PD-1 level was lower than the mean of PD-1 level of normal samples were defined as PD-1 negative samples, while those whose PD-1 level was higher were considered PD-1 positive samples
3. We obtained 407 samples, including 375 tumor samples and 32 normal samples, from TCGA, which contained 188 PD-1 negative samples and 187 PD-1 positive samples, while methylation data contained 162 PD-1 negative samples and 176 PD-1 positive samples

### 2.2. Methylation Data Analysis

There were 485,577 probes in the Illumina HumanMethylation450 BeadChip array, accounting for 99% (*n* = 21231) of RefSeq genes. In each probe, the initial methylation strength was articulated as a *β* value. We compared the methylation data between normal and tumor samples of gastric cancer using the R limma package to identify CpG sites (DMS) of differential methylation. We also used the Benjamini and Hochberg (BH) method to adjust each *P*-value to the false discovery rate (FDR). The thresholds recognized as DMS were absolute delta *β*-value >0.3 and FDR <0.05.

We obtained the CpG sites and gene matching files from Illumina's website (https://www.illumina.com). In different regions (3′-UTR (3′-untranslated region), TSS1500, intergenic region, TSS200, TSS (transcriptional start site), 5′-UTR (5′-untranslated region), first exon, and gene body), each gene's average *β* value was calculated according to the corresponding relationship. We then took the integrated gene methylation data to obtain differentially methylated genes (DMGs) using the R limma package. The methylation regions identified as FDR <0.01. And delta *β*-values >0.3 were classified as hypermethylated regions, while those identified as FDR <0.01 and delta *β*-values <−0.3 were classified as demethylated regions.

### 2.3. Expression Profile Data Analysis

The DEGs between tumor samples and normal samples were analyzed using the R limma package, and *P* values were adjusted using the BH method. TCGA data were log2 transformed, and DEGs were identified as follows: genes with FDR < 0.01 and log2FC > 1 were considered upregulated genes, while genes with FDR <0.01 and log2FC <− 1 were considered downregulated genes.

### 2.4. Differentially Expressed Methylated Genes in Different Regions

To identify the association between expression profile and methylation, we calculated the intersection of DEGs and DMGs as differentially methylated and expressed genes (DMEGs). We then divided them into four distinct groups, namely HyperDown, HypoDown, HypoUp, and HyperUp. The detailed grouping criteria are shown in Table 1.

### 2.5. Functional Enrichment Analysis

We conducted the Kyoto Encyclopedia of Genes and Genomes (KEGG), the gene ontology (GO) enrichment analysis, as well as the pathway functional annotations analysis on DMGs and DEGs using the clusterProfiler R package to detect the crucial functions linked to the differential genes and the essential biological processes and pathways involved.

### 2.6. Evaluation of the Markers of Methylation and Expression Profile

We conducted the principal component analysis (PCA) to differentiate gastric cancer and paracancer samples for DMEGs. We applied the linear discriminant analysis (LDA) when categorizing the samples using DMEGs expression profiles data and methylation data, respectively, and the leave-one-out cross-validation (LOOCV) was applied to validate this analysis. We plotted the ROC curve for the classification and calculated the AUC value for a more intuitive classification effect.

### 2.7. Identification of Potential Target Therapeutic Drugs

We identified the drugs potentially targeting upregulated DMEGs by screening DrugBank using NetworkAnalyst 3.0, a web-based visualization platform that provides a comprehensive analysis and interpretation of system-level gene expression data. We used it to analyze DMEGs and used the DrugBank database to analyze protein-drug interactions to generate a network map of gene-drug interactions.

Based on drug-target pairs from DrugBank and the key PPI network in the string (with a threshold score of 400), we calculated the proximity of the drug to gastric cancer. Here, we defined *S* as the gastric cancer-associated gene set, DMEGs; *D* as the node degree of the gastric cancer-associated gene set in PPI; *T* as the drug target gene set; and *d(s*, *t)* as the shortest path of *s* node and *t* node (where *s* ∈ *S* was gastric cancer-related gene and *t* ∈ *T* was the drug target gene):

(1) $$\begin{array}{c} d\left(S,T\;\right)=\frac{1}{\left|T\right|}\sum_{t\in T}\min_{s\in S}\left(d\left(s,t\right)+\omega \right). \end{array}$$

Where *ω* was the weight of the target gene. If the target gene was one of a gene in the BPH-related gene set, the calculation method was *ω* = –ln (*D* + 1). Otherwise, it was *ω* = 0.

We generated a simulated reference distance distribution for the drug. To do so, we simply randomly selected a group of protein nodes in the network as the target of the drug. The number of nodes was the same as the target size (*R*). Then, we calculated the distance d (*S*, *R*) between the simulated drug targets (representing the simulated drug) and DMEGs and generated the simulated reference distributions after 10,000 random repetitions. Meanwhile, the mean and standard deviation of the reference distribution of *μ*_*d*_ (*S*, *R*) and *σ*_*d*_ (*S*, *R*) and the corresponding observed distance were converted into standardized scores, namely, the degree of closeness (*Z*):

(2) $$\begin{array}{c} z\left(S,T\right)=\frac{d\left(S,T\right)-\mu_{d\left(S,R\right)}}{\sigma_{d\left(S,R\right)}}. \end{array}$$

We found that whether it was DMEGs or our randomly selected gene set (Supplementary Table 1); when drug distances distribution was concentrated at 1 to 2, we performed multiple hypothesis tests based on the random data from reference and selected 26 drugs (Supplementary Table 2) with small distances and FDR <0.01, as a candidate drug set related to the DMEGs gene set.

## 3. Results

### 3.1. PD-1 Expression and Immune Characteristics

**Figure 1:**
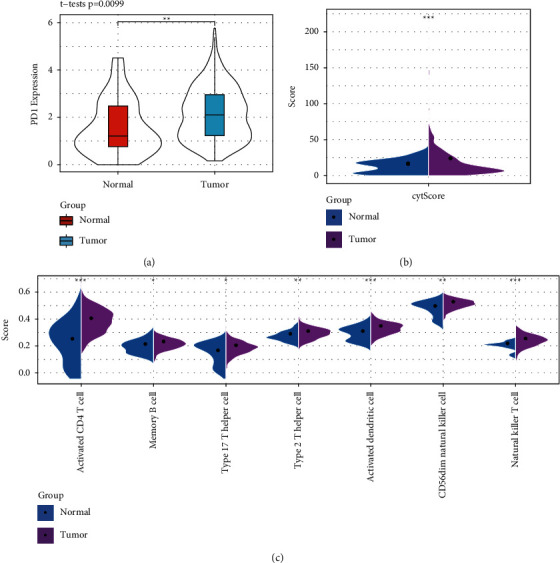
(a) The expression of PD-1 in tumor samples and normal samples, (b) violin plots of the scores difference of immune cell cytolytic activity (CYT) in tumor samples and normal samples, and (c) seven different immune T cell scores obtained by ssGSEA from tumor and normal tissue samples. ^*∗*^*P* < 0.05, ^*∗∗*^*P* < 0.01, and ^*∗∗∗*^*P* < 0.001.

To observe the expression of PD-1 in human gastric cancer, we compared the PD-1 expression in normal versus tumor cells, as illustrated in Figure 1(a). The distribution of PD-1 expression in tumor samples significantly differed from that in normal samples. Besides, we compared the microenvironment differences between normal and tumor samples, the scores of immune cells' cytolytic activity (CYT) (Figure 1(b)), and seven different types of immune T cells (Figure 1(c)) obtained by ssGESA. We found out that normal tumor samples' CYT and T cell scores were lower than those of tumor samples, which revealed the immunosuppression in gastric cancer tumor samples.

### 3.2. Analysis of DMGs

**Figure 2:**
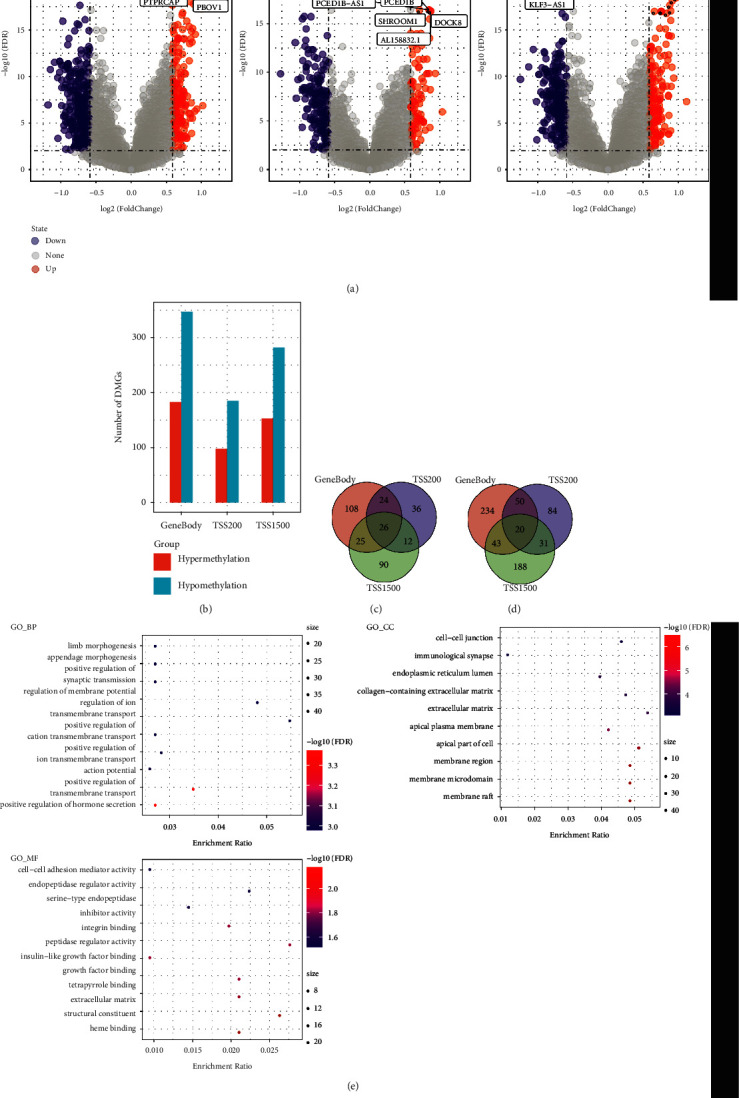
(a) The volcano plots of differential methylation in the gene body, TSS200, and TSS1500; (b) the histograms of differential methylation in three regions; (c) the Venn diagram of hypermethylation in three regions; (d) the Venn diagram of demethylation in three regions; and (e) the KEGG and GO functional enrichment analysis of differential methylation genes, where blue to red indicates FDR from large to small, and dots from small to large represent an increasing number of genes.

To identify differential methylation in gastric cancer, we compared methylation data from 188 PD-1 negative tumor samples and 187 PD-1 positive ones. In this research, we focused on two gene bodies, TSS200 and TSS1500. Throughout the three regions, we identified 971 differentially methylated genes (FDR < 0.05, | delta *β*-values | > 0.3). In the volcano map, as shown in Figure 2(a), there were 183 hypermethylated genes and 347 demethylated genes in the gene body region, 98 hypermethylation and 185 demethylations in the TSS200 region, and 153 hypermethylation and 282 demethylations in the TSS1500 region. We found that the number of hypermethylation was about twice higher than that of demethylation in all three regions, as shown in Figure 2(b). We can also see that 26 hypermethylated genes occurred in all three regions, while 61 were found in two regions, and 234 were only found in one region, as shown in Figure 2(c).

Twenty demethylated hypermethylated genes were present in all three regions, while 124 were present in two, and 506 were only present in one, as depicted in Figure 2(d), which confirms that methylation was related to the region. To explore these DMGs' roles, we analyzed the GO functional enrichment and KEGG pathway. There were 335 biological processes, 24 cellular processes, and 19 molecular functions, as depicted in Figure 2(e), which illustrates the regulation of ion transmembrane transport, collagen-containing extracellular matrix, cell-cell junction, and peptidase regulator activity.

### 3.3. Analysis of DEGs

**Figure 3:**
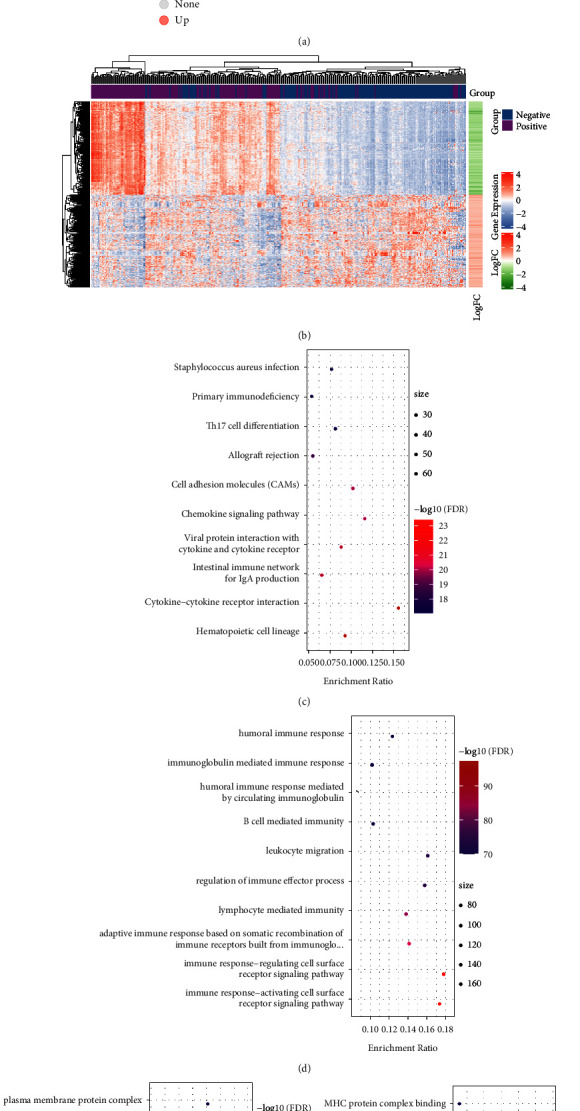
(a) The volcano plots of DEGs, (b) the heat map of DEGs, (c) the KEGG enrichment result of DEGs, (d) the GO BP enrichment result of DEGs, (e) the GO CC enrichment result of DEGs, and (f) the GO MF enrichment result of DEGs. The colors of CDEF, from blue to red, represent the FDR from large to small; the dots' sizes represent the enrichment result of the number of genes, while dots from small to large represent an increasing number of genes.

We used the limma package to analyze the DEGs between 188 PD-1 negative and 187 PD-1 positive samples. We finally obtained 1,189 differentially expressed genes, out of which 986 were downregulated, while 203 were upregulated (FDR < 0.01, | FC | > 1.5). The volcano map of the DEGs is depicted in Figure 3(a). Through an unsupervised hierarchical cluster analysis of these DEGs, we found that differentially expressed genes could distinguish tumor samples from normal ones, as illustrated in Figure 3(b). We performed the KEGG pathway and GO enrichment analyses of down- and upregulated genes using the clusterProfiler R software package. These genes were enriched into 51 KEGG pathways, 54 cellular components, 754 biological processes, and 68 molecular functions. As illustrated in Figure 3(c), the most prominent of these pathways included cytokine-cytokine receptor interaction, S, viral protein interaction with cytokine and cytokine receptor, and intestinal immune network for IgA production, which is known to influence the development of gastric cancer. The result of the GO enrichment analysis of the DEGs is illustrated in Figures 3(d)–3(f).

### 3.4. Joint Analysis of DEGs and Methylated Genes

**Figure 4:**
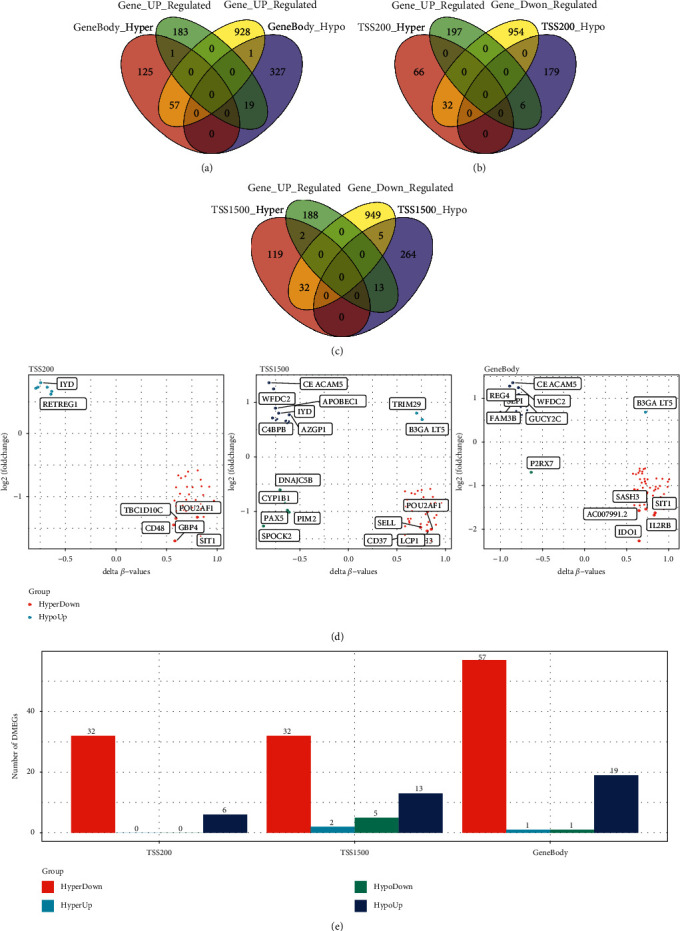
(a) The Venn diagram of DEGs and DMGs in the GeneBody region; (b) the Venn diagram of DEGs and DMGs in the TSS200 region; (c) the Venn diagram of DEGs and DMGs in the TSS1500 region; (d) the quadrant plots of DEGs and DMGs genes in the TSS200, TSS1500, and GeneBody regions; and (e) the histogram of four regulation modes of DEGs and DMGs in the TSS200, TS1500, and GeneBody regions.

We examined DEGs and DMGs in three different regions (gene body, TSS200, and TSS1500), and to further explore these genes' impact on stomach cancer, we determined the intersection of the DEGs and DMGs as DMEGs (differentially methylated and expressed genes), which were believed to play a more determining role in promoting or inhibiting the development of gastric cancer. We obtained 78 DMEGs in the gene body region, as shown in Figure 4(a); 38 in the TSS200 region; and 52 in the TSS1500 region, as shown in Figures 4(b) and 4(c).  Figure 4(d) shows the differential methylation fold change and differential expression fold change of these DMEGs. As we can see, we obtained five genes with the greatest fold change of expression, among which some, such as POU2AF1 and IYD, can be found in different regions at the same time and may have a pertinent role in leading to the occurrence of gastric cancer. We counted DMEGs in three regions and identified a total of 114 DMEGs, including 80 in Hyperdown, 2 in HyperUp, 6 in HypoDwon, and 26 in HypoUp.

### 3.5. Analysis of DMEGs Genes

**Figure 5:**
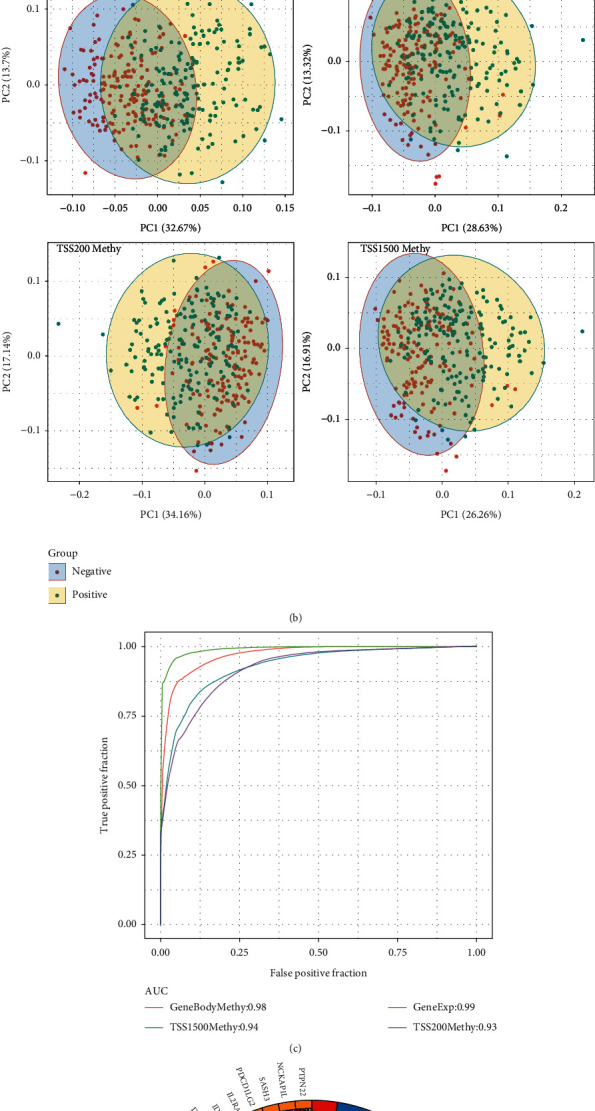
(a) The distribution of DMEGs in the genome, (b) the PCA analysis of gene expression and methylation of DMEGs, (c) the ROC curves of predicting tumor and normal samples based on the linear discriminant classification model constructed by the DMEGs gene expression profiles and methylation data, and (d) the KEGG pathway and GO enrichment analysis of DMEGs, in which distinct colors denote different pathways, and connections denote genes associated with pathways.

We identified 114 DMEGs and illustrated their distribution on chromosomes, as shown in Figure 5(a). There were 17 DMEGs on the chr1 chromosome and more than 8 on each chromosome of chr2, chr3, chr8, chr11, and chr12. Besides, we found that the methylation patterns of DMEGs were similar and consistent in similar gene regions. To explore the differences in gene expression and DNA methylation patterns between tumor and normal samples, we constructed a linear discriminant classification model using the DMEGs gene expression profiles and methylation data obtained from GeneBody, TSS200, and TSS1500 and carried out a PCA (principal component analysis) and ROC analysis (Figures 5(b) and 5(c), respectively). The PCA's results showed that both gene expression profiles and methylation data from different regions were able to classify the tumor and normal samples. We analyzed 114 DMEGs using the clusterProfiler R software to conduct the KEGG pathway and the GO enrichment analysis and enriched 184 biological pathways. We selected the 10 most significant ones, displayed in Figure 5(d).

### 3.6. Potential Target Therapeutic Drugs

We used the NetworkAnalyst 3.0 tool and DrugBank database to analyze the DMEGs gene for protein-drug interactions and found 10 genes that interacted with the drug, as shown in Table 2. At the same time, we thought that DMEGs were crucial genes related to gastric cancer, and drugs targeting these genes may have a greater impact on the occurrence of gastric cancer. Based on the description given in the methodology section, we generated a simulated reference distance distribution for the drug. We found that whether DMEGs or randomly selected gene sets are used as samples, the drug distance is concentrated in 1 to 2. We performed multiple hypothesis tests based on the random data and selected the small distance and FDR. A total of 26 drugs with <0.01, which were used as the candidate drug set related to the DMEGs gene set, were obtained through analysis (Supplementary Figure 1).

Based on the DMEGs obtained in step 6 and the candidate drug set, we selected the intersection and obtained the table shown above. We selected the AP-22408 with a significant distance of Lck to the gene set.

**Figure 6:**
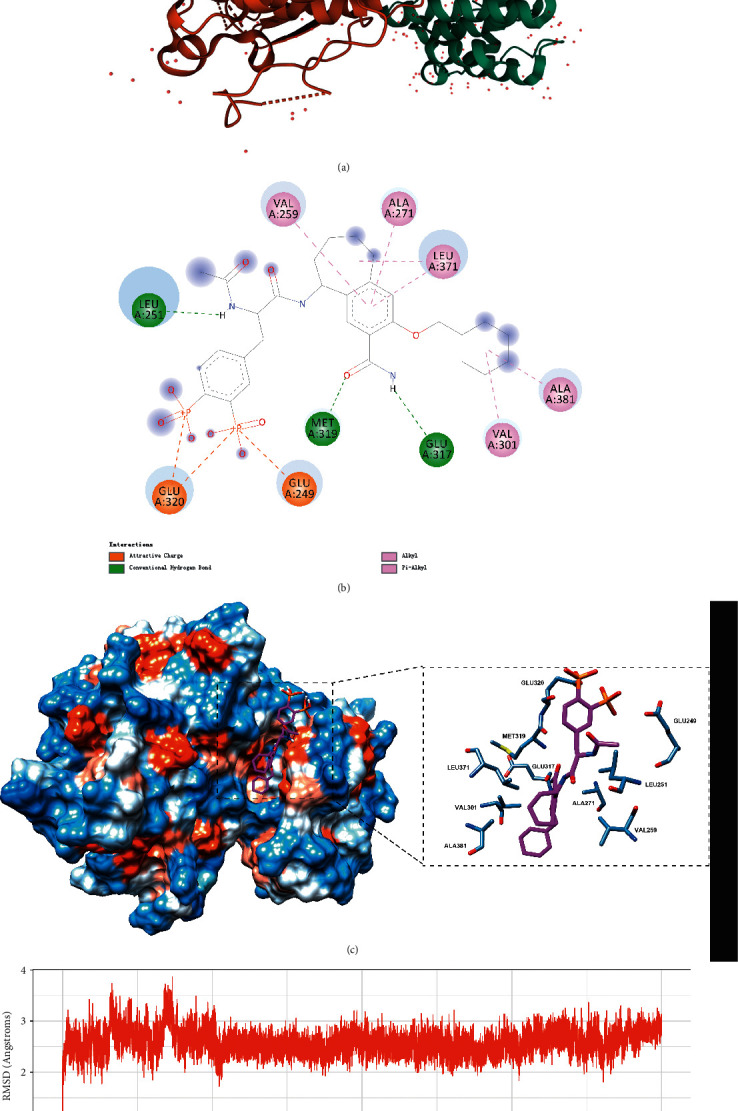
(a) 3D structure of the LCK protein, (b) binding diagram of the LCK protein to the compound DB01830, (c) 2D interaction diagram of the LCK protein with the compound DB01830, and (d) RMSD value of the compound DB01830 during 100 ns molecular dynamics simulation. Note: The amino acid residues in the protein were shown as steel blue sticks, and the heteroatoms on the residues were shown by element type. The compound DB01830 was displayed as a magenta stick.

We took LCK-AP-22408 as an example of molecular docking analysis to clarify the binding model between the candidate drug and the target. We first searched the PDB database and downloaded the LCK protein to the docking experiments and the 3D structure shown in Figure 6(a). With Autodock Vina, we analyzed the binding pattern of the target and the candidate drug. The results showed that the compound DB01830 could bind well to the active site of the LCK protein with a docking score of –9.2 kcal/mol and produced favorable hydrogen bonding with the amino acid residues LEU251, GLU317, and MET319 of the LCK protein. It also had hydrophobic interaction with VAL259, ALA271, LEU371, VAL301, and ALA381, as well as S-Π interaction with GLU320 and GLU249, as shown in Figures 6(b) and 6(c). These important interactions suggested, to some extent, that the compound can closely bind to the LCK protein. In addition, Figure 6(d) showed the conformational changes of the compound DB01830 bound to the LCK protein during the 100 ns molecular dynamics simulation. The conformations of this compound remained relatively stable and generally lower than 3 Å, except for slight fluctuations in the first 25 ns. These results indicate that the compound DB01830 could bind to the LCK protein stably. Therefore, this compound may be a potential inhibitor of the LCK protein.

### 3.7. Establishing the Prognostic Gene Signatures Related to the DMEGs

**Figure 7:**
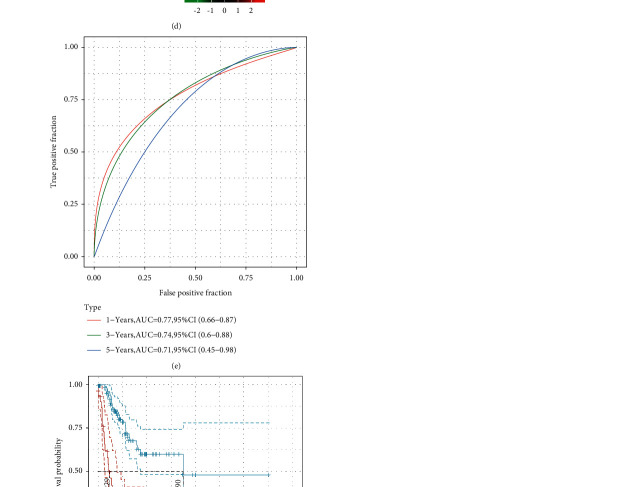
(a) The frequency of 1,000 lasso regression in each gene combination; (b) the variation coefficients trajectory of each gene with different lambda; (c) the standard deviation distribution of models with different lambda; (d) the survival time and status, RiskScore, and expression of 10 genes in the training set; (e) the AUC and ROC curve of 10-gene signature categories in the training set; and (f) the KM 10-gene signature survival distribution curve in the training set.

To examine the relationship between DMEGs and prognosis, we randomly grouped 188 PD-1 negative samples into a validation set (*N* = 47) and a training set (*N* = 141) by a ratio of 1:3. For the 114 DMEGs gene expression and clinical survival data, we used tenfold cross-validation, performed 1,000 times lasso regression analysis in the training set, summarized the dimensionality reduction results, and counted the number of each probe appearing for each of the 1,000 times (Figure 7(a)). Ten genes can be observed for a combination of the maximum occurrence frequency (Table 3). These 10 genes, with different lambda variation coefficient trajectories, are depicted in Figure 7(b), and different lambda standard deviation distributions can be seen in Figure 7(c). Finally, the KM curve analysis showed that these genes could differentiate the low- and high-risk groups.

Finally, we obtained the following risk score formula:

(3) $$\begin{array}{c} \text{RiskScore}=+0.621*\text{SELL}+0.247*\text{EOMES}-0.028*IYD\\ +0.086*RP11-44K6.2-0.4*JPH1-0.084*\text{TRIM}29+0.348*\text{PCED}1B\\ -0.045*\text{TSPAN}8-0.084*\text{CEACAM}5-0.944*PPP1R16B. \end{array}$$

We calculated the RiskScore for each sample according to their expression levels and arranged the distribution of this RiskScore, as illustrated in Figure 7(d). Furthermore, we applied the R software package timeROC to analyze prognostic categorization efficiency for 1 year, 3 years, and 5 years, as illustrated in Figure 7(e). The area under the ROC curve (AUC) from the model was quite high, with the 5-year AUC above 0.7. We also performed a *z*-score for the RiskScore and used the R software to determine the cut-off value. We used Maxistat to group low- and high-risk samples and plotted the KM curve, as indicated in Figure 7(f). The results revealed a significant difference between the two samples with log rank *P* > 0.0001. Among those, 35 samples were categorized within the high-risk group, while 105 were classified within the low-risk group. However, one of the samples was missing survival information.

**Figure 8:**
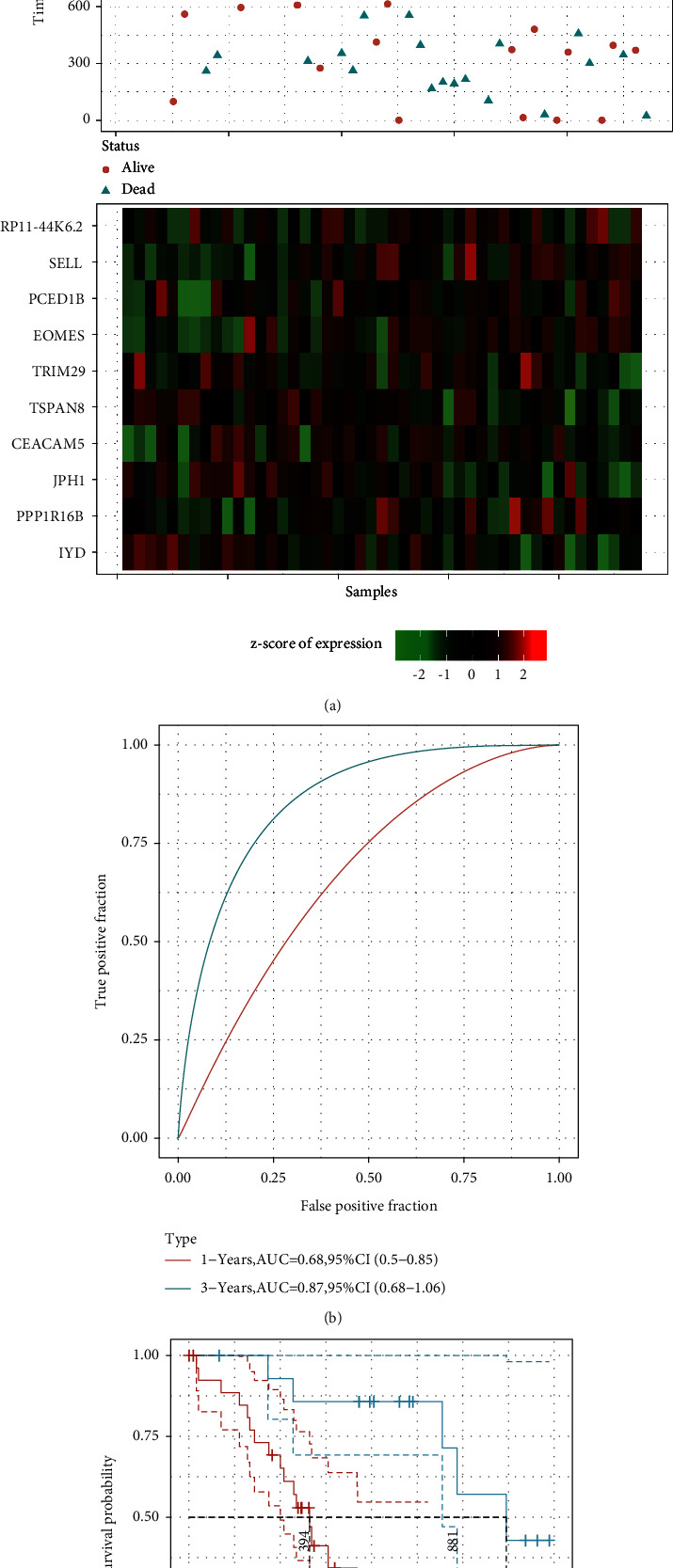
(a) The survival status and time, RiskScore, and expression of 10 genes in the training set; (b) the AUC and ROC curve of 10-gene signature categories in the training set; and (c) the KM 10-gene signature survival distribution curve in the training set.

To evaluate the 10-gene signature's predictive value, we applied the same models and constants to those used for the training set and verification set. Similarly, we calculated the RiskScore for each sample in relation to their expression level and arranged the RiskScore distribution, as illustrated in Figure 8(a). Moreover, we performed the analysis of the ROC of the prognostic categorization efficiency for 1 year, 3 years, and 5 years, as illustrated in Figure 8(b). The AUC of 3 years was above 0.87. We also conducted a *z*-score for RiskScore and determined the cut-off value using the R software package Maxistat to group the samples into a low-risk group and a high-risk group and plotted the KM curve, as depicted in Figure 8(c). A significant difference was observed between the two groups with log rank *P* = 0.0067 and HR = 2.48. Among those, 30 samples were grouped into the high-risk group, while 15 of them were classified into the low-risk group. However, two of the samples were missing survival information.

**Figure 9:**
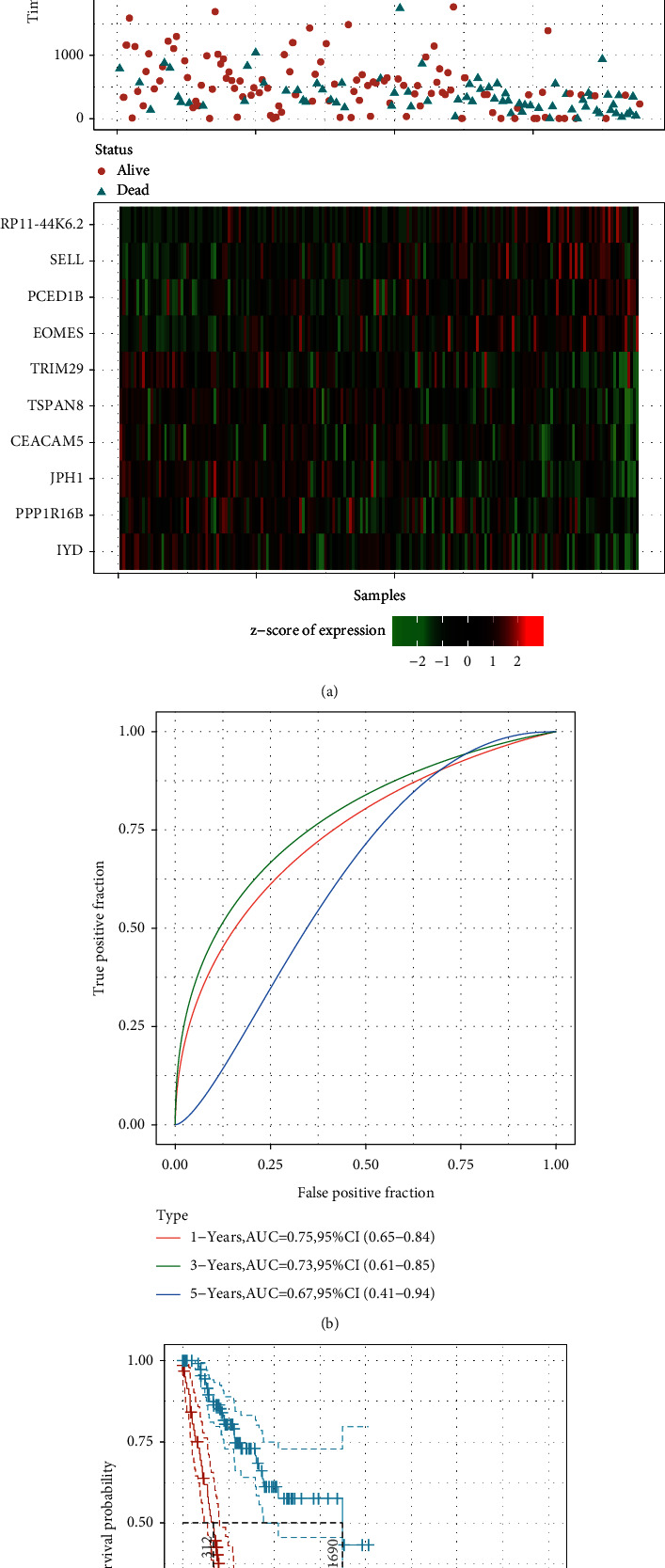
(a) The survival time and status, RiskScore, and expression of 10 genes in the PD-I negative samples from TCGA; (b) the AUC and ROC curve of 10-gene signature categories in the verification set; and (c) the KM 10-gene signature survival distribution curve in the PD-1 negative samples from TCGA.

To evaluate the 10-gene signature's predictive value, we applied similar models and coefficients to those used in the training set to the PD-1 negative sample from TCGA. Similarly, we calculated each sample's RiskScore according to their expression level and plotted the RiskScore distribution, as depicted in Figure 9(a). It is evident that the group with a high RiskScore had relatively shorter OS compared to that with a low RiskScore, indicating that high-RiskScore samples had a poorer prognosis. Moreover, we performed the analysis of the ROC of prognostic categorization efficiency for 1 year, 3 years, and 5 years, as illustrated in Figure 9(b). The 3-year AUC was 0.73, while that of 5 years was 0.67. We also conducted a *z*-score to evaluate the RiskScore, determined the cut-off value using the R software package Maxistat to group the samples into a low-risk group and a high-risk group, and plotted the KM curve, as depicted in Figure 9(c). We noticed a significant difference between the two groups with log rank *P* > 0.0001, while HR = 2.48. Among those, 66 samples were classified into the high-risk group, while 119 were categorized into the low-risk group. However, three of the samples were missing survival information.

**Figure 10:**
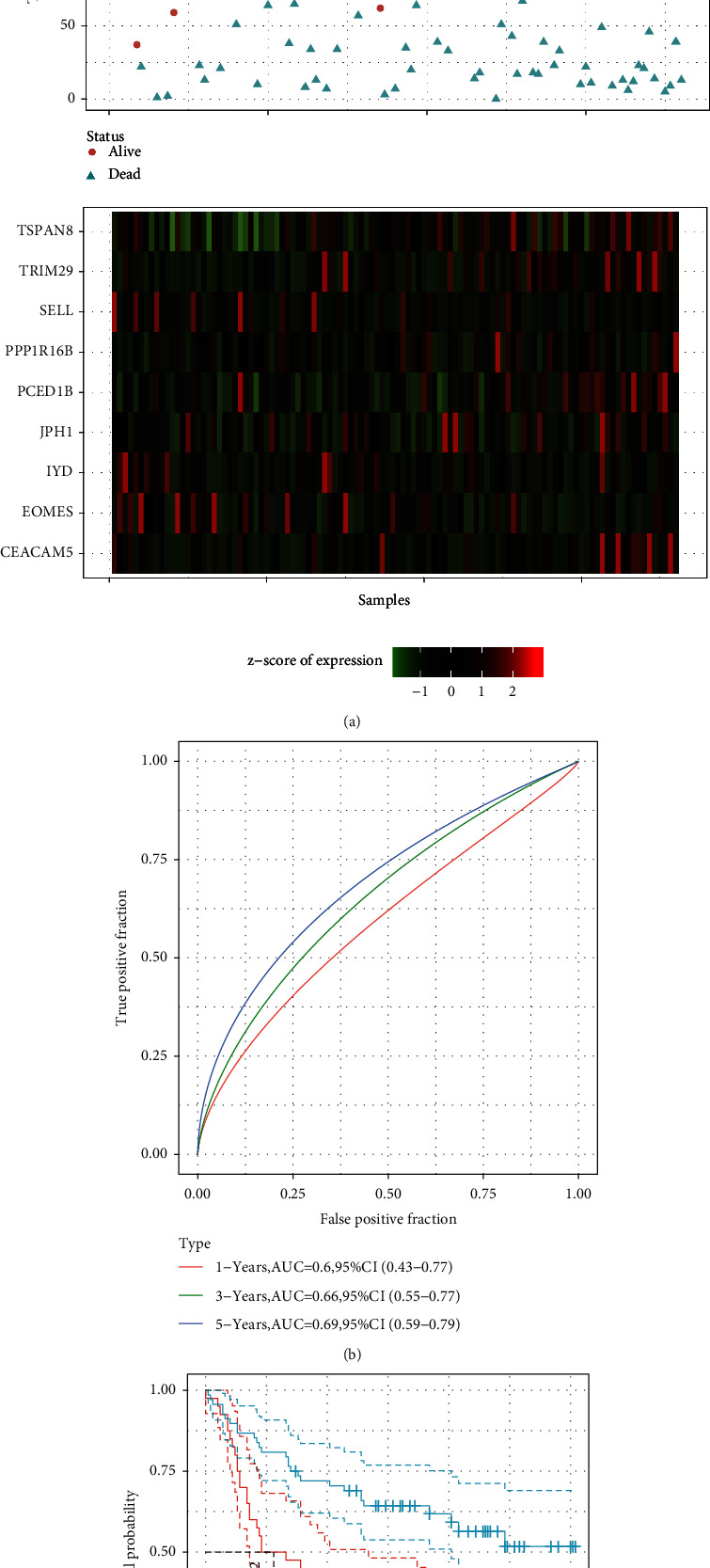
(a) The survival time and status, RiskScore, and expression of 10 genes in the PD-I negative samples from GSE84437; (b) the AUC and ROC curve of 10-gene signature categories in the verification set; (c) the KM 10-gene signature survival distribution curve in GSE84437.

To evaluate the 10-gene signature's predictive value, we applied similar models and coefficients to those used in the training set for GSE84437. Considering the RP11-44K6.2 gene was not examined in the GEO data set, we obtained the expression profiles of nine genes that had been detected and used the same methods to develop a prognostic risk model to assess patients' prognoses. Based on the results of the proportion of PD-1 negative obtained in outcome 1, we found that the expression of PD-1 in the normal samples was approximately equal to the quartiles of the expression of PD-1 in the gastric cancer samples. So we took the lowest one-quarter from the 433 samples of GSE84437, namely, the 108 samples with the lowest PD-1 expression. Each sample's RiskScore was calculated according to their expression level, and the RiskScore distribution was plotted, as depicted in Figure 10(a). It is evident that the group with a high RiskScore had relatively shorter OS than the one with a low RiskScore, indicating that high-RiskScore samples had a poorer prognosis. The expression of nine distinct signature genes rose with the increase of the RiskScore. Furthermore, we used the R software timeROC to analyze the ROC of RiskScore prognostic categories. We performed the analysis of the ROC of prognostic categorization efficiency for 1 year, 3 years, and 5 years, as illustrated in Figure 10(b). The 5-year AUC was 0.69. We also conducted a *z*-score for the RiskScore and determined the cut-off value using the R software package Maxistat to group the samples into a low-risk group and a high-risk group. We also plotted the KM curve, as depicted in Figure 10(c). We observed a significant difference between the two groups with log rank *P* > 0.00011, while HR = 1.75. Among those, 40 samples were regrouped into the high-risk group, while 68 were classified into the low-risk group.

## 4. Discussion

Gastric cancer is the most common cancer that affects the gastrointestinal system and is ranked as the second leading cause of cancer-related death in the world [19]. Multiple gene methylation is closely linked to the development and occurrence of gastric cancer. In this study, we combined and analyzed two different types of PD-1 negative gastric cancer gene chips using a bioinformatics analysis tool, to reveal the epigenetic and genetic mechanisms of gastric cancer. We found some hub genes, which provided new ideas for the diagnosis and treatment of gastric cancer.

We identified 971 DMGs using 188 PD-1 negative tumor samples and 187 PD-1 positive samples of gastric cancer in TCGA. The results of the KEGG and GO function enrichment analysis indicated that the DMGs were linked to biological processes, including the regulation of ion transmembrane transport, collagen-containing extracellular matrix, cell-cell junction, peptidase regulator activity, and so on. At the same time, we obtained 1,189 DEGs, out of which 986 were downregulated, while 203 were upregulated. The GO enrichment and KEGG pathways examination indicated that the most significant top pathways included the cytokine-cytokine receptor interaction, *Staphylococcus aureus* infection, viral protein interaction with cytokine and cytokine receptor, and intestinal immune network for IgA production, which has been linked with gastric cancer. DMEGs were chosen from the intersection of the two sets, suggesting that such genes may play a greater role in promoting or inhibiting the development of gastric cancer. Some genes, such as POU2AF1 and IYD, can be seen in different regions. POU domain class 2-associating factor 1 (POU2AF1) was a known B-cell transcription coactivator. This gene was both expressed in lymphocyte cells and the whole-genome RNA sequencing of human airway epithelial cells. POU2AF1, as well as its related pathways, may be a therapeutic target for smoking-related airway diseases [20]. This gene promoted multiple myeloma through amplification or other mechanisms as an oncogene [21]. The functional polymorphism of the POU2AF1 gene 3′-UTR was linked with an increased predisposition to lymphoma [22]. In addition, it has been known for being present in systemic autoimmune diseases, including multiple sclerosis and rheumatoid arthritis [23, 24]. Iodotyrosin deiodinase (IYD) is an essential thyroid hormone enzyme for the iodination homeostasis invertebrates, which enables the effective synthesis of thyroid hormone [25, 26]. We found a specific mutation of IYD in patients with abnormal iodine metabolism and congenital hypothyroidism [27–30]. Currently, there is no tumor-related research focusing on these genes. As newly discovered gene targets, POU2AF1 and IYD might have a pertinent function in the development of stomach cancer and have yet to be further explored.

We have identified 114 DMEGs and noticed the methylation patterns of these DMEGs were similar and consistent in close gene regions. We constructed a linear decision classification model based on DMEGs. PCA results showed that both gene expression profiles and methylation data from different regions were able to separate normal and tumor samples with high accuracy. To clarify the binding model between the candidate drug and the target, we used LCK-AP-22408 as an example to study the docking analysis. The results showed that the compound DB01830 could bind well to the active site of the LCK protein, which shows good stability and may be a potential inhibitor of the LCK protein. To observe the relationship between DMEGs expression and prognosis, we obtained 10 genes—TRIM29, TSPAN8, RP11-44K6.2, EOMES, PPP1R16 B, SELL, PCED1B, IYD, JPH1, and CEACAM5—according to the expression and clinical survival data of 114 DMEGs.

TRIM29 was reported to have different effects on different types of tumors. For instance, its overexpression showed a poor prognosis in gastric cancer and the progression of a tumor, as well as bowel, pancreatic, bladder, lung, pancreatic, liver, thyroid, endometrial, and ovarian cancers [31–38]. However, TRIM29 appeared to have an inhibitory effect on other tumors, including breast and prostate cancers [39, 40]. A meta-analysis has been conducted to clarify the predictive value of TRIM29 for different human malignant diseases. The study eventually included 2,046 eligible patients, and the results suggested an important relationship between TRIM29's expression upregulation and a poor prognosis in patients with malignant tumors [40]. Overall, TRIM29 may have a pertinent function in the carcinogenesis of various human malignant tumors and be a useful biomarker for predicting the prognosis of patients.

TSPAN8 was a member of the TSPAN superfamily and played a crucial function in regulating leukocyte transport, wound repair, and angiogenesis in the physical-biological progress [41]. Over the last few years, an increasing amount of studies have shown that the overexpression of TSPAN8 is determining metastasis and the development of various tumors. [42–45] The specific targeting of monoclonal antibodies in Span8, as an emerging cancer therapeutic target, is becoming increasingly important in cancer therapy [46].

Eomesodermin (EOMES) is a T-box transcription factor that promoted the differentiation and function of cytotoxic lymphocytes, regulated natural killer (NK) cells mediating antiviral and antitumor responses, and participated in inducing the exhaustion of CD8+ T cells [47–50]. Eomes had different effects on different types of tumors. A high expression of Eomes was linked to short overall survival in patients with colorectal cancer [51]. However, it was also considered an independent good prognostic factor in patients with metastatic renal cell carcinoma [52]. The level of Eomes methylation was also closely related to the tumor. EOMES showed tumor-specific DNA hypermethylation in people with advanced bladder cancer [53, 54]. In addition, the aberrant methylation of the Eomes gene promoter region resulted in its downregulation and hepatocellular carcinoma initiation and progression [55]. These studies showed that the occurrence and development of malignant tumors were closely related to the development of malignancies. In 133 cases of esophageal squamous cell carcinoma (ESCC), higher Eomes levels were associated with a poor clinical prognosis [56]. Eomes hypermethylation may provide an effective approach in the diagnosis of ESCC patients [57].

PCED1B-AS1 increased significantly in glioblastomas (GBM) tissues and was closely related to the tumor's grade and size. The high PCED1B-AS1 survival time was shorter compared to that of the low PCED1B-AS1 group. Practical experiments illustrated that PCED1B-AS1's gene silencing repressed the proliferation of glioma cells and induced apoptosis, indicating that PCED1B-AS1 provided an auspicious biomarker for the prognosis, as well as drug targets of glioma [58, 59]. It has been demonstrated that PCED1B-AS1 can promote the proliferation, migration, and epithelial to mesenchymal transition (EMT) process, thus promoting the progression of clear renal cell carcinoma [60]. At the same time, PCED1B-AS1 can increase the function and expression of PD-L1 in hepatocellular carcinoma and induce the apoptosis of immunosuppressive cells [61].

Carcinoembryonic antigen-associated cell adhesion molecule 5 (CEACAM5), a highly glycosylated protein of the CEACAM family, increased its expression in the human breast, stomach, colorectal, and non-small-cell lung carcinoma cancers by promoting the proliferation and migration of cancer cells to promote the progression of the tumor [62–65]. CEACAM5 was recognized as a tumor biomarker and an indicator of cancer recurrence. CEACAM5 might become a potential target for a variety of cancer therapies.

The results obtained in this study may be limited considering all the genes and pathways were based on the bioinformatics approach. As a result, this research lacks clinical samples that would have been useful to validate the data obtained. Moreover, we did not examine clinical gene expression profiles and clinical gene methylation data in this study. Therefore, to enhance the reliability of our results, we need to conduct additional experiments, including a wide range of animal and cell experiments in future work.

## 5. Conclusions

In short, this study used the bioinformatics method to perform a combined analysis of PD-1 negative gastric cancer, using a gene methylation microarray and a gene expression microarray. This helped provide better insights into the molecular mechanism and pathogenesis of gastric cancer. We found out that the hub genes might represent molecular targets and diagnostic markers for accurate diagnosis and effective treatment of gastric cancer. Our study has also revealed a wealth of gastric cancer-specific signatures, many of which may serve as drug targets and probable biomarkers for clinical applications in the future.

## Figures and Tables

**Table 1:** Grouping criteria of DMEGs.

Groups	Methylation cut-off	Expression cut-off
HypoUp	FDR < 0.01 and delta *β*-value < −0.3	FDR < 0.01 and log2FC > 1
HypoDown	FDR < 0.01 and delta *β*-value < −0.3	FDR < 0.01 and log2FC < −1
HyperUp	FDR < 0.01 and delta *β*-value > 0.3	FDR < 0.01 and log2FC > 1
HyperDown	FDR < 0.01 and delta *β*-value > 0.3	FDR < 0.01 and log2FC < −1

**Table 2:** Molecular docking.

Gene	Type	DrugCount	Vals
CD79 B	HyperDown	1	Polatuzumab vedotin
CEACAM5	HypoUp	2	Labetuzumab, S-[(1-hydroxy-2,2,5,5-tetramethyl-2,5-dihydro-1h-pyrrol-3-yl)methyl] methanesulfonothioate
GUCY2C	HypoUp	1	Linaclotide
Ido1	HyperDown	1	Nitric oxide
IL2RA	HyperDown	3	Denileukin diftitox, Aldesleukin, Basiliximab
IL2RB	HyperDown	3	Denileukin diftitox, Aldesleukin, Basiliximab
ITGA4	HyperDown	4	ATL1102, CDP323, R411
LCK	HyperDown	9	AP-22408, 1-tert-butyl-3-(4-chloro-phenyl)-1h-Pyrazolo[3,4-D]Pyrimidin-4-Ylamine, {4-[(2S)-2-acetamido-3-({(1S)-1-[3-carbamoyl-4-(cyclohexylmethoxy)phenyl]ethyl}amino)-3-oxopropyl]-2-phosphonophenoxy}acetic acid
PIK3CD	HyperDown	5	TG100-115, 2-((9h-PURIN-6-ylthio)METHYL)-5-CHLORO-3-(2-methoxyphenyl)QUINAZOLIN-4(3H)-ONE, Idelalisib

**Table 3:** Coefficients and confidence intervals of 10 genes.

Gene symbol	*p*-value	HR	Low 95% CI	High 95% CI
SELL	0.014331	1.332837	1.059037	1.677425
EOMES	0.021994	1.510369	1.061306	2.149441
IYD	0.085053	0.836981	0.683527	1.024886
RP11-44K6.2	0.006019	1.51889	1.127189	2.046708
JPH1	0.010396	0.73733	0.584031	0.930868
TRIM29	0.052902	0.868474	0.752917	1.001768
PCED1B	0.053152	1.423409	0.995213	2.035839
TSPAN8	0.028584	0.835056	0.710604	0.981304
CEACAM5	0.045096	0.907382	0.825093	0.997878
PPP1R16 B	0.251053	0.830685	0.605155	1.140266

## Data Availability

The data used to support the findings of this study can be obtained from the corresponding author upon reasonable request.

## Supplementary Materials

Supplementary Materials Supplementary Figure 1. The distance density distribution map from the drug to the DMEGs gene set. Supplementary Table 1. Randomly selected gene set. Supplementary Table 2. Twenty-six drugs with small distances.s
