# Supplementary material for: Identification of Signature Genes in the PD-1 Relative Gastric Cancer Using a Combined Analysis of Gene Expression and Methylation Data
Source: J Oncol. 2022 Dec 15;2022:4994815. doi: 10.1155/2022/4994815 (PMC9780002; doi:10.1155/2022/4994815)
Supplement: Supplementary Materials — Supplementary Figure 1. The distance density distribution map from the drug to the DMEGs gene set. Supplementary Table 1. Randomly selected gene set. Supplementary Table 2. Twenty-six drugs with small distances.s [file 4994815.f1.docx]

**
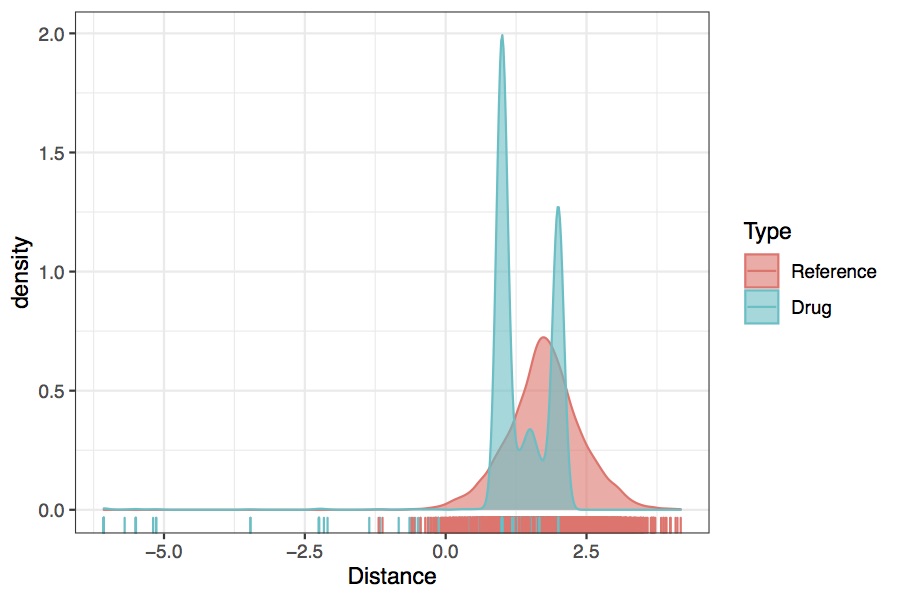
**

Supplement Figure 1. the distance density distribution map from drug to the DMEGs gene set.

**Supplement Table 1. Randomly selected gene set**

drug_id distances random_dis Mean SD pvalue FDR global_pvalue global_FDR

DB00001 1 3.295858654 1.75379047 0.753349628 0.1585137 1 0.12201228 1

DB00002 1 1.585653643 1.770629523 0.307115021 0.006049327 1 0.12201228 1

DB00004 -3.4657334 1.927406839 1.757244872 0.464542708 1.25E-29 6.86E-26 4.52E-16 2.48E-12

DB00005 1 1.614895193 1.761473636 0.259363203 0.001662717 1 0.12201228 1

DB00006 1 1.020971354 1.75379047 0.753349628 0.1585137 1 0.12201228 1

DB00007 1 2.674412389 1.75379047 0.753349628 0.1585137 1 0.12201228 1

DB00008 1.5 2.148961764 1.745287633 0.542671379 0.325634379 1 0.346299761 1

DB00009 1 1.3082654 1.757244872 0.464542708 0.051541591 1 0.12201228 1

DB00010 2 2.246358696 1.75379047 0.753349628 0.628097886 1 0.645919359 1

DB00011 1.5 1.134754596 1.745287633 0.542671379 0.325634379 1 0.346299761 1

DB00012 1 1.203127597 1.75379047 0.753349628 0.1585137 1 0.12201228 1

DB00013 1.4285714 1.60871195 1.736866356 0.292454388 0.145903866 1 0.306680831 1

DB00014 1.5 2.332624707 1.745287633 0.542671379 0.325634379 1 0.346299761 1

DB00015 1 2.572683757 1.745287633 0.542671379 0.08481895 1 0.12201228 1

DB00016 1 1.631928639 1.75379047 0.753349628 0.1585137 1 0.12201228 1

DB00018 1.5 1.661507945 1.745287633 0.542671379 0.325634379 1 0.346299761 1

DB00019 1 2.184981316 1.75379047 0.753349628 0.1585137 1 0.12201228 1

DB00020 1.2 2.122145288 1.757979426 0.337271213 0.049023756 1 0.195688273 1

DB00022 1.5 2.73746333 1.745287633 0.542671379 0.325634379 1 0.346299761 1

DB00025 1.5454545 1.379059624 1.759747174 0.221112233 0.166233199 1 0.372453903 1

DB00026 1 0.569443602 1.75379047 0.753349628 0.1585137 1 0.12201228 1

DB00028 1 2.011115545 1.736866356 0.292454388 0.005874511 1 0.12201228 1

DB00029 1 1.941127853 1.745287633 0.542671379 0.08481895 1 0.12201228 1

DB00030 1.3333333 1.737412408 1.757244872 0.464542708 0.180743525 1 0.257238652 1

DB00031 1.2 1.983295039 1.759693587 0.247672276 0.01191636 1 0.195688273 1

DB00032 2 1.598988357 1.75379047 0.753349628 0.628097886 1 0.645919359 1

DB00033 2 1.742848545 1.745287633 0.542671379 0.680596551 1 0.645919359 1

DB00034 1.5 3.124479294 1.745287633 0.542671379 0.325634379 1 0.346299761 1

DB00035 1 2.219523833 1.745287633 0.542671379 0.08481895 1 0.12201228 1

DB00036 1.8 1.601788466 1.757979426 0.337271213 0.549575873 1 0.52649583 1

DB00038 2 2.24055455 1.75379047 0.753349628 0.628097886 1 0.645919359 1

DB00039 1.1666667 1.70012531 1.770629523 0.307115021 0.024616471 1 0.181824474 1

DB00040 1.6666667 1.8870568 1.757244872 0.464542708 0.422702925 1 0.444812957 1

DB00041 -3.4657334 1.370917897 1.757244872 0.464542708 1.25E-29 6.86E-26 4.52E-16 2.48E-12

DB00042 2 1.328157692 1.757244872 0.464542708 0.699362524 1 0.645919359 1

DB00043 1 2.2168367 1.745287633 0.542671379 0.08481895 1 0.12201228 1

DB00044 2 -0.594319218 1.75379047 0.753349628 0.628097886 1 0.645919359 1

DB00045 1 0.859109795 1.75379047 0.753349628 0.1585137 1 0.12201228 1

DB00046 1 2.178943677 1.75379047 0.753349628 0.1585137 1 0.12201228 1

DB00047 1 1.898830556 1.75379047 0.753349628 0.1585137 1 0.12201228 1

DB00048 1.25 1.493559235 1.753692879 0.367879736 0.085471804 1 0.217648936 1

DB00050 1.5 1.62632167 1.745287633 0.542671379 0.325634379 1 0.346299761 1

DB00051 1 1.314221084 1.75379047 0.753349628 0.1585137 1 0.12201228 1

DB00052 1 2.309658478 1.75379047 0.753349628 0.1585137 1 0.12201228 1

DB00054 1 1.868166377 1.757979426 0.337271213 0.012307606 1 0.12201228 1

DB00055 1.1111111 1.933878839 1.753207263 0.264393406 0.007579386 1 0.160123241 1

DB00056 1 2.615736034 1.745287633 0.542671379 0.08481895 1 0.12201228 1

DB00058 1 1.026391431 1.75379047 0.753349628 0.1585137 1 0.12201228 1

DB00060 1.5 1.07414067 1.745287633 0.542671379 0.325634379 1 0.346299761 1

DB00061 1 1.478714155 1.75379047 0.753349628 0.1585137 1 0.12201228 1

DB00063 1 1.909160311 1.75379047 0.753349628 0.1585137 1 0.12201228 1

DB00065 1 2.535899993 1.75379047 0.753349628 0.1585137 1 0.12201228 1

DB00067 1 2.350112359 1.745287633 0.542671379 0.08481895 1 0.12201228 1

DB00068 1.5 1.162529104 1.745287633 0.542671379 0.325634379 1 0.346299761 1

DB00069 1.5 2.569058389 1.745287633 0.542671379 0.325634379 1 0.346299761 1

DB00071 1.5454545 1.579153016 1.759747174 0.221112233 0.166233199 1 0.372453903 1

DB00072 1 0.951918957 1.75379047 0.753349628 0.1585137 1 0.12201228 1

DB00073 1 1.330093144 1.75379047 0.753349628 0.1585137 1 0.12201228 1

DB00074 -5.6986002 1.618506962 1.745287633 0.542671379 4.01E-43 2.20E-39 8.68E-31 4.76E-27

DB00075 1 1.615068009 1.753692879 0.367879736 0.020243381 1 0.12201228 1

DB00078 1 1.428316586 1.75379047 0.753349628 0.1585137 1 0.12201228 1

DB00081 1 1.273587855 1.745287633 0.542671379 0.08481895 1 0.12201228 1

DB00083 1 1.16150024 1.745287633 0.542671379 0.08481895 1 0.12201228 1

DB00086 1 2.656076775 1.745287633 0.542671379 0.08481895 1 0.12201228 1

DB00087 -0.5397638 2.18324794 1.753692879 0.367879736 2.27E-10 1.24E-06 0.000203761 1

DB00089 1 1.8552199 1.75379047 0.753349628 0.1585137 1 0.12201228 1

DB00091 1.25 1.669587404 1.753692879 0.367879736 0.085471804 1 0.217648936 1

DB00092 1 1.906698419 1.75379047 0.753349628 0.1585137 1 0.12201228 1

DB00093 1 0.253875683 1.75379047 0.753349628 0.1585137 1 0.12201228 1

DB00095 1 1.135132915 1.75379047 0.753349628 0.1585137 1 0.12201228 1

DB00097 2 2.161475505 1.75379047 0.753349628 0.628097886 1 0.645919359 1

DB00098 1.1111111 1.902546056 1.753207263 0.264393406 0.007579386 1 0.160123241 1

DB00099 1 1.701814269 1.745287633 0.542671379 0.08481895 1 0.12201228 1

DB00100 1.7142857 1.407628486 1.736866356 0.292454388 0.4692279 1 0.473897096 1

DB00102 1 1.891327681 1.757244872 0.464542708 0.051541591 1 0.12201228 1

DB00105 1.5 1.508151934 1.745287633 0.542671379 0.325634379 1 0.346299761 1

DB00106 1 1.184573277 1.75379047 0.753349628 0.1585137 1 0.12201228 1

DB00107 1 1.603550988 1.745287633 0.542671379 0.08481895 1 0.12201228 1

DB00108 -1.1670861 1.34894454 1.757244872 0.464542708 1.54E-10 8.39E-07 3.38E-06 0.018494281

DB00110 1 1.987728672 1.770629523 0.307115021 0.006049327 1 0.12201228 1

DB00111 -0.4885778 1.311958775 1.753207263 0.264393406 1.14E-17 6.22E-14 0.000273748 1

DB00112 1 1.693492945 1.770629523 0.307115021 0.006049327 1 0.12201228 1

DB00113 1 3.898789791 1.75379047 0.753349628 0.1585137 1 0.12201228 1

DB00114 1.6595745 1.960451979 1.759489714 0.107048152 0.175315253 1 0.440502749 1

DB00115 1.8 1.850395011 1.757979426 0.337271213 0.549575873 1 0.52649583 1

DB00116 1.8181818 1.672271438 1.759747174 0.221112233 0.604216321 1 0.537624777 1

DB00117 1.75 1.707084614 1.753692879 0.367879736 0.495995374 1 0.495810565 1

DB00118 1.625 1.806309901 1.761473636 0.259363203 0.299379022 1 0.419600475 1

DB00119 1.7142857 1.701922181 1.773663076 0.187305714 0.375618834 1 0.473897096 1

DB00120 1.7142857 1.636395156 1.736866356 0.292454388 0.4692279 1 0.473897096 1

DB00121 1.875 1.741601189 1.761473636 0.259363203 0.669202435 1 0.572174329 1

DB00122 1.5 2.082361929 1.761473636 0.259363203 0.156694354 1 0.346299761 1

DB00123 2 1.867802951 1.757979426 0.337271213 0.763493223 1 0.645919359 1

DB00125 1.7777778 1.800348485 1.753207263 0.264393406 0.537021102 1 0.512866521 1

DB00126 1.8 1.84838063 1.758534835 0.16776606 0.597608044 1 0.52649583 1

DB00127 1.5 2.100145621 1.770629523 0.307115021 0.189104995 1 0.346299761 1

DB00128 1.7058824 1.764128608 1.748450254 0.194078018 0.413195047 1 0.468750029 1

DB00129 1.8 1.671596176 1.759693587 0.247672276 0.564638785 1 0.52649583 1

DB00130 1.5 2.584759856 1.745287633 0.542671379 0.325634379 1 0.346299761 1

DB00131 1.1333333 2.068431634 1.760312929 0.204781867 0.001100438 1 0.168591892 1

DB00132 1.6 2.167879912 1.759693587 0.247672276 0.259535593 1 0.404623142 1

DB00133 1.7142857 1.496668197 1.736866356 0.292454388 0.4692279 1 0.473897096 1

DB00134 2 2.113419265 1.757979426 0.337271213 0.763493223 1 0.645919359 1

DB00135 1.5 1.635362046 1.753692879 0.367879736 0.245220308 1 0.346299761 1

DB00136 2 1.743203229 1.745287633 0.542671379 0.680596551 1 0.645919359 1

DB00138 2 0.922946353 1.745287633 0.542671379 0.680596551 1 0.645919359 1

DB00139 1.5 1.829311709 1.745287633 0.542671379 0.325634379 1 0.346299761 1

DB00140 1.5 1.267485041 1.745287633 0.542671379 0.325634379 1 0.346299761 1

DB00141 1.6666667 2.047536564 1.770629523 0.307115021 0.367487831 1 0.444812957 1

DB00142 1.6271186 1.656132323 1.762923433 0.093835335 0.073911006 1 0.42087541 1

DB00143 1.3888889 1.505316639 1.762980177 0.123919082 0.001268743 1 0.285572999 1

DB00144 1.7 1.337439785 1.759693587 0.247672276 0.40477041 1 0.46515008 1

DB00145 1.7272727 1.719365674 1.757638217 0.154827625 0.422256293 1 0.481859977 1

DB00146 2 1.384997757 1.75379047 0.753349628 0.628097886 1 0.645919359 1

DB00147 1 1.227494031 1.75379047 0.753349628 0.1585137 1 0.12201228 1

DB00148 2 1.646342767 1.770629523 0.307115021 0.772424541 1 0.645919359 1

DB00149 2 1.254497509 1.757979426 0.337271213 0.763493223 1 0.645919359 1

DB00150 1 0.897633185 1.745287633 0.542671379 0.08481895 1 0.12201228 1

DB00151 1.6 1.560782408 1.759693587 0.247672276 0.259535593 1 0.404623142 1

DB00152 2 1.816539955 1.75379047 0.753349628 0.628097886 1 0.645919359 1

DB00153 1.9230769 1.674221868 1.752565715 0.14361026 0.882449084 1 0.600992908 1

DB00154 1.5 1.605802326 1.745287633 0.542671379 0.325634379 1 0.346299761 1

DB00155 1.6666667 1.826430214 1.763093512 0.211303302 0.324071624 1 0.444812957 1

DB00156 2 1.12657255 1.745287633 0.542671379 0.680596551 1 0.645919359 1

DB00157 1.7785714 1.787549221 1.757113925 0.062757383 0.633791277 1 0.513353605 1

DB00158 1.3333333 1.77618444 1.757244872 0.464542708 0.180743525 1 0.257238652 1

DB00159 1.3333333 2.215643024 1.753207263 0.264393406 0.0561358 1 0.257238652 1

DB00160 1.6153846 1.824186323 1.754873752 0.200318376 0.243108818 1 0.413824896 1

DB00161 1.6666667 1.879394812 1.757244872 0.464542708 0.422702925 1 0.444812957 1

DB00162 1.8 1.719942075 1.760312929 0.204781867 0.576834417 1 0.52649583 1

DB00163 1.5555556 1.583670998 1.753207263 0.264393406 0.227360828 1 0.378351838 1

DB00165 1 2.246655133 1.75379047 0.753349628 0.1585137 1 0.12201228 1

DB00166 2 2.548204679 1.745287633 0.542671379 0.680596551 1 0.645919359 1

DB00167 2 2.003552083 1.757979426 0.337271213 0.763493223 1 0.645919359 1

DB00168 1 1.392274745 1.757244872 0.464542708 0.051541591 1 0.12201228 1

DB00169 2 1.068649903 1.75379047 0.753349628 0.628097886 1 0.645919359 1

DB00170 1.5 2.033859846 1.759693587 0.247672276 0.147195593 1 0.346299761 1

DB00171 1.5405405 1.82404896 1.752578353 0.121036105 0.039899267 1 0.369595348 1

DB00172 1.75 1.837958418 1.758534835 0.16776606 0.479713189 1 0.495810565 1

DB00173 1.8 1.816678163 1.757979426 0.337271213 0.549575873 1 0.52649583 1

DB00174 1.625 1.514869109 1.761473636 0.259363203 0.299379022 1 0.419600475 1

DB00175 1.5 1.512266872 1.745287633 0.542671379 0.325634379 1 0.346299761 1

DB00176 1 0.980997146 1.745287633 0.542671379 0.08481895 1 0.12201228 1

DB00177 1 0.510896216 1.75379047 0.753349628 0.1585137 1 0.12201228 1

DB00178 1 2.765242351 1.745287633 0.542671379 0.08481895 1 0.12201228 1

DB00179 1.5 1.851624387 1.745287633 0.542671379 0.325634379 1 0.346299761 1

DB00180 1 2.299864451 1.75379047 0.753349628 0.1585137 1 0.12201228 1

DB00181 1 1.672659347 1.745287633 0.542671379 0.08481895 1 0.12201228 1

DB00182 1.1764706 1.95026636 1.748450254 0.194078018 0.001603518 1 0.18583682 1

DB00183 1 0.91595549 1.75379047 0.753349628 0.1585137 1 0.12201228 1

DB00184 1.7692308 1.999886372 1.754873752 0.200318376 0.528568192 1 0.507619602 1

DB00185 1 2.068551425 1.745287633 0.542671379 0.08481895 1 0.12201228 1

DB00186 2 1.752196873 1.759334798 0.191532594 0.895536882 1 0.645919359 1

DB00187 2 1.617127463 1.75379047 0.753349628 0.628097886 1 0.645919359 1

DB00188 1 1.25893937 1.745287633 0.542671379 0.08481895 1 0.12201228 1

DB00189 2 1.980511033 1.759334798 0.191532594 0.895536882 1 0.645919359 1

DB00190 1 1.336222166 1.75379047 0.753349628 0.1585137 1 0.12201228 1

DB00191 1 2.166247225 1.770629523 0.307115021 0.006049327 1 0.12201228 1

DB00193 1.3181818 1.759769078 1.757638217 0.154827625 0.002267306 1 0.249772801 1

DB00195 2 1.18573848 1.745287633 0.542671379 0.680596551 1 0.645919359 1

DB00197 1.25 2.308300105 1.761473636 0.259363203 0.024302735 1 0.217648936 1

DB00198 1.5 1.588391674 1.745287633 0.542671379 0.325634379 1 0.346299761 1

DB00199 1 0.885751058 1.745287633 0.542671379 0.08481895 1 0.12201228 1

DB00200 1.6666667 1.513907712 1.770629523 0.307115021 0.367487831 1 0.444812957 1

DB00201 0.8500944 1.735260916 1.760507724 0.1309174 1.77E-12 9.69E-09 0.081396722 1

DB00202 1.25 1.517460878 1.753692879 0.367879736 0.085471804 1 0.217648936 1

DB00203 1 1.373506018 1.757244872 0.464542708 0.051541591 1 0.12201228 1

DB00204 1.6666667 1.537926241 1.757244872 0.464542708 0.422702925 1 0.444812957 1

DB00205 1.5 1.367693249 1.745287633 0.542671379 0.325634379 1 0.346299761 1

DB00206 2 1.063135457 1.75379047 0.753349628 0.628097886 1 0.645919359 1

DB00207 1 1.282825533 1.75379047 0.753349628 0.1585137 1 0.12201228 1

DB00208 1 0.425898095 1.75379047 0.753349628 0.1585137 1 0.12201228 1

DB00209 1 2.961588491 1.745287633 0.542671379 0.08481895 1 0.12201228 1

DB00210 1.5555556 1.983915021 1.753207263 0.264393406 0.227360828 1 0.378351838 1

DB00211 1 0.265981526 1.757244872 0.464542708 0.051541591 1 0.12201228 1

DB00212 2 1.510876386 1.75379047 0.753349628 0.628097886 1 0.645919359 1

DB00213 2 0.960832392 1.745287633 0.542671379 0.680596551 1 0.645919359 1

DB00214 2 2.48404198 1.745287633 0.542671379 0.680596551 1 0.645919359 1

DB00215 1 2.008100433 1.745287633 0.542671379 0.08481895 1 0.12201228 1

DB00216 1 1.552499713 1.753692879 0.367879736 0.020243381 1 0.12201228 1

DB00217 1.4285714 2.15622465 1.736866356 0.292454388 0.145903866 1 0.306680831 1

DB00218 2 1.051019244 1.75379047 0.753349628 0.628097886 1 0.645919359 1

DB00219 1 1.871818085 1.75379047 0.753349628 0.1585137 1 0.12201228 1

DB00221 2 1.656949215 1.745287633 0.542671379 0.680596551 1 0.645919359 1

DB00222 1.3333333 2.323831664 1.757244872 0.464542708 0.180743525 1 0.257238652 1

DB00223 1 2.1384493 1.75379047 0.753349628 0.1585137 1 0.12201228 1

DB00226 1 1.015603147 1.75379047 0.753349628 0.1585137 1 0.12201228 1

DB00227 1.3333333 1.257118469 1.757244872 0.464542708 0.180743525 1 0.257238652 1

DB00228 1.875 1.660048205 1.757186269 0.077658999 0.935374833 1 0.572174329 1

DB00230 2 2.070269726 1.75379047 0.753349628 0.628097886 1 0.645919359 1

DB00231 2 1.527615539 1.759334798 0.191532594 0.895536882 1 0.645919359 1

DB00232 2 1.436527908 1.753692879 0.367879736 0.748421796 1 0.645919359 1

DB00233 1.25 2.020056228 1.753692879 0.367879736 0.085471804 1 0.217648936 1

DB00234 1 1.620541491 1.75379047 0.753349628 0.1585137 1 0.12201228 1

DB00235 1 1.766103789 1.75379047 0.753349628 0.1585137 1 0.12201228 1

DB00237 1.9166667 1.648994187 1.762980177 0.123919082 0.892552385 1 0.597178491 1

DB00239 1 1.459068652 1.75379047 0.753349628 0.1585137 1 0.12201228 1

DB00240 1 1.676437172 1.75379047 0.753349628 0.1585137 1 0.12201228 1

DB00241 1.95 1.744740823 1.758534835 0.16776606 0.873119713 1 0.616904557 1

DB00242 1.7777778 1.956403789 1.753207263 0.264393406 0.537021102 1 0.512866521 1

DB00243 1.8148148 1.639260561 1.755736801 0.142706481 0.660556574 1 0.53556582 1

DB00244 1.2857143 1.751104838 1.736866356 0.292454388 0.061458983 1 0.234170029 1

DB00245 1 1.794370577 1.757979426 0.337271213 0.012307606 1 0.12201228 1

DB00246 1.2333333 2.241583665 1.760507724 0.1309174 2.83E-05 0.153471264 0.210175259 1

DB00247 1 2.252772885 1.761473636 0.259363203 0.001662717 1 0.12201228 1

DB00248 1.2 1.69960705 1.758534835 0.16776606 0.000435404 1 0.195688273 1

DB00252 1.8709677 1.515591473 1.748076871 0.135381851 0.817990846 1 0.569737381 1

DB00253 1 1.626800711 1.75379047 0.753349628 0.1585137 1 0.12201228 1

DB00255 1.1111111 1.447703698 1.753207263 0.264393406 0.007579386 1 0.160123241 1

DB00257 1 1.567169759 1.745287633 0.542671379 0.08481895 1 0.12201228 1

DB00261 1 1.831783837 1.75379047 0.753349628 0.1585137 1 0.12201228 1

DB00262 2 2.0872248 1.75379047 0.753349628 0.628097886 1 0.645919359 1

DB00264 2 1.690368234 1.745287633 0.542671379 0.680596551 1 0.645919359 1

DB00266 1.3333333 1.475153128 1.757244872 0.464542708 0.180743525 1 0.257238652 1

DB00268 1 1.885680707 1.753207263 0.264393406 0.002194136 1 0.12201228 1

DB00269 1 2.092989947 1.75379047 0.753349628 0.1585137 1 0.12201228 1

DB00270 1.7142857 1.71261244 1.736866356 0.292454388 0.4692279 1 0.473897096 1

DB00272 2 2.000385599 1.75379047 0.753349628 0.628097886 1 0.645919359 1

DB00273 1.8846154 1.753619643 1.752565715 0.14361026 0.82108304 1 0.577974197 1

DB00275 1 1.514150383 1.75379047 0.753349628 0.1585137 1 0.12201228 1

DB00276 1.25 2.177209772 1.753692879 0.367879736 0.085471804 1 0.217648936 1

DB00277 1.3333333 1.573397304 1.763093512 0.211303302 0.020983124 1 0.257238652 1

DB00278 1 0.572875634 1.75379047 0.753349628 0.1585137 1 0.12201228 1

DB00279 1 1.553485813 1.757244872 0.464542708 0.051541591 1 0.12201228 1

DB00280 1.1428571 1.702168982 1.736866356 0.292454388 0.021121533 1 0.172307877 1

DB00281 1.5 1.619404751 1.770629523 0.307115021 0.189104995 1 0.346299761 1

DB00282 1.5 1.824216271 1.753692879 0.367879736 0.245220308 1 0.346299761 1

DB00283 1 2.727883065 1.75379047 0.753349628 0.1585137 1 0.12201228 1

DB00284 1.25 1.711113005 1.753692879 0.367879736 0.085471804 1 0.217648936 1

DB00285 1 2.144589327 1.757244872 0.464542708 0.051541591 1 0.12201228 1

DB00286 1 1.638962994 1.745287633 0.542671379 0.08481895 1 0.12201228 1

DB00287 1 2.998089625 1.75379047 0.753349628 0.1585137 1 0.12201228 1

DB00288 1 1.267357564 1.745287633 0.542671379 0.08481895 1 0.12201228 1

DB00289 1.6363636 1.732827333 1.759747174 0.221112233 0.28841764 1 0.426448428 1

DB00290 2 0.838514562 1.745287633 0.542671379 0.680596551 1 0.645919359 1

DB00292 1.9411765 1.68439882 1.748450254 0.194078018 0.839653527 1 0.611710091 1

DB00293 1.5 2.561202903 1.745287633 0.542671379 0.325634379 1 0.346299761 1

DB00294 1 2.512123895 1.75379047 0.753349628 0.1585137 1 0.12201228 1

DB00295 1 1.893637156 1.753692879 0.367879736 0.020243381 1 0.12201228 1

DB00296 2 1.584758687 1.75379047 0.753349628 0.628097886 1 0.645919359 1

DB00297 1.5 2.019314288 1.745287633 0.542671379 0.325634379 1 0.346299761 1

DB00298 1 1.830610928 1.757244872 0.464542708 0.051541591 1 0.12201228 1

DB00302 1 0.746231895 1.75379047 0.753349628 0.1585137 1 0.12201228 1

DB00304 1 2.215639716 1.745287633 0.542671379 0.08481895 1 0.12201228 1

DB00306 1.95 1.862251551 1.758534835 0.16776606 0.873119713 1 0.616904557 1

DB00307 1.6666667 2.302384079 1.757244872 0.464542708 0.422702925 1 0.444812957 1

DB00308 1.7 1.963172227 1.759693587 0.247672276 0.40477041 1 0.46515008 1

DB00309 1 2.193081016 1.75379047 0.753349628 0.1585137 1 0.12201228 1

DB00310 2 0.702820888 1.745287633 0.542671379 0.680596551 1 0.645919359 1

DB00311 2 1.735127635 1.757979426 0.337271213 0.763493223 1 0.645919359 1

DB00312 1.8928571 1.645390493 1.757145597 0.141561363 0.831139043 1 0.582932095 1

DB00313 1.7368421 1.908108235 1.755240506 0.172383815 0.457501864 1 0.487732195 1

DB00315 1 1.957277611 1.761473636 0.259363203 0.001662717 1 0.12201228 1

DB00316 1.3333333 1.296010367 1.757244872 0.464542708 0.180743525 1 0.257238652 1

DB00317 1 2.652906329 1.75379047 0.753349628 0.1585137 1 0.12201228 1

DB00318 1 1.247585663 1.757244872 0.464542708 0.051541591 1 0.12201228 1

DB00320 1 1.41466842 1.753692879 0.367879736 0.020243381 1 0.12201228 1

DB00321 1.2727273 1.94083623 1.751829593 0.13569543 0.000207217 1 0.228083394 1

DB00322 1 3.189473805 1.75379047 0.753349628 0.1585137 1 0.12201228 1

DB00323 1 2.931656611 1.75379047 0.753349628 0.1585137 1 0.12201228 1

DB00324 1 1.658969475 1.75379047 0.753349628 0.1585137 1 0.12201228 1

DB00325 1 1.4717021 1.75379047 0.753349628 0.1585137 1 0.12201228 1

DB00327 1 1.643298897 1.757244872 0.464542708 0.051541591 1 0.12201228 1

DB00328 1.3333333 2.101799889 1.753207263 0.264393406 0.0561358 1 0.257238652 1

DB00331 1.6666667 1.363877316 1.757244872 0.464542708 0.422702925 1 0.444812957 1

DB00332 1 1.673853682 1.757244872 0.464542708 0.051541591 1 0.12201228 1

DB00333 1.7142857 1.809631922 1.773663076 0.187305714 0.375618834 1 0.473897096 1

DB00334 1.3870968 1.858786774 1.748076871 0.135381851 0.003833597 1 0.284636289 1

DB00335 2 1.470820029 1.745287633 0.542671379 0.680596551 1 0.645919359 1

DB00337 1.5 1.739087968 1.745287633 0.542671379 0.325634379 1 0.346299761 1

DB00338 1.5 1.674293749 1.745287633 0.542671379 0.325634379 1 0.346299761 1

DB00340 1 1.884217816 1.757979426 0.337271213 0.012307606 1 0.12201228 1

DB00341 1 1.503535137 1.75379047 0.753349628 0.1585137 1 0.12201228 1

DB00342 1 1.729069967 1.736866356 0.292454388 0.005874511 1 0.12201228 1

DB00343 1.5 1.671531646 1.745287633 0.542671379 0.325634379 1 0.346299761 1

DB00344 1 1.878805057 1.745287633 0.542671379 0.08481895 1 0.12201228 1

DB00346 1 1.591580463 1.753692879 0.367879736 0.020243381 1 0.12201228 1

DB00347 2 1.342467151 1.75379047 0.753349628 0.628097886 1 0.645919359 1

DB00349 2 1.746790938 1.759334798 0.191532594 0.895536882 1 0.645919359 1

DB00350 2 1.686204424 1.757244872 0.464542708 0.699362524 1 0.645919359 1

DB00351 1 1.2280741 1.745287633 0.542671379 0.08481895 1 0.12201228 1

DB00353 1.5 2.256591684 1.745287633 0.542671379 0.325634379 1 0.346299761 1

DB00354 1 1.210893869 1.745287633 0.542671379 0.08481895 1 0.12201228 1

DB00356 1 3.321355168 1.75379047 0.753349628 0.1585137 1 0.12201228 1

DB00357 1.5 2.437836649 1.745287633 0.542671379 0.325634379 1 0.346299761 1

DB00358 1 1.398682019 1.75379047 0.753349628 0.1585137 1 0.12201228 1

DB00360 1.25 1.335026056 1.753692879 0.367879736 0.085471804 1 0.217648936 1

DB00361 1 3.04691807 1.75379047 0.753349628 0.1585137 1 0.12201228 1

DB00363 1.1111111 1.573672081 1.755736801 0.142706481 3.13E-06 0.017039377 0.160123241 1

DB00364 1.25 1.814650708 1.753692879 0.367879736 0.085471804 1 0.217648936 1

DB00366 1 2.186915955 1.736866356 0.292454388 0.005874511 1 0.12201228 1

DB00367 1.1666667 1.442522295 1.770629523 0.307115021 0.024616471 1 0.181824474 1

DB00368 1.3636364 1.699825925 1.759747174 0.221112233 0.036610823 1 0.272511088 1

DB00370 1 1.795546033 1.761473636 0.259363203 0.001662717 1 0.12201228 1

DB00371 2 1.63860344 1.759334798 0.191532594 0.895536882 1 0.645919359 1

DB00372 1 1.159518397 1.75379047 0.753349628 0.1585137 1 0.12201228 1

DB00373 2 1.994671147 1.745287633 0.542671379 0.680596551 1 0.645919359 1

DB00374 1.3333333 1.591631106 1.757244872 0.464542708 0.180743525 1 0.257238652 1

DB00376 1 1.680009788 1.757979426 0.337271213 0.012307606 1 0.12201228 1

DB00377 1 2.275229785 1.75379047 0.753349628 0.1585137 1 0.12201228 1

DB00378 1 2.17276286 1.75379047 0.753349628 0.1585137 1 0.12201228 1

DB00379 1 1.792164622 1.75379047 0.753349628 0.1585137 1 0.12201228 1

DB00380 2 1.815155173 1.745287633 0.542671379 0.680596551 1 0.645919359 1

DB00381 1.7142857 1.762319746 1.736866356 0.292454388 0.4692279 1 0.473897096 1

DB00382 2 2.607096942 1.757244872 0.464542708 0.699362524 1 0.645919359 1

DB00383 1 1.725153899 1.757244872 0.464542708 0.051541591 1 0.12201228 1

DB00384 2 1.570840107 1.753692879 0.367879736 0.748421796 1 0.645919359 1

DB00385 2 -0.445037068 1.75379047 0.753349628 0.628097886 1 0.645919359 1

DB00387 1 2.214574845 1.753692879 0.367879736 0.020243381 1 0.12201228 1

DB00388 1 1.701067035 1.757244872 0.464542708 0.051541591 1 0.12201228 1

DB00389 1 1.51609606 1.75379047 0.753349628 0.1585137 1 0.12201228 1

DB00390 2 -0.449325176 1.75379047 0.753349628 0.628097886 1 0.645919359 1

DB00391 1.5 1.382141352 1.753692879 0.367879736 0.245220308 1 0.346299761 1

DB00392 1.3333333 2.115899067 1.757244872 0.464542708 0.180743525 1 0.257238652 1

DB00393 1.7 1.577289136 1.759693587 0.247672276 0.40477041 1 0.46515008 1

DB00394 1 2.178126558 1.75379047 0.753349628 0.1585137 1 0.12201228 1

DB00395 2 1.959105842 1.757979426 0.337271213 0.763493223 1 0.645919359 1

DB00396 1.2 1.996389198 1.759693587 0.247672276 0.01191636 1 0.195688273 1

DB00397 1.5 1.593106676 1.770629523 0.307115021 0.189104995 1 0.346299761 1

DB00398 1 1.671132413 1.753207263 0.264393406 0.002194136 1 0.12201228 1

DB00399 2 1.555425584 1.745287633 0.542671379 0.680596551 1 0.645919359 1

DB00400 2 0.587817812 1.75379047 0.753349628 0.628097886 1 0.645919359 1

DB00401 1.6 1.680873686 1.757979426 0.337271213 0.319747599 1 0.404623142 1

DB00402 2 1.755287792 1.759334798 0.191532594 0.895536882 1 0.645919359 1

DB00403 1 1.527703215 1.75379047 0.753349628 0.1585137 1 0.12201228 1

DB00404 2 2.002688418 1.759334798 0.191532594 0.895536882 1 0.645919359 1

DB00405 1 1.594572478 1.75379047 0.753349628 0.1585137 1 0.12201228 1

DB00407 1.5 2.101761557 1.745287633 0.542671379 0.325634379 1 0.346299761 1

DB00408 1.15625 1.91979934 1.752088368 0.130643051 2.55E-06 0.013855001 0.177621196 1

DB00409 1.2 1.943850774 1.757979426 0.337271213 0.049023756 1 0.195688273 1

DB00411 1.3333333 1.601766321 1.770629523 0.307115021 0.07724 1 0.257238652 1

DB00412 1.4285714 1.14581592 1.736866356 0.292454388 0.145903866 1 0.306680831 1

DB00413 1 1.725853831 1.757979426 0.337271213 0.012307606 1 0.12201228 1

DB00414 2 2.459387608 1.75379047 0.753349628 0.628097886 1 0.645919359 1

DB00415 1.3333333 1.673198175 1.757244872 0.464542708 0.180743525 1 0.257238652 1

DB00416 2 1.793371081 1.75379047 0.753349628 0.628097886 1 0.645919359 1

DB00417 2 2.207138284 1.75379047 0.753349628 0.628097886 1 0.645919359 1

DB00418 1.8823529 1.599949444 1.748450254 0.194078018 0.754884782 1 0.576610962 1

DB00419 1 2.604781222 1.75379047 0.753349628 0.1585137 1 0.12201228 1

DB00420 1 1.708906397 1.770629523 0.307115021 0.006049327 1 0.12201228 1

DB00421 1.75 1.682947711 1.762980177 0.123919082 0.458288204 1 0.495810565 1

DB00422 1 1.376495227 1.757244872 0.464542708 0.051541591 1 0.12201228 1

DB00423 2 2.098208861 1.75379047 0.753349628 0.628097886 1 0.645919359 1

DB00424 1 2.025071917 1.753692879 0.367879736 0.020243381 1 0.12201228 1

DB00425 2 2.064828136 1.753692879 0.367879736 0.748421796 1 0.645919359 1

DB00427 1 1.342636338 1.75379047 0.753349628 0.1585137 1 0.12201228 1

DB00428 1.5 2.264859011 1.745287633 0.542671379 0.325634379 1 0.346299761 1

DB00429 1 2.108157137 1.75379047 0.753349628 0.1585137 1 0.12201228 1

DB00431 1.7 1.85634232 1.759693587 0.247672276 0.40477041 1 0.46515008 1

DB00432 1 0.618076532 1.75379047 0.753349628 0.1585137 1 0.12201228 1

DB00433 1 1.643160954 1.761473636 0.259363203 0.001662717 1 0.12201228 1

DB00434 1 1.588675718 1.736866356 0.292454388 0.005874511 1 0.12201228 1

DB00435 -2.161505 2.921438424 1.745287633 0.542671379 3.03E-13 1.66E-09 8.12E-10 4.44E-06

DB00436 1.8 2.357883664 1.757979426 0.337271213 0.549575873 1 0.52649583 1

DB00437 2 2.621892508 1.75379047 0.753349628 0.628097886 1 0.645919359 1

DB00439 2 0.873453189 1.75379047 0.753349628 0.628097886 1 0.645919359 1

DB00441 1.3333333 1.166363568 1.757244872 0.464542708 0.180743525 1 0.257238652 1

DB00443 1 2.688157113 1.75379047 0.753349628 0.1585137 1 0.12201228 1

DB00444 2 1.828175125 1.75379047 0.753349628 0.628097886 1 0.645919359 1

DB00445 2 1.132860737 1.75379047 0.753349628 0.628097886 1 0.645919359 1

DB00448 2 0.743203719 1.745287633 0.542671379 0.680596551 1 0.645919359 1

DB00449 1.6 2.234388874 1.757979426 0.337271213 0.319747599 1 0.404623142 1

DB00450 1 1.115240707 1.745287633 0.542671379 0.08481895 1 0.12201228 1

DB00451 1 1.620305765 1.753692879 0.367879736 0.020243381 1 0.12201228 1

DB00452 1 0.559249808 1.75379047 0.753349628 0.1585137 1 0.12201228 1

DB00454 1.3125 1.600865254 1.759334798 0.191532594 0.009825545 1 0.247003068 1

DB00455 1 1.51994921 1.745287633 0.542671379 0.08481895 1 0.12201228 1

DB00457 1 1.922509021 1.770629523 0.307115021 0.006049327 1 0.12201228 1

DB00458 1.2 1.575763966 1.76622404 0.151326267 9.14E-05 0.494790664 0.195688273 1

DB00459 1.6666667 1.758361695 1.770629523 0.307115021 0.367487831 1 0.444812957 1

DB00461 1.5 2.106347722 1.745287633 0.542671379 0.325634379 1 0.346299761 1

DB00462 1 1.025115838 1.757244872 0.464542708 0.051541591 1 0.12201228 1

DB00463 1.95 1.819351946 1.758534835 0.16776606 0.873119713 1 0.616904557 1

DB00464 1 1.085964899 1.745287633 0.542671379 0.08481895 1 0.12201228 1

DB00465 1.5 2.116174968 1.745287633 0.542671379 0.325634379 1 0.346299761 1

DB00466 2 1.81410192 1.757979426 0.337271213 0.763493223 1 0.645919359 1

DB00467 2 3.189766549 1.75379047 0.753349628 0.628097886 1 0.645919359 1

DB00468 1 1.421417676 1.75379047 0.753349628 0.1585137 1 0.12201228 1

DB00469 1.5 1.419279779 1.745287633 0.542671379 0.325634379 1 0.346299761 1

DB00470 1 1.053877976 1.745287633 0.542671379 0.08481895 1 0.12201228 1

DB00471 1.5 2.167519323 1.745287633 0.542671379 0.325634379 1 0.346299761 1

DB00472 1.2857143 1.776640772 1.736866356 0.292454388 0.061458983 1 0.234170029 1

DB00473 2 1.736612078 1.75379047 0.753349628 0.628097886 1 0.645919359 1

DB00474 2 2.183211017 1.75379047 0.753349628 0.628097886 1 0.645919359 1

DB00475 2 1.759451207 1.759334798 0.191532594 0.895536882 1 0.645919359 1

DB00476 1 2.023708944 1.757244872 0.464542708 0.051541591 1 0.12201228 1

DB00477 1.1153846 1.630497289 1.752565715 0.14361026 4.56E-06 0.024808071 0.161729849 1

DB00480 1 1.577404434 1.753692879 0.367879736 0.020243381 1 0.12201228 1

DB00481 1.5 1.607779467 1.753692879 0.367879736 0.245220308 1 0.346299761 1

DB00482 1.5 1.134052119 1.753692879 0.367879736 0.245220308 1 0.346299761 1

DB00483 1.4 1.545182721 1.757979426 0.337271213 0.14425424 1 0.291413148 1

DB00484 1 1.395057134 1.757244872 0.464542708 0.051541591 1 0.12201228 1

DB00486 1 1.787418825 1.745287633 0.542671379 0.08481895 1 0.12201228 1

DB00487 2 2.581011786 1.75379047 0.753349628 0.628097886 1 0.645919359 1

DB00489 1.6666667 1.266155995 1.757244872 0.464542708 0.422702925 1 0.444812957 1

DB00490 1 1.361123249 1.736866356 0.292454388 0.005874511 1 0.12201228 1

DB00491 1.5 1.495059555 1.753692879 0.367879736 0.245220308 1 0.346299761 1

DB00492 1 1.283941549 1.75379047 0.753349628 0.1585137 1 0.12201228 1

DB00493 1.5714286 1.814306396 1.736866356 0.292454388 0.285803159 1 0.387676968 1

DB00494 1 1.276616886 1.75379047 0.753349628 0.1585137 1 0.12201228 1

DB00495 1 2.550766294 1.75379047 0.753349628 0.1585137 1 0.12201228 1

DB00496 1 2.084928001 1.757979426 0.337271213 0.012307606 1 0.12201228 1

DB00497 1 1.727762814 1.757244872 0.464542708 0.051541591 1 0.12201228 1

DB00498 2 3.693536585 1.75379047 0.753349628 0.628097886 1 0.645919359 1

DB00499 1 1.372029776 1.757244872 0.464542708 0.051541591 1 0.12201228 1

DB00500 1.5 1.533467713 1.745287633 0.542671379 0.325634379 1 0.346299761 1

DB00501 2 1.929514284 1.75379047 0.753349628 0.628097886 1 0.645919359 1

DB00502 1.2352941 1.451576588 1.748450254 0.194078018 0.004095768 1 0.211046617 1

DB00503 1 1.935254685 1.75379047 0.753349628 0.1585137 1 0.12201228 1

DB00504 1 1.967129361 1.75379047 0.753349628 0.1585137 1 0.12201228 1

DB00505 1 1.277615491 1.757244872 0.464542708 0.051541591 1 0.12201228 1

DB00508 1.2 1.780672545 1.757979426 0.337271213 0.049023756 1 0.195688273 1

DB00509 1 1.302010036 1.757244872 0.464542708 0.051541591 1 0.12201228 1

DB00511 2 1.852552961 1.75379047 0.753349628 0.628097886 1 0.645919359 1

DB00513 1.6666667 1.86582932 1.757244872 0.464542708 0.422702925 1 0.444812957 1

DB00514 1.3333333 1.873887179 1.763665626 0.163272037 0.004198483 1 0.257238652 1

DB00515 1.25 2.369663714 1.753692879 0.367879736 0.085471804 1 0.217648936 1

DB00517 1 0.810185105 1.757244872 0.464542708 0.051541591 1 0.12201228 1

DB00518 2 0.924266854 1.745287633 0.542671379 0.680596551 1 0.645919359 1

DB00519 1 1.476628467 1.75379047 0.753349628 0.1585137 1 0.12201228 1

DB00521 2 2.618686746 1.745287633 0.542671379 0.680596551 1 0.645919359 1

DB00522 1 1.663312494 1.75379047 0.753349628 0.1585137 1 0.12201228 1

DB00523 1.75 2.33904932 1.761473636 0.259363203 0.482357459 1 0.495810565 1

DB00524 2 3.514748268 1.75379047 0.753349628 0.628097886 1 0.645919359 1

DB00527 1.5 2.46197534 1.745287633 0.542671379 0.325634379 1 0.346299761 1

DB00528 2 2.602574402 1.75379047 0.753349628 0.628097886 1 0.645919359 1

DB00530 1 1.380982627 1.745287633 0.542671379 0.08481895 1 0.12201228 1

DB00531 1 1.128192399 1.75379047 0.753349628 0.1585137 1 0.12201228 1

DB00532 1 1.386609348 1.75379047 0.753349628 0.1585137 1 0.12201228 1

DB00533 1 2.193473243 1.745287633 0.542671379 0.08481895 1 0.12201228 1

DB00534 2 2.263721448 1.745287633 0.542671379 0.680596551 1 0.645919359 1

DB00535 1 0.50955258 1.75379047 0.753349628 0.1585137 1 0.12201228 1

DB00536 2 1.159487026 1.757244872 0.464542708 0.699362524 1 0.645919359 1

DB00537 1.5 2.391663408 1.745287633 0.542671379 0.325634379 1 0.346299761 1

DB00539 1 2.357428262 1.745287633 0.542671379 0.08481895 1 0.12201228 1

DB00540 1.1304348 1.491040826 1.750829326 0.165162414 8.62E-05 0.467121488 0.16747126 1

DB00541 1.5 1.757054745 1.745287633 0.542671379 0.325634379 1 0.346299761 1

DB00542 1 1.969357036 1.75379047 0.753349628 0.1585137 1 0.12201228 1

DB00543 1.4090909 1.829960651 1.758339856 0.115069137 0.001202189 1 0.296232505 1

DB00544 1 0.217451151 1.75379047 0.753349628 0.1585137 1 0.12201228 1

DB00545 1.6666667 1.519550977 1.757244872 0.464542708 0.422702925 1 0.444812957 1

DB00546 2 1.958697975 1.759334798 0.191532594 0.895536882 1 0.645919359 1

DB00547 1 2.413971334 1.75379047 0.753349628 0.1585137 1 0.12201228 1

DB00548 1.3333333 1.714456165 1.757244872 0.464542708 0.180743525 1 0.257238652 1

DB00549 1 1.463531484 1.75379047 0.753349628 0.1585137 1 0.12201228 1

DB00550 1 1.792750477 1.75379047 0.753349628 0.1585137 1 0.12201228 1

DB00551 1 1.685349364 1.75379047 0.753349628 0.1585137 1 0.12201228 1

DB00552 1 -0.138430586 1.75379047 0.753349628 0.1585137 1 0.12201228 1

DB00554 1.5 2.870496142 1.745287633 0.542671379 0.325634379 1 0.346299761 1

DB00555 1.7368421 1.842860998 1.751411435 0.118886128 0.451232258 1 0.487732195 1

DB00557 1 1.860650401 1.745287633 0.542671379 0.08481895 1 0.12201228 1

DB00558 2 3.363106138 1.75379047 0.753349628 0.628097886 1 0.645919359 1

DB00559 1 1.621144021 1.745287633 0.542671379 0.08481895 1 0.12201228 1

DB00561 2 1.568592826 1.745287633 0.542671379 0.680596551 1 0.645919359 1

DB00562 1.9166667 1.894464173 1.763093512 0.211303302 0.766322785 1 0.597178491 1

DB00563 1.3333333 1.941788462 1.757244872 0.464542708 0.180743525 1 0.257238652 1

DB00564 1.8888889 1.749815173 1.753207263 0.264393406 0.696087596 1 0.580546569 1

DB00565 2 1.274170781 1.75379047 0.753349628 0.628097886 1 0.645919359 1

DB00568 1.4375 1.950946778 1.759334798 0.191532594 0.04644817 1 0.311523401 1

DB00569 2 2.062606183 1.745287633 0.542671379 0.680596551 1 0.645919359 1

DB00570 1.5 1.687139869 1.770629523 0.307115021 0.189104995 1 0.346299761 1

DB00571 1.4 1.636498631 1.757979426 0.337271213 0.14425424 1 0.291413148 1

DB00572 1.375 1.791050394 1.761473636 0.259363203 0.068100732 1 0.278352103 1

DB00573 1.25 1.581344396 1.753692879 0.367879736 0.085471804 1 0.217648936 1

DB00574 1 2.119437891 1.753692879 0.367879736 0.020243381 1 0.12201228 1

DB00575 1 1.555875265 1.770629523 0.307115021 0.006049327 1 0.12201228 1

DB00579 1 1.127219261 1.757244872 0.464542708 0.051541591 1 0.12201228 1

DB00580 1.6666667 2.093391894 1.757244872 0.464542708 0.422702925 1 0.444812957 1

DB00583 1.6363636 1.745852811 1.759747174 0.221112233 0.28841764 1 0.426448428 1

DB00584 1 3.013482539 1.75379047 0.753349628 0.1585137 1 0.12201228 1

DB00585 2 2.486158935 1.75379047 0.753349628 0.628097886 1 0.645919359 1

DB00586 1.5 1.086405884 1.745287633 0.542671379 0.325634379 1 0.346299761 1

DB00587 1 1.912621819 1.75379047 0.753349628 0.1585137 1 0.12201228 1

DB00588 1.25 1.535753281 1.753692879 0.367879736 0.085471804 1 0.217648936 1

DB00589 1.1333333 1.661967834 1.760312929 0.204781867 0.001100438 1 0.168591892 1

DB00590 1.3333333 1.680193313 1.770629523 0.307115021 0.07724 1 0.257238652 1

DB00591 1.1666667 2.118822003 1.770629523 0.307115021 0.024616471 1 0.181824474 1

DB00592 2 2.322660595 1.75379047 0.753349628 0.628097886 1 0.645919359 1

DB00593 2 0.899929677 1.75379047 0.753349628 0.628097886 1 0.645919359 1

DB00594 1.7777778 1.699548412 1.753207263 0.264393406 0.537021102 1 0.512866521 1

DB00596 1 1.228674127 1.75379047 0.753349628 0.1585137 1 0.12201228 1

DB00598 1.4 2.009968983 1.757979426 0.337271213 0.14425424 1 0.291413148 1

DB00599 1.75 1.791426151 1.763093512 0.211303302 0.475295159 1 0.495810565 1

DB00600 1 1.041537905 1.75379047 0.753349628 0.1585137 1 0.12201228 1

DB00602 2 0.705577795 1.745287633 0.542671379 0.680596551 1 0.645919359 1

DB00603 1.8421053 1.739486599 1.755240506 0.172383815 0.692835377 1 0.552222038 1

DB00604 1.25 1.90369073 1.753692879 0.367879736 0.085471804 1 0.217648936 1

DB00605 1.4285714 1.384837629 1.736866356 0.292454388 0.145903866 1 0.306680831 1

DB00606 1.8333333 1.860411335 1.763093512 0.211303302 0.630210974 1 0.546876824 1

DB00608 1 1.623481783 1.770629523 0.307115021 0.006049327 1 0.12201228 1

DB00610 1 1.021958851 1.75379047 0.753349628 0.1585137 1 0.12201228 1

DB00611 1 2.10249317 1.757244872 0.464542708 0.051541591 1 0.12201228 1

DB00612 2 2.356625792 1.745287633 0.542671379 0.680596551 1 0.645919359 1

DB00613 2 1.006451949 1.75379047 0.753349628 0.628097886 1 0.645919359 1

DB00615 1 2.280617775 1.745287633 0.542671379 0.08481895 1 0.12201228 1

DB00616 1 1.701218925 1.745287633 0.542671379 0.08481895 1 0.12201228 1

DB00617 2 2.564708347 1.75379047 0.753349628 0.628097886 1 0.645919359 1

DB00619 1.125 2.030430535 1.761473636 0.259363203 0.007064127 1 0.165382999 1

DB00620 1 2.879097247 1.75379047 0.753349628 0.1585137 1 0.12201228 1

DB00621 1 1.911240448 1.75379047 0.753349628 0.1585137 1 0.12201228 1

DB00622 1.2 1.814900774 1.760312929 0.204781867 0.003108179 1 0.195688273 1

DB00623 1.1666667 1.689412323 1.770629523 0.307115021 0.024616471 1 0.181824474 1

DB00624 1.3333333 1.874381003 1.757244872 0.464542708 0.180743525 1 0.257238652 1

DB00626 1.5 2.031158209 1.745287633 0.542671379 0.325634379 1 0.346299761 1

DB00627 1.25 2.223205755 1.753692879 0.367879736 0.085471804 1 0.217648936 1

DB00628 2 1.79621322 1.759334798 0.191532594 0.895536882 1 0.645919359 1

DB00629 1 0.981354096 1.745287633 0.542671379 0.08481895 1 0.12201228 1

DB00630 2 2.094071262 1.753692879 0.367879736 0.748421796 1 0.645919359 1

DB00631 1.5 2.900619119 1.745287633 0.542671379 0.325634379 1 0.346299761 1

DB00633 1 2.191463675 1.75379047 0.753349628 0.1585137 1 0.12201228 1

DB00635 1 2.60044013 1.75379047 0.753349628 0.1585137 1 0.12201228 1

DB00636 1 2.091196997 1.75379047 0.753349628 0.1585137 1 0.12201228 1

DB00637 1.5 2.117357228 1.753692879 0.367879736 0.245220308 1 0.346299761 1

DB00640 1 1.808321599 1.757244872 0.464542708 0.051541591 1 0.12201228 1

DB00641 1.3333333 2.487463334 1.757244872 0.464542708 0.180743525 1 0.257238652 1

DB00642 1.25 1.714597669 1.753692879 0.367879736 0.085471804 1 0.217648936 1

DB00643 2 2.44233594 1.745287633 0.542671379 0.680596551 1 0.645919359 1

DB00644 1 2.886738863 1.75379047 0.753349628 0.1585137 1 0.12201228 1

DB00645 2 4.113451239 1.75379047 0.753349628 0.628097886 1 0.645919359 1

DB00647 1.25 1.808029382 1.753692879 0.367879736 0.085471804 1 0.217648936 1

DB00648 1.4 1.988398344 1.757979426 0.337271213 0.14425424 1 0.291413148 1

DB00651 1 2.225395472 1.757979426 0.337271213 0.012307606 1 0.12201228 1

DB00652 1.3333333 1.176756264 1.757244872 0.464542708 0.180743525 1 0.257238652 1

DB00653 1.7777778 2.056345971 1.753207263 0.264393406 0.537021102 1 0.512866521 1

DB00654 1 1.198648487 1.75379047 0.753349628 0.1585137 1 0.12201228 1

DB00655 1 1.646375628 1.757979426 0.337271213 0.012307606 1 0.12201228 1

DB00656 1 1.39894377 1.736866356 0.292454388 0.005874511 1 0.12201228 1

DB00657 2 1.508226695 1.753692879 0.367879736 0.748421796 1 0.645919359 1

DB00659 1.9166667 1.711782649 1.751434425 0.152818387 0.860203247 1 0.597178491 1

DB00661 1.3636364 1.507977505 1.759747174 0.221112233 0.036610823 1 0.272511088 1

DB00663 1 1.790747459 1.75379047 0.753349628 0.1585137 1 0.12201228 1

DB00665 1 2.66361654 1.75379047 0.753349628 0.1585137 1 0.12201228 1

DB00666 1 1.465956996 1.75379047 0.753349628 0.1585137 1 0.12201228 1

DB00668 1.25 1.787268082 1.761473636 0.259363203 0.024302735 1 0.217648936 1

DB00669 1 1.301188428 1.753692879 0.367879736 0.020243381 1 0.12201228 1

DB00670 1 1.390575554 1.75379047 0.753349628 0.1585137 1 0.12201228 1

DB00672 1 1.898341354 1.75379047 0.753349628 0.1585137 1 0.12201228 1

DB00673 1 2.452985395 1.75379047 0.753349628 0.1585137 1 0.12201228 1

DB00674 2 0.972408624 1.757244872 0.464542708 0.699362524 1 0.645919359 1

DB00675 1.125 1.655357585 1.759334798 0.191532594 0.000463341 1 0.165382999 1

DB00677 1.6666667 1.586323138 1.757244872 0.464542708 0.422702925 1 0.444812957 1

DB00678 1 1.951255198 1.75379047 0.753349628 0.1585137 1 0.12201228 1

DB00679 1.1666667 2.153531447 1.770629523 0.307115021 0.024616471 1 0.181824474 1

DB00682 1.5 1.999476178 1.745287633 0.542671379 0.325634379 1 0.346299761 1

DB00683 2 1.801603113 1.759334798 0.191532594 0.895536882 1 0.645919359 1

DB00685 2 2.946369145 1.75379047 0.753349628 0.628097886 1 0.645919359 1

DB00686 1.5 1.358507555 1.745287633 0.542671379 0.325634379 1 0.346299761 1

DB00687 1.5 2.38298717 1.745287633 0.542671379 0.325634379 1 0.346299761 1

DB00688 1.6666667 2.078088711 1.757244872 0.464542708 0.422702925 1 0.444812957 1

DB00690 2 1.870765992 1.759334798 0.191532594 0.895536882 1 0.645919359 1

DB00691 1 1.567996602 1.75379047 0.753349628 0.1585137 1 0.12201228 1

DB00692 1 1.524021386 1.753692879 0.367879736 0.020243381 1 0.12201228 1

DB00694 2 0.615569327 1.745287633 0.542671379 0.680596551 1 0.645919359 1

DB00695 1.6666667 2.324906022 1.757244872 0.464542708 0.422702925 1 0.444812957 1

DB00696 1.1333333 1.741651864 1.760312929 0.204781867 0.001100438 1 0.168591892 1

DB00697 1.1428571 1.650693175 1.736866356 0.292454388 0.021121533 1 0.172307877 1

DB00699 1 1.100038326 1.75379047 0.753349628 0.1585137 1 0.12201228 1

DB00700 2 0.446419945 1.75379047 0.753349628 0.628097886 1 0.645919359 1

DB00703 2 2.14807545 1.757979426 0.337271213 0.763493223 1 0.645919359 1

DB00704 1 2.369528293 1.757244872 0.464542708 0.051541591 1 0.12201228 1

DB00706 1 1.362420431 1.757244872 0.464542708 0.051541591 1 0.12201228 1

DB00707 1 0.657617431 1.745287633 0.542671379 0.08481895 1 0.12201228 1

DB00708 1 2.720119638 1.757244872 0.464542708 0.051541591 1 0.12201228 1

DB00710 2 2.06548085 1.745287633 0.542671379 0.680596551 1 0.645919359 1

DB00711 2 2.249137972 1.745287633 0.542671379 0.680596551 1 0.645919359 1

DB00712 1.5 2.064708106 1.745287633 0.542671379 0.325634379 1 0.346299761 1

DB00713 1.5 1.746759246 1.745287633 0.542671379 0.325634379 1 0.346299761 1

DB00714 1.1428571 1.670688812 1.773663076 0.187305714 0.000378869 1 0.172307877 1

DB00715 1.2777778 1.793982261 1.762980177 0.123919082 4.51E-05 0.24464635 0.230439784 1

DB00716 1.2 1.561654116 1.757979426 0.337271213 0.049023756 1 0.195688273 1

DB00717 1 1.891719726 1.757244872 0.464542708 0.051541591 1 0.12201228 1

DB00719 1 3.513782988 1.75379047 0.753349628 0.1585137 1 0.12201228 1

DB00720 2 1.738023993 1.757244872 0.464542708 0.699362524 1 0.645919359 1

DB00721 1.5882353 1.526041706 1.748450254 0.194078018 0.204538701 1 0.397621638 1

DB00722 1.5 1.100687666 1.745287633 0.542671379 0.325634379 1 0.346299761 1

DB00723 1 1.841993847 1.757244872 0.464542708 0.051541591 1 0.12201228 1

DB00724 1 1.439977082 1.745287633 0.542671379 0.08481895 1 0.12201228 1

DB00725 1 2.482876735 1.757979426 0.337271213 0.012307606 1 0.12201228 1

DB00726 1.1666667 1.806690024 1.751434425 0.152818387 6.50E-05 0.352164361 0.181824474 1

DB00727 1 1.244128875 1.75379047 0.753349628 0.1585137 1 0.12201228 1

DB00728 1.3333333 1.675450039 1.757244872 0.464542708 0.180743525 1 0.257238652 1

DB00731 1 0.990890692 1.745287633 0.542671379 0.08481895 1 0.12201228 1

DB00732 2 0.615162291 1.75379047 0.753349628 0.628097886 1 0.645919359 1

DB00733 2 1.881607731 1.745287633 0.542671379 0.680596551 1 0.645919359 1

DB00734 1.0833333 1.760384314 1.763093512 0.211303302 0.00064772 1 0.149935463 1

DB00735 2 2.27886534 1.75379047 0.753349628 0.628097886 1 0.645919359 1

DB00736 2 2.985183488 1.745287633 0.542671379 0.680596551 1 0.645919359 1

DB00737 1 1.451356121 1.745287633 0.542671379 0.08481895 1 0.12201228 1

DB00738 2 0.878436959 1.75379047 0.753349628 0.628097886 1 0.645919359 1

DB00740 2 1.542053738 1.75379047 0.753349628 0.628097886 1 0.645919359 1

DB00741 1 1.184083477 1.745287633 0.542671379 0.08481895 1 0.12201228 1

DB00744 2 1.576568643 1.75379047 0.753349628 0.628097886 1 0.645919359 1

DB00745 1 1.607321912 1.745287633 0.542671379 0.08481895 1 0.12201228 1

DB00746 1 1.844594038 1.75379047 0.753349628 0.1585137 1 0.12201228 1

DB00747 1.25 1.59231193 1.761473636 0.259363203 0.024302735 1 0.217648936 1

DB00748 1 2.056018849 1.75379047 0.753349628 0.1585137 1 0.12201228 1

DB00749 1.6666667 1.115880038 1.757244872 0.464542708 0.422702925 1 0.444812957 1

DB00751 1.1666667 1.820998589 1.770629523 0.307115021 0.024616471 1 0.181824474 1

DB00752 1 1.700771663 1.745287633 0.542671379 0.08481895 1 0.12201228 1

DB00753 1.9583333 1.768199214 1.751434425 0.152818387 0.912113834 1 0.621791228 1

DB00754 1 1.836933468 1.75379047 0.753349628 0.1585137 1 0.12201228 1

DB00755 1.5882353 1.693026032 1.748450254 0.194078018 0.204538701 1 0.397621638 1

DB00756 1.3333333 1.589013966 1.757244872 0.464542708 0.180743525 1 0.257238652 1

DB00757 1 3.211609425 1.75379047 0.753349628 0.1585137 1 0.12201228 1

DB00758 1 0.467207301 1.75379047 0.753349628 0.1585137 1 0.12201228 1

DB00759 1.5 1.078287817 1.745287633 0.542671379 0.325634379 1 0.346299761 1

DB00761 2 1.648064965 1.770629523 0.307115021 0.772424541 1 0.645919359 1

DB00762 2 2.601679685 1.745287633 0.542671379 0.680596551 1 0.645919359 1

DB00763 1 2.30716836 1.75379047 0.753349628 0.1585137 1 0.12201228 1

DB00764 1 0.947267307 1.745287633 0.542671379 0.08481895 1 0.12201228 1

DB00765 1 0.918566816 1.75379047 0.753349628 0.1585137 1 0.12201228 1

DB00767 1 1.955597646 1.770629523 0.307115021 0.006049327 1 0.12201228 1

DB00768 1.2857143 1.414955798 1.736866356 0.292454388 0.061458983 1 0.234170029 1

DB00769 1 1.23003433 1.75379047 0.753349628 0.1585137 1 0.12201228 1

DB00770 1.3333333 1.991122117 1.757244872 0.464542708 0.180743525 1 0.257238652 1

DB00771 1 3.013650197 1.75379047 0.753349628 0.1585137 1 0.12201228 1

DB00772 2 1.447306054 1.75379047 0.753349628 0.628097886 1 0.645919359 1

DB00773 2 1.649191264 1.745287633 0.542671379 0.680596551 1 0.645919359 1

DB00774 1.8888889 1.621341674 1.753207263 0.264393406 0.696087596 1 0.580546569 1

DB00775 1 3.015195153 1.745287633 0.542671379 0.08481895 1 0.12201228 1

DB00776 2 1.953894653 1.759747174 0.221112233 0.861385463 1 0.645919359 1

DB00777 1 1.607757981 1.759693587 0.247672276 0.001079887 1 0.12201228 1

DB00778 1 0.885551451 1.75379047 0.753349628 0.1585137 1 0.12201228 1

DB00780 1.3333333 1.616952701 1.770629523 0.307115021 0.07724 1 0.257238652 1

DB00782 1 1.837276242 1.75379047 0.753349628 0.1585137 1 0.12201228 1

DB00783 1.2857143 1.607893613 1.736866356 0.292454388 0.061458983 1 0.234170029 1

DB00784 1.5 2.336935201 1.745287633 0.542671379 0.325634379 1 0.346299761 1

DB00785 1.5 2.048852179 1.753692879 0.367879736 0.245220308 1 0.346299761 1

DB00786 1.5652174 1.555375099 1.750829326 0.165162414 0.13054585 1 0.384019936 1

DB00788 1.5 1.60022628 1.745287633 0.542671379 0.325634379 1 0.346299761 1

DB00789 2 1.093197696 1.75379047 0.753349628 0.628097886 1 0.645919359 1

DB00790 1.5 1.42142746 1.745287633 0.542671379 0.325634379 1 0.346299761 1

DB00792 1 2.021685117 1.75379047 0.753349628 0.1585137 1 0.12201228 1

DB00794 1.95 1.927789138 1.758534835 0.16776606 0.873119713 1 0.616904557 1

DB00795 1.4444444 2.146738715 1.753207263 0.264393406 0.121440487 1 0.315312538 1

DB00796 1 1.934947405 1.75379047 0.753349628 0.1585137 1 0.12201228 1

DB00797 1.1666667 2.021478648 1.770629523 0.307115021 0.024616471 1 0.181824474 1

DB00798 2 2.745533792 1.75379047 0.753349628 0.628097886 1 0.645919359 1

DB00799 1.5 1.822633848 1.753692879 0.367879736 0.245220308 1 0.346299761 1

DB00800 1.25 1.632102931 1.761473636 0.259363203 0.024302735 1 0.217648936 1

DB00801 2 1.852670222 1.759334798 0.191532594 0.895536882 1 0.645919359 1

DB00802 1 2.690398586 1.75379047 0.753349628 0.1585137 1 0.12201228 1

DB00804 1 2.591952673 1.745287633 0.542671379 0.08481895 1 0.12201228 1

DB00805 1.2222222 1.608580737 1.753207263 0.264393406 0.022304951 1 0.205277671 1

DB00806 1 2.238812372 1.757244872 0.464542708 0.051541591 1 0.12201228 1

DB00807 2 1.844766483 1.75379047 0.753349628 0.628097886 1 0.645919359 1

DB00808 2 0.539979201 1.75379047 0.753349628 0.628097886 1 0.645919359 1

DB00809 1 1.774104606 1.753692879 0.367879736 0.020243381 1 0.12201228 1

DB00810 1 1.280035344 1.75379047 0.753349628 0.1585137 1 0.12201228 1

DB00811 2 1.831576839 1.745287633 0.542671379 0.680596551 1 0.645919359 1

DB00812 1.6666667 1.811359242 1.757244872 0.464542708 0.422702925 1 0.444812957 1

DB00813 1 1.76610722 1.753692879 0.367879736 0.020243381 1 0.12201228 1

DB00814 1.5 1.133610626 1.745287633 0.542671379 0.325634379 1 0.346299761 1

DB00816 2 1.204791733 1.75379047 0.753349628 0.628097886 1 0.645919359 1

DB00818 2 1.498451384 1.755399642 0.180590294 0.912203427 1 0.645919359 1

DB00819 1.875 1.441471845 1.761473636 0.259363203 0.669202435 1 0.572174329 1

DB00820 1 1.577418235 1.745287633 0.542671379 0.08481895 1 0.12201228 1

DB00821 1.5 2.03505382 1.745287633 0.542671379 0.325634379 1 0.346299761 1

DB00822 2 2.287540957 1.745287633 0.542671379 0.680596551 1 0.645919359 1

DB00823 1 0.891432024 1.745287633 0.542671379 0.08481895 1 0.12201228 1

DB00824 1 1.388803787 1.757979426 0.337271213 0.012307606 1 0.12201228 1

DB00825 1.7692308 1.744384725 1.754873752 0.200318376 0.528568192 1 0.507619602 1

DB00829 2 1.258283418 1.759334798 0.191532594 0.895536882 1 0.645919359 1

DB00830 1 1.22168983 1.745287633 0.542671379 0.08481895 1 0.12201228 1

DB00831 1.3333333 2.36736754 1.770629523 0.307115021 0.07724 1 0.257238652 1

DB00834 1 2.156047027 1.753692879 0.367879736 0.020243381 1 0.12201228 1

DB00835 1 1.520737155 1.770629523 0.307115021 0.006049327 1 0.12201228 1

DB00836 1.1428571 1.891698003 1.736866356 0.292454388 0.021121533 1 0.172307877 1

DB00837 1.5 2.340852941 1.745287633 0.542671379 0.325634379 1 0.346299761 1

DB00838 1 1.433097891 1.75379047 0.753349628 0.1585137 1 0.12201228 1

DB00839 1 1.001522924 1.745287633 0.542671379 0.08481895 1 0.12201228 1

DB00841 1.3333333 2.418920271 1.770629523 0.307115021 0.07724 1 0.257238652 1

DB00842 2 1.906185079 1.759334798 0.191532594 0.895536882 1 0.645919359 1

DB00843 1.5333333 1.943944286 1.760312929 0.204781867 0.133845177 1 0.365415847 1

DB00844 1 1.658977418 1.757244872 0.464542708 0.051541591 1 0.12201228 1

DB00845 1 1.456900427 1.745287633 0.542671379 0.08481895 1 0.12201228 1

DB00846 1 0.641256474 1.75379047 0.753349628 0.1585137 1 0.12201228 1

DB00847 1 1.851117973 1.745287633 0.542671379 0.08481895 1 0.12201228 1

DB00848 1.5 0.360398295 1.745287633 0.542671379 0.325634379 1 0.346299761 1

DB00849 1.8181818 1.826265336 1.759747174 0.221112233 0.604216321 1 0.537624777 1

DB00850 1.3333333 1.37898532 1.757244872 0.464542708 0.180743525 1 0.257238652 1

DB00851 2 1.551845165 1.745287633 0.542671379 0.680596551 1 0.645919359 1

DB00852 1.3809524 2.062422821 1.763665626 0.163272037 0.009538526 1 0.281435865 1

DB00854 1 0.991925271 1.757244872 0.464542708 0.051541591 1 0.12201228 1

DB00855 2 1.134869764 1.75379047 0.753349628 0.628097886 1 0.645919359 1

DB00857 2 1.519524611 1.75379047 0.753349628 0.628097886 1 0.645919359 1

DB00858 1 2.751682648 1.75379047 0.753349628 0.1585137 1 0.12201228 1

DB00860 1 0.33085724 1.75379047 0.753349628 0.1585137 1 0.12201228 1

DB00861 1.5 1.326562201 1.745287633 0.542671379 0.325634379 1 0.346299761 1

DB00862 1 1.339017954 1.757244872 0.464542708 0.051541591 1 0.12201228 1

DB00863 2 1.037393528 1.745287633 0.542671379 0.680596551 1 0.645919359 1

DB00864 2 1.931522633 1.75379047 0.753349628 0.628097886 1 0.645919359 1

DB00865 1 0.907499435 1.757244872 0.464542708 0.051541591 1 0.12201228 1

DB00866 1.5 1.913112683 1.753692879 0.367879736 0.245220308 1 0.346299761 1

DB00867 2 1.976058942 1.75379047 0.753349628 0.628097886 1 0.645919359 1

DB00869 2 1.970346735 1.753692879 0.367879736 0.748421796 1 0.645919359 1

DB00870 1.5 2.404026755 1.745287633 0.542671379 0.325634379 1 0.346299761 1

DB00871 1.6666667 1.333344992 1.757244872 0.464542708 0.422702925 1 0.444812957 1

DB00872 1 2.574281958 1.75379047 0.753349628 0.1585137 1 0.12201228 1

DB00874 1.8571429 1.897388312 1.736866356 0.292454388 0.659561248 1 0.56136264 1

DB00875 1.2 1.194695061 1.757979426 0.337271213 0.049023756 1 0.195688273 1

DB00876 1 0.766457214 1.75379047 0.753349628 0.1585137 1 0.12201228 1

DB00877 1.5 2.005905219 1.745287633 0.542671379 0.325634379 1 0.346299761 1

DB00880 2 1.352296048 1.757244872 0.464542708 0.699362524 1 0.645919359 1

DB00881 1 1.569818597 1.75379047 0.753349628 0.1585137 1 0.12201228 1

DB00882 1 1.741883445 1.745287633 0.542671379 0.08481895 1 0.12201228 1

DB00883 1 2.407644706 1.75379047 0.753349628 0.1585137 1 0.12201228 1

DB00884 2 1.171569644 1.75379047 0.753349628 0.628097886 1 0.645919359 1

DB00886 1 1.29825867 1.745287633 0.542671379 0.08481895 1 0.12201228 1

DB00887 1.8 1.658439215 1.757979426 0.337271213 0.549575873 1 0.52649583 1

DB00889 1 0.857025765 1.75379047 0.753349628 0.1585137 1 0.12201228 1

DB00890 1 1.868332562 1.745287633 0.542671379 0.08481895 1 0.12201228 1

DB00892 2 1.409069988 1.75379047 0.753349628 0.628097886 1 0.645919359 1

DB00893 2 1.933061921 1.753692879 0.367879736 0.748421796 1 0.645919359 1

DB00894 1 3.04609204 1.75379047 0.753349628 0.1585137 1 0.12201228 1

DB00895 1 2.472903088 1.745287633 0.542671379 0.08481895 1 0.12201228 1

DB00896 1 0.634968497 1.75379047 0.753349628 0.1585137 1 0.12201228 1

DB00897 2 1.981546761 1.759334798 0.191532594 0.895536882 1 0.645919359 1

DB00898 1.8823529 1.738215911 1.752899726 0.106742633 0.887389165 1 0.576610962 1

DB00899 1 1.660813313 1.757244872 0.464542708 0.051541591 1 0.12201228 1

DB00900 2 2.61580158 1.75379047 0.753349628 0.628097886 1 0.645919359 1

DB00901 2 1.737867181 1.75379047 0.753349628 0.628097886 1 0.645919359 1

DB00902 1 0.543969805 1.75379047 0.753349628 0.1585137 1 0.12201228 1

DB00903 1.5 1.914343758 1.753692879 0.367879736 0.245220308 1 0.346299761 1

DB00904 1.2 1.526647359 1.757979426 0.337271213 0.049023756 1 0.195688273 1

DB00905 1 1.521294662 1.757244872 0.464542708 0.051541591 1 0.12201228 1

DB00906 2 1.588337751 1.75379047 0.753349628 0.628097886 1 0.645919359 1

DB00907 1.7222222 2.060964205 1.755399642 0.180590294 0.427117876 1 0.478762265 1

DB00908 1.3333333 1.68156832 1.770629523 0.307115021 0.07724 1 0.257238652 1

DB00909 1.8965517 1.739780841 1.749502043 0.145004846 0.84473288 1 0.585150403 1

DB00910 2 2.706256967 1.75379047 0.753349628 0.628097886 1 0.645919359 1

DB00912 1 1.550481939 1.745287633 0.542671379 0.08481895 1 0.12201228 1

DB00913 1 0.300350078 1.75379047 0.753349628 0.1585137 1 0.12201228 1

DB00914 2 1.451903102 1.745287633 0.542671379 0.680596551 1 0.645919359 1

DB00915 1.8 1.69603623 1.757979426 0.337271213 0.549575873 1 0.52649583 1

DB00917 1.2 1.91346708 1.757979426 0.337271213 0.049023756 1 0.195688273 1

DB00918 1 2.009284371 1.745287633 0.542671379 0.08481895 1 0.12201228 1

DB00920 1.5 2.001228394 1.745287633 0.542671379 0.325634379 1 0.346299761 1

DB00921 1 2.414681414 1.753692879 0.367879736 0.020243381 1 0.12201228 1

DB00922 1.5 1.592468455 1.753692879 0.367879736 0.245220308 1 0.346299761 1

DB00924 1.2222222 1.556041813 1.753207263 0.264393406 0.022304951 1 0.205277671 1

DB00925 1.125 1.870693937 1.761473636 0.259363203 0.007064127 1 0.165382999 1

DB00926 1.6666667 1.481002522 1.770629523 0.307115021 0.367487831 1 0.444812957 1

DB00927 2 0.120340665 1.75379047 0.753349628 0.628097886 1 0.645919359 1

DB00928 1 1.530320418 1.75379047 0.753349628 0.1585137 1 0.12201228 1

DB00929 1.25 1.301656421 1.753692879 0.367879736 0.085471804 1 0.217648936 1

DB00933 1 2.134029359 1.745287633 0.542671379 0.08481895 1 0.12201228 1

DB00934 1 1.261427913 1.748450254 0.194078018 5.75E-05 0.311843753 0.12201228 1

DB00935 1 1.632925753 1.770629523 0.307115021 0.006049327 1 0.12201228 1

DB00936 1.3333333 1.814246894 1.757244872 0.464542708 0.180743525 1 0.257238652 1

DB00937 1 2.901424194 1.745287633 0.542671379 0.08481895 1 0.12201228 1

DB00938 1.6666667 1.614866371 1.757244872 0.464542708 0.422702925 1 0.444812957 1

DB00939 1.8 1.990703822 1.757979426 0.337271213 0.549575873 1 0.52649583 1

DB00940 1.1666667 1.668709525 1.770629523 0.307115021 0.024616471 1 0.181824474 1

DB00941 2 1.414356828 1.75379047 0.753349628 0.628097886 1 0.645919359 1

DB00942 1 1.824376358 1.75379047 0.753349628 0.1585137 1 0.12201228 1

DB00944 2 1.44138564 1.745287633 0.542671379 0.680596551 1 0.645919359 1

DB00945 1.2222222 1.687926306 1.755736801 0.142706481 9.25E-05 0.50103422 0.205277671 1

DB00946 2 1.312817276 1.75379047 0.753349628 0.628097886 1 0.645919359 1

DB00947 1 1.131999457 1.75379047 0.753349628 0.1585137 1 0.12201228 1

DB00949 1.5 1.659344373 1.745287633 0.542671379 0.325634379 1 0.346299761 1

DB00950 1 -0.073359393 1.75379047 0.753349628 0.1585137 1 0.12201228 1

DB00951 1.3333333 0.850004967 1.757244872 0.464542708 0.180743525 1 0.257238652 1

DB00952 1 1.90205369 1.753692879 0.367879736 0.020243381 1 0.12201228 1

DB00953 1 2.028375843 1.757244872 0.464542708 0.051541591 1 0.12201228 1

DB00956 1.3333333 2.251341701 1.757244872 0.464542708 0.180743525 1 0.257238652 1

DB00957 1 1.247358149 1.757244872 0.464542708 0.051541591 1 0.12201228 1

DB00959 1 2.013847862 1.745287633 0.542671379 0.08481895 1 0.12201228 1

DB00960 1.4 1.363498468 1.757979426 0.337271213 0.14425424 1 0.291413148 1

DB00961 2 2.252984789 1.75379047 0.753349628 0.628097886 1 0.645919359 1

DB00962 2 1.401696466 1.75379047 0.753349628 0.628097886 1 0.645919359 1

DB00963 1.5 2.170641902 1.745287633 0.542671379 0.325634379 1 0.346299761 1

DB00964 1 2.278192804 1.757244872 0.464542708 0.051541591 1 0.12201228 1

DB00966 1 2.586205916 1.745287633 0.542671379 0.08481895 1 0.12201228 1

DB00967 1 1.591305858 1.75379047 0.753349628 0.1585137 1 0.12201228 1

DB00968 1 2.503097805 1.745287633 0.542671379 0.08481895 1 0.12201228 1

DB00969 1 2.603516317 1.75379047 0.753349628 0.1585137 1 0.12201228 1

DB00970 2 1.547583421 1.745287633 0.542671379 0.680596551 1 0.645919359 1

DB00972 1.5 1.769366776 1.753692879 0.367879736 0.245220308 1 0.346299761 1

DB00973 1.6666667 1.583764088 1.757244872 0.464542708 0.422702925 1 0.444812957 1

DB00975 1 1.956683464 1.753692879 0.367879736 0.020243381 1 0.12201228 1

DB00977 1 2.422030146 1.745287633 0.542671379 0.08481895 1 0.12201228 1

DB00978 2 2.272378924 1.75379047 0.753349628 0.628097886 1 0.645919359 1

DB00979 1 2.289499501 1.75379047 0.753349628 0.1585137 1 0.12201228 1

DB00980 1 1.296512489 1.745287633 0.542671379 0.08481895 1 0.12201228 1

DB00981 2 1.213794429 1.757244872 0.464542708 0.699362524 1 0.645919359 1

DB00982 1.5 1.253552516 1.745287633 0.542671379 0.325634379 1 0.346299761 1

DB00983 1.6666667 1.99216326 1.757244872 0.464542708 0.422702925 1 0.444812957 1

DB00984 1 1.390612482 1.75379047 0.753349628 0.1585137 1 0.12201228 1

DB00985 1 2.256724795 1.75379047 0.753349628 0.1585137 1 0.12201228 1

DB00986 1 1.878298737 1.757244872 0.464542708 0.051541591 1 0.12201228 1

DB00987 2 1.309268421 1.75379047 0.753349628 0.628097886 1 0.645919359 1

DB00988 1.2857143 1.869525899 1.773663076 0.187305714 0.004592468 1 0.234170029 1

DB00989 2 1.741017584 1.745287633 0.542671379 0.680596551 1 0.645919359 1

DB00990 1 2.52210661 1.75379047 0.753349628 0.1585137 1 0.12201228 1

DB00991 1.5 1.293935677 1.745287633 0.542671379 0.325634379 1 0.346299761 1

DB00993 1 2.976982839 1.75379047 0.753349628 0.1585137 1 0.12201228 1

DB00994 1 0.796412213 1.75379047 0.753349628 0.1585137 1 0.12201228 1

DB00995 1.5 1.355175392 1.745287633 0.542671379 0.325634379 1 0.346299761 1

DB00996 1.8333333 1.478371177 1.770629523 0.307115021 0.580889804 1 0.546876824 1

DB00997 2 2.205063097 1.745287633 0.542671379 0.680596551 1 0.645919359 1

DB00998 1 1.30207175 1.745287633 0.542671379 0.08481895 1 0.12201228 1

DB00999 1.5 1.505674594 1.745287633 0.542671379 0.325634379 1 0.346299761 1

DB01001 1.6666667 1.78332372 1.757244872 0.464542708 0.422702925 1 0.444812957 1

DB01002 2 2.419546989 1.75379047 0.753349628 0.628097886 1 0.645919359 1

DB01003 2 0.892824361 1.75379047 0.753349628 0.628097886 1 0.645919359 1

DB01005 1 0.512718077 1.75379047 0.753349628 0.1585137 1 0.12201228 1

DB01006 1 2.143266665 1.75379047 0.753349628 0.1585137 1 0.12201228 1

DB01009 1.3333333 1.632126509 1.757244872 0.464542708 0.180743525 1 0.257238652 1

DB01010 2 2.078752291 1.745287633 0.542671379 0.680596551 1 0.645919359 1

DB01011 2 1.179025251 1.75379047 0.753349628 0.628097886 1 0.645919359 1

DB01012 1 1.072609605 1.75379047 0.753349628 0.1585137 1 0.12201228 1

DB01013 1.3333333 1.876880176 1.757244872 0.464542708 0.180743525 1 0.257238652 1

DB01014 1.5 1.595141048 1.753692879 0.367879736 0.245220308 1 0.346299761 1

DB01016 1.5 1.850768459 1.761473636 0.259363203 0.156694354 1 0.346299761 1

DB01017 1.1578947 1.784058523 1.755240506 0.172383815 0.000264911 1 0.178280747 1

DB01018 1 2.302885249 1.745287633 0.542671379 0.08481895 1 0.12201228 1

DB01019 1 1.966211795 1.753692879 0.367879736 0.020243381 1 0.12201228 1

DB01020 1 1.403894018 1.75379047 0.753349628 0.1585137 1 0.12201228 1

DB01021 2 1.991845828 1.757979426 0.337271213 0.763493223 1 0.645919359 1

DB01022 2 2.226142971 1.745287633 0.542671379 0.680596551 1 0.645919359 1

DB01023 1.6153846 1.861746854 1.754873752 0.200318376 0.243108818 1 0.413824896 1

DB01024 2 1.902471021 1.745287633 0.542671379 0.680596551 1 0.645919359 1

DB01025 1.3333333 1.802202787 1.757244872 0.464542708 0.180743525 1 0.257238652 1

DB01026 1.2 1.48474316 1.757979426 0.337271213 0.049023756 1 0.195688273 1

DB01028 2 2.073092927 1.763665626 0.163272037 0.926119903 1 0.645919359 1

DB01029 1 1.871941885 1.745287633 0.542671379 0.08481895 1 0.12201228 1

DB01030 2 1.869419892 1.745287633 0.542671379 0.680596551 1 0.645919359 1

DB01031 2 2.384081734 1.745287633 0.542671379 0.680596551 1 0.645919359 1

DB01032 1.4 1.882947395 1.757979426 0.337271213 0.14425424 1 0.291413148 1

DB01033 1.5 2.164041046 1.753692879 0.367879736 0.245220308 1 0.346299761 1

DB01034 1 1.933223986 1.75379047 0.753349628 0.1585137 1 0.12201228 1

DB01035 1 0.484616362 1.745287633 0.542671379 0.08481895 1 0.12201228 1

DB01036 1 1.775231853 1.757979426 0.337271213 0.012307606 1 0.12201228 1

DB01037 1 0.771563045 1.745287633 0.542671379 0.08481895 1 0.12201228 1

DB01038 1.6666667 1.243767824 1.757244872 0.464542708 0.422702925 1 0.444812957 1

DB01039 1 1.602133498 1.757244872 0.464542708 0.051541591 1 0.12201228 1

DB01041 1 1.465805828 1.736866356 0.292454388 0.005874511 1 0.12201228 1

DB01043 1.8965517 1.736691994 1.749502043 0.145004846 0.84473288 1 0.585150403 1

DB01045 1 1.916489395 1.75379047 0.753349628 0.1585137 1 0.12201228 1

DB01046 2 2.418496742 1.75379047 0.753349628 0.628097886 1 0.645919359 1

DB01047 1.5 1.812718461 1.745287633 0.542671379 0.325634379 1 0.346299761 1

DB01048 1 1.469463337 1.75379047 0.753349628 0.1585137 1 0.12201228 1

DB01049 1.3333333 1.65259265 1.760507724 0.1309174 0.000551332 1 0.257238652 1

DB01050 1.3 1.671825369 1.759693587 0.247672276 0.031722796 1 0.240967937 1

DB01053 1.6666667 1.814516686 1.757244872 0.464542708 0.422702925 1 0.444812957 1

DB01054 1.7142857 1.515767549 1.736866356 0.292454388 0.4692279 1 0.473897096 1

DB01055 1.3333333 2.740587249 1.757244872 0.464542708 0.180743525 1 0.257238652 1

DB01057 2 2.722389788 1.75379047 0.753349628 0.628097886 1 0.645919359 1

DB01059 2 0.99820429 1.75379047 0.753349628 0.628097886 1 0.645919359 1

DB01062 1 1.052644572 1.757244872 0.464542708 0.051541591 1 0.12201228 1

DB01063 1 1.893436957 1.745287633 0.542671379 0.08481895 1 0.12201228 1

DB01064 1.25 1.70075199 1.761473636 0.259363203 0.024302735 1 0.217648936 1

DB01065 1.1 1.702684073 1.759693587 0.247672276 0.003865763 1 0.155995064 1

DB01067 1 0.440757915 1.745287633 0.542671379 0.08481895 1 0.12201228 1

DB01068 1.9411765 1.722138242 1.748450254 0.194078018 0.839653527 1 0.611710091 1

DB01069 1.4638269 1.702429282 1.752457508 0.080835985 0.000178102 0.963354106 0.325990629 1

DB01070 2 2.248059392 1.75379047 0.753349628 0.628097886 1 0.645919359 1

DB01071 1 1.259547555 1.75379047 0.753349628 0.1585137 1 0.12201228 1

DB01073 1.6666667 0.996422143 1.757244872 0.464542708 0.422702925 1 0.444812957 1

DB01074 1 1.616470397 1.757244872 0.464542708 0.051541591 1 0.12201228 1

DB01075 1 2.304784676 1.745287633 0.542671379 0.08481895 1 0.12201228 1

DB01076 1.2 1.234077768 1.757979426 0.337271213 0.049023756 1 0.195688273 1

DB01077 2 1.999060796 1.753692879 0.367879736 0.748421796 1 0.645919359 1

DB01078 2 2.064105874 1.75379047 0.753349628 0.628097886 1 0.645919359 1

DB01079 1.25 1.864794545 1.753692879 0.367879736 0.085471804 1 0.217648936 1

DB01080 2 2.234727818 1.75379047 0.753349628 0.628097886 1 0.645919359 1

DB01081 1 1.624231147 1.745287633 0.542671379 0.08481895 1 0.12201228 1

DB01082 1 1.973368406 1.75379047 0.753349628 0.1585137 1 0.12201228 1

DB01083 1.3333333 2.077126055 1.757244872 0.464542708 0.180743525 1 0.257238652 1

DB01084 1 0.530368453 1.75379047 0.753349628 0.1585137 1 0.12201228 1

DB01085 1 1.557519096 1.753692879 0.367879736 0.020243381 1 0.12201228 1

DB01086 2 2.73300873 1.75379047 0.753349628 0.628097886 1 0.645919359 1

DB01087 1.5 2.122076027 1.745287633 0.542671379 0.325634379 1 0.346299761 1

DB01088 1.2857143 1.97155934 1.736866356 0.292454388 0.061458983 1 0.234170029 1

DB01090 1.6666667 2.330312578 1.757244872 0.464542708 0.422702925 1 0.444812957 1

DB01091 2 1.660623138 1.75379047 0.753349628 0.628097886 1 0.645919359 1

DB01092 2 2.081858404 1.757244872 0.464542708 0.699362524 1 0.645919359 1

DB01094 1.3333333 1.994882935 1.757244872 0.464542708 0.180743525 1 0.257238652 1

DB01095 1.5 0.747216623 1.745287633 0.542671379 0.325634379 1 0.346299761 1

DB01097 1.3333333 1.322242738 1.757244872 0.464542708 0.180743525 1 0.257238652 1

DB01098 1.5 1.478978013 1.745287633 0.542671379 0.325634379 1 0.346299761 1

DB01099 1 2.041507018 1.75379047 0.753349628 0.1585137 1 0.12201228 1

DB01100 1 1.502225684 1.753692879 0.367879736 0.020243381 1 0.12201228 1

DB01101 1 3.341301305 1.75379047 0.753349628 0.1585137 1 0.12201228 1

DB01102 1.6666667 1.714049318 1.757244872 0.464542708 0.422702925 1 0.444812957 1

DB01103 1.3333333 2.085787329 1.757244872 0.464542708 0.180743525 1 0.257238652 1

DB01104 1.1666667 2.012264893 1.770629523 0.307115021 0.024616471 1 0.181824474 1

DB01105 1 2.664386416 1.757244872 0.464542708 0.051541591 1 0.12201228 1

DB01106 1 2.318935297 1.745287633 0.542671379 0.08481895 1 0.12201228 1

DB01107 2 1.790011672 1.759334798 0.191532594 0.895536882 1 0.645919359 1

DB01108 1 2.17844706 1.753692879 0.367879736 0.020243381 1 0.12201228 1

DB01109 1.3636364 2.030711943 1.759747174 0.221112233 0.036610823 1 0.272511088 1

DB01110 1.7878788 1.743729253 1.754322285 0.088696116 0.647407744 1 0.519064484 1

DB01113 1 1.052998714 1.75379047 0.753349628 0.1585137 1 0.12201228 1

DB01114 1 2.108785073 1.753692879 0.367879736 0.020243381 1 0.12201228 1

DB01115 1.5 1.942867637 1.759693587 0.247672276 0.147195593 1 0.346299761 1

DB01116 2 1.296715974 1.75379047 0.753349628 0.628097886 1 0.645919359 1

DB01117 2 1.754960306 1.75379047 0.753349628 0.628097886 1 0.645919359 1

DB01118 1.6 1.775704228 1.758534835 0.16776606 0.172335664 1 0.404623142 1

DB01119 1.6 1.936753796 1.757979426 0.337271213 0.319747599 1 0.404623142 1

DB01120 1 0.997751968 1.75379047 0.753349628 0.1585137 1 0.12201228 1

DB01121 2 1.211178037 1.75379047 0.753349628 0.628097886 1 0.645919359 1

DB01122 2 1.49727216 1.75379047 0.753349628 0.628097886 1 0.645919359 1

DB01123 1 2.013257074 1.75379047 0.753349628 0.1585137 1 0.12201228 1

DB01124 1.5 2.056154247 1.745287633 0.542671379 0.325634379 1 0.346299761 1

DB01125 2 1.635184837 1.75379047 0.753349628 0.628097886 1 0.645919359 1

DB01126 1.5 1.682546648 1.745287633 0.542671379 0.325634379 1 0.346299761 1

DB01127 1 2.165804108 1.75379047 0.753349628 0.1585137 1 0.12201228 1

DB01128 1 1.114143538 1.75379047 0.753349628 0.1585137 1 0.12201228 1

DB01129 2 0.083670046 1.75379047 0.753349628 0.628097886 1 0.645919359 1

DB01130 1 1.747858978 1.75379047 0.753349628 0.1585137 1 0.12201228 1

DB01131 2 1.046022822 1.75379047 0.753349628 0.628097886 1 0.645919359 1

DB01132 1 1.100633998 1.745287633 0.542671379 0.08481895 1 0.12201228 1

DB01133 1.8 1.732571252 1.76622404 0.151326267 0.588309913 1 0.52649583 1

DB01134 2 1.736140116 1.75379047 0.753349628 0.628097886 1 0.645919359 1

DB01135 1.5 1.74108078 1.745287633 0.542671379 0.325634379 1 0.346299761 1

DB01136 1.2352941 1.781261945 1.748450254 0.194078018 0.004095768 1 0.211046617 1

DB01138 1.6666667 1.907516694 1.757244872 0.464542708 0.422702925 1 0.444812957 1

DB01142 1.1052632 1.671486594 1.755240506 0.172383815 8.15E-05 0.441391506 0.157941711 1

DB01143 1 2.283242887 1.75379047 0.753349628 0.1585137 1 0.12201228 1

DB01144 2 2.275901517 1.757979426 0.337271213 0.763493223 1 0.645919359 1

DB01146 1 2.252529717 1.745287633 0.542671379 0.08481895 1 0.12201228 1

DB01148 1 1.582519821 1.745287633 0.542671379 0.08481895 1 0.12201228 1

DB01149 1 1.217867226 1.759693587 0.247672276 0.001079887 1 0.12201228 1

DB01151 1.0952381 1.624479458 1.763665626 0.163272037 2.12E-05 0.115139392 0.154247512 1

DB01154 1.6666667 1.742617918 1.757244872 0.464542708 0.422702925 1 0.444812957 1

DB01156 1.25 1.79468235 1.753692879 0.367879736 0.085471804 1 0.217648936 1

DB01157 2 2.240509392 1.75379047 0.753349628 0.628097886 1 0.645919359 1

DB01158 2 1.818003834 1.75379047 0.753349628 0.628097886 1 0.645919359 1

DB01159 1.8484848 1.699745809 1.751829593 0.13569543 0.761859011 1 0.556103492 1

DB01160 1.5 1.87322866 1.745287633 0.542671379 0.325634379 1 0.346299761 1

DB01161 1.5 1.544742334 1.745287633 0.542671379 0.325634379 1 0.346299761 1

DB01162 1.3333333 1.674537373 1.770629523 0.307115021 0.07724 1 0.257238652 1

DB01165 2 2.288167665 1.75379047 0.753349628 0.628097886 1 0.645919359 1

DB01166 1 2.078543771 1.75379047 0.753349628 0.1585137 1 0.12201228 1

DB01168 1 2.066950222 1.745287633 0.542671379 0.08481895 1 0.12201228 1

DB01169 1 1.680358163 1.759693587 0.247672276 0.001079887 1 0.12201228 1

DB01170 1 1.315691045 1.75379047 0.753349628 0.1585137 1 0.12201228 1

DB01171 1 1.28478371 1.745287633 0.542671379 0.08481895 1 0.12201228 1

DB01173 1.7142857 1.838002394 1.736866356 0.292454388 0.4692279 1 0.473897096 1

DB01174 1.7692308 1.539618752 1.754873752 0.200318376 0.528568192 1 0.507619602 1

DB01175 1 2.174089494 1.760312929 0.204781867 0.000102492 0.554685511 0.12201228 1

DB01176 1 2.355521098 1.745287633 0.542671379 0.08481895 1 0.12201228 1

DB01177 2 1.551398353 1.75379047 0.753349628 0.628097886 1 0.645919359 1

DB01178 2 1.509172906 1.75379047 0.753349628 0.628097886 1 0.645919359 1

DB01179 1.6666667 1.677231338 1.757244872 0.464542708 0.422702925 1 0.444812957 1

DB01180 1 2.149343011 1.75379047 0.753349628 0.1585137 1 0.12201228 1

DB01181 1 1.729966885 1.75379047 0.753349628 0.1585137 1 0.12201228 1

DB01182 1.6666667 1.334581726 1.757244872 0.464542708 0.422702925 1 0.444812957 1

DB01183 1.1428571 1.778706103 1.736866356 0.292454388 0.021121533 1 0.172307877 1

DB01184 1 1.165156103 1.745287633 0.542671379 0.08481895 1 0.12201228 1

DB01185 1 2.143340769 1.753692879 0.367879736 0.020243381 1 0.12201228 1

DB01186 1.1176471 1.674104014 1.748450254 0.194078018 0.000576507 1 0.162584666 1

DB01188 2 1.639611041 1.75379047 0.753349628 0.628097886 1 0.645919359 1

DB01189 1.9310345 1.747605986 1.749502043 0.145004846 0.894697966 1 0.605714689 1

DB01191 1 2.608821737 1.745287633 0.542671379 0.08481895 1 0.12201228 1

DB01192 1 1.462008556 1.745287633 0.542671379 0.08481895 1 0.12201228 1

DB01193 2 1.46499741 1.745287633 0.542671379 0.680596551 1 0.645919359 1

DB01194 2 2.210386585 1.753692879 0.367879736 0.748421796 1 0.645919359 1

DB01195 1.3333333 2.237541611 1.757244872 0.464542708 0.180743525 1 0.257238652 1

DB01196 1.5 1.47802791 1.753692879 0.367879736 0.245220308 1 0.346299761 1

DB01197 1.2 1.502742783 1.757979426 0.337271213 0.049023756 1 0.195688273 1

DB01198 2 1.505706808 1.757979426 0.337271213 0.763493223 1 0.645919359 1

DB01199 1.75 1.598545571 1.753692879 0.367879736 0.495995374 1 0.495810565 1

DB01200 1.1111111 1.766998643 1.755399642 0.180590294 0.000180077 0.973858354 0.160123241 1

DB01202 2 2.52661909 1.745287633 0.542671379 0.680596551 1 0.645919359 1

DB01203 2 2.043507396 1.745287633 0.542671379 0.680596551 1 0.645919359 1

DB01204 2 2.625575076 1.75379047 0.753349628 0.628097886 1 0.645919359 1

DB01205 2 1.624110021 1.759334798 0.191532594 0.895536882 1 0.645919359 1

DB01206 2 2.619944048 1.75379047 0.753349628 0.628097886 1 0.645919359 1

DB01207 1 2.750683718 1.75379047 0.753349628 0.1585137 1 0.12201228 1

DB01208 2 1.296648382 1.75379047 0.753349628 0.628097886 1 0.645919359 1

DB01209 1 1.854551638 1.745287633 0.542671379 0.08481895 1 0.12201228 1

DB01210 2 1.484029003 1.745287633 0.542671379 0.680596551 1 0.645919359 1

DB01211 1 2.532490225 1.757244872 0.464542708 0.051541591 1 0.12201228 1

DB01212 1.75 1.425852427 1.753692879 0.367879736 0.495995374 1 0.495810565 1

DB01213 1.75 1.652694241 1.753692879 0.367879736 0.495995374 1 0.495810565 1

DB01214 2 1.277865155 1.745287633 0.542671379 0.680596551 1 0.645919359 1

DB01215 2 1.832486345 1.759334798 0.191532594 0.895536882 1 0.645919359 1

DB01216 1.6666667 1.608448527 1.757244872 0.464542708 0.422702925 1 0.444812957 1

DB01217 1 2.32135643 1.75379047 0.753349628 0.1585137 1 0.12201228 1

DB01218 1 1.601262104 1.75379047 0.753349628 0.1585137 1 0.12201228 1

DB01219 1 2.01337007 1.75379047 0.753349628 0.1585137 1 0.12201228 1

DB01220 1 0.813094808 1.75379047 0.753349628 0.1585137 1 0.12201228 1

DB01221 1.0869565 1.855719054 1.750829326 0.165162414 2.92E-05 0.158245969 0.151239231 1

DB01222 1 1.754037739 1.745287633 0.542671379 0.08481895 1 0.12201228 1

DB01223 1 2.136475166 1.757244872 0.464542708 0.051541591 1 0.12201228 1

DB01224 1.1153846 2.04380143 1.752565715 0.14361026 4.56E-06 0.024808071 0.161729849 1

DB01225 2 0.133521845 1.745287633 0.542671379 0.680596551 1 0.645919359 1

DB01226 1.5 2.147806 1.753692879 0.367879736 0.245220308 1 0.346299761 1

DB01227 1.3333333 0.603823347 1.757244872 0.464542708 0.180743525 1 0.257238652 1

DB01229 1.6666667 1.702522994 1.770629523 0.307115021 0.367487831 1 0.444812957 1

DB01231 1 1.206243899 1.757244872 0.464542708 0.051541591 1 0.12201228 1

DB01233 1.25 2.064575694 1.753692879 0.367879736 0.085471804 1 0.217648936 1

DB01234 1 1.607803588 1.757979426 0.337271213 0.012307606 1 0.12201228 1

DB01235 1.4 1.47922867 1.757979426 0.337271213 0.14425424 1 0.291413148 1

DB01236 2 1.952025907 1.763665626 0.163272037 0.926119903 1 0.645919359 1

DB01237 1 2.28032783 1.75379047 0.753349628 0.1585137 1 0.12201228 1

DB01238 1.2727273 1.771846487 1.758339856 0.115069137 1.22E-05 0.066311542 0.228083394 1

DB01239 1.28 1.8156491 1.76622404 0.151326267 0.000656589 1 0.231480882 1

DB01240 1.6666667 1.97795212 1.757244872 0.464542708 0.422702925 1 0.444812957 1

DB01241 1.25 1.638803513 1.753692879 0.367879736 0.085471804 1 0.217648936 1

DB01242 1 1.013213149 1.770629523 0.307115021 0.006049327 1 0.12201228 1

DB01244 1.5 1.866006085 1.759693587 0.247672276 0.147195593 1 0.346299761 1

DB01245 2 2.244563496 1.753692879 0.367879736 0.748421796 1 0.645919359 1

DB01246 1 1.346297814 1.75379047 0.753349628 0.1585137 1 0.12201228 1

DB01247 1 1.731637118 1.745287633 0.542671379 0.08481895 1 0.12201228 1

DB01248 1.6666667 1.975936977 1.770629523 0.307115021 0.367487831 1 0.444812957 1

DB01250 1.5 0.917453815 1.745287633 0.542671379 0.325634379 1 0.346299761 1

DB01251 1.5 1.505694818 1.745287633 0.542671379 0.325634379 1 0.346299761 1

DB01252 1 1.992413254 1.745287633 0.542671379 0.08481895 1 0.12201228 1

DB01253 1 1.49542334 1.75379047 0.753349628 0.1585137 1 0.12201228 1

DB01254 0.8605261 2.175140237 1.757638217 0.154827625 3.43E-09 1.87E-05 0.083842562 1

DB01255 2 0.797358607 1.75379047 0.753349628 0.628097886 1 0.645919359 1

DB01257 1 1.92013175 1.75379047 0.753349628 0.1585137 1 0.12201228 1

DB01259 1 2.152013032 1.745287633 0.542671379 0.08481895 1 0.12201228 1

DB01260 1 1.6000666 1.75379047 0.753349628 0.1585137 1 0.12201228 1

DB01261 1 2.523081734 1.75379047 0.753349628 0.1585137 1 0.12201228 1

DB01262 1 0.959456586 1.75379047 0.753349628 0.1585137 1 0.12201228 1

DB01267 1.0666667 1.740892135 1.760312929 0.204781867 0.000352992 1 0.144034932 1

DB01268 1 1.733935079 1.761473636 0.259363203 0.001662717 1 0.12201228 1

DB01269 1 1.357460072 1.75379047 0.753349628 0.1585137 1 0.12201228 1

DB01271 2 1.949654031 1.75379047 0.753349628 0.628097886 1 0.645919359 1

DB01272 1 1.174683664 1.75379047 0.753349628 0.1585137 1 0.12201228 1

DB01273 2 1.263148249 1.757979426 0.337271213 0.763493223 1 0.645919359 1

DB01274 2 3.269625066 1.75379047 0.753349628 0.628097886 1 0.645919359 1

DB01275 1.3333333 2.238347781 1.757244872 0.464542708 0.180743525 1 0.257238652 1

DB01276 2 1.341021611 1.75379047 0.753349628 0.628097886 1 0.645919359 1

DB01277 1.3333333 0.923484077 1.757244872 0.464542708 0.180743525 1 0.257238652 1

DB01278 2 0.623188287 1.757244872 0.464542708 0.699362524 1 0.645919359 1

DB01279 2 2.820792248 1.75379047 0.753349628 0.628097886 1 0.645919359 1

DB01280 2 0.633595439 1.75379047 0.753349628 0.628097886 1 0.645919359 1

DB01281 1 1.794178573 1.745287633 0.542671379 0.08481895 1 0.12201228 1

DB01282 1 1.739872555 1.75379047 0.753349628 0.1585137 1 0.12201228 1

DB01283 1.5 2.197037559 1.745287633 0.542671379 0.325634379 1 0.346299761 1

DB01284 2 0.835705003 1.75379047 0.753349628 0.628097886 1 0.645919359 1

DB01285 2 2.634525926 1.745287633 0.542671379 0.680596551 1 0.645919359 1

DB01288 1.6666667 1.02287041 1.757244872 0.464542708 0.422702925 1 0.444812957 1

DB01289 2 1.304591382 1.75379047 0.753349628 0.628097886 1 0.645919359 1

DB01291 2 2.118420851 1.745287633 0.542671379 0.680596551 1 0.645919359 1

DB01295 1.6666667 1.585872923 1.757244872 0.464542708 0.422702925 1 0.444812957 1

DB01296 1 1.571650296 1.753692879 0.367879736 0.020243381 1 0.12201228 1

DB01297 2 2.233762412 1.75379047 0.753349628 0.628097886 1 0.645919359 1

DB01303 1 1.970487161 1.757979426 0.337271213 0.012307606 1 0.12201228 1

DB01306 1 1.467544493 1.75379047 0.753349628 0.1585137 1 0.12201228 1

DB01307 1 2.137390955 1.75379047 0.753349628 0.1585137 1 0.12201228 1

DB01309 1 2.084365663 1.75379047 0.753349628 0.1585137 1 0.12201228 1

DB01324 2 1.953631784 1.75379047 0.753349628 0.628097886 1 0.645919359 1

DB01325 2 1.869509486 1.757979426 0.337271213 0.763493223 1 0.645919359 1

DB01327 1.3333333 1.737592751 1.757244872 0.464542708 0.180743525 1 0.257238652 1

DB01336 1.5 1.789009896 1.745287633 0.542671379 0.325634379 1 0.346299761 1

DB01337 1.3333333 1.370465278 1.757244872 0.464542708 0.180743525 1 0.257238652 1

DB01338 1.3333333 1.738534069 1.757244872 0.464542708 0.180743525 1 0.257238652 1

DB01339 2 2.935174489 1.75379047 0.753349628 0.628097886 1 0.645919359 1

DB01340 1 1.58578382 1.75379047 0.753349628 0.1585137 1 0.12201228 1

DB01341 2 2.195915416 1.75379047 0.753349628 0.628097886 1 0.645919359 1

DB01342 1 1.44136524 1.75379047 0.753349628 0.1585137 1 0.12201228 1

DB01345 2 4.080985989 1.75379047 0.753349628 0.628097886 1 0.645919359 1

DB01346 2 1.960390504 1.753692879 0.367879736 0.748421796 1 0.645919359 1

DB01347 1 3.193397135 1.75379047 0.753349628 0.1585137 1 0.12201228 1

DB01348 1 2.104562565 1.75379047 0.753349628 0.1585137 1 0.12201228 1

DB01349 1 2.201977519 1.745287633 0.542671379 0.08481895 1 0.12201228 1

DB01351 1.9 1.841920565 1.759693587 0.247672276 0.714473128 1 0.587218383 1

DB01352 1.9 2.056803509 1.759693587 0.247672276 0.714473128 1 0.587218383 1

DB01353 1.9 1.867422469 1.759693587 0.247672276 0.714473128 1 0.587218383 1

DB01354 1.9 1.405237027 1.759693587 0.247672276 0.714473128 1 0.587218383 1

DB01355 1.9 2.168247551 1.759693587 0.247672276 0.714473128 1 0.587218383 1

DB01356 1.75 1.54696642 1.753692879 0.367879736 0.495995374 1 0.495810565 1

DB01357 1 1.583890908 1.75379047 0.753349628 0.1585137 1 0.12201228 1

DB01359 1.5 2.795885534 1.753692879 0.367879736 0.245220308 1 0.346299761 1

DB01363 1.25 1.318608472 1.763093512 0.211303302 0.00758631 1 0.217648936 1

DB01364 1.5 2.505185469 1.753692879 0.367879736 0.245220308 1 0.346299761 1

DB01365 1.2222222 1.908322081 1.753207263 0.264393406 0.022304951 1 0.205277671 1

DB01366 2 2.511552592 1.75379047 0.753349628 0.628097886 1 0.645919359 1

DB01367 1.5 1.357690851 1.745287633 0.542671379 0.325634379 1 0.346299761 1

DB01370 1.5 1.736953791 1.753692879 0.367879736 0.245220308 1 0.346299761 1

DB01373 1.5263158 1.713556474 1.755240506 0.172383815 0.092090012 1 0.361361649 1

DB01375 1.75 1.413805679 1.753692879 0.367879736 0.495995374 1 0.495810565 1

DB01378 2 0.08288187 1.75379047 0.753349628 0.628097886 1 0.645919359 1

DB01380 1 0.168299467 1.75379047 0.753349628 0.1585137 1 0.12201228 1

DB01381 1.6666667 1.587952585 1.770629523 0.307115021 0.367487831 1 0.444812957 1

DB01382 1.5 1.975174947 1.745287633 0.542671379 0.325634379 1 0.346299761 1

DB01384 1 3.019279931 1.75379047 0.753349628 0.1585137 1 0.12201228 1

DB01388 1.8181818 1.466415947 1.759747174 0.221112233 0.604216321 1 0.537624777 1

DB01392 1.2941176 2.07326419 1.748450254 0.194078018 0.009616613 1 0.23815585 1

DB01393 1.2857143 1.687797447 1.736866356 0.292454388 0.061458983 1 0.234170029 1

DB01394 1 1.626247468 1.75379047 0.753349628 0.1585137 1 0.12201228 1

DB01395 1.25 2.112040443 1.753692879 0.367879736 0.085471804 1 0.217648936 1

DB01396 2 1.666759148 1.75379047 0.753349628 0.628097886 1 0.645919359 1

DB01397 1.5 2.454692921 1.745287633 0.542671379 0.325634379 1 0.346299761 1

DB01399 1.5 1.670019094 1.745287633 0.542671379 0.325634379 1 0.346299761 1

DB01400 2 2.480017954 1.75379047 0.753349628 0.628097886 1 0.645919359 1

DB01401 1.5 1.861242035 1.745287633 0.542671379 0.325634379 1 0.346299761 1

DB01403 1.1052632 1.680524414 1.755240506 0.172383815 8.15E-05 0.441391506 0.157941711 1

DB01404 1 1.200272617 1.757244872 0.464542708 0.051541591 1 0.12201228 1

DB01406 1 1.817758754 1.757979426 0.337271213 0.012307606 1 0.12201228 1

DB01407 1.4 1.592680487 1.757979426 0.337271213 0.14425424 1 0.291413148 1

DB01408 2 0.979215457 1.75379047 0.753349628 0.628097886 1 0.645919359 1

DB01409 1 2.168480253 1.757979426 0.337271213 0.012307606 1 0.12201228 1

DB01410 1 1.303513691 1.75379047 0.753349628 0.1585137 1 0.12201228 1

DB01411 1.2 2.623794018 1.757979426 0.337271213 0.049023756 1 0.195688273 1

DB01412 1 0.999438236 1.757244872 0.464542708 0.051541591 1 0.12201228 1

DB01418 2 1.356340767 1.75379047 0.753349628 0.628097886 1 0.645919359 1

DB01419 1.5 1.926097114 1.745287633 0.542671379 0.325634379 1 0.346299761 1

DB01420 1 2.439847303 1.75379047 0.753349628 0.1585137 1 0.12201228 1

DB01422 2 1.625242548 1.75379047 0.753349628 0.628097886 1 0.645919359 1

DB01425 1 2.731538324 1.75379047 0.753349628 0.1585137 1 0.12201228 1

DB01427 1 1.927767816 1.770629523 0.307115021 0.006049327 1 0.12201228 1

DB01428 1 1.570279161 1.753692879 0.367879736 0.020243381 1 0.12201228 1

DB01429 1 1.416601443 1.75379047 0.753349628 0.1585137 1 0.12201228 1

DB01430 2 2.362565721 1.75379047 0.753349628 0.628097886 1 0.645919359 1

DB01431 1 2.580685864 1.745287633 0.542671379 0.08481895 1 0.12201228 1

DB01433 1 1.989200624 1.75379047 0.753349628 0.1585137 1 0.12201228 1

DB01435 1.5 2.975551427 1.745287633 0.542671379 0.325634379 1 0.346299761 1

DB01436 1.6666667 1.492265413 1.757244872 0.464542708 0.422702925 1 0.444812957 1

DB01437 2 2.185033606 1.759334798 0.191532594 0.895536882 1 0.645919359 1

DB01438 2 0.758971053 1.75379047 0.753349628 0.628097886 1 0.645919359 1

DB01439 1 1.177392816 1.757244872 0.464542708 0.051541591 1 0.12201228 1

DB01440 2 1.613082598 1.75379047 0.753349628 0.628097886 1 0.645919359 1

DB01442 1.1428571 1.959606028 1.736866356 0.292454388 0.021121533 1 0.172307877 1

DB01444 1 1.919490449 1.745287633 0.542671379 0.08481895 1 0.12201228 1

DB01452 1.25 1.298623096 1.753692879 0.367879736 0.085471804 1 0.217648936 1

DB01454 1 1.755099873 1.770629523 0.307115021 0.006049327 1 0.12201228 1

DB01463 1 1.775685494 1.75379047 0.753349628 0.1585137 1 0.12201228 1

DB01466 1 2.45510449 1.75379047 0.753349628 0.1585137 1 0.12201228 1

DB01472 1 1.654335041 1.770629523 0.307115021 0.006049327 1 0.12201228 1

DB01481 1 2.816236913 1.75379047 0.753349628 0.1585137 1 0.12201228 1

DB01483 1.9 1.245077298 1.759693587 0.247672276 0.714473128 1 0.587218383 1

DB01488 1.6666667 1.442672866 1.757244872 0.464542708 0.422702925 1 0.444812957 1

DB01489 2 1.678602774 1.759334798 0.191532594 0.895536882 1 0.645919359 1

DB01496 1.9 1.484424948 1.759693587 0.247672276 0.714473128 1 0.587218383 1

DB01497 1 1.786821533 1.770629523 0.307115021 0.006049327 1 0.12201228 1

DB01511 2 2.098175727 1.759334798 0.191532594 0.895536882 1 0.645919359 1

DB01520 1.8333333 1.932870484 1.770629523 0.307115021 0.580889804 1 0.546876824 1

DB01535 1 1.54199751 1.757244872 0.464542708 0.051541591 1 0.12201228 1

DB01536 1 2.224508738 1.757244872 0.464542708 0.051541591 1 0.12201228 1

DB01537 1 1.822106294 1.745287633 0.542671379 0.08481895 1 0.12201228 1

DB01541 1 1.010824769 1.75379047 0.753349628 0.1585137 1 0.12201228 1

DB01544 2 2.196866463 1.759334798 0.191532594 0.895536882 1 0.645919359 1

DB01545 2 2.024720656 1.759334798 0.191532594 0.895536882 1 0.645919359 1

DB01548 1 2.018200798 1.753692879 0.367879736 0.020243381 1 0.12201228 1

DB01549 1.5 1.444103298 1.745287633 0.542671379 0.325634379 1 0.346299761 1

DB01553 2 1.94589719 1.759334798 0.191532594 0.895536882 1 0.645919359 1

DB01558 2 2.306410025 1.759334798 0.191532594 0.895536882 1 0.645919359 1

DB01559 2 1.961576764 1.759334798 0.191532594 0.895536882 1 0.645919359 1

DB01561 1 0.907342408 1.75379047 0.753349628 0.1585137 1 0.12201228 1

DB01564 1 1.648877577 1.75379047 0.753349628 0.1585137 1 0.12201228 1

DB01565 1 1.445484519 1.757979426 0.337271213 0.012307606 1 0.12201228 1

DB01567 2 1.754537578 1.759334798 0.191532594 0.895536882 1 0.645919359 1

DB01569 1 1.998211325 1.75379047 0.753349628 0.1585137 1 0.12201228 1

DB01571 1 1.997345322 1.757244872 0.464542708 0.051541591 1 0.12201228 1

DB01576 1.1111111 1.685204441 1.753207263 0.264393406 0.007579386 1 0.160123241 1

DB01577 1.2 1.571999792 1.759693587 0.247672276 0.01191636 1 0.195688273 1

DB01579 1 1.362964817 1.757244872 0.464542708 0.051541591 1 0.12201228 1

DB01580 1.6666667 1.51603169 1.757244872 0.464542708 0.422702925 1 0.444812957 1

DB01583 1 1.778872311 1.745287633 0.542671379 0.08481895 1 0.12201228 1

DB01586 2 1.461646918 1.745287633 0.542671379 0.680596551 1 0.645919359 1

DB01587 2 1.746448093 1.759334798 0.191532594 0.895536882 1 0.645919359 1

DB01588 2 1.747765385 1.759334798 0.191532594 0.895536882 1 0.645919359 1

DB01589 2 1.509391954 1.759334798 0.191532594 0.895536882 1 0.645919359 1

DB01590 1 1.661548622 1.75379047 0.753349628 0.1585137 1 0.12201228 1

DB01591 1 1.201564284 1.757979426 0.337271213 0.012307606 1 0.12201228 1

DB01592 1.6666667 1.411913923 1.763093512 0.211303302 0.324071624 1 0.444812957 1

DB01593 1.3905527 1.796916959 1.75792302 0.071843204 1.58E-07 0.000860968 0.28644396 1

DB01594 2 1.595853702 1.759334798 0.191532594 0.895536882 1 0.645919359 1

DB01595 2 1.7386251 1.748450254 0.194078018 0.902534121 1 0.645919359 1

DB01597 2 0.738439501 1.75379047 0.753349628 0.628097886 1 0.645919359 1

DB01599 2 1.755014735 1.745287633 0.542671379 0.680596551 1 0.645919359 1

DB01600 1.5 1.063326687 1.745287633 0.542671379 0.325634379 1 0.346299761 1

DB01608 1.25 2.342840888 1.753692879 0.367879736 0.085471804 1 0.217648936 1

DB01611 1 1.732966488 1.757244872 0.464542708 0.051541591 1 0.12201228 1

DB01612 1 1.665203234 1.75379047 0.753349628 0.1585137 1 0.12201228 1

DB01613 1 1.228314347 1.745287633 0.542671379 0.08481895 1 0.12201228 1

DB01614 1.1666667 2.450931967 1.770629523 0.307115021 0.024616471 1 0.181824474 1

DB01615 1 2.088641102 1.75379047 0.753349628 0.1585137 1 0.12201228 1

DB01616 1 3.199937376 1.75379047 0.753349628 0.1585137 1 0.12201228 1

DB01618 1 1.87515186 1.753692879 0.367879736 0.020243381 1 0.12201228 1

DB01619 1 1.412765525 1.75379047 0.753349628 0.1585137 1 0.12201228 1

DB01620 1 1.793883817 1.757244872 0.464542708 0.051541591 1 0.12201228 1

DB01621 1.25 1.459067908 1.753692879 0.367879736 0.085471804 1 0.217648936 1

DB01622 1.1666667 1.746533205 1.770629523 0.307115021 0.024616471 1 0.181824474 1

DB01623 1.3333333 1.976872662 1.757244872 0.464542708 0.180743525 1 0.257238652 1

DB01624 1.2857143 1.254251488 1.736866356 0.292454388 0.061458983 1 0.234170029 1

DB01625 1 1.989368482 1.745287633 0.542671379 0.08481895 1 0.12201228 1

DB01626 1 2.467811896 1.745287633 0.542671379 0.08481895 1 0.12201228 1

DB01628 1 3.008329124 1.75379047 0.753349628 0.1585137 1 0.12201228 1

DB01629 1 0.662135396 1.75379047 0.753349628 0.1585137 1 0.12201228 1

DB01630 1 1.560690358 1.75379047 0.753349628 0.1585137 1 0.12201228 1

DB01631 1 0.661987218 1.75379047 0.753349628 0.1585137 1 0.12201228 1

DB01632 1.6666667 1.4696782 1.757244872 0.464542708 0.422702925 1 0.444812957 1

DB01640 1 2.134181639 1.75379047 0.753349628 0.1585137 1 0.12201228 1

DB01643 1.5 1.623465585 1.745287633 0.542671379 0.325634379 1 0.346299761 1

DB01644 2 2.363148027 1.75379047 0.753349628 0.628097886 1 0.645919359 1

DB01645 0.6979036 1.701579489 1.754873752 0.200318376 6.59E-08 0.000358711 0.051549994 1

DB01646 1 1.768885041 1.75379047 0.753349628 0.1585137 1 0.12201228 1

DB01647 1 1.884307808 1.75379047 0.753349628 0.1585137 1 0.12201228 1

DB01649 1.5 2.419974139 1.745287633 0.542671379 0.325634379 1 0.346299761 1

DB01651 2 2.970581738 1.75379047 0.753349628 0.628097886 1 0.645919359 1

DB01656 1 1.817598522 1.757244872 0.464542708 0.051541591 1 0.12201228 1

DB01662 2 2.975583855 1.75379047 0.753349628 0.628097886 1 0.645919359 1

DB01664 2 1.962015119 1.75379047 0.753349628 0.628097886 1 0.645919359 1

DB01665 2 1.599415439 1.75379047 0.753349628 0.628097886 1 0.645919359 1

DB01666 1 1.192723762 1.75379047 0.753349628 0.1585137 1 0.12201228 1

DB01667 2 2.211607178 1.75379047 0.753349628 0.628097886 1 0.645919359 1

DB01671 2 2.126696047 1.75379047 0.753349628 0.628097886 1 0.645919359 1

DB01672 1 1.505357601 1.75379047 0.753349628 0.1585137 1 0.12201228 1

DB01675 2 2.051665253 1.75379047 0.753349628 0.628097886 1 0.645919359 1

DB01677 2 2.821959339 1.745287633 0.542671379 0.680596551 1 0.645919359 1

DB01678 1 2.541300769 1.75379047 0.753349628 0.1585137 1 0.12201228 1

DB01681 2 2.260901508 1.75379047 0.753349628 0.628097886 1 0.645919359 1

DB01685 2 1.048610552 1.75379047 0.753349628 0.628097886 1 0.645919359 1

DB01686 1 1.846156643 1.745287633 0.542671379 0.08481895 1 0.12201228 1

DB01689 2 1.363127033 1.75379047 0.753349628 0.628097886 1 0.645919359 1

DB01692 1 1.63159094 1.75379047 0.753349628 0.1585137 1 0.12201228 1

DB01694 1.4 1.773213555 1.757979426 0.337271213 0.14425424 1 0.291413148 1

DB01695 1 1.809943027 1.75379047 0.753349628 0.1585137 1 0.12201228 1

DB01698 1 1.744057122 1.745287633 0.542671379 0.08481895 1 0.12201228 1

DB01699 2 1.586956757 1.75379047 0.753349628 0.628097886 1 0.645919359 1

DB01700 1 2.470594547 1.75379047 0.753349628 0.1585137 1 0.12201228 1

DB01704 2 2.942967714 1.75379047 0.753349628 0.628097886 1 0.645919359 1

DB01705 2 0.729756685 1.75379047 0.753349628 0.628097886 1 0.645919359 1

DB01708 1.7666667 1.79094072 1.760507724 0.1309174 0.518761218 1 0.506045228 1

DB01709 2 1.794908448 1.75379047 0.753349628 0.628097886 1 0.645919359 1

DB01710 1 1.719181607 1.75379047 0.753349628 0.1585137 1 0.12201228 1

DB01711 2 2.118300534 1.75379047 0.753349628 0.628097886 1 0.645919359 1

DB01712 2 1.822216697 1.75379047 0.753349628 0.628097886 1 0.645919359 1

DB01717 2 2.089344562 1.75379047 0.753349628 0.628097886 1 0.645919359 1

DB01723 2 2.447842046 1.75379047 0.753349628 0.628097886 1 0.645919359 1

DB01725 1.3333333 2.059148477 1.757244872 0.464542708 0.180743525 1 0.257238652 1

DB01727 2 2.130251677 1.757244872 0.464542708 0.699362524 1 0.645919359 1

DB01728 1 1.937014515 1.75379047 0.753349628 0.1585137 1 0.12201228 1

DB01731 1 2.469377372 1.75379047 0.753349628 0.1585137 1 0.12201228 1

DB01733 1 2.586744218 1.75379047 0.753349628 0.1585137 1 0.12201228 1

DB01734 1 0.150821625 1.75379047 0.753349628 0.1585137 1 0.12201228 1

DB01736 2 1.476563359 1.75379047 0.753349628 0.628097886 1 0.645919359 1

DB01737 2 2.826743304 1.75379047 0.753349628 0.628097886 1 0.645919359 1

DB01741 2 1.378137744 1.75379047 0.753349628 0.628097886 1 0.645919359 1

DB01742 1 2.424059309 1.75379047 0.753349628 0.1585137 1 0.12201228 1

DB01744 1.75 1.689569361 1.753692879 0.367879736 0.495995374 1 0.495810565 1

DB01745 2 2.50953727 1.75379047 0.753349628 0.628097886 1 0.645919359 1

DB01748 2 2.214156913 1.75379047 0.753349628 0.628097886 1 0.645919359 1

DB01750 1 3.194704079 1.75379047 0.753349628 0.1585137 1 0.12201228 1

DB01752 1.7272727 1.752828362 1.759747174 0.221112233 0.441617815 1 0.481859977 1

DB01754 1 2.241538171 1.75379047 0.753349628 0.1585137 1 0.12201228 1

DB01756 2 2.364341407 1.75379047 0.753349628 0.628097886 1 0.645919359 1

DB01760 2 0.961523477 1.75379047 0.753349628 0.628097886 1 0.645919359 1

DB01761 1 2.122316982 1.75379047 0.753349628 0.1585137 1 0.12201228 1

DB01762 2 1.295285595 1.75379047 0.753349628 0.628097886 1 0.645919359 1

DB01763 2 2.365433465 1.75379047 0.753349628 0.628097886 1 0.645919359 1

DB01765 2 1.75364378 1.75379047 0.753349628 0.628097886 1 0.645919359 1

DB01766 1.6666667 1.628968009 1.757244872 0.464542708 0.422702925 1 0.444812957 1

DB01767 1.5 2.310499336 1.745287633 0.542671379 0.325634379 1 0.346299761 1

DB01771 2 2.743267724 1.75379047 0.753349628 0.628097886 1 0.645919359 1

DB01772 1 1.156795228 1.75379047 0.753349628 0.1585137 1 0.12201228 1

DB01776 1 1.944545261 1.75379047 0.753349628 0.1585137 1 0.12201228 1

DB01777 1 2.333830805 1.75379047 0.753349628 0.1585137 1 0.12201228 1

DB01780 1 1.502358692 1.75379047 0.753349628 0.1585137 1 0.12201228 1

DB01782 1.6666667 1.903956836 1.757244872 0.464542708 0.422702925 1 0.444812957 1

DB01784 2 0.919825978 1.75379047 0.753349628 0.628097886 1 0.645919359 1

DB01785 2 1.69774627 1.745287633 0.542671379 0.680596551 1 0.645919359 1

DB01790 1 1.652961722 1.75379047 0.753349628 0.1585137 1 0.12201228 1

DB01791 1 1.743079882 1.757244872 0.464542708 0.051541591 1 0.12201228 1

DB01793 1 0.536640953 1.75379047 0.753349628 0.1585137 1 0.12201228 1

DB01805 2 2.028038371 1.745287633 0.542671379 0.680596551 1 0.645919359 1

DB01807 1 2.203206924 1.75379047 0.753349628 0.1585137 1 0.12201228 1

DB01809 1 2.690237864 1.75379047 0.753349628 0.1585137 1 0.12201228 1

DB01810 2 2.490565796 1.75379047 0.753349628 0.628097886 1 0.645919359 1

DB01811 1 1.267079009 1.75379047 0.753349628 0.1585137 1 0.12201228 1

DB01812 1.4 1.927703566 1.759693587 0.247672276 0.073209586 1 0.291413148 1

DB01818 2 1.84930934 1.75379047 0.753349628 0.628097886 1 0.645919359 1

DB01819 1.5 1.048052964 1.745287633 0.542671379 0.325634379 1 0.346299761 1

DB01820 1 1.984356678 1.75379047 0.753349628 0.1585137 1 0.12201228 1

DB01821 1 1.821449243 1.745287633 0.542671379 0.08481895 1 0.12201228 1

DB01823 2 1.536972475 1.75379047 0.753349628 0.628097886 1 0.645919359 1

DB01826 1 0.795098945 1.75379047 0.753349628 0.1585137 1 0.12201228 1

DB01827 1 1.394394214 1.75379047 0.753349628 0.1585137 1 0.12201228 1

DB01830 -6.0684256 1.83543417 1.75379047 0.753349628 1.48E-25 8.11E-22 1.02E-33 5.62E-30

DB01831 1 0.290734623 1.75379047 0.753349628 0.1585137 1 0.12201228 1

DB01832 2 1.298131174 1.75379047 0.753349628 0.628097886 1 0.645919359 1

DB01833 1 2.596418814 1.75379047 0.753349628 0.1585137 1 0.12201228 1

DB01834 1 1.041629818 1.745287633 0.542671379 0.08481895 1 0.12201228 1

DB01835 1 1.727058567 1.75379047 0.753349628 0.1585137 1 0.12201228 1

DB01836 2 0.580624749 1.75379047 0.753349628 0.628097886 1 0.645919359 1

DB01837 2 1.107748517 1.75379047 0.753349628 0.628097886 1 0.645919359 1

DB01840 2 2.128931718 1.75379047 0.753349628 0.628097886 1 0.645919359 1

DB01842 2 1.307131526 1.75379047 0.753349628 0.628097886 1 0.645919359 1

DB01843 2 1.444664475 1.75379047 0.753349628 0.628097886 1 0.645919359 1

DB01844 2 0.807344504 1.745287633 0.542671379 0.680596551 1 0.645919359 1

DB01852 2 1.767834822 1.75379047 0.753349628 0.628097886 1 0.645919359 1

DB01854 1 2.801375882 1.75379047 0.753349628 0.1585137 1 0.12201228 1

DB01858 2 1.656510713 1.75379047 0.753349628 0.628097886 1 0.645919359 1

DB01860 2 1.707658246 1.75379047 0.753349628 0.628097886 1 0.645919359 1

DB01861 2 2.092995144 1.757244872 0.464542708 0.699362524 1 0.645919359 1

DB01863 1.25 2.083013266 1.761473636 0.259363203 0.024302735 1 0.217648936 1

DB01864 1.3333333 1.074085038 1.757244872 0.464542708 0.180743525 1 0.257238652 1

DB01866 1 2.013872644 1.75379047 0.753349628 0.1585137 1 0.12201228 1

DB01870 2 0.379116002 1.75379047 0.753349628 0.628097886 1 0.645919359 1

DB01871 2 3.045714705 1.75379047 0.753349628 0.628097886 1 0.645919359 1

DB01873 1.5454545 1.539921983 1.759747174 0.221112233 0.166233199 1 0.372453903 1

DB01876 2 1.788227952 1.75379047 0.753349628 0.628097886 1 0.645919359 1

DB01877 2 1.044534792 1.75379047 0.753349628 0.628097886 1 0.645919359 1

DB01878 1 2.483322525 1.745287633 0.542671379 0.08481895 1 0.12201228 1

DB01880 2 0.585971962 1.75379047 0.753349628 0.628097886 1 0.645919359 1

DB01884 1 1.921872971 1.75379047 0.753349628 0.1585137 1 0.12201228 1

DB01888 1 2.206570181 1.75379047 0.753349628 0.1585137 1 0.12201228 1

DB01889 1 1.115098901 1.75379047 0.753349628 0.1585137 1 0.12201228 1

DB01890 1 2.985476254 1.745287633 0.542671379 0.08481895 1 0.12201228 1

DB01892 1.6666667 2.304581527 1.757244872 0.464542708 0.422702925 1 0.444812957 1

DB01893 1 2.168641015 1.75379047 0.753349628 0.1585137 1 0.12201228 1

DB01897 1 1.344556843 1.75379047 0.753349628 0.1585137 1 0.12201228 1

DB01901 1.5 2.230768066 1.745287633 0.542671379 0.325634379 1 0.346299761 1

DB01902 2 2.548993304 1.745287633 0.542671379 0.680596551 1 0.645919359 1

DB01905 1.5 1.405982178 1.745287633 0.542671379 0.325634379 1 0.346299761 1

DB01908 1 1.172545515 1.75379047 0.753349628 0.1585137 1 0.12201228 1

DB01915 1.4 1.271092976 1.757979426 0.337271213 0.14425424 1 0.291413148 1

DB01917 1.75 0.896435341 1.753692879 0.367879736 0.495995374 1 0.495810565 1

DB01919 1 1.593171049 1.75379047 0.753349628 0.1585137 1 0.12201228 1

DB01922 2 2.567025002 1.75379047 0.753349628 0.628097886 1 0.645919359 1

DB01926 1 2.360764278 1.75379047 0.753349628 0.1585137 1 0.12201228 1

DB01927 1 0.857693398 1.75379047 0.753349628 0.1585137 1 0.12201228 1

DB01929 2 3.076274297 1.75379047 0.753349628 0.628097886 1 0.645919359 1

DB01931 2 2.592259988 1.75379047 0.753349628 0.628097886 1 0.645919359 1

DB01933 1 1.711021874 1.75379047 0.753349628 0.1585137 1 0.12201228 1

DB01939 2 0.484706868 1.75379047 0.753349628 0.628097886 1 0.645919359 1

DB01940 1 2.427664046 1.75379047 0.753349628 0.1585137 1 0.12201228 1

DB01941 1 1.625639076 1.75379047 0.753349628 0.1585137 1 0.12201228 1

DB01942 1.7272727 1.80939204 1.759747174 0.221112233 0.441617815 1 0.481859977 1

DB01946 1.5 1.255921898 1.745287633 0.542671379 0.325634379 1 0.346299761 1

DB01947 1 2.467076679 1.75379047 0.753349628 0.1585137 1 0.12201228 1

DB01948 1 0.653827806 1.75379047 0.753349628 0.1585137 1 0.12201228 1

DB01949 1 0.661325694 1.75379047 0.753349628 0.1585137 1 0.12201228 1

DB01950 1 1.35041885 1.75379047 0.753349628 0.1585137 1 0.12201228 1

DB01951 2 1.652019963 1.75379047 0.753349628 0.628097886 1 0.645919359 1

DB01953 1 0.947714748 1.75379047 0.753349628 0.1585137 1 0.12201228 1

DB01954 1 1.515953957 1.745287633 0.542671379 0.08481895 1 0.12201228 1

DB01955 1.3333333 1.500191573 1.757244872 0.464542708 0.180743525 1 0.257238652 1

DB01956 1.952381 1.59227151 1.763665626 0.163272037 0.876125471 1 0.61830272 1

DB01958 2 2.605456065 1.75379047 0.753349628 0.628097886 1 0.645919359 1

DB01959 1 1.05660936 1.745287633 0.542671379 0.08481895 1 0.12201228 1

DB01960 1.5 1.117059808 1.745287633 0.542671379 0.325634379 1 0.346299761 1

DB01961 2 1.283827173 1.75379047 0.753349628 0.628097886 1 0.645919359 1

DB01962 1 1.825969364 1.745287633 0.542671379 0.08481895 1 0.12201228 1

DB01963 2 0.751000815 1.75379047 0.753349628 0.628097886 1 0.645919359 1

DB01964 2 1.010765926 1.75379047 0.753349628 0.628097886 1 0.645919359 1

DB01966 1 1.636759724 1.75379047 0.753349628 0.1585137 1 0.12201228 1

DB01969 1 2.449255751 1.75379047 0.753349628 0.1585137 1 0.12201228 1

DB01970 1 1.366660786 1.75379047 0.753349628 0.1585137 1 0.12201228 1

DB01972 1.3333333 1.836365065 1.770629523 0.307115021 0.07724 1 0.257238652 1

DB01977 1 3.122809824 1.75379047 0.753349628 0.1585137 1 0.12201228 1

DB01979 2 0.437474491 1.75379047 0.753349628 0.628097886 1 0.645919359 1

DB01983 1 2.387620709 1.75379047 0.753349628 0.1585137 1 0.12201228 1

DB01988 1 1.344443892 1.75379047 0.753349628 0.1585137 1 0.12201228 1

DB01989 2 1.521607768 1.75379047 0.753349628 0.628097886 1 0.645919359 1

DB01990 1 1.177957075 1.75379047 0.753349628 0.1585137 1 0.12201228 1

DB01992 1.5714286 1.531520086 1.736866356 0.292454388 0.285803159 1 0.387676968 1

DB01996 2 2.225502833 1.745287633 0.542671379 0.680596551 1 0.645919359 1

DB01997 1 1.539976741 1.757244872 0.464542708 0.051541591 1 0.12201228 1

DB01998 1 0.019875275 1.75379047 0.753349628 0.1585137 1 0.12201228 1

DB02001 2 1.790865517 1.75379047 0.753349628 0.628097886 1 0.645919359 1

DB02004 1 1.241712382 1.75379047 0.753349628 0.1585137 1 0.12201228 1

DB02007 1.6666667 1.890305466 1.757244872 0.464542708 0.422702925 1 0.444812957 1

DB02008 1 0.30581062 1.75379047 0.753349628 0.1585137 1 0.12201228 1

DB02010 0.533198 1.754643025 1.754873752 0.200318376 5.35E-10 2.92E-06 0.029813571 1

DB02011 2 2.217608728 1.75379047 0.753349628 0.628097886 1 0.645919359 1

DB02014 1 3.164202979 1.75379047 0.753349628 0.1585137 1 0.12201228 1

DB02016 2 2.132633075 1.75379047 0.753349628 0.628097886 1 0.645919359 1

DB02018 2 2.265933662 1.75379047 0.753349628 0.628097886 1 0.645919359 1

DB02020 2 0.901107957 1.75379047 0.753349628 0.628097886 1 0.645919359 1

DB02021 2 1.382024188 1.745287633 0.542671379 0.680596551 1 0.645919359 1

DB02027 1 2.470672954 1.745287633 0.542671379 0.08481895 1 0.12201228 1

DB02028 2 2.104203299 1.75379047 0.753349628 0.628097886 1 0.645919359 1

DB02029 2 3.098540217 1.75379047 0.753349628 0.628097886 1 0.645919359 1

DB02032 1 0.92732864 1.75379047 0.753349628 0.1585137 1 0.12201228 1

DB02034 2 0.769025304 1.75379047 0.753349628 0.628097886 1 0.645919359 1

DB02039 1 0.285761097 1.75379047 0.753349628 0.1585137 1 0.12201228 1

DB02044 1 1.46993395 1.757244872 0.464542708 0.051541591 1 0.12201228 1

DB02045 2 1.148442743 1.75379047 0.753349628 0.628097886 1 0.645919359 1

DB02046 1 2.013990314 1.75379047 0.753349628 0.1585137 1 0.12201228 1

DB02047 2 0.502469799 1.75379047 0.753349628 0.628097886 1 0.645919359 1

DB02048 1 0.832011799 1.75379047 0.753349628 0.1585137 1 0.12201228 1

DB02049 2 2.251029158 1.75379047 0.753349628 0.628097886 1 0.645919359 1

DB02052 1.1666667 1.450138191 1.770629523 0.307115021 0.024616471 1 0.181824474 1

DB02054 2 0.865227879 1.75379047 0.753349628 0.628097886 1 0.645919359 1

DB02056 1 1.573210353 1.745287633 0.542671379 0.08481895 1 0.12201228 1

DB02057 2 1.35080904 1.75379047 0.753349628 0.628097886 1 0.645919359 1

DB02058 1 2.114015591 1.745287633 0.542671379 0.08481895 1 0.12201228 1

DB02059 1.25 1.9207191 1.753692879 0.367879736 0.085471804 1 0.217648936 1

DB02062 1.5 2.263643515 1.745287633 0.542671379 0.325634379 1 0.346299761 1

DB02063 2 2.635244804 1.75379047 0.753349628 0.628097886 1 0.645919359 1

DB02067 2 2.07907788 1.75379047 0.753349628 0.628097886 1 0.645919359 1

DB02068 2 1.946298969 1.745287633 0.542671379 0.680596551 1 0.645919359 1

DB02069 2 2.014892322 1.75379047 0.753349628 0.628097886 1 0.645919359 1

DB02071 2 1.066525304 1.75379047 0.753349628 0.628097886 1 0.645919359 1

DB02072 1 0.403186726 1.75379047 0.753349628 0.1585137 1 0.12201228 1

DB02073 1 2.175151831 1.757244872 0.464542708 0.051541591 1 0.12201228 1

DB02076 2 2.361508209 1.75379047 0.753349628 0.628097886 1 0.645919359 1

DB02077 1 1.788331493 1.745287633 0.542671379 0.08481895 1 0.12201228 1

DB02078 1.3333333 1.138165357 1.757244872 0.464542708 0.180743525 1 0.257238652 1

DB02079 1 0.847605554 1.75379047 0.753349628 0.1585137 1 0.12201228 1

DB02080 1 0.900429409 1.75379047 0.753349628 0.1585137 1 0.12201228 1

DB02081 2 1.204491569 1.75379047 0.753349628 0.628097886 1 0.645919359 1

DB02082 2 2.496528592 1.75379047 0.753349628 0.628097886 1 0.645919359 1

DB02084 2 2.970111612 1.75379047 0.753349628 0.628097886 1 0.645919359 1

DB02087 2 0.471119483 1.75379047 0.753349628 0.628097886 1 0.645919359 1

DB02090 2 2.078113656 1.75379047 0.753349628 0.628097886 1 0.645919359 1

DB02091 1 1.604186299 1.745287633 0.542671379 0.08481895 1 0.12201228 1

DB02095 1 2.822192027 1.75379047 0.753349628 0.1585137 1 0.12201228 1

DB02096 1 2.043585742 1.75379047 0.753349628 0.1585137 1 0.12201228 1

DB02097 2 1.473306353 1.75379047 0.753349628 0.628097886 1 0.645919359 1

DB02098 1.6666667 2.267977611 1.757244872 0.464542708 0.422702925 1 0.444812957 1

DB02101 2 1.77021366 1.75379047 0.753349628 0.628097886 1 0.645919359 1

DB02103 1 1.232856239 1.75379047 0.753349628 0.1585137 1 0.12201228 1

DB02104 2 1.189425846 1.75379047 0.753349628 0.628097886 1 0.645919359 1

DB02105 1 3.032863757 1.75379047 0.753349628 0.1585137 1 0.12201228 1

DB02106 1 2.298633253 1.75379047 0.753349628 0.1585137 1 0.12201228 1

DB02107 2 1.06684844 1.75379047 0.753349628 0.628097886 1 0.645919359 1

DB02108 1 0.353093874 1.75379047 0.753349628 0.1585137 1 0.12201228 1

DB02109 2 2.909135137 1.75379047 0.753349628 0.628097886 1 0.645919359 1

DB02110 1.5 2.132578263 1.745287633 0.542671379 0.325634379 1 0.346299761 1

DB02112 2 1.712261378 1.75379047 0.753349628 0.628097886 1 0.645919359 1

DB02114 2 1.743772206 1.75379047 0.753349628 0.628097886 1 0.645919359 1

DB02115 2 0.663621857 1.75379047 0.753349628 0.628097886 1 0.645919359 1

DB02116 1.25 2.092838985 1.753692879 0.367879736 0.085471804 1 0.217648936 1

DB02118 1 2.058425745 1.75379047 0.753349628 0.1585137 1 0.12201228 1

DB02126 2 0.921249831 1.745287633 0.542671379 0.680596551 1 0.645919359 1

DB02131 2 1.378716882 1.75379047 0.753349628 0.628097886 1 0.645919359 1

DB02132 2 1.468324356 1.75379047 0.753349628 0.628097886 1 0.645919359 1

DB02140 2 2.32622656 1.75379047 0.753349628 0.628097886 1 0.645919359 1

DB02141 1 1.258478413 1.75379047 0.753349628 0.1585137 1 0.12201228 1

DB02142 2 1.628408569 1.75379047 0.753349628 0.628097886 1 0.645919359 1

DB02143 1 1.269840139 1.75379047 0.753349628 0.1585137 1 0.12201228 1

DB02144 2 2.706571656 1.75379047 0.753349628 0.628097886 1 0.645919359 1

DB02148 1 1.12723534 1.75379047 0.753349628 0.1585137 1 0.12201228 1

DB02152 1 1.867017624 1.745287633 0.542671379 0.08481895 1 0.12201228 1

DB02153 2 1.829282779 1.757244872 0.464542708 0.699362524 1 0.645919359 1

DB02155 1 2.174212955 1.75379047 0.753349628 0.1585137 1 0.12201228 1

DB02159 1 1.62179088 1.75379047 0.753349628 0.1585137 1 0.12201228 1

DB02161 2 1.974199764 1.75379047 0.753349628 0.628097886 1 0.645919359 1

DB02162 1 1.865423708 1.75379047 0.753349628 0.1585137 1 0.12201228 1

DB02164 2 1.749822156 1.75379047 0.753349628 0.628097886 1 0.645919359 1

DB02165 1 0.873083091 1.75379047 0.753349628 0.1585137 1 0.12201228 1

DB02166 2 2.97562244 1.75379047 0.753349628 0.628097886 1 0.645919359 1

DB02169 1 0.124721276 1.75379047 0.753349628 0.1585137 1 0.12201228 1

DB02170 2 2.781421918 1.75379047 0.753349628 0.628097886 1 0.645919359 1

DB02175 1.5 1.367608645 1.745287633 0.542671379 0.325634379 1 0.346299761 1

DB02177 1 1.303737556 1.75379047 0.753349628 0.1585137 1 0.12201228 1

DB02180 2 1.314644971 1.75379047 0.753349628 0.628097886 1 0.645919359 1

DB02181 1 -0.275047578 1.75379047 0.753349628 0.1585137 1 0.12201228 1

DB02183 1 1.522008603 1.75379047 0.753349628 0.1585137 1 0.12201228 1

DB02185 2 0.540148957 1.75379047 0.753349628 0.628097886 1 0.645919359 1

DB02187 1 2.24618483 1.745287633 0.542671379 0.08481895 1 0.12201228 1

DB02189 1 1.33698362 1.75379047 0.753349628 0.1585137 1 0.12201228 1

DB02195 1 1.865679177 1.75379047 0.753349628 0.1585137 1 0.12201228 1

DB02196 2 1.765963608 1.757244872 0.464542708 0.699362524 1 0.645919359 1

DB02197 1 2.128141004 1.75379047 0.753349628 0.1585137 1 0.12201228 1

DB02198 2 1.936446573 1.75379047 0.753349628 0.628097886 1 0.645919359 1

DB02201 2 1.460110057 1.75379047 0.753349628 0.628097886 1 0.645919359 1

DB02205 2 3.570835235 1.75379047 0.753349628 0.628097886 1 0.645919359 1

DB02207 1 1.996437821 1.745287633 0.542671379 0.08481895 1 0.12201228 1

DB02209 1 2.480535131 1.75379047 0.753349628 0.1585137 1 0.12201228 1

DB02210 1 1.929284577 1.757244872 0.464542708 0.051541591 1 0.12201228 1

DB02211 1 1.155395406 1.75379047 0.753349628 0.1585137 1 0.12201228 1

DB02215 1 2.396900334 1.75379047 0.753349628 0.1585137 1 0.12201228 1

DB02216 1 2.577750212 1.75379047 0.753349628 0.1585137 1 0.12201228 1

DB02217 2 2.761797321 1.75379047 0.753349628 0.628097886 1 0.645919359 1

DB02218 2 1.378339873 1.745287633 0.542671379 0.680596551 1 0.645919359 1

DB02220 2 1.786315453 1.75379047 0.753349628 0.628097886 1 0.645919359 1

DB02221 2 2.181125342 1.75379047 0.753349628 0.628097886 1 0.645919359 1

DB02222 2 1.547159378 1.75379047 0.753349628 0.628097886 1 0.645919359 1

DB02226 2 0.613112552 1.75379047 0.753349628 0.628097886 1 0.645919359 1

DB02230 2 1.413176195 1.75379047 0.753349628 0.628097886 1 0.645919359 1

DB02234 1 0.83073178 1.745287633 0.542671379 0.08481895 1 0.12201228 1

DB02235 1 2.287854929 1.75379047 0.753349628 0.1585137 1 0.12201228 1

DB02236 1 1.33065649 1.75379047 0.753349628 0.1585137 1 0.12201228 1

DB02239 2 2.088075312 1.75379047 0.753349628 0.628097886 1 0.645919359 1

DB02243 2 0.547978628 1.75379047 0.753349628 0.628097886 1 0.645919359 1

DB02249 2 1.974618436 1.75379047 0.753349628 0.628097886 1 0.645919359 1

DB02255 1 0.909088575 1.745287633 0.542671379 0.08481895 1 0.12201228 1

DB02258 2 2.653335128 1.75379047 0.753349628 0.628097886 1 0.645919359 1

DB02259 1 1.383366626 1.75379047 0.753349628 0.1585137 1 0.12201228 1

DB02260 2 2.087237629 1.75379047 0.753349628 0.628097886 1 0.645919359 1

DB02261 1.5 1.638206468 1.745287633 0.542671379 0.325634379 1 0.346299761 1

DB02262 2 2.098175983 1.75379047 0.753349628 0.628097886 1 0.645919359 1

DB02264 1.6666667 2.511728359 1.757244872 0.464542708 0.422702925 1 0.444812957 1

DB02266 1.1666667 1.716124857 1.770629523 0.307115021 0.024616471 1 0.181824474 1

DB02267 2 1.980984194 1.75379047 0.753349628 0.628097886 1 0.645919359 1

DB02269 2 1.823110379 1.75379047 0.753349628 0.628097886 1 0.645919359 1

DB02271 2 2.258781457 1.75379047 0.753349628 0.628097886 1 0.645919359 1

DB02272 2 1.304466014 1.75379047 0.753349628 0.628097886 1 0.645919359 1

DB02277 1 2.112582996 1.75379047 0.753349628 0.1585137 1 0.12201228 1

DB02279 2 2.485321245 1.75379047 0.753349628 0.628097886 1 0.645919359 1

DB02285 2 2.050015423 1.75379047 0.753349628 0.628097886 1 0.645919359 1

DB02287 1.3333333 1.887224837 1.757244872 0.464542708 0.180743525 1 0.257238652 1

DB02288 2 2.060936105 1.75379047 0.753349628 0.628097886 1 0.645919359 1

DB02292 2 1.346365089 1.745287633 0.542671379 0.680596551 1 0.645919359 1

DB02296 2 1.982664958 1.75379047 0.753349628 0.628097886 1 0.645919359 1

DB02297 1 1.746539279 1.75379047 0.753349628 0.1585137 1 0.12201228 1

DB02299 2 1.320713648 1.75379047 0.753349628 0.628097886 1 0.645919359 1

DB02300 2 0.683069212 1.75379047 0.753349628 0.628097886 1 0.645919359 1

DB02303 2 3.017607366 1.75379047 0.753349628 0.628097886 1 0.645919359 1

DB02306 2 2.618234423 1.75379047 0.753349628 0.628097886 1 0.645919359 1

DB02308 2 1.255079631 1.75379047 0.753349628 0.628097886 1 0.645919359 1

DB02309 1 2.085829259 1.745287633 0.542671379 0.08481895 1 0.12201228 1

DB02310 2 2.529048318 1.75379047 0.753349628 0.628097886 1 0.645919359 1

DB02311 2 1.19458978 1.75379047 0.753349628 0.628097886 1 0.645919359 1

DB02315 1.3333333 2.293964679 1.757244872 0.464542708 0.180743525 1 0.257238652 1

DB02318 2 2.774961859 1.75379047 0.753349628 0.628097886 1 0.645919359 1

DB02320 2 1.890179284 1.75379047 0.753349628 0.628097886 1 0.645919359 1

DB02322 1 2.043411609 1.75379047 0.753349628 0.1585137 1 0.12201228 1

DB02323 1 1.68892634 1.75379047 0.753349628 0.1585137 1 0.12201228 1

DB02326 1 1.84106807 1.75379047 0.753349628 0.1585137 1 0.12201228 1

DB02328 2 1.918842053 1.75379047 0.753349628 0.628097886 1 0.645919359 1

DB02329 2 2.132896779 1.75379047 0.753349628 0.628097886 1 0.645919359 1

DB02335 1 1.878959122 1.75379047 0.753349628 0.1585137 1 0.12201228 1

DB02336 1 2.117517151 1.75379047 0.753349628 0.1585137 1 0.12201228 1

DB02337 2 1.134283882 1.75379047 0.753349628 0.628097886 1 0.645919359 1

DB02338 1.6666667 1.903470366 1.757244872 0.464542708 0.422702925 1 0.444812957 1

DB02340 1 2.096141562 1.75379047 0.753349628 0.1585137 1 0.12201228 1

DB02341 1.5 1.288427988 1.745287633 0.542671379 0.325634379 1 0.346299761 1

DB02342 -0.1854507 1.786710695 1.757979426 0.337271213 4.15E-09 2.26E-05 0.001395957 1

DB02343 2 0.800893334 1.75379047 0.753349628 0.628097886 1 0.645919359 1

DB02345 1 1.610225875 1.75379047 0.753349628 0.1585137 1 0.12201228 1

DB02347 2 1.413091907 1.75379047 0.753349628 0.628097886 1 0.645919359 1

DB02348 2 3.100632729 1.75379047 0.753349628 0.628097886 1 0.645919359 1

DB02350 2 -0.276665042 1.75379047 0.753349628 0.628097886 1 0.645919359 1

DB02352 1.5 1.976025964 1.745287633 0.542671379 0.325634379 1 0.346299761 1

DB02353 1 1.509005018 1.75379047 0.753349628 0.1585137 1 0.12201228 1

DB02354 2 1.649742646 1.75379047 0.753349628 0.628097886 1 0.645919359 1

DB02358 2 0.329376173 1.75379047 0.753349628 0.628097886 1 0.645919359 1

DB02359 1 1.866523133 1.75379047 0.753349628 0.1585137 1 0.12201228 1

DB02360 1 1.492434637 1.75379047 0.753349628 0.1585137 1 0.12201228 1

DB02363 2 1.37255818 1.75379047 0.753349628 0.628097886 1 0.645919359 1

DB02366 2 1.102707835 1.75379047 0.753349628 0.628097886 1 0.645919359 1

DB02367 2 2.643557976 1.75379047 0.753349628 0.628097886 1 0.645919359 1

DB02371 2 1.906142919 1.75379047 0.753349628 0.628097886 1 0.645919359 1

DB02373 2 2.197961302 1.75379047 0.753349628 0.628097886 1 0.645919359 1

DB02375 1 3.212287067 1.745287633 0.542671379 0.08481895 1 0.12201228 1

DB02376 2 1.984574895 1.75379047 0.753349628 0.628097886 1 0.645919359 1

DB02377 1.5 1.636834411 1.745287633 0.542671379 0.325634379 1 0.346299761 1

DB02378 1 1.322378145 1.75379047 0.753349628 0.1585137 1 0.12201228 1

DB02379 1.7142857 1.931350077 1.763665626 0.163272037 0.381158495 1 0.473897096 1

DB02381 1 2.119912306 1.75379047 0.753349628 0.1585137 1 0.12201228 1

DB02383 2 2.089817702 1.757244872 0.464542708 0.699362524 1 0.645919359 1

DB02386 2 1.688257331 1.75379047 0.753349628 0.628097886 1 0.645919359 1

DB02391 2 2.662725584 1.75379047 0.753349628 0.628097886 1 0.645919359 1

DB02393 2 2.293753996 1.75379047 0.753349628 0.628097886 1 0.645919359 1

DB02395 1 2.545119629 1.75379047 0.753349628 0.1585137 1 0.12201228 1

DB02396 1 2.529625609 1.75379047 0.753349628 0.1585137 1 0.12201228 1

DB02398 1 2.257241677 1.75379047 0.753349628 0.1585137 1 0.12201228 1

DB02400 1 1.46249641 1.75379047 0.753349628 0.1585137 1 0.12201228 1

DB02401 2 1.855394633 1.75379047 0.753349628 0.628097886 1 0.645919359 1

DB02402 2 0.86580089 1.75379047 0.753349628 0.628097886 1 0.645919359 1

DB02404 2 2.711487584 1.75379047 0.753349628 0.628097886 1 0.645919359 1

DB02405 1 1.353065848 1.75379047 0.753349628 0.1585137 1 0.12201228 1

DB02407 1 1.902013674 1.745287633 0.542671379 0.08481895 1 0.12201228 1

DB02418 1 2.271318309 1.75379047 0.753349628 0.1585137 1 0.12201228 1

DB02419 1 0.780353458 1.75379047 0.753349628 0.1585137 1 0.12201228 1

DB02420 1 1.640812911 1.75379047 0.753349628 0.1585137 1 0.12201228 1

DB02422 2 1.792189814 1.75379047 0.753349628 0.628097886 1 0.645919359 1

DB02424 1 1.815120477 1.757244872 0.464542708 0.051541591 1 0.12201228 1

DB02426 2 1.366143692 1.75379047 0.753349628 0.628097886 1 0.645919359 1

DB02427 2 2.190316249 1.75379047 0.753349628 0.628097886 1 0.645919359 1

DB02429 2 1.505576573 1.75379047 0.753349628 0.628097886 1 0.645919359 1

DB02431 2 2.168233018 1.75379047 0.753349628 0.628097886 1 0.645919359 1

DB02432 1 1.840325239 1.75379047 0.753349628 0.1585137 1 0.12201228 1

DB02435 2 2.09947973 1.75379047 0.753349628 0.628097886 1 0.645919359 1

DB02436 1 1.001597757 1.75379047 0.753349628 0.1585137 1 0.12201228 1

DB02445 2 1.735835139 1.75379047 0.753349628 0.628097886 1 0.645919359 1

DB02447 2 1.006886455 1.75379047 0.753349628 0.628097886 1 0.645919359 1

DB02448 1 1.843610793 1.757244872 0.464542708 0.051541591 1 0.12201228 1

DB02449 2 1.528898829 1.75379047 0.753349628 0.628097886 1 0.645919359 1

DB02451 1.4 1.64204887 1.757979426 0.337271213 0.14425424 1 0.291413148 1

DB02452 1.5 1.164970084 1.745287633 0.542671379 0.325634379 1 0.346299761 1

DB02457 2 2.231147674 1.75379047 0.753349628 0.628097886 1 0.645919359 1

DB02458 1 1.489816372 1.745287633 0.542671379 0.08481895 1 0.12201228 1

DB02459 2 2.139403766 1.75379047 0.753349628 0.628097886 1 0.645919359 1

DB02462 1 0.935053121 1.75379047 0.753349628 0.1585137 1 0.12201228 1

DB02463 2 1.247613624 1.75379047 0.753349628 0.628097886 1 0.645919359 1

DB02464 2 1.581757177 1.75379047 0.753349628 0.628097886 1 0.645919359 1

DB02465 1 2.120062491 1.75379047 0.753349628 0.1585137 1 0.12201228 1

DB02466 2 1.097280871 1.75379047 0.753349628 0.628097886 1 0.645919359 1

DB02467 2 0.952083324 1.75379047 0.753349628 0.628097886 1 0.645919359 1

DB02468 1 1.728024139 1.75379047 0.753349628 0.1585137 1 0.12201228 1

DB02471 2 1.443900384 1.757244872 0.464542708 0.699362524 1 0.645919359 1

DB02472 1 -0.01029528 1.75379047 0.753349628 0.1585137 1 0.12201228 1

DB02473 1 1.308651868 1.75379047 0.753349628 0.1585137 1 0.12201228 1

DB02479 2 2.758674533 1.75379047 0.753349628 0.628097886 1 0.645919359 1

DB02481 2 1.116462727 1.75379047 0.753349628 0.628097886 1 0.645919359 1

DB02482 1.1111111 1.946378274 1.753207263 0.264393406 0.007579386 1 0.160123241 1

DB02483 1.5 1.85832075 1.745287633 0.542671379 0.325634379 1 0.346299761 1

DB02485 2 1.663581337 1.75379047 0.753349628 0.628097886 1 0.645919359 1

DB02486 1 1.222177073 1.75379047 0.753349628 0.1585137 1 0.12201228 1

DB02490 2 2.115837678 1.75379047 0.753349628 0.628097886 1 0.645919359 1

DB02491 1 0.956926604 1.75379047 0.753349628 0.1585137 1 0.12201228 1

DB02492 2 2.177060567 1.75379047 0.753349628 0.628097886 1 0.645919359 1

DB02494 2 1.558741678 1.773663076 0.187305714 0.886549892 1 0.645919359 1

DB02497 1 2.169877792 1.75379047 0.753349628 0.1585137 1 0.12201228 1

DB02498 2 1.259644288 1.75379047 0.753349628 0.628097886 1 0.645919359 1

DB02499 1 0.751483464 1.75379047 0.753349628 0.1585137 1 0.12201228 1

DB02504 1 0.689863785 1.75379047 0.753349628 0.1585137 1 0.12201228 1

DB02506 1.2 1.908510954 1.757979426 0.337271213 0.049023756 1 0.195688273 1

DB02508 2 2.173860488 1.75379047 0.753349628 0.628097886 1 0.645919359 1

DB02509 1.5 1.864437499 1.745287633 0.542671379 0.325634379 1 0.346299761 1

DB02510 2 1.919742164 1.75379047 0.753349628 0.628097886 1 0.645919359 1

DB02515 2 1.043026022 1.75379047 0.753349628 0.628097886 1 0.645919359 1

DB02518 2 2.138514616 1.75379047 0.753349628 0.628097886 1 0.645919359 1

DB02519 2 0.996752818 1.75379047 0.753349628 0.628097886 1 0.645919359 1

DB02526 1.5 1.990190667 1.745287633 0.542671379 0.325634379 1 0.346299761 1

DB02527 1.6666667 1.428480795 1.757244872 0.464542708 0.422702925 1 0.444812957 1

DB02528 1 2.633632024 1.75379047 0.753349628 0.1585137 1 0.12201228 1

DB02530 1.3333333 1.582947538 1.757244872 0.464542708 0.180743525 1 0.257238652 1

DB02535 2 1.912950023 1.75379047 0.753349628 0.628097886 1 0.645919359 1

DB02538 1 1.89244837 1.75379047 0.753349628 0.1585137 1 0.12201228 1

DB02541 2 1.039379407 1.75379047 0.753349628 0.628097886 1 0.645919359 1

DB02542 1 2.234746713 1.75379047 0.753349628 0.1585137 1 0.12201228 1

DB02545 2 2.374671486 1.75379047 0.753349628 0.628097886 1 0.645919359 1

DB02546 1.6 1.700587311 1.757979426 0.337271213 0.319747599 1 0.404623142 1

DB02547 1 0.415808633 1.75379047 0.753349628 0.1585137 1 0.12201228 1

DB02550 1 1.556636927 1.75379047 0.753349628 0.1585137 1 0.12201228 1

DB02551 1 2.290347361 1.75379047 0.753349628 0.1585137 1 0.12201228 1

DB02552 2 1.683109346 1.745287633 0.542671379 0.680596551 1 0.645919359 1

DB02553 2 2.216279111 1.75379047 0.753349628 0.628097886 1 0.645919359 1

DB02555 1 2.047920746 1.75379047 0.753349628 0.1585137 1 0.12201228 1

DB02556 2 2.121726778 1.75379047 0.753349628 0.628097886 1 0.645919359 1

DB02557 1.5 2.07315667 1.745287633 0.542671379 0.325634379 1 0.346299761 1

DB02558 1 1.598059662 1.75379047 0.753349628 0.1585137 1 0.12201228 1

DB02559 2 0.750534093 1.75379047 0.753349628 0.628097886 1 0.645919359 1

DB02562 2 1.987116978 1.75379047 0.753349628 0.628097886 1 0.645919359 1

DB02563 2 2.95450266 1.75379047 0.753349628 0.628097886 1 0.645919359 1

DB02565 2 2.029435933 1.75379047 0.753349628 0.628097886 1 0.645919359 1

DB02568 2 1.9384181 1.75379047 0.753349628 0.628097886 1 0.645919359 1

DB02569 1 2.289925406 1.75379047 0.753349628 0.1585137 1 0.12201228 1

DB02570 1 1.809653045 1.75379047 0.753349628 0.1585137 1 0.12201228 1

DB02573 2 1.261390916 1.75379047 0.753349628 0.628097886 1 0.645919359 1

DB02580 1 1.98132208 1.75379047 0.753349628 0.1585137 1 0.12201228 1

DB02581 1 2.988080005 1.75379047 0.753349628 0.1585137 1 0.12201228 1

DB02583 2 1.513511606 1.75379047 0.753349628 0.628097886 1 0.645919359 1

DB02585 2 0.865114776 1.75379047 0.753349628 0.628097886 1 0.645919359 1

DB02587 1 1.623286212 1.757244872 0.464542708 0.051541591 1 0.12201228 1

DB02589 1 2.95754034 1.75379047 0.753349628 0.1585137 1 0.12201228 1

DB02594 2 0.981738734 1.745287633 0.542671379 0.680596551 1 0.645919359 1

DB02596 2 1.287166796 1.75379047 0.753349628 0.628097886 1 0.645919359 1

DB02597 1 1.193538745 1.75379047 0.753349628 0.1585137 1 0.12201228 1

DB02598 2 1.723593736 1.75379047 0.753349628 0.628097886 1 0.645919359 1

DB02602 2 1.222521946 1.75379047 0.753349628 0.628097886 1 0.645919359 1

DB02603 1 1.795723718 1.75379047 0.753349628 0.1585137 1 0.12201228 1

DB02604 2 1.496971054 1.75379047 0.753349628 0.628097886 1 0.645919359 1

DB02607 1 2.815669847 1.75379047 0.753349628 0.1585137 1 0.12201228 1

DB02610 2 1.810080392 1.75379047 0.753349628 0.628097886 1 0.645919359 1

DB02611 1 1.254579786 1.75379047 0.753349628 0.1585137 1 0.12201228 1

DB02613 2 0.656361154 1.75379047 0.753349628 0.628097886 1 0.645919359 1

DB02615 1 2.387679145 1.745287633 0.542671379 0.08481895 1 0.12201228 1

DB02616 1 2.683597499 1.75379047 0.753349628 0.1585137 1 0.12201228 1

DB02620 1 1.554667403 1.75379047 0.753349628 0.1585137 1 0.12201228 1

DB02621 1 2.007850968 1.745287633 0.542671379 0.08481895 1 0.12201228 1

DB02622 1 1.859699068 1.75379047 0.753349628 0.1585137 1 0.12201228 1

DB02623 1 0.902144847 1.75379047 0.753349628 0.1585137 1 0.12201228 1

DB02624 2 2.32821588 1.75379047 0.753349628 0.628097886 1 0.645919359 1

DB02628 1 2.109931299 1.75379047 0.753349628 0.1585137 1 0.12201228 1

DB02633 1 1.886255727 1.745287633 0.542671379 0.08481895 1 0.12201228 1

DB02635 2 1.772768568 1.75379047 0.753349628 0.628097886 1 0.645919359 1

DB02636 1 2.15210655 1.75379047 0.753349628 0.1585137 1 0.12201228 1

DB02637 1.5 1.497180553 1.745287633 0.542671379 0.325634379 1 0.346299761 1

DB02638 1 1.932259548 1.745287633 0.542671379 0.08481895 1 0.12201228 1

DB02640 2 0.974088559 1.75379047 0.753349628 0.628097886 1 0.645919359 1

DB02643 1 1.321795473 1.745287633 0.542671379 0.08481895 1 0.12201228 1

DB02644 1 1.855506461 1.745287633 0.542671379 0.08481895 1 0.12201228 1

DB02646 1 2.061243708 1.75379047 0.753349628 0.1585137 1 0.12201228 1

DB02647 1 2.562549795 1.75379047 0.753349628 0.1585137 1 0.12201228 1

DB02648 2 1.841637691 1.745287633 0.542671379 0.680596551 1 0.645919359 1

DB02651 1 0.847508557 1.75379047 0.753349628 0.1585137 1 0.12201228 1

DB02652 2 1.367608633 1.75379047 0.753349628 0.628097886 1 0.645919359 1

DB02656 1 0.47450467 1.745287633 0.542671379 0.08481895 1 0.12201228 1

DB02659 1.6666667 1.779406306 1.755399642 0.180590294 0.311589654 1 0.444812957 1

DB02660 1 3.175262059 1.75379047 0.753349628 0.1585137 1 0.12201228 1

DB02661 1 2.098229951 1.75379047 0.753349628 0.1585137 1 0.12201228 1

DB02662 1 2.560199633 1.75379047 0.753349628 0.1585137 1 0.12201228 1

DB02665 2 3.102477015 1.75379047 0.753349628 0.628097886 1 0.645919359 1

DB02670 2 3.151945224 1.75379047 0.753349628 0.628097886 1 0.645919359 1

DB02671 1 2.468404293 1.75379047 0.753349628 0.1585137 1 0.12201228 1

DB02673 2 1.850019488 1.75379047 0.753349628 0.628097886 1 0.645919359 1

DB02676 1 1.345349487 1.75379047 0.753349628 0.1585137 1 0.12201228 1

DB02678 1 1.841871488 1.75379047 0.753349628 0.1585137 1 0.12201228 1

DB02679 2 0.784501894 1.745287633 0.542671379 0.680596551 1 0.645919359 1

DB02681 1 1.478602285 1.75379047 0.753349628 0.1585137 1 0.12201228 1

DB02684 2 2.023659233 1.75379047 0.753349628 0.628097886 1 0.645919359 1

DB02685 1 2.025819215 1.75379047 0.753349628 0.1585137 1 0.12201228 1

DB02689 1 2.055220935 1.75379047 0.753349628 0.1585137 1 0.12201228 1

DB02690 2 1.166441594 1.75379047 0.753349628 0.628097886 1 0.645919359 1

DB02691 2 2.662309054 1.75379047 0.753349628 0.628097886 1 0.645919359 1

DB02696 1 1.085717918 1.75379047 0.753349628 0.1585137 1 0.12201228 1

DB02697 2 2.584105616 1.745287633 0.542671379 0.680596551 1 0.645919359 1

DB02701 1.5 2.032968995 1.753692879 0.367879736 0.245220308 1 0.346299761 1

DB02705 1 1.893212478 1.75379047 0.753349628 0.1585137 1 0.12201228 1

DB02709 1.3461538 1.917378566 1.752565715 0.14361026 0.00232766 1 0.263645339 1

DB02710 1 0.427705545 1.75379047 0.753349628 0.1585137 1 0.12201228 1

DB02712 2 1.959315735 1.75379047 0.753349628 0.628097886 1 0.645919359 1

DB02714 2 1.989859228 1.75379047 0.753349628 0.628097886 1 0.645919359 1

DB02715 1 1.401423797 1.75379047 0.753349628 0.1585137 1 0.12201228 1

DB02716 1 1.082897027 1.75379047 0.753349628 0.1585137 1 0.12201228 1

DB02719 2 1.447071134 1.75379047 0.753349628 0.628097886 1 0.645919359 1

DB02720 2 3.471543668 1.75379047 0.753349628 0.628097886 1 0.645919359 1

DB02721 2 1.272999937 1.757244872 0.464542708 0.699362524 1 0.645919359 1

DB02723 1 1.713734583 1.75379047 0.753349628 0.1585137 1 0.12201228 1

DB02726 1.2 1.936390721 1.757979426 0.337271213 0.049023756 1 0.195688273 1

DB02727 1 2.884353284 1.75379047 0.753349628 0.1585137 1 0.12201228 1

DB02728 2 1.556820319 1.75379047 0.753349628 0.628097886 1 0.645919359 1

DB02730 2 0.100597455 1.75379047 0.753349628 0.628097886 1 0.645919359 1

DB02731 2 1.518490632 1.75379047 0.753349628 0.628097886 1 0.645919359 1

DB02733 1.2 1.967514074 1.757979426 0.337271213 0.049023756 1 0.195688273 1

DB02735 2 2.59548298 1.75379047 0.753349628 0.628097886 1 0.645919359 1

DB02740 1 1.840885796 1.75379047 0.753349628 0.1585137 1 0.12201228 1

DB02741 2 2.868853269 1.75379047 0.753349628 0.628097886 1 0.645919359 1

DB02742 2 1.592079665 1.745287633 0.542671379 0.680596551 1 0.645919359 1

DB02744 2 1.360172298 1.75379047 0.753349628 0.628097886 1 0.645919359 1

DB02745 2 1.558815536 1.75379047 0.753349628 0.628097886 1 0.645919359 1

DB02746 1.2222222 1.623962031 1.753207263 0.264393406 0.022304951 1 0.205277671 1

DB02750 2 1.099234927 1.75379047 0.753349628 0.628097886 1 0.645919359 1

DB02751 2 1.37013591 1.75379047 0.753349628 0.628097886 1 0.645919359 1

DB02754 1 1.522506733 1.745287633 0.542671379 0.08481895 1 0.12201228 1

DB02755 1 1.622522621 1.75379047 0.753349628 0.1585137 1 0.12201228 1

DB02757 1.3333333 1.930904297 1.757244872 0.464542708 0.180743525 1 0.257238652 1

DB02758 1 1.852305742 1.75379047 0.753349628 0.1585137 1 0.12201228 1

DB02759 1 1.636728183 1.75379047 0.753349628 0.1585137 1 0.12201228 1

DB02761 2 1.906346685 1.75379047 0.753349628 0.628097886 1 0.645919359 1

DB02762 1 1.065029277 1.75379047 0.753349628 0.1585137 1 0.12201228 1

DB02763 1 1.262977329 1.75379047 0.753349628 0.1585137 1 0.12201228 1

DB02767 1 2.201926564 1.745287633 0.542671379 0.08481895 1 0.12201228 1

DB02772 1.4 1.325691583 1.757979426 0.337271213 0.14425424 1 0.291413148 1

DB02773 2 2.899191429 1.75379047 0.753349628 0.628097886 1 0.645919359 1

DB02774 1 2.303420006 1.75379047 0.753349628 0.1585137 1 0.12201228 1

DB02778 1 1.440262925 1.75379047 0.753349628 0.1585137 1 0.12201228 1

DB02779 1 1.647107761 1.75379047 0.753349628 0.1585137 1 0.12201228 1

DB02783 1 1.260633204 1.75379047 0.753349628 0.1585137 1 0.12201228 1

DB02784 1 1.250777746 1.75379047 0.753349628 0.1585137 1 0.12201228 1

DB02789 1.5 1.744010753 1.745287633 0.542671379 0.325634379 1 0.346299761 1

DB02795 1 1.341924323 1.745287633 0.542671379 0.08481895 1 0.12201228 1

DB02796 2 2.094453952 1.75379047 0.753349628 0.628097886 1 0.645919359 1

DB02800 2 1.821380386 1.75379047 0.753349628 0.628097886 1 0.645919359 1

DB02803 2 1.102852087 1.75379047 0.753349628 0.628097886 1 0.645919359 1

DB02805 2 2.316804764 1.75379047 0.753349628 0.628097886 1 0.645919359 1

DB02811 2 2.720069963 1.75379047 0.753349628 0.628097886 1 0.645919359 1

DB02812 2 1.701476801 1.75379047 0.753349628 0.628097886 1 0.645919359 1

DB02813 1 1.465914616 1.75379047 0.753349628 0.1585137 1 0.12201228 1

DB02818 2 1.571004512 1.75379047 0.753349628 0.628097886 1 0.645919359 1

DB02820 2 3.501774142 1.75379047 0.753349628 0.628097886 1 0.645919359 1

DB02821 2 2.085701321 1.75379047 0.753349628 0.628097886 1 0.645919359 1

DB02822 2 2.047697124 1.75379047 0.753349628 0.628097886 1 0.645919359 1

DB02824 1.5 1.969725273 1.745287633 0.542671379 0.325634379 1 0.346299761 1

DB02825 2 2.067434262 1.75379047 0.753349628 0.628097886 1 0.645919359 1

DB02827 1 2.561436335 1.75379047 0.753349628 0.1585137 1 0.12201228 1

DB02830 1 0.971718598 1.75379047 0.753349628 0.1585137 1 0.12201228 1

DB02831 2 0.449986388 1.75379047 0.753349628 0.628097886 1 0.645919359 1

DB02833 1 2.72879848 1.745287633 0.542671379 0.08481895 1 0.12201228 1

DB02834 2 1.027781264 1.75379047 0.753349628 0.628097886 1 0.645919359 1

DB02835 2 2.394033868 1.75379047 0.753349628 0.628097886 1 0.645919359 1

DB02837 2 2.12103772 1.75379047 0.753349628 0.628097886 1 0.645919359 1

DB02838 2 0.241244929 1.75379047 0.753349628 0.628097886 1 0.645919359 1

DB02840 1 2.287760025 1.75379047 0.753349628 0.1585137 1 0.12201228 1

DB02843 2 1.695070756 1.75379047 0.753349628 0.628097886 1 0.645919359 1

DB02845 2 1.568903749 1.745287633 0.542671379 0.680596551 1 0.645919359 1

DB02846 1 1.328407162 1.75379047 0.753349628 0.1585137 1 0.12201228 1

DB02848 1 1.840212194 1.75379047 0.753349628 0.1585137 1 0.12201228 1

DB02852 1 3.064303661 1.75379047 0.753349628 0.1585137 1 0.12201228 1

DB02854 1.5 1.923298225 1.745287633 0.542671379 0.325634379 1 0.346299761 1

DB02855 1 1.435511136 1.75379047 0.753349628 0.1585137 1 0.12201228 1

DB02857 2 1.45155877 1.75379047 0.753349628 0.628097886 1 0.645919359 1

DB02859 2 0.644116491 1.75379047 0.753349628 0.628097886 1 0.645919359 1

DB02860 1 1.682911537 1.75379047 0.753349628 0.1585137 1 0.12201228 1

DB02861 2 2.161744412 1.75379047 0.753349628 0.628097886 1 0.645919359 1

DB02866 2 1.205895139 1.75379047 0.753349628 0.628097886 1 0.645919359 1

DB02868 1 0.567344319 1.75379047 0.753349628 0.1585137 1 0.12201228 1

DB02869 2 1.018774455 1.745287633 0.542671379 0.680596551 1 0.645919359 1

DB02871 2 1.728050605 1.75379047 0.753349628 0.628097886 1 0.645919359 1

DB02872 1 1.497862769 1.75379047 0.753349628 0.1585137 1 0.12201228 1

DB02873 1 0.914480742 1.75379047 0.753349628 0.1585137 1 0.12201228 1

DB02875 2 0.063691168 1.75379047 0.753349628 0.628097886 1 0.645919359 1

DB02877 2 1.776707316 1.75379047 0.753349628 0.628097886 1 0.645919359 1

DB02878 2 0.837704528 1.75379047 0.753349628 0.628097886 1 0.645919359 1

DB02887 1 1.818630663 1.75379047 0.753349628 0.1585137 1 0.12201228 1

DB02888 2 2.638902088 1.75379047 0.753349628 0.628097886 1 0.645919359 1

DB02889 2 2.041884162 1.75379047 0.753349628 0.628097886 1 0.645919359 1

DB02890 2 1.617303437 1.75379047 0.753349628 0.628097886 1 0.645919359 1

DB02893 2 2.633103084 1.75379047 0.753349628 0.628097886 1 0.645919359 1

DB02894 2 1.797298382 1.75379047 0.753349628 0.628097886 1 0.645919359 1

DB02895 2 1.255146519 1.75379047 0.753349628 0.628097886 1 0.645919359 1

DB02898 1 2.6029823 1.75379047 0.753349628 0.1585137 1 0.12201228 1

DB02900 1 2.014952045 1.745287633 0.542671379 0.08481895 1 0.12201228 1

DB02901 1.25 2.181712553 1.753692879 0.367879736 0.085471804 1 0.217648936 1

DB02902 1 1.541089215 1.75379047 0.753349628 0.1585137 1 0.12201228 1

DB02908 1 1.102106489 1.75379047 0.753349628 0.1585137 1 0.12201228 1

DB02910 2 2.549087199 1.745287633 0.542671379 0.680596551 1 0.645919359 1

DB02911 1 1.345590508 1.75379047 0.753349628 0.1585137 1 0.12201228 1

DB02915 1 1.161737936 1.745287633 0.542671379 0.08481895 1 0.12201228 1

DB02917 2 2.599872463 1.75379047 0.753349628 0.628097886 1 0.645919359 1

DB02918 1 1.802068854 1.75379047 0.753349628 0.1585137 1 0.12201228 1

DB02919 2 1.223124257 1.75379047 0.753349628 0.628097886 1 0.645919359 1

DB02924 2 2.092732084 1.75379047 0.753349628 0.628097886 1 0.645919359 1

DB02925 2 2.507642561 1.75379047 0.753349628 0.628097886 1 0.645919359 1

DB02929 1 1.267997883 1.75379047 0.753349628 0.1585137 1 0.12201228 1

DB02930 1.8 1.176573584 1.757979426 0.337271213 0.549575873 1 0.52649583 1

DB02931 1 1.158455379 1.75379047 0.753349628 0.1585137 1 0.12201228 1

DB02932 1 0.421764854 1.75379047 0.753349628 0.1585137 1 0.12201228 1

DB02935 1 1.67831509 1.75379047 0.753349628 0.1585137 1 0.12201228 1

DB02936 1 1.08493548 1.75379047 0.753349628 0.1585137 1 0.12201228 1

DB02938 1 1.62500925 1.75379047 0.753349628 0.1585137 1 0.12201228 1

DB02942 2 1.988464377 1.75379047 0.753349628 0.628097886 1 0.645919359 1

DB02943 1 1.128651632 1.75379047 0.753349628 0.1585137 1 0.12201228 1

DB02950 1.3333333 2.33828631 1.757244872 0.464542708 0.180743525 1 0.257238652 1

DB02952 1.5 1.752879602 1.745287633 0.542671379 0.325634379 1 0.346299761 1

DB02953 1 2.316824978 1.75379047 0.753349628 0.1585137 1 0.12201228 1

DB02955 1 0.731065189 1.757244872 0.464542708 0.051541591 1 0.12201228 1

DB02963 1 2.277727624 1.75379047 0.753349628 0.1585137 1 0.12201228 1

DB02964 2 2.385911352 1.75379047 0.753349628 0.628097886 1 0.645919359 1

DB02966 2 1.580014266 1.75379047 0.753349628 0.628097886 1 0.645919359 1

DB02967 2 0.623822394 1.75379047 0.753349628 0.628097886 1 0.645919359 1

DB02973 1 0.440773454 1.75379047 0.753349628 0.1585137 1 0.12201228 1

DB02974 1 2.136315429 1.75379047 0.753349628 0.1585137 1 0.12201228 1

DB02977 1 1.855365918 1.75379047 0.753349628 0.1585137 1 0.12201228 1

DB02979 1 -1.120957622 1.75379047 0.753349628 0.1585137 1 0.12201228 1

DB02983 1.5 1.066282782 1.745287633 0.542671379 0.325634379 1 0.346299761 1

DB02984 1 2.006299476 1.75379047 0.753349628 0.1585137 1 0.12201228 1

DB02986 2 1.525845865 1.75379047 0.753349628 0.628097886 1 0.645919359 1

DB02987 1.5 1.398748675 1.745287633 0.542671379 0.325634379 1 0.346299761 1

DB02988 2 0.763632116 1.75379047 0.753349628 0.628097886 1 0.645919359 1

DB02989 2 0.884072322 1.75379047 0.753349628 0.628097886 1 0.645919359 1

DB02991 1 1.941195267 1.75379047 0.753349628 0.1585137 1 0.12201228 1

DB02994 1.6666667 0.98225449 1.757244872 0.464542708 0.422702925 1 0.444812957 1

DB02996 1 2.550954259 1.75379047 0.753349628 0.1585137 1 0.12201228 1

DB02998 1.25 1.801997058 1.753692879 0.367879736 0.085471804 1 0.217648936 1

DB02999 1.5 1.580624386 1.745287633 0.542671379 0.325634379 1 0.346299761 1

DB03003 1 2.530037673 1.757244872 0.464542708 0.051541591 1 0.12201228 1

DB03005 2 0.929709588 1.75379047 0.753349628 0.628097886 1 0.645919359 1

DB03006 1 2.99365493 1.75379047 0.753349628 0.1585137 1 0.12201228 1

DB03008 2 1.924996143 1.75379047 0.753349628 0.628097886 1 0.645919359 1

DB03010 1.5454545 1.841687441 1.759747174 0.221112233 0.166233199 1 0.372453903 1

DB03012 2 1.562053082 1.75379047 0.753349628 0.628097886 1 0.645919359 1

DB03013 1 0.303247138 1.745287633 0.542671379 0.08481895 1 0.12201228 1

DB03015 1 0.403951482 1.75379047 0.753349628 0.1585137 1 0.12201228 1

DB03016 2 2.563592557 1.75379047 0.753349628 0.628097886 1 0.645919359 1

DB03017 1.3636364 2.060411933 1.759747174 0.221112233 0.036610823 1 0.272511088 1

DB03019 1 1.7091534 1.75379047 0.753349628 0.1585137 1 0.12201228 1

DB03020 2 0.908777396 1.75379047 0.753349628 0.628097886 1 0.645919359 1

DB03021 1 2.970717561 1.75379047 0.753349628 0.1585137 1 0.12201228 1

DB03023 -1.3561419 1.115847217 1.757244872 0.464542708 1.03E-11 5.61E-08 8.26E-07 0.00451601

DB03024 2 2.377969396 1.75379047 0.753349628 0.628097886 1 0.645919359 1

DB03026 1 1.661816252 1.75379047 0.753349628 0.1585137 1 0.12201228 1

DB03028 1 2.281955024 1.75379047 0.753349628 0.1585137 1 0.12201228 1

DB03032 1 1.337106715 1.75379047 0.753349628 0.1585137 1 0.12201228 1

DB03033 2 1.005985351 1.745287633 0.542671379 0.680596551 1 0.645919359 1

DB03035 2 2.364264011 1.75379047 0.753349628 0.628097886 1 0.645919359 1

DB03039 2 1.630929615 1.75379047 0.753349628 0.628097886 1 0.645919359 1

DB03040 1 2.012059514 1.75379047 0.753349628 0.1585137 1 0.12201228 1

DB03041 2 2.291323634 1.745287633 0.542671379 0.680596551 1 0.645919359 1

DB03044 1 1.685523375 1.75379047 0.753349628 0.1585137 1 0.12201228 1

DB03046 1 3.462758718 1.75379047 0.753349628 0.1585137 1 0.12201228 1

DB03048 2 2.704964594 1.75379047 0.753349628 0.628097886 1 0.645919359 1

DB03056 2 1.148349426 1.75379047 0.753349628 0.628097886 1 0.645919359 1

DB03058 1 1.472153291 1.75379047 0.753349628 0.1585137 1 0.12201228 1

DB03059 1.6666667 1.16757728 1.757244872 0.464542708 0.422702925 1 0.444812957 1

DB03060 2 2.603970521 1.75379047 0.753349628 0.628097886 1 0.645919359 1

DB03061 2 1.144602928 1.75379047 0.753349628 0.628097886 1 0.645919359 1

DB03062 1 0.871860471 1.75379047 0.753349628 0.1585137 1 0.12201228 1

DB03064 2 1.460711521 1.75379047 0.753349628 0.628097886 1 0.645919359 1

DB03065 1 1.503181005 1.75379047 0.753349628 0.1585137 1 0.12201228 1

DB03067 2 2.232022472 1.75379047 0.753349628 0.628097886 1 0.645919359 1

DB03070 2 0.876238257 1.75379047 0.753349628 0.628097886 1 0.645919359 1

DB03072 2 1.691245996 1.75379047 0.753349628 0.628097886 1 0.645919359 1

DB03073 2 2.317396062 1.75379047 0.753349628 0.628097886 1 0.645919359 1

DB03078 1 2.22279672 1.75379047 0.753349628 0.1585137 1 0.12201228 1

DB03081 2 0.891297576 1.75379047 0.753349628 0.628097886 1 0.645919359 1

DB03082 1 2.055671524 1.75379047 0.753349628 0.1585137 1 0.12201228 1

DB03083 2 1.601835377 1.75379047 0.753349628 0.628097886 1 0.645919359 1

DB03085 2 1.440364575 1.75379047 0.753349628 0.628097886 1 0.645919359 1

DB03086 2 1.984894809 1.75379047 0.753349628 0.628097886 1 0.645919359 1

DB03087 2 1.661705812 1.75379047 0.753349628 0.628097886 1 0.645919359 1

DB03088 1.5714286 1.674795301 1.736866356 0.292454388 0.285803159 1 0.387676968 1

DB03092 2 1.540847804 1.757244872 0.464542708 0.699362524 1 0.645919359 1

DB03093 1 0.805077948 1.75379047 0.753349628 0.1585137 1 0.12201228 1

DB03095 2 2.103944205 1.75379047 0.753349628 0.628097886 1 0.645919359 1

DB03096 1 1.29890094 1.75379047 0.753349628 0.1585137 1 0.12201228 1

DB03100 1 1.920019216 1.745287633 0.542671379 0.08481895 1 0.12201228 1

DB03101 2 1.041740008 1.75379047 0.753349628 0.628097886 1 0.645919359 1

DB03102 1 2.675802979 1.75379047 0.753349628 0.1585137 1 0.12201228 1

DB03103 1 2.475992641 1.75379047 0.753349628 0.1585137 1 0.12201228 1

DB03104 1 2.263158966 1.75379047 0.753349628 0.1585137 1 0.12201228 1

DB03106 1 1.71807313 1.745287633 0.542671379 0.08481895 1 0.12201228 1

DB03109 1 1.9421538 1.75379047 0.753349628 0.1585137 1 0.12201228 1

DB03110 1 2.719957546 1.75379047 0.753349628 0.1585137 1 0.12201228 1

DB03114 1 2.817778153 1.75379047 0.753349628 0.1585137 1 0.12201228 1

DB03115 1 2.792364847 1.75379047 0.753349628 0.1585137 1 0.12201228 1

DB03120 1.5 2.16207517 1.745287633 0.542671379 0.325634379 1 0.346299761 1

DB03121 1 1.876588512 1.75379047 0.753349628 0.1585137 1 0.12201228 1

DB03124 1 1.684500107 1.75379047 0.753349628 0.1585137 1 0.12201228 1

DB03125 2 0.438749747 1.75379047 0.753349628 0.628097886 1 0.645919359 1

DB03127 1.7777778 1.455539198 1.753207263 0.264393406 0.537021102 1 0.512866521 1

DB03128 1.3333333 1.674797712 1.770629523 0.307115021 0.07724 1 0.257238652 1

DB03129 2 1.855530675 1.75379047 0.753349628 0.628097886 1 0.645919359 1

DB03130 2 1.848401728 1.75379047 0.753349628 0.628097886 1 0.645919359 1

DB03132 1 2.002208885 1.75379047 0.753349628 0.1585137 1 0.12201228 1

DB03133 2 1.502010798 1.75379047 0.753349628 0.628097886 1 0.645919359 1

DB03135 1 0.981246448 1.75379047 0.753349628 0.1585137 1 0.12201228 1

DB03136 1.3333333 1.535843725 1.757244872 0.464542708 0.180743525 1 0.257238652 1

DB03137 1 2.132863821 1.75379047 0.753349628 0.1585137 1 0.12201228 1

DB03144 1 1.487062768 1.753692879 0.367879736 0.020243381 1 0.12201228 1

DB03147 1.6086957 1.552969721 1.750829326 0.165162414 0.194737778 1 0.409818034 1

DB03150 2 2.160914582 1.75379047 0.753349628 0.628097886 1 0.645919359 1

DB03152 1.5 1.666932329 1.745287633 0.542671379 0.325634379 1 0.346299761 1

DB03153 1 1.947227661 1.75379047 0.753349628 0.1585137 1 0.12201228 1

DB03154 1 2.323847593 1.75379047 0.753349628 0.1585137 1 0.12201228 1

DB03155 2 1.517567549 1.75379047 0.753349628 0.628097886 1 0.645919359 1

DB03158 1 0.422136442 1.75379047 0.753349628 0.1585137 1 0.12201228 1

DB03159 1.3333333 1.854638265 1.757244872 0.464542708 0.180743525 1 0.257238652 1

DB03169 2 2.722650111 1.75379047 0.753349628 0.628097886 1 0.645919359 1

DB03173 2 3.278880749 1.75379047 0.753349628 0.628097886 1 0.645919359 1

DB03175 1 1.351939989 1.75379047 0.753349628 0.1585137 1 0.12201228 1

DB03176 1 1.432478614 1.745287633 0.542671379 0.08481895 1 0.12201228 1

DB03179 2 2.314047352 1.75379047 0.753349628 0.628097886 1 0.645919359 1

DB03181 1 1.556015191 1.75379047 0.753349628 0.1585137 1 0.12201228 1

DB03182 1 1.787266625 1.75379047 0.753349628 0.1585137 1 0.12201228 1

DB03183 1 1.561973991 1.75379047 0.753349628 0.1585137 1 0.12201228 1

DB03185 1 3.700656178 1.75379047 0.753349628 0.1585137 1 0.12201228 1

DB03186 2 2.480865162 1.75379047 0.753349628 0.628097886 1 0.645919359 1

DB03187 2 1.025078619 1.75379047 0.753349628 0.628097886 1 0.645919359 1

DB03189 1 1.206094339 1.75379047 0.753349628 0.1585137 1 0.12201228 1

DB03191 2 1.192400069 1.75379047 0.753349628 0.628097886 1 0.645919359 1

DB03193 1 2.540750067 1.745287633 0.542671379 0.08481895 1 0.12201228 1

DB03194 2 1.639510719 1.75379047 0.753349628 0.628097886 1 0.645919359 1

DB03195 2 2.227790181 1.75379047 0.753349628 0.628097886 1 0.645919359 1

DB03201 2 2.05239131 1.75379047 0.753349628 0.628097886 1 0.645919359 1

DB03202 2 2.887599653 1.75379047 0.753349628 0.628097886 1 0.645919359 1

DB03203 2 2.587453538 1.75379047 0.753349628 0.628097886 1 0.645919359 1

DB03206 2 1.645800416 1.75379047 0.753349628 0.628097886 1 0.645919359 1

DB03207 1 1.042138846 1.75379047 0.753349628 0.1585137 1 0.12201228 1

DB03213 2 2.423555656 1.75379047 0.753349628 0.628097886 1 0.645919359 1

DB03214 1.5 2.320662176 1.745287633 0.542671379 0.325634379 1 0.346299761 1

DB03216 2 1.764385683 1.75379047 0.753349628 0.628097886 1 0.645919359 1

DB03217 1 3.169521213 1.75379047 0.753349628 0.1585137 1 0.12201228 1

DB03218 2 1.518233038 1.75379047 0.753349628 0.628097886 1 0.645919359 1

DB03220 1 1.734027931 1.75379047 0.753349628 0.1585137 1 0.12201228 1

DB03221 2 2.279109088 1.75379047 0.753349628 0.628097886 1 0.645919359 1

DB03222 2 1.702333353 1.75379047 0.753349628 0.628097886 1 0.645919359 1

DB03223 2 1.418338083 1.75379047 0.753349628 0.628097886 1 0.645919359 1

DB03225 2 2.24548315 1.75379047 0.753349628 0.628097886 1 0.645919359 1

DB03226 1.5 1.564773059 1.745287633 0.542671379 0.325634379 1 0.346299761 1

DB03227 1.5 1.712153002 1.745287633 0.542671379 0.325634379 1 0.346299761 1

DB03233 2 2.387679378 1.75379047 0.753349628 0.628097886 1 0.645919359 1

DB03240 2 1.231130866 1.75379047 0.753349628 0.628097886 1 0.645919359 1

DB03243 2 1.789298323 1.75379047 0.753349628 0.628097886 1 0.645919359 1

DB03245 1 1.231160962 1.75379047 0.753349628 0.1585137 1 0.12201228 1

DB03247 1.6363636 1.689277295 1.759747174 0.221112233 0.28841764 1 0.426448428 1

DB03250 2 1.288832223 1.75379047 0.753349628 0.628097886 1 0.645919359 1

DB03251 2 2.163316321 1.75379047 0.753349628 0.628097886 1 0.645919359 1

DB03253 1 0.54977685 1.75379047 0.753349628 0.1585137 1 0.12201228 1

DB03255 1 2.208136524 1.75379047 0.753349628 0.1585137 1 0.12201228 1

DB03256 1 1.320591672 1.75379047 0.753349628 0.1585137 1 0.12201228 1

DB03262 2 -0.134673334 1.75379047 0.753349628 0.628097886 1 0.645919359 1

DB03267 1 1.359941531 1.75379047 0.753349628 0.1585137 1 0.12201228 1

DB03268 1 1.397248945 1.75379047 0.753349628 0.1585137 1 0.12201228 1

DB03270 2 0.605126254 1.75379047 0.753349628 0.628097886 1 0.645919359 1

DB03273 2 1.520447249 1.75379047 0.753349628 0.628097886 1 0.645919359 1

DB03276 1 1.75341764 1.75379047 0.753349628 0.1585137 1 0.12201228 1

DB03277 2 2.343934368 1.75379047 0.753349628 0.628097886 1 0.645919359 1

DB03279 2 0.746163501 1.75379047 0.753349628 0.628097886 1 0.645919359 1

DB03280 2 2.567436595 1.75379047 0.753349628 0.628097886 1 0.645919359 1

DB03283 2 2.185395932 1.75379047 0.753349628 0.628097886 1 0.645919359 1

DB03286 2 1.412610083 1.75379047 0.753349628 0.628097886 1 0.645919359 1

DB03292 2 3.036696332 1.75379047 0.753349628 0.628097886 1 0.645919359 1

DB03294 2 1.677043508 1.75379047 0.753349628 0.628097886 1 0.645919359 1

DB03297 1 2.41577587 1.757244872 0.464542708 0.051541591 1 0.12201228 1

DB03298 1 1.651793633 1.75379047 0.753349628 0.1585137 1 0.12201228 1

DB03305 1 1.259781445 1.75379047 0.753349628 0.1585137 1 0.12201228 1

DB03306 1 1.590845041 1.75379047 0.753349628 0.1585137 1 0.12201228 1

DB03307 1 2.492047998 1.75379047 0.753349628 0.1585137 1 0.12201228 1

DB03309 1.5 1.878533073 1.745287633 0.542671379 0.325634379 1 0.346299761 1

DB03310 1.3 1.575167252 1.758534835 0.16776606 0.003136302 1 0.240967937 1

DB03311 1 2.836892684 1.75379047 0.753349628 0.1585137 1 0.12201228 1

DB03312 2 1.516388876 1.75379047 0.753349628 0.628097886 1 0.645919359 1

DB03314 1 2.111616019 1.745287633 0.542671379 0.08481895 1 0.12201228 1

DB03316 1.3333333 1.229754471 1.757244872 0.464542708 0.180743525 1 0.257238652 1

DB03317 1.5 1.328540595 1.745287633 0.542671379 0.325634379 1 0.346299761 1

DB03319 2 2.331630174 1.75379047 0.753349628 0.628097886 1 0.645919359 1

DB03323 2 2.181980286 1.75379047 0.753349628 0.628097886 1 0.645919359 1

DB03326 2 0.84362994 1.75379047 0.753349628 0.628097886 1 0.645919359 1

DB03328 2 2.48289657 1.75379047 0.753349628 0.628097886 1 0.645919359 1

DB03329 1 2.261122008 1.75379047 0.753349628 0.1585137 1 0.12201228 1

DB03330 2 1.915974747 1.75379047 0.753349628 0.628097886 1 0.645919359 1

DB03331 2 1.373614441 1.75379047 0.753349628 0.628097886 1 0.645919359 1

DB03332 1 2.350133839 1.75379047 0.753349628 0.1585137 1 0.12201228 1

DB03333 2 1.152484778 1.75379047 0.753349628 0.628097886 1 0.645919359 1

DB03336 1 0.656183067 1.75379047 0.753349628 0.1585137 1 0.12201228 1

DB03337 2 -0.612136129 1.75379047 0.753349628 0.628097886 1 0.645919359 1

DB03338 1.5 0.448692329 1.745287633 0.542671379 0.325634379 1 0.346299761 1

DB03341 1 1.72606695 1.75379047 0.753349628 0.1585137 1 0.12201228 1

DB03344 1 1.149197422 1.75379047 0.753349628 0.1585137 1 0.12201228 1

DB03345 1.6 2.050013488 1.757979426 0.337271213 0.319747599 1 0.404623142 1

DB03346 1 2.087926462 1.75379047 0.753349628 0.1585137 1 0.12201228 1

DB03347 1 1.952313368 1.75379047 0.753349628 0.1585137 1 0.12201228 1

DB03348 2 1.165341948 1.75379047 0.753349628 0.628097886 1 0.645919359 1

DB03349 1 1.772195594 1.745287633 0.542671379 0.08481895 1 0.12201228 1

DB03351 2 2.094800604 1.75379047 0.753349628 0.628097886 1 0.645919359 1

DB03354 2 1.963121061 1.75379047 0.753349628 0.628097886 1 0.645919359 1

DB03359 2 0.625964797 1.75379047 0.753349628 0.628097886 1 0.645919359 1

DB03365 1 3.196712342 1.75379047 0.753349628 0.1585137 1 0.12201228 1

DB03366 1.375 1.419126082 1.761473636 0.259363203 0.068100732 1 0.278352103 1

DB03367 1 2.193887805 1.75379047 0.753349628 0.1585137 1 0.12201228 1

DB03368 2 2.27423261 1.75379047 0.753349628 0.628097886 1 0.645919359 1

DB03370 1 2.366413748 1.75379047 0.753349628 0.1585137 1 0.12201228 1

DB03372 1 1.024367189 1.75379047 0.753349628 0.1585137 1 0.12201228 1

DB03373 2 1.347946968 1.75379047 0.753349628 0.628097886 1 0.645919359 1

DB03374 0.3039526 1.705955585 1.753692879 0.367879736 4.06E-05 0.220242837 0.012662103 1

DB03376 2 1.3117695 1.75379047 0.753349628 0.628097886 1 0.645919359 1

DB03379 1 1.848399814 1.75379047 0.753349628 0.1585137 1 0.12201228 1

DB03380 1.5 1.868268839 1.745287633 0.542671379 0.325634379 1 0.346299761 1

DB03381 1 1.502867823 1.745287633 0.542671379 0.08481895 1 0.12201228 1

DB03382 1 1.679582772 1.75379047 0.753349628 0.1585137 1 0.12201228 1

DB03383 2 1.777392332 1.75379047 0.753349628 0.628097886 1 0.645919359 1

DB03385 1.5 1.521619456 1.745287633 0.542671379 0.325634379 1 0.346299761 1

DB03389 2 1.543785211 1.75379047 0.753349628 0.628097886 1 0.645919359 1

DB03390 1 1.704266653 1.75379047 0.753349628 0.1585137 1 0.12201228 1

DB03392 2 1.926513953 1.75379047 0.753349628 0.628097886 1 0.645919359 1

DB03393 1 1.417727628 1.75379047 0.753349628 0.1585137 1 0.12201228 1

DB03394 1 1.630259895 1.75379047 0.753349628 0.1585137 1 0.12201228 1

DB03395 2 3.108180305 1.75379047 0.753349628 0.628097886 1 0.645919359 1

DB03396 2 1.660266849 1.75379047 0.753349628 0.628097886 1 0.645919359 1

DB03397 1.75 2.584176286 1.753692879 0.367879736 0.495995374 1 0.495810565 1

DB03399 1 0.909241996 1.75379047 0.753349628 0.1585137 1 0.12201228 1

DB03401 1.5 1.662322796 1.770629523 0.307115021 0.189104995 1 0.346299761 1

DB03403 2 1.960506497 1.745287633 0.542671379 0.680596551 1 0.645919359 1

DB03405 2 -0.042686233 1.75379047 0.753349628 0.628097886 1 0.645919359 1

DB03413 2 1.400154878 1.75379047 0.753349628 0.628097886 1 0.645919359 1

DB03414 2 2.496157737 1.75379047 0.753349628 0.628097886 1 0.645919359 1

DB03417 2 2.417885103 1.75379047 0.753349628 0.628097886 1 0.645919359 1

DB03419 2 2.428798056 1.745287633 0.542671379 0.680596551 1 0.645919359 1

DB03424 2 3.098204169 1.75379047 0.753349628 0.628097886 1 0.645919359 1

DB03425 1 2.081505899 1.75379047 0.753349628 0.1585137 1 0.12201228 1

DB03428 1.3333333 1.667345023 1.757244872 0.464542708 0.180743525 1 0.257238652 1

DB03429 2 1.652214154 1.75379047 0.753349628 0.628097886 1 0.645919359 1

DB03430 2 0.68310922 1.75379047 0.753349628 0.628097886 1 0.645919359 1

DB03434 1 2.17103943 1.75379047 0.753349628 0.1585137 1 0.12201228 1

DB03435 1.8333333 1.104894331 1.770629523 0.307115021 0.580889804 1 0.546876824 1

DB03439 2 1.188448098 1.745287633 0.542671379 0.680596551 1 0.645919359 1

DB03441 2 2.660359479 1.75379047 0.753349628 0.628097886 1 0.645919359 1

DB03442 1 1.601313567 1.75379047 0.753349628 0.1585137 1 0.12201228 1

DB03443 2 1.036572684 1.75379047 0.753349628 0.628097886 1 0.645919359 1

DB03444 1 2.737872428 1.75379047 0.753349628 0.1585137 1 0.12201228 1

DB03447 2 2.051799933 1.75379047 0.753349628 0.628097886 1 0.645919359 1

DB03448 2 2.36102162 1.745287633 0.542671379 0.680596551 1 0.645919359 1

DB03449 1 1.081496839 1.745287633 0.542671379 0.08481895 1 0.12201228 1

DB03451 2 3.273636486 1.75379047 0.753349628 0.628097886 1 0.645919359 1

DB03453 1 1.078460714 1.75379047 0.753349628 0.1585137 1 0.12201228 1

DB03455 1 2.040974396 1.75379047 0.753349628 0.1585137 1 0.12201228 1

DB03456 2 2.594828248 1.75379047 0.753349628 0.628097886 1 0.645919359 1

DB03459 2 1.864500191 1.75379047 0.753349628 0.628097886 1 0.645919359 1

DB03461 1.6538462 1.690231756 1.752565715 0.14361026 0.245911353 1 0.43702655 1

DB03466 2 1.313437072 1.75379047 0.753349628 0.628097886 1 0.645919359 1

DB03467 0.2961066 0.9750848 1.736866356 0.292454388 4.19E-07 0.002278152 0.012272212 1

DB03468 2 1.834648649 1.75379047 0.753349628 0.628097886 1 0.645919359 1

DB03471 1 2.022925144 1.75379047 0.753349628 0.1585137 1 0.12201228 1

DB03476 1 0.935206193 1.75379047 0.753349628 0.1585137 1 0.12201228 1

DB03477 1 2.083590653 1.75379047 0.753349628 0.1585137 1 0.12201228 1

DB03478 1 1.557246068 1.75379047 0.753349628 0.1585137 1 0.12201228 1

DB03479 2 1.333611769 1.75379047 0.753349628 0.628097886 1 0.645919359 1

DB03480 2 1.184288594 1.75379047 0.753349628 0.628097886 1 0.645919359 1

DB03483 1 1.89539929 1.75379047 0.753349628 0.1585137 1 0.12201228 1

DB03484 1 2.330837455 1.75379047 0.753349628 0.1585137 1 0.12201228 1

DB03485 1 1.698028021 1.75379047 0.753349628 0.1585137 1 0.12201228 1

DB03486 1 1.970793476 1.75379047 0.753349628 0.1585137 1 0.12201228 1

DB03487 1 0.837764788 1.75379047 0.753349628 0.1585137 1 0.12201228 1

DB03490 1 1.624160771 1.75379047 0.753349628 0.1585137 1 0.12201228 1

DB03491 1 1.373284824 1.75379047 0.753349628 0.1585137 1 0.12201228 1

DB03495 2 1.007357771 1.75379047 0.753349628 0.628097886 1 0.645919359 1

DB03496 1.5454545 1.785506228 1.759747174 0.221112233 0.166233199 1 0.372453903 1

DB03499 1.5 0.897899128 1.745287633 0.542671379 0.325634379 1 0.346299761 1

DB03501 1.5 2.706370201 1.745287633 0.542671379 0.325634379 1 0.346299761 1

DB03504 1 1.987734793 1.75379047 0.753349628 0.1585137 1 0.12201228 1

DB03506 2 2.191083896 1.75379047 0.753349628 0.628097886 1 0.645919359 1

DB03507 1 2.156215237 1.75379047 0.753349628 0.1585137 1 0.12201228 1

DB03509 2 2.570573651 1.75379047 0.753349628 0.628097886 1 0.645919359 1

DB03510 2 2.563443993 1.75379047 0.753349628 0.628097886 1 0.645919359 1

DB03512 2 2.825395688 1.75379047 0.753349628 0.628097886 1 0.645919359 1

DB03516 2 2.650206294 1.757244872 0.464542708 0.699362524 1 0.645919359 1

DB03523 2 1.064202861 1.75379047 0.753349628 0.628097886 1 0.645919359 1

DB03525 1 2.686527126 1.75379047 0.753349628 0.1585137 1 0.12201228 1

DB03526 2 2.631633933 1.75379047 0.753349628 0.628097886 1 0.645919359 1

DB03531 2 1.825091827 1.75379047 0.753349628 0.628097886 1 0.645919359 1

DB03536 2 0.861507475 1.75379047 0.753349628 0.628097886 1 0.645919359 1

DB03539 1 2.418879952 1.75379047 0.753349628 0.1585137 1 0.12201228 1

DB03541 1.5 1.380839475 1.745287633 0.542671379 0.325634379 1 0.346299761 1

DB03542 2 0.489488449 1.75379047 0.753349628 0.628097886 1 0.645919359 1

DB03546 1 3.994495902 1.75379047 0.753349628 0.1585137 1 0.12201228 1

DB03551 2 1.487098713 1.75379047 0.753349628 0.628097886 1 0.645919359 1

DB03552 1 0.601739151 1.75379047 0.753349628 0.1585137 1 0.12201228 1

DB03554 2 1.322901241 1.75379047 0.753349628 0.628097886 1 0.645919359 1

DB03555 2 2.026660833 1.75379047 0.753349628 0.628097886 1 0.645919359 1

DB03556 1 1.252267631 1.75379047 0.753349628 0.1585137 1 0.12201228 1

DB03557 1 2.597590119 1.75379047 0.753349628 0.1585137 1 0.12201228 1

DB03559 2 1.040442416 1.745287633 0.542671379 0.680596551 1 0.645919359 1

DB03565 1 0.966046554 1.75379047 0.753349628 0.1585137 1 0.12201228 1

DB03566 1.6666667 1.458571298 1.757244872 0.464542708 0.422702925 1 0.444812957 1

DB03568 2 1.861084614 1.75379047 0.753349628 0.628097886 1 0.645919359 1

DB03572 1 2.049582475 1.75379047 0.753349628 0.1585137 1 0.12201228 1

DB03573 2 1.989602337 1.75379047 0.753349628 0.628097886 1 0.645919359 1

DB03575 2 0.832547715 1.745287633 0.542671379 0.680596551 1 0.645919359 1

DB03577 1 2.093239975 1.75379047 0.753349628 0.1585137 1 0.12201228 1

DB03583 1 2.2220005 1.75379047 0.753349628 0.1585137 1 0.12201228 1

DB03585 1 3.151822004 1.75379047 0.753349628 0.1585137 1 0.12201228 1

DB03587 1 0.074072294 1.75379047 0.753349628 0.1585137 1 0.12201228 1

DB03588 1 2.288569996 1.75379047 0.753349628 0.1585137 1 0.12201228 1

DB03589 2 2.241390455 1.75379047 0.753349628 0.628097886 1 0.645919359 1

DB03591 1 1.092402165 1.75379047 0.753349628 0.1585137 1 0.12201228 1

DB03593 2 2.530327939 1.75379047 0.753349628 0.628097886 1 0.645919359 1

DB03594 2 2.582185291 1.75379047 0.753349628 0.628097886 1 0.645919359 1

DB03595 2 2.253435065 1.75379047 0.753349628 0.628097886 1 0.645919359 1

DB03596 2 1.304546887 1.75379047 0.753349628 0.628097886 1 0.645919359 1

DB03597 1 2.285112683 1.757244872 0.464542708 0.051541591 1 0.12201228 1

DB03598 2 2.438377713 1.75379047 0.753349628 0.628097886 1 0.645919359 1

DB03600 1.3333333 1.625418296 1.757244872 0.464542708 0.180743525 1 0.257238652 1

DB03602 1.5 2.329577685 1.745287633 0.542671379 0.325634379 1 0.346299761 1

DB03604 1 1.231646502 1.75379047 0.753349628 0.1585137 1 0.12201228 1

DB03606 1 2.640560492 1.745287633 0.542671379 0.08481895 1 0.12201228 1

DB03608 1.6666667 1.894156982 1.757244872 0.464542708 0.422702925 1 0.444812957 1

DB03609 2 1.675518231 1.75379047 0.753349628 0.628097886 1 0.645919359 1

DB03611 2 1.325648362 1.75379047 0.753349628 0.628097886 1 0.645919359 1

DB03612 1 0.83254593 1.75379047 0.753349628 0.1585137 1 0.12201228 1

DB03614 2 0.896331741 1.75379047 0.753349628 0.628097886 1 0.645919359 1

DB03615 1 2.795000023 1.75379047 0.753349628 0.1585137 1 0.12201228 1

DB03616 1 2.784573618 1.75379047 0.753349628 0.1585137 1 0.12201228 1

DB03619 1.75 2.250647333 1.753692879 0.367879736 0.495995374 1 0.495810565 1

DB03621 2 1.94677039 1.75379047 0.753349628 0.628097886 1 0.645919359 1

DB03622 1 1.326147461 1.75379047 0.753349628 0.1585137 1 0.12201228 1

DB03626 1 2.81102932 1.75379047 0.753349628 0.1585137 1 0.12201228 1

DB03628 1 1.545266101 1.75379047 0.753349628 0.1585137 1 0.12201228 1

DB03632 1 1.499957973 1.75379047 0.753349628 0.1585137 1 0.12201228 1

DB03633 1.5 0.982522311 1.745287633 0.542671379 0.325634379 1 0.346299761 1

DB03635 1 1.707501674 1.75379047 0.753349628 0.1585137 1 0.12201228 1

DB03636 1 1.668456338 1.75379047 0.753349628 0.1585137 1 0.12201228 1

DB03637 2 1.023183915 1.75379047 0.753349628 0.628097886 1 0.645919359 1

DB03639 2 1.641071516 1.75379047 0.753349628 0.628097886 1 0.645919359 1

DB03642 2 1.51349291 1.75379047 0.753349628 0.628097886 1 0.645919359 1

DB03643 1.5 2.385549625 1.745287633 0.542671379 0.325634379 1 0.346299761 1

DB03645 2 2.770412641 1.75379047 0.753349628 0.628097886 1 0.645919359 1

DB03650 1 2.769909777 1.75379047 0.753349628 0.1585137 1 0.12201228 1

DB03657 2 1.079974134 1.75379047 0.753349628 0.628097886 1 0.645919359 1

DB03660 1 2.770989577 1.75379047 0.753349628 0.1585137 1 0.12201228 1

DB03661 1.25 1.401712488 1.753692879 0.367879736 0.085471804 1 0.217648936 1

DB03663 1 1.348928983 1.75379047 0.753349628 0.1585137 1 0.12201228 1

DB03664 2 0.67566087 1.75379047 0.753349628 0.628097886 1 0.645919359 1

DB03666 2 1.594331143 1.75379047 0.753349628 0.628097886 1 0.645919359 1

DB03667 2 0.996455962 1.75379047 0.753349628 0.628097886 1 0.645919359 1

DB03670 1 2.238938947 1.75379047 0.753349628 0.1585137 1 0.12201228 1

DB03672 2 2.344970104 1.75379047 0.753349628 0.628097886 1 0.645919359 1

DB03673 2 1.304586827 1.75379047 0.753349628 0.628097886 1 0.645919359 1

DB03677 2 0.528078954 1.75379047 0.753349628 0.628097886 1 0.645919359 1

DB03680 2 2.283338132 1.745287633 0.542671379 0.680596551 1 0.645919359 1

DB03683 1 0.689982024 1.75379047 0.753349628 0.1585137 1 0.12201228 1

DB03685 1.6 2.129190008 1.757979426 0.337271213 0.319747599 1 0.404623142 1

DB03686 1 0.719099588 1.75379047 0.753349628 0.1585137 1 0.12201228 1

DB03690 2 0.987292619 1.75379047 0.753349628 0.628097886 1 0.645919359 1

DB03691 2 2.275778074 1.75379047 0.753349628 0.628097886 1 0.645919359 1

DB03693 2 3.475066595 1.75379047 0.753349628 0.628097886 1 0.645919359 1

DB03695 2 2.79405193 1.75379047 0.753349628 0.628097886 1 0.645919359 1

DB03696 2 1.759751871 1.75379047 0.753349628 0.628097886 1 0.645919359 1

DB03697 2 0.988199272 1.75379047 0.753349628 0.628097886 1 0.645919359 1

DB03701 1 1.267822726 1.75379047 0.753349628 0.1585137 1 0.12201228 1

DB03702 2 1.591368024 1.75379047 0.753349628 0.628097886 1 0.645919359 1

DB03703 2 2.126064549 1.75379047 0.753349628 0.628097886 1 0.645919359 1

DB03704 2 1.479778004 1.75379047 0.753349628 0.628097886 1 0.645919359 1

DB03706 1 0.626205021 1.75379047 0.753349628 0.1585137 1 0.12201228 1

DB03707 1 2.11612628 1.745287633 0.542671379 0.08481895 1 0.12201228 1

DB03708 1 1.451785186 1.75379047 0.753349628 0.1585137 1 0.12201228 1

DB03709 1 1.408402403 1.75379047 0.753349628 0.1585137 1 0.12201228 1

DB03710 1 1.823375909 1.75379047 0.753349628 0.1585137 1 0.12201228 1

DB03711 2 1.032870861 1.75379047 0.753349628 0.628097886 1 0.645919359 1

DB03712 1 1.4788334 1.75379047 0.753349628 0.1585137 1 0.12201228 1

DB03714 1 2.148898906 1.75379047 0.753349628 0.1585137 1 0.12201228 1

DB03719 1 1.746578348 1.75379047 0.753349628 0.1585137 1 0.12201228 1

DB03721 0.6579358 1.938628682 1.736866356 0.292454388 0.000112468 0.608566151 0.045367802 1

DB03722 2 2.490351031 1.75379047 0.753349628 0.628097886 1 0.645919359 1

DB03725 1 0.921219645 1.75379047 0.753349628 0.1585137 1 0.12201228 1

DB03726 2 2.807714456 1.75379047 0.753349628 0.628097886 1 0.645919359 1

DB03727 2 1.801051164 1.75379047 0.753349628 0.628097886 1 0.645919359 1

DB03729 1 1.727689447 1.75379047 0.753349628 0.1585137 1 0.12201228 1

DB03731 1.5 1.423543612 1.745287633 0.542671379 0.325634379 1 0.346299761 1

DB03732 1 0.184818271 1.75379047 0.753349628 0.1585137 1 0.12201228 1

DB03736 2 1.406724832 1.75379047 0.753349628 0.628097886 1 0.645919359 1

DB03737 1 2.400366489 1.75379047 0.753349628 0.1585137 1 0.12201228 1

DB03738 2 1.369766701 1.75379047 0.753349628 0.628097886 1 0.645919359 1

DB03740 1.25 2.124762673 1.753692879 0.367879736 0.085471804 1 0.217648936 1

DB03742 1 1.591750134 1.75379047 0.753349628 0.1585137 1 0.12201228 1

DB03747 1 1.02613796 1.75379047 0.753349628 0.1585137 1 0.12201228 1

DB03749 1 1.872989544 1.75379047 0.753349628 0.1585137 1 0.12201228 1

DB03752 2 1.386931546 1.75379047 0.753349628 0.628097886 1 0.645919359 1

DB03753 2 1.750877194 1.75379047 0.753349628 0.628097886 1 0.645919359 1

DB03754 1 2.877112813 1.75379047 0.753349628 0.1585137 1 0.12201228 1

DB03756 1.3333333 1.352215039 1.770629523 0.307115021 0.07724 1 0.257238652 1

DB03757 2 2.309252392 1.75379047 0.753349628 0.628097886 1 0.645919359 1

DB03758 1.3333333 1.490182679 1.757244872 0.464542708 0.180743525 1 0.257238652 1

DB03759 2 3.14604896 1.75379047 0.753349628 0.628097886 1 0.645919359 1

DB03760 2 1.503373105 1.745287633 0.542671379 0.680596551 1 0.645919359 1

DB03765 2 2.082171614 1.75379047 0.753349628 0.628097886 1 0.645919359 1

DB03766 2 0.824281956 1.75379047 0.753349628 0.628097886 1 0.645919359 1

DB03767 1 1.468014345 1.75379047 0.753349628 0.1585137 1 0.12201228 1

DB03769 2 1.775895661 1.75379047 0.753349628 0.628097886 1 0.645919359 1

DB03773 2 1.188140158 1.75379047 0.753349628 0.628097886 1 0.645919359 1

DB03777 1 2.287945299 1.757244872 0.464542708 0.051541591 1 0.12201228 1

DB03781 2 1.953240542 1.75379047 0.753349628 0.628097886 1 0.645919359 1

DB03782 1 1.461276556 1.75379047 0.753349628 0.1585137 1 0.12201228 1

DB03783 2 2.048388117 1.75379047 0.753349628 0.628097886 1 0.645919359 1

DB03784 1 1.957036894 1.75379047 0.753349628 0.1585137 1 0.12201228 1

DB03788 1 3.204799372 1.75379047 0.753349628 0.1585137 1 0.12201228 1

DB03791 2 0.17737728 1.75379047 0.753349628 0.628097886 1 0.645919359 1

DB03792 2 1.09888507 1.75379047 0.753349628 0.628097886 1 0.645919359 1

DB03793 2 0.982977666 1.745287633 0.542671379 0.680596551 1 0.645919359 1

DB03796 1.3333333 2.059566978 1.770629523 0.307115021 0.07724 1 0.257238652 1

DB03800 1 3.684982412 1.75379047 0.753349628 0.1585137 1 0.12201228 1

DB03801 1 2.390420557 1.75379047 0.753349628 0.1585137 1 0.12201228 1

DB03802 1 0.252917508 1.75379047 0.753349628 0.1585137 1 0.12201228 1

DB03807 1 1.342615803 1.75379047 0.753349628 0.1585137 1 0.12201228 1

DB03809 1 2.403066926 1.75379047 0.753349628 0.1585137 1 0.12201228 1

DB03814 1.6666667 1.859970553 1.763093512 0.211303302 0.324071624 1 0.444812957 1

DB03822 2 0.344063516 1.75379047 0.753349628 0.628097886 1 0.645919359 1

DB03824 2 0.637214866 1.75379047 0.753349628 0.628097886 1 0.645919359 1

DB03828 1 2.045863916 1.75379047 0.753349628 0.1585137 1 0.12201228 1

DB03832 2 2.294674456 1.75379047 0.753349628 0.628097886 1 0.645919359 1

DB03835 2 2.491032124 1.75379047 0.753349628 0.628097886 1 0.645919359 1

DB03837 1 1.996186096 1.75379047 0.753349628 0.1585137 1 0.12201228 1

DB03841 2 0.875220092 1.75379047 0.753349628 0.628097886 1 0.645919359 1

DB03844 2 2.908993361 1.75379047 0.753349628 0.628097886 1 0.645919359 1

DB03845 2 1.738014303 1.75379047 0.753349628 0.628097886 1 0.645919359 1

DB03847 1.6666667 1.444477805 1.757244872 0.464542708 0.422702925 1 0.444812957 1

DB03848 2 2.30004186 1.75379047 0.753349628 0.628097886 1 0.645919359 1

DB03849 1 1.951355167 1.745287633 0.542671379 0.08481895 1 0.12201228 1

DB03850 1 1.553884549 1.75379047 0.753349628 0.1585137 1 0.12201228 1

DB03856 1 0.153041239 1.75379047 0.753349628 0.1585137 1 0.12201228 1

DB03860 1 2.280389196 1.75379047 0.753349628 0.1585137 1 0.12201228 1

DB03861 1.5 0.941147409 1.745287633 0.542671379 0.325634379 1 0.346299761 1

DB03865 1.25 2.332004226 1.753692879 0.367879736 0.085471804 1 0.217648936 1

DB03866 1 1.77056317 1.75379047 0.753349628 0.1585137 1 0.12201228 1

DB03867 2 1.21594596 1.75379047 0.753349628 0.628097886 1 0.645919359 1

DB03876 1.5 2.072765256 1.745287633 0.542671379 0.325634379 1 0.346299761 1

DB03877 2 1.633636131 1.75379047 0.753349628 0.628097886 1 0.645919359 1

DB03878 1 2.343751003 1.75379047 0.753349628 0.1585137 1 0.12201228 1

DB03879 2 2.549073104 1.75379047 0.753349628 0.628097886 1 0.645919359 1

DB03880 1.2 1.530747157 1.757979426 0.337271213 0.049023756 1 0.195688273 1

DB03881 2 2.08561067 1.75379047 0.753349628 0.628097886 1 0.645919359 1

DB03882 1 1.760663377 1.75379047 0.753349628 0.1585137 1 0.12201228 1

DB03884 2 2.59437809 1.75379047 0.753349628 0.628097886 1 0.645919359 1

DB03886 1.6666667 1.936212608 1.757244872 0.464542708 0.422702925 1 0.444812957 1

DB03889 2 1.888168576 1.75379047 0.753349628 0.628097886 1 0.645919359 1

DB03890 2 2.335204043 1.75379047 0.753349628 0.628097886 1 0.645919359 1

DB03891 2 1.956971229 1.75379047 0.753349628 0.628097886 1 0.645919359 1

DB03892 1 1.951059744 1.75379047 0.753349628 0.1585137 1 0.12201228 1

DB03893 1 1.648375055 1.75379047 0.753349628 0.1585137 1 0.12201228 1

DB03894 1 3.28801543 1.75379047 0.753349628 0.1585137 1 0.12201228 1

DB03896 2 2.244172017 1.75379047 0.753349628 0.628097886 1 0.645919359 1

DB03899 1 1.300580011 1.75379047 0.753349628 0.1585137 1 0.12201228 1

DB03900 1.5 2.040778448 1.753692879 0.367879736 0.245220308 1 0.346299761 1

DB03902 1 1.476606261 1.75379047 0.753349628 0.1585137 1 0.12201228 1

DB03903 1 2.42956653 1.75379047 0.753349628 0.1585137 1 0.12201228 1

DB03904 1.3333333 1.665335234 1.757244872 0.464542708 0.180743525 1 0.257238652 1

DB03906 1 1.738484355 1.75379047 0.753349628 0.1585137 1 0.12201228 1

DB03907 1 2.192139408 1.75379047 0.753349628 0.1585137 1 0.12201228 1

DB03909 1.4 1.473820337 1.757979426 0.337271213 0.14425424 1 0.291413148 1

DB03910 1 1.42066468 1.75379047 0.753349628 0.1585137 1 0.12201228 1

DB03918 1 2.208693472 1.75379047 0.753349628 0.1585137 1 0.12201228 1

DB03921 2 0.637033349 1.75379047 0.753349628 0.628097886 1 0.645919359 1

DB03923 2 1.157369142 1.75379047 0.753349628 0.628097886 1 0.645919359 1

DB03924 2 0.841416628 1.75379047 0.753349628 0.628097886 1 0.645919359 1

DB03925 1.5 2.00393401 1.745287633 0.542671379 0.325634379 1 0.346299761 1

DB03928 2 0.669144048 1.75379047 0.753349628 0.628097886 1 0.645919359 1

DB03929 1.6666667 2.232805283 1.757244872 0.464542708 0.422702925 1 0.444812957 1

DB03932 1 1.579443724 1.75379047 0.753349628 0.1585137 1 0.12201228 1

DB03934 1 1.050802952 1.75379047 0.753349628 0.1585137 1 0.12201228 1

DB03935 1 0.657313694 1.75379047 0.753349628 0.1585137 1 0.12201228 1

DB03940 2 2.046487375 1.745287633 0.542671379 0.680596551 1 0.645919359 1

DB03944 2 1.634506327 1.75379047 0.753349628 0.628097886 1 0.645919359 1

DB03945 1.5 2.349499292 1.745287633 0.542671379 0.325634379 1 0.346299761 1

DB03948 2 0.733702986 1.745287633 0.542671379 0.680596551 1 0.645919359 1

DB03950 2 0.99963432 1.75379047 0.753349628 0.628097886 1 0.645919359 1

DB03951 2 1.581845241 1.75379047 0.753349628 0.628097886 1 0.645919359 1

DB03953 1 3.069378792 1.75379047 0.753349628 0.1585137 1 0.12201228 1

DB03955 2 1.88619971 1.75379047 0.753349628 0.628097886 1 0.645919359 1

DB03956 1 1.71435444 1.75379047 0.753349628 0.1585137 1 0.12201228 1

DB03957 1 1.475338112 1.75379047 0.753349628 0.1585137 1 0.12201228 1

DB03958 2 1.891531372 1.75379047 0.753349628 0.628097886 1 0.645919359 1

DB03959 1.5 1.345840434 1.753692879 0.367879736 0.245220308 1 0.346299761 1

DB03962 2 2.03554427 1.75379047 0.753349628 0.628097886 1 0.645919359 1

DB03963 1.5 2.606283447 1.753692879 0.367879736 0.245220308 1 0.346299761 1

DB03964 2 1.471165688 1.75379047 0.753349628 0.628097886 1 0.645919359 1

DB03967 1 1.355313853 1.75379047 0.753349628 0.1585137 1 0.12201228 1

DB03968 2 1.554138816 1.75379047 0.753349628 0.628097886 1 0.645919359 1

DB03971 2 2.011922815 1.75379047 0.753349628 0.628097886 1 0.645919359 1

DB03974 1 2.082884127 1.75379047 0.753349628 0.1585137 1 0.12201228 1

DB03975 2 1.697822024 1.75379047 0.753349628 0.628097886 1 0.645919359 1

DB03976 2 1.849359007 1.745287633 0.542671379 0.680596551 1 0.645919359 1

DB03977 1 2.204778406 1.745287633 0.542671379 0.08481895 1 0.12201228 1

DB03978 2 3.246530733 1.75379047 0.753349628 0.628097886 1 0.645919359 1

DB03980 1 2.865556974 1.75379047 0.753349628 0.1585137 1 0.12201228 1

DB03981 1.5 1.105162934 1.745287633 0.542671379 0.325634379 1 0.346299761 1

DB03982 1 2.722444722 1.75379047 0.753349628 0.1585137 1 0.12201228 1

DB03983 2 1.47412309 1.75379047 0.753349628 0.628097886 1 0.645919359 1

DB03984 1 2.188832233 1.75379047 0.753349628 0.1585137 1 0.12201228 1

DB03987 2 2.967001403 1.75379047 0.753349628 0.628097886 1 0.645919359 1

DB03988 2 2.352351349 1.75379047 0.753349628 0.628097886 1 0.645919359 1

DB03991 2 2.243652371 1.75379047 0.753349628 0.628097886 1 0.645919359 1

DB03994 1 1.46746605 1.75379047 0.753349628 0.1585137 1 0.12201228 1

DB03996 2 1.547654049 1.75379047 0.753349628 0.628097886 1 0.645919359 1

DB04000 2 2.482673913 1.75379047 0.753349628 0.628097886 1 0.645919359 1

DB04001 1 1.647362235 1.75379047 0.753349628 0.1585137 1 0.12201228 1

DB04002 2 3.500782512 1.75379047 0.753349628 0.628097886 1 0.645919359 1

DB04003 -6.0684256 2.587706365 1.75379047 0.753349628 1.48E-25 8.11E-22 1.02E-33 5.62E-30

DB04005 2 1.648801796 1.75379047 0.753349628 0.628097886 1 0.645919359 1

DB04006 1 2.453419756 1.75379047 0.753349628 0.1585137 1 0.12201228 1

DB04008 2 0.444385572 1.75379047 0.753349628 0.628097886 1 0.645919359 1

DB04009 1 0.136655815 1.75379047 0.753349628 0.1585137 1 0.12201228 1

DB04010 2 1.673010614 1.75379047 0.753349628 0.628097886 1 0.645919359 1

DB04012 2 1.180772733 1.75379047 0.753349628 0.628097886 1 0.645919359 1

DB04013 2 1.937065216 1.75379047 0.753349628 0.628097886 1 0.645919359 1

DB04014 1.3333333 2.556755927 1.757244872 0.464542708 0.180743525 1 0.257238652 1

DB04016 1 2.436888876 1.745287633 0.542671379 0.08481895 1 0.12201228 1

DB04017 1 1.169531854 1.75379047 0.753349628 0.1585137 1 0.12201228 1

DB04018 1 2.836329808 1.75379047 0.753349628 0.1585137 1 0.12201228 1

DB04020 1 2.553068983 1.75379047 0.753349628 0.1585137 1 0.12201228 1

DB04021 2 2.692978826 1.75379047 0.753349628 0.628097886 1 0.645919359 1

DB04027 2 3.052959987 1.75379047 0.753349628 0.628097886 1 0.645919359 1

DB04043 1 1.956762265 1.75379047 0.753349628 0.1585137 1 0.12201228 1

DB04044 2 2.270868625 1.75379047 0.753349628 0.628097886 1 0.645919359 1

DB04048 2 0.522488878 1.75379047 0.753349628 0.628097886 1 0.645919359 1

DB04050 1 2.751247732 1.75379047 0.753349628 0.1585137 1 0.12201228 1

DB04054 1 2.22595914 1.75379047 0.753349628 0.1585137 1 0.12201228 1

DB04055 2 1.649077126 1.75379047 0.753349628 0.628097886 1 0.645919359 1

DB04057 1 3.080872734 1.75379047 0.753349628 0.1585137 1 0.12201228 1

DB04058 2 1.964481548 1.75379047 0.753349628 0.628097886 1 0.645919359 1

DB04059 1 1.094047242 1.75379047 0.753349628 0.1585137 1 0.12201228 1

DB04065 2 1.874356763 1.75379047 0.753349628 0.628097886 1 0.645919359 1

DB04066 2 1.465212654 1.75379047 0.753349628 0.628097886 1 0.645919359 1

DB04068 1 2.899683458 1.75379047 0.753349628 0.1585137 1 0.12201228 1

DB04069 2 1.959359869 1.75379047 0.753349628 0.628097886 1 0.645919359 1

DB04071 2 1.486064463 1.75379047 0.753349628 0.628097886 1 0.645919359 1

DB04072 2 1.911944834 1.75379047 0.753349628 0.628097886 1 0.645919359 1

DB04074 2 2.253924812 1.75379047 0.753349628 0.628097886 1 0.645919359 1

DB04076 2 1.689667146 1.75379047 0.753349628 0.628097886 1 0.645919359 1

DB04079 1 1.731904147 1.75379047 0.753349628 0.1585137 1 0.12201228 1

DB04080 1 1.68432188 1.75379047 0.753349628 0.1585137 1 0.12201228 1

DB04081 2 2.70798276 1.75379047 0.753349628 0.628097886 1 0.645919359 1

DB04083 1.3333333 1.852720822 1.757244872 0.464542708 0.180743525 1 0.257238652 1

DB04088 1 1.404158209 1.75379047 0.753349628 0.1585137 1 0.12201228 1

DB04089 2 1.521179023 1.75379047 0.753349628 0.628097886 1 0.645919359 1

DB04090 1 1.302149117 1.75379047 0.753349628 0.1585137 1 0.12201228 1

DB04094 2 1.530975506 1.75379047 0.753349628 0.628097886 1 0.645919359 1

DB04098 1 1.942911683 1.75379047 0.753349628 0.1585137 1 0.12201228 1

DB04099 2 2.10719483 1.75379047 0.753349628 0.628097886 1 0.645919359 1

DB04101 1 3.352244244 1.75379047 0.753349628 0.1585137 1 0.12201228 1

DB04105 2 -0.318524939 1.75379047 0.753349628 0.628097886 1 0.645919359 1

DB04106 1 2.204196456 1.75379047 0.753349628 0.1585137 1 0.12201228 1

DB04107 2 0.056459145 1.75379047 0.753349628 0.628097886 1 0.645919359 1

DB04108 2 1.752564403 1.75379047 0.753349628 0.628097886 1 0.645919359 1

DB04109 2 2.154456332 1.75379047 0.753349628 0.628097886 1 0.645919359 1

DB04112 1 1.528139939 1.75379047 0.753349628 0.1585137 1 0.12201228 1

DB04113 2 2.052727514 1.75379047 0.753349628 0.628097886 1 0.645919359 1

DB04114 2 2.915017 1.75379047 0.753349628 0.628097886 1 0.645919359 1

DB04117 2 2.097315115 1.75379047 0.753349628 0.628097886 1 0.645919359 1

DB04119 1 3.112010862 1.75379047 0.753349628 0.1585137 1 0.12201228 1

DB04121 1 1.325142381 1.75379047 0.753349628 0.1585137 1 0.12201228 1

DB04125 2 2.524549547 1.75379047 0.753349628 0.628097886 1 0.645919359 1

DB04126 1.5 2.491992399 1.745287633 0.542671379 0.325634379 1 0.346299761 1

DB04129 2 1.491593898 1.75379047 0.753349628 0.628097886 1 0.645919359 1

DB04132 1.25 2.075796839 1.753692879 0.367879736 0.085471804 1 0.217648936 1

DB04136 1 0.703837235 1.75379047 0.753349628 0.1585137 1 0.12201228 1

DB04137 1.6 1.581426306 1.759693587 0.247672276 0.259535593 1 0.404623142 1

DB04140 2 1.932972728 1.75379047 0.753349628 0.628097886 1 0.645919359 1

DB04141 1.6428571 1.500541205 1.773663076 0.187305714 0.242477448 1 0.43037167 1

DB04142 1 1.994875604 1.75379047 0.753349628 0.1585137 1 0.12201228 1

DB04144 1 2.547120778 1.75379047 0.753349628 0.1585137 1 0.12201228 1

DB04147 1.4285714 1.894111676 1.736866356 0.292454388 0.145903866 1 0.306680831 1

DB04149 1 1.29396461 1.745287633 0.542671379 0.08481895 1 0.12201228 1

DB04150 2 2.358350971 1.75379047 0.753349628 0.628097886 1 0.645919359 1

DB04151 1 1.141645569 1.75379047 0.753349628 0.1585137 1 0.12201228 1

DB04152 2 0.723593961 1.75379047 0.753349628 0.628097886 1 0.645919359 1

DB04153 2 2.676628195 1.75379047 0.753349628 0.628097886 1 0.645919359 1

DB04156 2 1.41927264 1.75379047 0.753349628 0.628097886 1 0.645919359 1

DB04163 2 1.621944909 1.75379047 0.753349628 0.628097886 1 0.645919359 1

DB04166 1.5 1.620789614 1.745287633 0.542671379 0.325634379 1 0.346299761 1

DB04172 1 2.732181933 1.75379047 0.753349628 0.1585137 1 0.12201228 1

DB04175 1 3.428030543 1.75379047 0.753349628 0.1585137 1 0.12201228 1

DB04178 1.5 1.826924148 1.745287633 0.542671379 0.325634379 1 0.346299761 1

DB04180 2 1.672003624 1.75379047 0.753349628 0.628097886 1 0.645919359 1

DB04185 2 0.916050117 1.745287633 0.542671379 0.680596551 1 0.645919359 1

DB04186 1 0.676589141 1.75379047 0.753349628 0.1585137 1 0.12201228 1

DB04187 1 0.801639252 1.75379047 0.753349628 0.1585137 1 0.12201228 1

DB04188 1 1.629082926 1.75379047 0.753349628 0.1585137 1 0.12201228 1

DB04194 1 1.125983241 1.75379047 0.753349628 0.1585137 1 0.12201228 1

DB04195 2 2.198143601 1.75379047 0.753349628 0.628097886 1 0.645919359 1

DB04197 1 3.075097269 1.75379047 0.753349628 0.1585137 1 0.12201228 1

DB04200 2 1.238414658 1.75379047 0.753349628 0.628097886 1 0.645919359 1

DB04203 2 1.758226959 1.75379047 0.753349628 0.628097886 1 0.645919359 1

DB04204 1 1.019203558 1.75379047 0.753349628 0.1585137 1 0.12201228 1

DB04207 2 1.936608441 1.75379047 0.753349628 0.628097886 1 0.645919359 1

DB04209 1 2.034223315 1.757244872 0.464542708 0.051541591 1 0.12201228 1

DB04213 2 1.428219023 1.75379047 0.753349628 0.628097886 1 0.645919359 1

DB04214 1 1.850172248 1.745287633 0.542671379 0.08481895 1 0.12201228 1

DB04215 2 0.971712349 1.75379047 0.753349628 0.628097886 1 0.645919359 1

DB04216 1.2029099 1.478652309 1.76622404 0.151326267 9.86E-05 0.533854454 0.196928262 1

DB04217 2 1.991319927 1.75379047 0.753349628 0.628097886 1 0.645919359 1

DB04218 1 1.362937169 1.75379047 0.753349628 0.1585137 1 0.12201228 1

DB04223 1 2.223357445 1.745287633 0.542671379 0.08481895 1 0.12201228 1

DB04224 1.4 1.958405792 1.757979426 0.337271213 0.14425424 1 0.291413148 1

DB04230 1 2.56948149 1.75379047 0.753349628 0.1585137 1 0.12201228 1

DB04232 2 2.04301198 1.75379047 0.753349628 0.628097886 1 0.645919359 1

DB04233 1 2.046924523 1.757244872 0.464542708 0.051541591 1 0.12201228 1

DB04234 2 1.920082529 1.75379047 0.753349628 0.628097886 1 0.645919359 1

DB04235 2 1.080037656 1.75379047 0.753349628 0.628097886 1 0.645919359 1

DB04237 2 2.04555343 1.75379047 0.753349628 0.628097886 1 0.645919359 1

DB04238 2 0.998929144 1.75379047 0.753349628 0.628097886 1 0.645919359 1

DB04244 2 0.41029731 1.75379047 0.753349628 0.628097886 1 0.645919359 1

DB04246 2 2.158060341 1.75379047 0.753349628 0.628097886 1 0.645919359 1

DB04249 1 0.892465815 1.75379047 0.753349628 0.1585137 1 0.12201228 1

DB04250 2 1.019933555 1.75379047 0.753349628 0.628097886 1 0.645919359 1

DB04253 2 1.085239448 1.75379047 0.753349628 0.628097886 1 0.645919359 1

DB04254 1 1.518662657 1.75379047 0.753349628 0.1585137 1 0.12201228 1

DB04256 1 1.681674427 1.75379047 0.753349628 0.1585137 1 0.12201228 1

DB04258 2 0.900916442 1.75379047 0.753349628 0.628097886 1 0.645919359 1

DB04260 2 0.572851936 1.75379047 0.753349628 0.628097886 1 0.645919359 1

DB04263 1 0.931525796 1.75379047 0.753349628 0.1585137 1 0.12201228 1

DB04268 1 2.170385288 1.75379047 0.753349628 0.1585137 1 0.12201228 1

DB04269 2 3.227575435 1.75379047 0.753349628 0.628097886 1 0.645919359 1

DB04270 1 1.087419984 1.75379047 0.753349628 0.1585137 1 0.12201228 1

DB04271 1 1.796804661 1.75379047 0.753349628 0.1585137 1 0.12201228 1

DB04272 1.6428571 1.550172706 1.773663076 0.187305714 0.242477448 1 0.43037167 1

DB04273 2 1.113711637 1.75379047 0.753349628 0.628097886 1 0.645919359 1

DB04275 2 2.486732347 1.75379047 0.753349628 0.628097886 1 0.645919359 1

DB04276 2 2.34531817 1.75379047 0.753349628 0.628097886 1 0.645919359 1

DB04278 1 1.608649359 1.75379047 0.753349628 0.1585137 1 0.12201228 1

DB04281 2 2.209283274 1.75379047 0.753349628 0.628097886 1 0.645919359 1

DB04282 2 2.498099585 1.75379047 0.753349628 0.628097886 1 0.645919359 1

DB04285 1 1.56359733 1.75379047 0.753349628 0.1585137 1 0.12201228 1

DB04287 1 2.667587439 1.75379047 0.753349628 0.1585137 1 0.12201228 1

DB04288 1 2.562751526 1.75379047 0.753349628 0.1585137 1 0.12201228 1

DB04295 2 2.43332902 1.75379047 0.753349628 0.628097886 1 0.645919359 1

DB04297 2 0.806831882 1.745287633 0.542671379 0.680596551 1 0.645919359 1

DB04299 2 2.049898566 1.75379047 0.753349628 0.628097886 1 0.645919359 1

DB04301 2 1.361417089 1.75379047 0.753349628 0.628097886 1 0.645919359 1

DB04306 2 2.970139096 1.75379047 0.753349628 0.628097886 1 0.645919359 1

DB04307 1 1.604510339 1.75379047 0.753349628 0.1585137 1 0.12201228 1

DB04310 2 1.647841103 1.75379047 0.753349628 0.628097886 1 0.645919359 1

DB04311 2 1.347600854 1.75379047 0.753349628 0.628097886 1 0.645919359 1

DB04312 2 1.147840413 1.75379047 0.753349628 0.628097886 1 0.645919359 1

DB04315 1.5714286 1.76371856 1.756224429 0.126770408 0.072458793 1 0.387676968 1

DB04316 2 2.440302119 1.75379047 0.753349628 0.628097886 1 0.645919359 1

DB04318 2 1.52711754 1.75379047 0.753349628 0.628097886 1 0.645919359 1

DB04322 2 2.319368903 1.75379047 0.753349628 0.628097886 1 0.645919359 1

DB04324 2 1.676134967 1.745287633 0.542671379 0.680596551 1 0.645919359 1

DB04325 2 1.861710648 1.75379047 0.753349628 0.628097886 1 0.645919359 1

DB04326 1 1.813521739 1.75379047 0.753349628 0.1585137 1 0.12201228 1

DB04327 1 0.566253378 1.745287633 0.542671379 0.08481895 1 0.12201228 1

DB04331 2 1.157296488 1.75379047 0.753349628 0.628097886 1 0.645919359 1

DB04334 1 2.98172582 1.75379047 0.753349628 0.1585137 1 0.12201228 1

DB04335 2 1.548762099 1.75379047 0.753349628 0.628097886 1 0.645919359 1

DB04336 2 2.074014463 1.75379047 0.753349628 0.628097886 1 0.645919359 1

DB04337 1 2.214250949 1.75379047 0.753349628 0.1585137 1 0.12201228 1

DB04338 1 1.505953302 1.745287633 0.542671379 0.08481895 1 0.12201228 1

DB04339 1 2.07965849 1.75379047 0.753349628 0.1585137 1 0.12201228 1

DB04345 1 1.360082311 1.75379047 0.753349628 0.1585137 1 0.12201228 1

DB04348 2 1.731373646 1.757244872 0.464542708 0.699362524 1 0.645919359 1

DB04350 1 1.100436745 1.75379047 0.753349628 0.1585137 1 0.12201228 1

DB04351 2 1.879404653 1.75379047 0.753349628 0.628097886 1 0.645919359 1

DB04352 2 2.328731672 1.75379047 0.753349628 0.628097886 1 0.645919359 1

DB04356 1 1.726405641 1.75379047 0.753349628 0.1585137 1 0.12201228 1

DB04363 1 1.477073764 1.75379047 0.753349628 0.1585137 1 0.12201228 1

DB04365 1 1.160814823 1.745287633 0.542671379 0.08481895 1 0.12201228 1

DB04366 1 1.410784612 1.75379047 0.753349628 0.1585137 1 0.12201228 1

DB04367 2 2.367042379 1.75379047 0.753349628 0.628097886 1 0.645919359 1

DB04368 2 0.800765121 1.75379047 0.753349628 0.628097886 1 0.645919359 1

DB04369 2 2.532602991 1.75379047 0.753349628 0.628097886 1 0.645919359 1

DB04371 2 1.060284957 1.75379047 0.753349628 0.628097886 1 0.645919359 1

DB04372 2 0.568641052 1.75379047 0.753349628 0.628097886 1 0.645919359 1

DB04374 2 2.861792552 1.75379047 0.753349628 0.628097886 1 0.645919359 1

DB04376 1 2.537949405 1.75379047 0.753349628 0.1585137 1 0.12201228 1

DB04377 2 2.705998451 1.75379047 0.753349628 0.628097886 1 0.645919359 1

DB04379 2 1.855287128 1.75379047 0.753349628 0.628097886 1 0.645919359 1

DB04381 2 1.719790798 1.75379047 0.753349628 0.628097886 1 0.645919359 1

DB04387 2 1.760188476 1.75379047 0.753349628 0.628097886 1 0.645919359 1

DB04389 2 1.600873464 1.75379047 0.753349628 0.628097886 1 0.645919359 1

DB04391 2 2.16395606 1.75379047 0.753349628 0.628097886 1 0.645919359 1

DB04392 1 1.633561251 1.75379047 0.753349628 0.1585137 1 0.12201228 1

DB04394 2 1.927959189 1.75379047 0.753349628 0.628097886 1 0.645919359 1

DB04395 1.2241625 2.096461986 1.757638217 0.154827625 0.000284885 1 0.206128003 1

DB04396 1.5 2.570079671 1.745287633 0.542671379 0.325634379 1 0.346299761 1

DB04397 2 1.876303695 1.75379047 0.753349628 0.628097886 1 0.645919359 1

DB04400 1.5 1.422742675 1.753692879 0.367879736 0.245220308 1 0.346299761 1

DB04404 1 2.011543877 1.75379047 0.753349628 0.1585137 1 0.12201228 1

DB04405 1 1.646042225 1.75379047 0.753349628 0.1585137 1 0.12201228 1

DB04407 1 1.400285148 1.75379047 0.753349628 0.1585137 1 0.12201228 1

DB04409 2 0.685341469 1.75379047 0.753349628 0.628097886 1 0.645919359 1

DB04410 2 0.602761208 1.75379047 0.753349628 0.628097886 1 0.645919359 1

DB04416 2 1.739127102 1.75379047 0.753349628 0.628097886 1 0.645919359 1

DB04417 1.5 2.042730025 1.745287633 0.542671379 0.325634379 1 0.346299761 1

DB04418 2 1.697715656 1.75379047 0.753349628 0.628097886 1 0.645919359 1

DB04419 1.5 1.270215973 1.745287633 0.542671379 0.325634379 1 0.346299761 1

DB04424 2 2.425193487 1.75379047 0.753349628 0.628097886 1 0.645919359 1

DB04426 2 1.889599796 1.75379047 0.753349628 0.628097886 1 0.645919359 1

DB04429 1 2.376422828 1.75379047 0.753349628 0.1585137 1 0.12201228 1

DB04432 2 1.562876819 1.75379047 0.753349628 0.628097886 1 0.645919359 1

DB04434 2 1.607465015 1.75379047 0.753349628 0.628097886 1 0.645919359 1

DB04436 1 2.071997145 1.75379047 0.753349628 0.1585137 1 0.12201228 1

DB04440 1 1.596678007 1.75379047 0.753349628 0.1585137 1 0.12201228 1

DB04442 2 1.108048177 1.75379047 0.753349628 0.628097886 1 0.645919359 1

DB04444 1.6 1.555525791 1.757979426 0.337271213 0.319747599 1 0.404623142 1

DB04445 1 1.522923657 1.75379047 0.753349628 0.1585137 1 0.12201228 1

DB04446 2 2.702258734 1.75379047 0.753349628 0.628097886 1 0.645919359 1

DB04447 1.4285714 1.474951383 1.736866356 0.292454388 0.145903866 1 0.306680831 1

DB04448 2 1.685148822 1.75379047 0.753349628 0.628097886 1 0.645919359 1

DB04450 1 2.459800119 1.75379047 0.753349628 0.1585137 1 0.12201228 1

DB04451 2 2.250511148 1.75379047 0.753349628 0.628097886 1 0.645919359 1

DB04453 2 2.74025206 1.75379047 0.753349628 0.628097886 1 0.645919359 1

DB04454 1.5 1.894732736 1.745287633 0.542671379 0.325634379 1 0.346299761 1

DB04459 1 1.763153325 1.75379047 0.753349628 0.1585137 1 0.12201228 1

DB04461 2 1.23371658 1.75379047 0.753349628 0.628097886 1 0.645919359 1

DB04462 2 0.729291112 1.75379047 0.753349628 0.628097886 1 0.645919359 1

DB04463 1 1.819718275 1.75379047 0.753349628 0.1585137 1 0.12201228 1

DB04464 1.6666667 1.640168708 1.763665626 0.163272037 0.276224647 1 0.444812957 1

DB04465 1.75 1.52731651 1.753692879 0.367879736 0.495995374 1 0.495810565 1

DB04466 1 2.187482989 1.75379047 0.753349628 0.1585137 1 0.12201228 1

DB04468 1.2857143 1.869477337 1.736866356 0.292454388 0.061458983 1 0.234170029 1

DB04469 1 1.551592241 1.75379047 0.753349628 0.1585137 1 0.12201228 1

DB04470 2 1.7424236 1.75379047 0.753349628 0.628097886 1 0.645919359 1

DB04471 1 1.240185964 1.75379047 0.753349628 0.1585137 1 0.12201228 1

DB04472 1 1.275641152 1.75379047 0.753349628 0.1585137 1 0.12201228 1

DB04474 1 2.603630695 1.75379047 0.753349628 0.1585137 1 0.12201228 1

DB04476 1 1.485287315 1.75379047 0.753349628 0.1585137 1 0.12201228 1

DB04477 2 2.813790342 1.75379047 0.753349628 0.628097886 1 0.645919359 1

DB04478 2 1.672853166 1.75379047 0.753349628 0.628097886 1 0.645919359 1

DB04480 2 1.087389185 1.75379047 0.753349628 0.628097886 1 0.645919359 1

DB04485 2 2.300589763 1.75379047 0.753349628 0.628097886 1 0.645919359 1

DB04487 1 2.127304159 1.75379047 0.753349628 0.1585137 1 0.12201228 1

DB04489 1 0.976234886 1.75379047 0.753349628 0.1585137 1 0.12201228 1

DB04491 1.5 2.483102548 1.745287633 0.542671379 0.325634379 1 0.346299761 1

DB04493 1.6666667 2.234125887 1.757244872 0.464542708 0.422702925 1 0.444812957 1

DB04495 1 0.847728966 1.75379047 0.753349628 0.1585137 1 0.12201228 1

DB04505 1 0.870356917 1.75379047 0.753349628 0.1585137 1 0.12201228 1

DB04510 1.6666667 2.440206127 1.757244872 0.464542708 0.422702925 1 0.444812957 1

DB04512 2 2.654699064 1.75379047 0.753349628 0.628097886 1 0.645919359 1

DB04513 1.6666667 1.350575334 1.757244872 0.464542708 0.422702925 1 0.444812957 1

DB04514 2 1.71089135 1.75379047 0.753349628 0.628097886 1 0.645919359 1

DB04516 2 0.002697323 1.75379047 0.753349628 0.628097886 1 0.645919359 1

DB04518 1 1.229396227 1.75379047 0.753349628 0.1585137 1 0.12201228 1

DB04519 1 2.708661324 1.75379047 0.753349628 0.1585137 1 0.12201228 1

DB04521 1 1.222577298 1.75379047 0.753349628 0.1585137 1 0.12201228 1

DB04522 1.3333333 1.624793542 1.763093512 0.211303302 0.020983124 1 0.257238652 1

DB04523 2 2.768165482 1.75379047 0.753349628 0.628097886 1 0.645919359 1

DB04525 1 3.198479373 1.75379047 0.753349628 0.1585137 1 0.12201228 1

DB04527 2 1.19082893 1.75379047 0.753349628 0.628097886 1 0.645919359 1

DB04530 1.1111111 2.15689704 1.753207263 0.264393406 0.007579386 1 0.160123241 1

DB04534 1 1.581230743 1.745287633 0.542671379 0.08481895 1 0.12201228 1

DB04537 1 1.72639666 1.75379047 0.753349628 0.1585137 1 0.12201228 1

DB04539 1 1.668893289 1.75379047 0.753349628 0.1585137 1 0.12201228 1

DB04540 1.5 1.807085798 1.753692879 0.367879736 0.245220308 1 0.346299761 1

DB04542 1 3.098998163 1.75379047 0.753349628 0.1585137 1 0.12201228 1

DB04544 2 1.965255364 1.75379047 0.753349628 0.628097886 1 0.645919359 1

DB04549 2 1.924665432 1.75379047 0.753349628 0.628097886 1 0.645919359 1

DB04551 1 2.021258632 1.75379047 0.753349628 0.1585137 1 0.12201228 1

DB04552 1.3333333 1.857868219 1.770629523 0.307115021 0.07724 1 0.257238652 1

DB04556 2 2.367600477 1.75379047 0.753349628 0.628097886 1 0.645919359 1

DB04557 1.75 1.483982892 1.753692879 0.367879736 0.495995374 1 0.495810565 1

DB04559 1 1.663656465 1.75379047 0.753349628 0.1585137 1 0.12201228 1

DB04560 2 2.668347979 1.75379047 0.753349628 0.628097886 1 0.645919359 1

DB04562 2 1.662699828 1.75379047 0.753349628 0.628097886 1 0.645919359 1

DB04563 2 1.884939136 1.75379047 0.753349628 0.628097886 1 0.645919359 1

DB04564 2 2.391479405 1.75379047 0.753349628 0.628097886 1 0.645919359 1

DB04566 2 1.960199562 1.757244872 0.464542708 0.699362524 1 0.645919359 1

DB04573 1 1.486848073 1.757244872 0.464542708 0.051541591 1 0.12201228 1

DB04574 1 1.243964662 1.745287633 0.542671379 0.08481895 1 0.12201228 1

DB04575 1 2.014446143 1.75379047 0.753349628 0.1585137 1 0.12201228 1

DB04576 2 1.9501126 1.75379047 0.753349628 0.628097886 1 0.645919359 1

DB04577 1 1.786523341 1.75379047 0.753349628 0.1585137 1 0.12201228 1

DB04578 1 1.846862991 1.75379047 0.753349628 0.1585137 1 0.12201228 1

DB04579 1 1.418043125 1.75379047 0.753349628 0.1585137 1 0.12201228 1

DB04581 1 1.58426174 1.75379047 0.753349628 0.1585137 1 0.12201228 1

DB04583 2 1.328209056 1.75379047 0.753349628 0.628097886 1 0.645919359 1

DB04585 1 2.143731105 1.75379047 0.753349628 0.1585137 1 0.12201228 1

DB04588 1 1.409370922 1.75379047 0.753349628 0.1585137 1 0.12201228 1

DB04590 2 1.484659394 1.75379047 0.753349628 0.628097886 1 0.645919359 1

DB04591 1 1.484723661 1.75379047 0.753349628 0.1585137 1 0.12201228 1

DB04593 2 2.832056294 1.75379047 0.753349628 0.628097886 1 0.645919359 1

DB04594 2 2.108413959 1.75379047 0.753349628 0.628097886 1 0.645919359 1

DB04599 1.5 1.727523162 1.753692879 0.367879736 0.245220308 1 0.346299761 1

DB04600 2 1.7350828 1.75379047 0.753349628 0.628097886 1 0.645919359 1

DB04601 2 1.038629149 1.75379047 0.753349628 0.628097886 1 0.645919359 1

DB04602 2 1.737597006 1.75379047 0.753349628 0.628097886 1 0.645919359 1

DB04604 1.6666667 1.200885959 1.757244872 0.464542708 0.422702925 1 0.444812957 1

DB04606 2 1.559925489 1.75379047 0.753349628 0.628097886 1 0.645919359 1

DB04607 1 1.312294681 1.75379047 0.753349628 0.1585137 1 0.12201228 1

DB04608 2 2.30260981 1.75379047 0.753349628 0.628097886 1 0.645919359 1

DB04612 1 0.406460804 1.75379047 0.753349628 0.1585137 1 0.12201228 1

DB04614 2 2.106231407 1.75379047 0.753349628 0.628097886 1 0.645919359 1

DB04615 2 2.007880228 1.75379047 0.753349628 0.628097886 1 0.645919359 1

DB04616 2 1.187027393 1.75379047 0.753349628 0.628097886 1 0.645919359 1

DB04617 2 0.672668866 1.75379047 0.753349628 0.628097886 1 0.645919359 1

DB04618 2 1.388057807 1.75379047 0.753349628 0.628097886 1 0.645919359 1

DB04620 2 2.056808643 1.75379047 0.753349628 0.628097886 1 0.645919359 1

DB04622 1 0.615045471 1.75379047 0.753349628 0.1585137 1 0.12201228 1

DB04629 1 1.461956328 1.75379047 0.753349628 0.1585137 1 0.12201228 1

DB04630 1.5 1.801289386 1.745287633 0.542671379 0.325634379 1 0.346299761 1

DB04632 1 2.327306814 1.75379047 0.753349628 0.1585137 1 0.12201228 1

DB04633 2 0.959759295 1.75379047 0.753349628 0.628097886 1 0.645919359 1

DB04636 1 2.619606059 1.75379047 0.753349628 0.1585137 1 0.12201228 1

DB04638 1 1.419458369 1.75379047 0.753349628 0.1585137 1 0.12201228 1

DB04642 2 1.524743376 1.75379047 0.753349628 0.628097886 1 0.645919359 1

DB04643 2 1.802073264 1.75379047 0.753349628 0.628097886 1 0.645919359 1

DB04644 2 1.957404544 1.75379047 0.753349628 0.628097886 1 0.645919359 1

DB04645 2 1.600060763 1.75379047 0.753349628 0.628097886 1 0.645919359 1

DB04648 1 2.108409368 1.75379047 0.753349628 0.1585137 1 0.12201228 1

DB04652 1.6666667 2.105776319 1.757244872 0.464542708 0.422702925 1 0.444812957 1

DB04653 2 0.385031328 1.75379047 0.753349628 0.628097886 1 0.645919359 1

DB04654 2 2.516815662 1.745287633 0.542671379 0.680596551 1 0.645919359 1

DB04655 2 2.401864166 1.75379047 0.753349628 0.628097886 1 0.645919359 1

DB04657 1 1.405792444 1.75379047 0.753349628 0.1585137 1 0.12201228 1

DB04659 2 1.870278804 1.75379047 0.753349628 0.628097886 1 0.645919359 1

DB04660 2 1.495792658 1.75379047 0.753349628 0.628097886 1 0.645919359 1

DB04661 1 2.571229074 1.75379047 0.753349628 0.1585137 1 0.12201228 1

DB04662 1 0.671775377 1.75379047 0.753349628 0.1585137 1 0.12201228 1

DB04664 1 2.104806969 1.75379047 0.753349628 0.1585137 1 0.12201228 1

DB04665 2 1.431199078 1.75379047 0.753349628 0.628097886 1 0.645919359 1

DB04669 1 2.325366081 1.75379047 0.753349628 0.1585137 1 0.12201228 1

DB04672 2 1.880042505 1.75379047 0.753349628 0.628097886 1 0.645919359 1

DB04673 2 1.849680316 1.75379047 0.753349628 0.628097886 1 0.645919359 1

DB04674 1 1.886793371 1.745287633 0.542671379 0.08481895 1 0.12201228 1

DB04677 1 1.227937129 1.75379047 0.753349628 0.1585137 1 0.12201228 1

DB04682 2 2.095684045 1.75379047 0.753349628 0.628097886 1 0.645919359 1

DB04683 2 2.616189272 1.75379047 0.753349628 0.628097886 1 0.645919359 1

DB04685 2 0.258056812 1.75379047 0.753349628 0.628097886 1 0.645919359 1

DB04689 1 1.719325715 1.75379047 0.753349628 0.1585137 1 0.12201228 1

DB04690 2 1.698206422 1.75379047 0.753349628 0.628097886 1 0.645919359 1

DB04695 2 1.660900054 1.745287633 0.542671379 0.680596551 1 0.645919359 1

DB04697 1 1.274335752 1.75379047 0.753349628 0.1585137 1 0.12201228 1

DB04700 2 1.836660587 1.75379047 0.753349628 0.628097886 1 0.645919359 1

DB04701 1 0.653878178 1.75379047 0.753349628 0.1585137 1 0.12201228 1

DB04703 1 2.305395911 1.75379047 0.753349628 0.1585137 1 0.12201228 1

DB04707 1.6666667 1.792779142 1.757244872 0.464542708 0.422702925 1 0.444812957 1

DB04709 1 1.84295292 1.75379047 0.753349628 0.1585137 1 0.12201228 1

DB04714 2 1.705189841 1.745287633 0.542671379 0.680596551 1 0.645919359 1

DB04715 1 2.38803335 1.75379047 0.753349628 0.1585137 1 0.12201228 1

DB04716 1 1.726874357 1.757979426 0.337271213 0.012307606 1 0.12201228 1

DB04719 2 2.117107562 1.75379047 0.753349628 0.628097886 1 0.645919359 1

DB04720 2 1.921510411 1.75379047 0.753349628 0.628097886 1 0.645919359 1

DB04721 2 1.917750056 1.75379047 0.753349628 0.628097886 1 0.645919359 1

DB04722 1 0.792906377 1.75379047 0.753349628 0.1585137 1 0.12201228 1

DB04723 2 0.358605526 1.75379047 0.753349628 0.628097886 1 0.645919359 1

DB04724 1 0.631794732 1.75379047 0.753349628 0.1585137 1 0.12201228 1

DB04725 1.3333333 2.104925767 1.757244872 0.464542708 0.180743525 1 0.257238652 1

DB04727 1 1.284964884 1.75379047 0.753349628 0.1585137 1 0.12201228 1

DB04736 2 3.115394009 1.75379047 0.753349628 0.628097886 1 0.645919359 1

DB04738 1 1.59669328 1.75379047 0.753349628 0.1585137 1 0.12201228 1

DB04739 1 1.964425515 1.75379047 0.753349628 0.1585137 1 0.12201228 1

DB04741 2 -0.249569149 1.75379047 0.753349628 0.628097886 1 0.645919359 1

DB04743 1 1.361784665 1.757244872 0.464542708 0.051541591 1 0.12201228 1

DB04751 1.3333333 1.023864791 1.757244872 0.464542708 0.180743525 1 0.257238652 1

DB04752 2 2.558086133 1.75379047 0.753349628 0.628097886 1 0.645919359 1

DB04753 2 0.83017075 1.75379047 0.753349628 0.628097886 1 0.645919359 1

DB04754 2 1.74469286 1.75379047 0.753349628 0.628097886 1 0.645919359 1

DB04757 2 2.476050676 1.75379047 0.753349628 0.628097886 1 0.645919359 1

DB04758 2 1.533012819 1.75379047 0.753349628 0.628097886 1 0.645919359 1

DB04759 2 1.739209801 1.75379047 0.753349628 0.628097886 1 0.645919359 1

DB04760 2 2.821897185 1.75379047 0.753349628 0.628097886 1 0.645919359 1

DB04761 2 1.249361153 1.75379047 0.753349628 0.628097886 1 0.645919359 1

DB04763 2 1.577687022 1.75379047 0.753349628 0.628097886 1 0.645919359 1

DB04764 2 2.073918991 1.75379047 0.753349628 0.628097886 1 0.645919359 1

DB04767 2 1.671146602 1.75379047 0.753349628 0.628097886 1 0.645919359 1

DB04768 2 1.140859887 1.75379047 0.753349628 0.628097886 1 0.645919359 1

DB04769 1 2.064822093 1.75379047 0.753349628 0.1585137 1 0.12201228 1

DB04770 1 0.658047722 1.75379047 0.753349628 0.1585137 1 0.12201228 1

DB04771 1 1.60764566 1.75379047 0.753349628 0.1585137 1 0.12201228 1

DB04772 1 2.658800441 1.75379047 0.753349628 0.1585137 1 0.12201228 1

DB04774 1 2.046294554 1.75379047 0.753349628 0.1585137 1 0.12201228 1

DB04775 1 2.136786869 1.75379047 0.753349628 0.1585137 1 0.12201228 1

DB04776 1 2.147741566 1.75379047 0.753349628 0.1585137 1 0.12201228 1

DB04779 2 1.785085 1.75379047 0.753349628 0.628097886 1 0.645919359 1

DB04781 2 1.869675996 1.75379047 0.753349628 0.628097886 1 0.645919359 1

DB04783 1 1.859566482 1.75379047 0.753349628 0.1585137 1 0.12201228 1

DB04786 1 1.611650235 1.757979426 0.337271213 0.012307606 1 0.12201228 1

DB04787 1 2.422090011 1.75379047 0.753349628 0.1585137 1 0.12201228 1

DB04789 2 1.975156341 1.75379047 0.753349628 0.628097886 1 0.645919359 1

DB04790 2 3.110018787 1.75379047 0.753349628 0.628097886 1 0.645919359 1

DB04791 2 1.171876128 1.75379047 0.753349628 0.628097886 1 0.645919359 1

DB04792 2 0.824368978 1.75379047 0.753349628 0.628097886 1 0.645919359 1

DB04793 2 2.445350167 1.75379047 0.753349628 0.628097886 1 0.645919359 1

DB04794 1 0.600024445 1.75379047 0.753349628 0.1585137 1 0.12201228 1

DB04795 1.5 2.037024392 1.745287633 0.542671379 0.325634379 1 0.346299761 1

DB04796 2 1.667105047 1.75379047 0.753349628 0.628097886 1 0.645919359 1

DB04797 1 1.877111921 1.75379047 0.753349628 0.1585137 1 0.12201228 1

DB04798 2 2.656063046 1.75379047 0.753349628 0.628097886 1 0.645919359 1

DB04799 1.7 1.350444889 1.759693587 0.247672276 0.40477041 1 0.46515008 1

DB04800 1 2.175469262 1.75379047 0.753349628 0.1585137 1 0.12201228 1

DB04801 1 2.161940723 1.75379047 0.753349628 0.1585137 1 0.12201228 1

DB04803 1 1.244709596 1.75379047 0.753349628 0.1585137 1 0.12201228 1

DB04805 2 2.480580241 1.75379047 0.753349628 0.628097886 1 0.645919359 1

DB04817 2 1.430754372 1.75379047 0.753349628 0.628097886 1 0.645919359 1

DB04820 1 1.139885561 1.757244872 0.464542708 0.051541591 1 0.12201228 1

DB04821 1 2.086064062 1.770629523 0.307115021 0.006049327 1 0.12201228 1

DB04824 1 2.343491502 1.757979426 0.337271213 0.012307606 1 0.12201228 1

DB04825 1.5 2.683976089 1.745287633 0.542671379 0.325634379 1 0.346299761 1

DB04828 1 2.72924684 1.75379047 0.753349628 0.1585137 1 0.12201228 1

DB04829 1.3333333 2.007012551 1.757244872 0.464542708 0.180743525 1 0.257238652 1

DB04832 1 1.580490513 1.757244872 0.464542708 0.051541591 1 0.12201228 1

DB04834 1 1.378348796 1.75379047 0.753349628 0.1585137 1 0.12201228 1

DB04835 1 2.30572921 1.75379047 0.753349628 0.1585137 1 0.12201228 1

DB04836 1 1.708789453 1.745287633 0.542671379 0.08481895 1 0.12201228 1

DB04837 1 0.584735102 1.75379047 0.753349628 0.1585137 1 0.12201228 1

DB04838 2 1.416652664 1.745287633 0.542671379 0.680596551 1 0.645919359 1

DB04839 1 1.945736941 1.745287633 0.542671379 0.08481895 1 0.12201228 1

DB04840 1 1.915909746 1.75379047 0.753349628 0.1585137 1 0.12201228 1

DB04841 1.6 1.545265294 1.757979426 0.337271213 0.319747599 1 0.404623142 1

DB04842 1.3333333 1.516385947 1.757244872 0.464542708 0.180743525 1 0.257238652 1

DB04843 1 2.414966207 1.745287633 0.542671379 0.08481895 1 0.12201228 1

DB04844 1 1.451889763 1.75379047 0.753349628 0.1585137 1 0.12201228 1

DB04845 1 1.239952386 1.75379047 0.753349628 0.1585137 1 0.12201228 1

DB04846 1.3333333 1.7636321 1.770629523 0.307115021 0.07724 1 0.257238652 1

DB04847 1.3333333 2.304251593 1.757244872 0.464542708 0.180743525 1 0.257238652 1

DB04849 1 0.286477503 1.75379047 0.753349628 0.1585137 1 0.12201228 1

DB04850 1 1.876412345 1.75379047 0.753349628 0.1585137 1 0.12201228 1

DB04851 1.6666667 1.95723755 1.757244872 0.464542708 0.422702925 1 0.444812957 1

DB04853 1 3.282975395 1.75379047 0.753349628 0.1585137 1 0.12201228 1

DB04854 2 1.087145435 1.75379047 0.753349628 0.628097886 1 0.645919359 1

DB04855 1.5384615 1.52864588 1.752565715 0.14361026 0.067997828 1 0.368388116 1

DB04856 1 2.393584839 1.75379047 0.753349628 0.1585137 1 0.12201228 1

DB04857 1 2.076761965 1.75379047 0.753349628 0.1585137 1 0.12201228 1

DB04859 2 2.548467896 1.75379047 0.753349628 0.628097886 1 0.645919359 1

DB04860 1 1.494055997 1.75379047 0.753349628 0.1585137 1 0.12201228 1

DB04861 1.6666667 2.023338463 1.757244872 0.464542708 0.422702925 1 0.444812957 1

DB04863 1 1.263992581 1.745287633 0.542671379 0.08481895 1 0.12201228 1

DB04864 2 2.013362387 1.75379047 0.753349628 0.628097886 1 0.645919359 1

DB04865 2 2.350359632 1.75379047 0.753349628 0.628097886 1 0.645919359 1

DB04866 1 2.045582145 1.745287633 0.542671379 0.08481895 1 0.12201228 1

DB04867 1 0.622060926 1.75379047 0.753349628 0.1585137 1 0.12201228 1

DB04868 1 1.263532643 1.745287633 0.542671379 0.08481895 1 0.12201228 1

DB04869 2 1.366207692 1.75379047 0.753349628 0.628097886 1 0.645919359 1

DB04871 1 0.225674071 1.75379047 0.753349628 0.1585137 1 0.12201228 1

DB04872 1 2.663987111 1.75379047 0.753349628 0.1585137 1 0.12201228 1

DB04873 2 1.793707199 1.75379047 0.753349628 0.628097886 1 0.645919359 1

DB04875 1 1.041971776 1.75379047 0.753349628 0.1585137 1 0.12201228 1

DB04876 1 0.425939862 1.75379047 0.753349628 0.1585137 1 0.12201228 1

DB04877 1 1.412077472 1.75379047 0.753349628 0.1585137 1 0.12201228 1

DB04878 1 2.994709982 1.75379047 0.753349628 0.1585137 1 0.12201228 1

DB04879 1 1.609344128 1.757244872 0.464542708 0.051541591 1 0.12201228 1

DB04880 1 2.797915504 1.75379047 0.753349628 0.1585137 1 0.12201228 1

DB04881 1 2.771673624 1.75379047 0.753349628 0.1585137 1 0.12201228 1

DB04882 2 3.093687447 1.75379047 0.753349628 0.628097886 1 0.645919359 1

DB04883 1 2.331750191 1.75379047 0.753349628 0.1585137 1 0.12201228 1

DB04884 1 2.214726527 1.757244872 0.464542708 0.051541591 1 0.12201228 1

DB04885 1 1.245219891 1.75379047 0.753349628 0.1585137 1 0.12201228 1

DB04888 1 1.657800322 1.745287633 0.542671379 0.08481895 1 0.12201228 1

DB04889 1 1.78521824 1.757244872 0.464542708 0.051541591 1 0.12201228 1

DB04890 1 1.670715216 1.75379047 0.753349628 0.1585137 1 0.12201228 1

DB04891 2 1.772621306 1.745287633 0.542671379 0.680596551 1 0.645919359 1

DB04892 1.6666667 1.348981435 1.757244872 0.464542708 0.422702925 1 0.444812957 1

DB04893 2 2.107785151 1.745287633 0.542671379 0.680596551 1 0.645919359 1

DB04894 1 2.182644976 1.757244872 0.464542708 0.051541591 1 0.12201228 1

DB04895 2 2.295127944 1.75379047 0.753349628 0.628097886 1 0.645919359 1

DB04896 1.6666667 1.657666949 1.753207263 0.264393406 0.371713916 1 0.444812957 1

DB04898 1 1.825183872 1.75379047 0.753349628 0.1585137 1 0.12201228 1

DB04899 1 1.236340825 1.757244872 0.464542708 0.051541591 1 0.12201228 1

DB04901 1 1.745925044 1.75379047 0.753349628 0.1585137 1 0.12201228 1

DB04903 2 2.139286531 1.745287633 0.542671379 0.680596551 1 0.645919359 1

DB04905 1 0.976702143 1.745287633 0.542671379 0.08481895 1 0.12201228 1

DB04908 1 1.071395858 1.757244872 0.464542708 0.051541591 1 0.12201228 1

DB04910 2 2.012146505 1.75379047 0.753349628 0.628097886 1 0.645919359 1

DB04912 1 2.812131105 1.75379047 0.753349628 0.1585137 1 0.12201228 1

DB04915 2 1.417801891 1.75379047 0.753349628 0.628097886 1 0.645919359 1

DB04917 1.5 1.527706743 1.745287633 0.542671379 0.325634379 1 0.346299761 1

DB04919 1 2.349294413 1.75379047 0.753349628 0.1585137 1 0.12201228 1

DB04920 1.5 1.768242871 1.753692879 0.367879736 0.245220308 1 0.346299761 1

DB04924 1.5 0.957431 1.745287633 0.542671379 0.325634379 1 0.346299761 1

DB04925 1 1.625052689 1.75379047 0.753349628 0.1585137 1 0.12201228 1

DB04926 2 1.918149278 1.75379047 0.753349628 0.628097886 1 0.645919359 1

DB04930 1.3333333 1.861463897 1.757244872 0.464542708 0.180743525 1 0.257238652 1

DB04931 1 3.008183833 1.75379047 0.753349628 0.1585137 1 0.12201228 1

DB04932 1 1.854257488 1.757244872 0.464542708 0.051541591 1 0.12201228 1

DB04933 1 2.207919357 1.75379047 0.753349628 0.1585137 1 0.12201228 1

DB04938 1 1.88581158 1.75379047 0.753349628 0.1585137 1 0.12201228 1

DB04941 1.5 2.315974597 1.745287633 0.542671379 0.325634379 1 0.346299761 1

DB04942 1.5 1.739273617 1.745287633 0.542671379 0.325634379 1 0.346299761 1

DB04946 1.1818182 1.752988371 1.759747174 0.221112233 0.004477912 1 0.188048345 1

DB04947 1 0.979620463 1.75379047 0.753349628 0.1585137 1 0.12201228 1

DB04948 1 2.332137567 1.770629523 0.307115021 0.006049327 1 0.12201228 1

DB04951 1 2.906044587 1.75379047 0.753349628 0.1585137 1 0.12201228 1

DB04953 2 1.66095036 1.753692879 0.367879736 0.748421796 1 0.645919359 1

DB04954 1 1.983260632 1.75379047 0.753349628 0.1585137 1 0.12201228 1

DB04956 1 0.627772848 1.75379047 0.753349628 0.1585137 1 0.12201228 1

DB04957 1.3333333 1.899555738 1.757244872 0.464542708 0.180743525 1 0.257238652 1

DB04958 1 0.782836231 1.75379047 0.753349628 0.1585137 1 0.12201228 1

DB04959 1 2.280162612 1.745287633 0.542671379 0.08481895 1 0.12201228 1

DB04960 2 1.381728598 1.75379047 0.753349628 0.628097886 1 0.645919359 1

DB04964 1 0.695477128 1.75379047 0.753349628 0.1585137 1 0.12201228 1

DB04967 2 2.159838553 1.757244872 0.464542708 0.699362524 1 0.645919359 1

DB04970 1 2.205914391 1.75379047 0.753349628 0.1585137 1 0.12201228 1

DB04971 1 1.330558939 1.745287633 0.542671379 0.08481895 1 0.12201228 1

DB04972 1 0.134490489 1.75379047 0.753349628 0.1585137 1 0.12201228 1

DB04973 1 1.024427791 1.75379047 0.753349628 0.1585137 1 0.12201228 1

DB04974 1 3.11775527 1.75379047 0.753349628 0.1585137 1 0.12201228 1

DB04975 2 1.313198988 1.75379047 0.753349628 0.628097886 1 0.645919359 1

DB04978 2 1.651070164 1.75379047 0.753349628 0.628097886 1 0.645919359 1

DB04982 2 1.723701985 1.753692879 0.367879736 0.748421796 1 0.645919359 1

DB04983 1 0.415305751 1.75379047 0.753349628 0.1585137 1 0.12201228 1

DB04985 1 2.712378067 1.75379047 0.753349628 0.1585137 1 0.12201228 1

DB04988 1 2.211303057 1.745287633 0.542671379 0.08481895 1 0.12201228 1

DB04991 1 2.251499192 1.75379047 0.753349628 0.1585137 1 0.12201228 1

DB04997 -5.5012582 2.370376057 1.75379047 0.753349628 2.98E-22 1.63E-18 2.78E-29 1.52E-25

DB04998 1 1.614612112 1.75379047 0.753349628 0.1585137 1 0.12201228 1

DB05001 1 2.10070823 1.75379047 0.753349628 0.1585137 1 0.12201228 1

DB05003 1.5 1.728703917 1.745287633 0.542671379 0.325634379 1 0.346299761 1

DB05004 1 1.559176471 1.753692879 0.367879736 0.020243381 1 0.12201228 1

DB05009 1 1.43721349 1.75379047 0.753349628 0.1585137 1 0.12201228 1

DB05010 1 1.345441433 1.745287633 0.542671379 0.08481895 1 0.12201228 1

DB05012 1 2.479975568 1.75379047 0.753349628 0.1585137 1 0.12201228 1

DB05013 1 1.869058087 1.745287633 0.542671379 0.08481895 1 0.12201228 1

DB05014 1 2.45927873 1.757979426 0.337271213 0.012307606 1 0.12201228 1

DB05015 1.6363636 1.532712252 1.759747174 0.221112233 0.28841764 1 0.426448428 1

DB05017 1 1.751396017 1.745287633 0.542671379 0.08481895 1 0.12201228 1

DB05018 2 2.510667954 1.75379047 0.753349628 0.628097886 1 0.645919359 1

DB05022 2 1.985788343 1.745287633 0.542671379 0.680596551 1 0.645919359 1

DB05024 2 0.747294836 1.745287633 0.542671379 0.680596551 1 0.645919359 1

DB05025 1 1.833770451 1.75379047 0.753349628 0.1585137 1 0.12201228 1

DB05032 1 2.826476349 1.75379047 0.753349628 0.1585137 1 0.12201228 1

DB05033 1 3.109931065 1.75379047 0.753349628 0.1585137 1 0.12201228 1

DB05034 1 1.243611987 1.75379047 0.753349628 0.1585137 1 0.12201228 1

DB05035 1 2.229926091 1.745287633 0.542671379 0.08481895 1 0.12201228 1

DB05036 1.6666667 2.263264044 1.757244872 0.464542708 0.422702925 1 0.444812957 1

DB05038 1 2.735360983 1.75379047 0.753349628 0.1585137 1 0.12201228 1

DB05039 2 1.888400525 1.75379047 0.753349628 0.628097886 1 0.645919359 1

DB05042 2 1.504012529 1.75379047 0.753349628 0.628097886 1 0.645919359 1

DB05045 1 2.044666982 1.745287633 0.542671379 0.08481895 1 0.12201228 1

DB05046 1 1.587683001 1.745287633 0.542671379 0.08481895 1 0.12201228 1

DB05047 1.8 1.9766537 1.757979426 0.337271213 0.549575873 1 0.52649583 1

DB05049 1 2.259939142 1.75379047 0.753349628 0.1585137 1 0.12201228 1

DB05050 1 2.480288836 1.75379047 0.753349628 0.1585137 1 0.12201228 1

DB05052 1 1.886689811 1.75379047 0.753349628 0.1585137 1 0.12201228 1

DB05053 1 0.676787313 1.75379047 0.753349628 0.1585137 1 0.12201228 1

DB05064 2 2.125947224 1.75379047 0.753349628 0.628097886 1 0.645919359 1

DB05069 1.5 1.707111547 1.745287633 0.542671379 0.325634379 1 0.346299761 1

DB05070 1 1.409347908 1.75379047 0.753349628 0.1585137 1 0.12201228 1

DB05072 1 2.555138918 1.75379047 0.753349628 0.1585137 1 0.12201228 1

DB05075 1 1.673269195 1.753692879 0.367879736 0.020243381 1 0.12201228 1

DB05077 1 2.60724057 1.75379047 0.753349628 0.1585137 1 0.12201228 1

DB05078 1.5 2.567867612 1.745287633 0.542671379 0.325634379 1 0.346299761 1

DB05079 1 2.12100402 1.745287633 0.542671379 0.08481895 1 0.12201228 1

DB05080 1 2.392208491 1.745287633 0.542671379 0.08481895 1 0.12201228 1

DB05084 1 2.054474467 1.75379047 0.753349628 0.1585137 1 0.12201228 1

DB05085 1 0.417358126 1.75379047 0.753349628 0.1585137 1 0.12201228 1

DB05087 2 2.296303993 1.770629523 0.307115021 0.772424541 1 0.645919359 1

DB05088 1 2.817987655 1.75379047 0.753349628 0.1585137 1 0.12201228 1

DB05092 -5.5012582 1.901187488 1.75379047 0.753349628 2.98E-22 1.63E-18 2.78E-29 1.52E-25

DB05095 1 1.282912554 1.75379047 0.753349628 0.1585137 1 0.12201228 1

DB05096 1 1.922046571 1.745287633 0.542671379 0.08481895 1 0.12201228 1

DB05097 -5.1929569 3.035184245 1.75379047 0.753349628 1.47E-20 8.05E-17 5.21E-27 2.85E-23

DB05098 1 2.824199629 1.75379047 0.753349628 0.1585137 1 0.12201228 1

DB05101 1 0.808145417 1.75379047 0.753349628 0.1585137 1 0.12201228 1

DB05104 1 0.674613198 1.75379047 0.753349628 0.1585137 1 0.12201228 1

DB05107 2 2.047790578 1.75379047 0.753349628 0.628097886 1 0.645919359 1

DB05110 1 2.307836164 1.75379047 0.753349628 0.1585137 1 0.12201228 1

DB05111 1 1.667079641 1.75379047 0.753349628 0.1585137 1 0.12201228 1

DB05115 1 0.046178309 1.75379047 0.753349628 0.1585137 1 0.12201228 1

DB05116 1 2.900813879 1.75379047 0.753349628 0.1585137 1 0.12201228 1

DB05119 1 1.801893534 1.75379047 0.753349628 0.1585137 1 0.12201228 1

DB05120 1 2.631845806 1.75379047 0.753349628 0.1585137 1 0.12201228 1

DB05121 1 1.638414395 1.757244872 0.464542708 0.051541591 1 0.12201228 1

DB05122 -1.1670861 1.291728957 1.757244872 0.464542708 1.54E-10 8.39E-07 3.38E-06 0.018494281

DB05125 2 0.839616027 1.75379047 0.753349628 0.628097886 1 0.645919359 1

DB05127 1 1.959628352 1.75379047 0.753349628 0.1585137 1 0.12201228 1

DB05129 2 2.62831297 1.745287633 0.542671379 0.680596551 1 0.645919359 1

DB05130 1 1.11121875 1.75379047 0.753349628 0.1585137 1 0.12201228 1

DB05131 1 2.476400255 1.75379047 0.753349628 0.1585137 1 0.12201228 1

DB05133 1 1.062016179 1.75379047 0.753349628 0.1585137 1 0.12201228 1

DB05134 1 1.945323394 1.745287633 0.542671379 0.08481895 1 0.12201228 1

DB05137 1.6666667 1.852888792 1.757244872 0.464542708 0.422702925 1 0.444812957 1

DB05139 2 0.463013401 1.75379047 0.753349628 0.628097886 1 0.645919359 1

DB05142 1 0.943806848 1.75379047 0.753349628 0.1585137 1 0.12201228 1

DB05146 1 1.365035452 1.753692879 0.367879736 0.020243381 1 0.12201228 1

DB05147 1.6428571 1.900617025 1.773663076 0.187305714 0.242477448 1 0.43037167 1

DB05149 2 2.261249112 1.745287633 0.542671379 0.680596551 1 0.645919359 1

DB05150 1 1.415117457 1.75379047 0.753349628 0.1585137 1 0.12201228 1

DB05152 1 2.973505724 1.75379047 0.753349628 0.1585137 1 0.12201228 1

DB05155 1 1.288700506 1.75379047 0.753349628 0.1585137 1 0.12201228 1

DB05157 1 1.823560005 1.753692879 0.367879736 0.020243381 1 0.12201228 1

DB05159 1 0.440543244 1.75379047 0.753349628 0.1585137 1 0.12201228 1

DB05161 1 1.058488474 1.745287633 0.542671379 0.08481895 1 0.12201228 1

DB05165 1 1.301061787 1.75379047 0.753349628 0.1585137 1 0.12201228 1

DB05166 2 1.907615946 1.75379047 0.753349628 0.628097886 1 0.645919359 1

DB05168 1.3333333 0.75596868 1.757244872 0.464542708 0.180743525 1 0.257238652 1

DB05169 1 1.904160824 1.745287633 0.542671379 0.08481895 1 0.12201228 1

DB05171 2 2.203758984 1.745287633 0.542671379 0.680596551 1 0.645919359 1

DB05177 2 3.297399479 1.75379047 0.753349628 0.628097886 1 0.645919359 1

DB05183 1 1.615248863 1.75379047 0.753349628 0.1585137 1 0.12201228 1

DB05184 1.3333333 1.169092545 1.757244872 0.464542708 0.180743525 1 0.257238652 1

DB05187 1 1.336126023 1.757244872 0.464542708 0.051541591 1 0.12201228 1

DB05188 1 2.959323856 1.75379047 0.753349628 0.1585137 1 0.12201228 1

DB05190 1 0.533693327 1.75379047 0.753349628 0.1585137 1 0.12201228 1

DB05191 1 0.820386649 1.75379047 0.753349628 0.1585137 1 0.12201228 1

DB05192 1 0.961225687 1.75379047 0.753349628 0.1585137 1 0.12201228 1

DB05194 1 1.542681982 1.745287633 0.542671379 0.08481895 1 0.12201228 1

DB05198 1 1.204511488 1.745287633 0.542671379 0.08481895 1 0.12201228 1

DB05199 1 1.505392652 1.75379047 0.753349628 0.1585137 1 0.12201228 1

DB05200 1 1.802571176 1.75379047 0.753349628 0.1585137 1 0.12201228 1

DB05201 1 1.189854656 1.75379047 0.753349628 0.1585137 1 0.12201228 1

DB05202 1 1.8262458 1.745287633 0.542671379 0.08481895 1 0.12201228 1

DB05203 1.5 1.724839232 1.745287633 0.542671379 0.325634379 1 0.346299761 1

DB05204 1 3.116450277 1.75379047 0.753349628 0.1585137 1 0.12201228 1

DB05205 1 2.553121883 1.75379047 0.753349628 0.1585137 1 0.12201228 1

DB05207 1 0.861494027 1.745287633 0.542671379 0.08481895 1 0.12201228 1

DB05210 1.2 1.441541929 1.757979426 0.337271213 0.049023756 1 0.195688273 1

DB05212 1 2.522850964 1.745287633 0.542671379 0.08481895 1 0.12201228 1

DB05214 1 2.864459207 1.75379047 0.753349628 0.1585137 1 0.12201228 1

DB05217 1 1.965223081 1.75379047 0.753349628 0.1585137 1 0.12201228 1

DB05218 1 1.291393585 1.75379047 0.753349628 0.1585137 1 0.12201228 1

DB05219 1 1.834674782 1.757244872 0.464542708 0.051541591 1 0.12201228 1

DB05220 1 2.368819151 1.75379047 0.753349628 0.1585137 1 0.12201228 1

DB05223 1.5 2.035547637 1.753692879 0.367879736 0.245220308 1 0.346299761 1

DB05227 1 2.328602757 1.75379047 0.753349628 0.1585137 1 0.12201228 1

DB05229 2 3.13626988 1.75379047 0.753349628 0.628097886 1 0.645919359 1

DB05232 2 1.495434104 1.757244872 0.464542708 0.699362524 1 0.645919359 1

DB05233 1 2.387530624 1.75379047 0.753349628 0.1585137 1 0.12201228 1

DB05234 1 0.577695407 1.75379047 0.753349628 0.1585137 1 0.12201228 1

DB05235 1 0.884724557 1.745287633 0.542671379 0.08481895 1 0.12201228 1

DB05239 1 2.612709183 1.75379047 0.753349628 0.1585137 1 0.12201228 1

DB05241 -0.2994336 1.94278705 1.757979426 0.337271213 5.30E-10 2.89E-06 0.000774825 1

DB05243 1 2.003460242 1.75379047 0.753349628 0.1585137 1 0.12201228 1

DB05246 2 2.686605907 1.75379047 0.753349628 0.628097886 1 0.645919359 1

DB05249 1 1.21945661 1.75379047 0.753349628 0.1585137 1 0.12201228 1

DB05252 1 1.135196936 1.75379047 0.753349628 0.1585137 1 0.12201228 1

DB05253 1 1.196968813 1.75379047 0.753349628 0.1585137 1 0.12201228 1

DB05254 1 1.140439191 1.745287633 0.542671379 0.08481895 1 0.12201228 1

DB05255 1.5 1.926647128 1.745287633 0.542671379 0.325634379 1 0.346299761 1

DB05258 1 0.704425404 1.75379047 0.753349628 0.1585137 1 0.12201228 1

DB05259 1 -0.010223701 1.75379047 0.753349628 0.1585137 1 0.12201228 1

DB05260 1.75 2.333259174 1.753692879 0.367879736 0.495995374 1 0.495810565 1

DB05262 2 2.085626201 1.75379047 0.753349628 0.628097886 1 0.645919359 1

DB05264 1 1.766377679 1.75379047 0.753349628 0.1585137 1 0.12201228 1

DB05265 2 1.491761717 1.75379047 0.753349628 0.628097886 1 0.645919359 1

DB05266 1 1.18206161 1.753692879 0.367879736 0.020243381 1 0.12201228 1

DB05268 1 2.828270326 1.75379047 0.753349628 0.1585137 1 0.12201228 1

DB05271 1.2857143 1.572519965 1.736866356 0.292454388 0.061458983 1 0.234170029 1

DB05282 2 1.17682391 1.75379047 0.753349628 0.628097886 1 0.645919359 1

DB05284 1 2.467815795 1.75379047 0.753349628 0.1585137 1 0.12201228 1

DB05289 1 0.47903643 1.75379047 0.753349628 0.1585137 1 0.12201228 1

DB05290 1 1.617535182 1.75379047 0.753349628 0.1585137 1 0.12201228 1

DB05294 1.25 1.793176581 1.753692879 0.367879736 0.085471804 1 0.217648936 1

DB05295 2 1.5884125 1.75379047 0.753349628 0.628097886 1 0.645919359 1

DB05297 2 2.72756461 1.75379047 0.753349628 0.628097886 1 0.645919359 1

DB05298 1 2.456528152 1.75379047 0.753349628 0.1585137 1 0.12201228 1

DB05299 1 1.639265574 1.75379047 0.753349628 0.1585137 1 0.12201228 1

DB05301 1 0.363258234 1.745287633 0.542671379 0.08481895 1 0.12201228 1

DB05303 1 1.648285154 1.745287633 0.542671379 0.08481895 1 0.12201228 1

DB05304 1.5 1.249280932 1.745287633 0.542671379 0.325634379 1 0.346299761 1

DB05305 1 1.262116044 1.75379047 0.753349628 0.1585137 1 0.12201228 1

DB05308 1 2.393109305 1.75379047 0.753349628 0.1585137 1 0.12201228 1

DB05309 1 1.897661593 1.75379047 0.753349628 0.1585137 1 0.12201228 1

DB05311 2 1.355162712 1.75379047 0.753349628 0.628097886 1 0.645919359 1

DB05316 1 1.065908646 1.745287633 0.542671379 0.08481895 1 0.12201228 1

DB05317 2 2.230036724 1.745287633 0.542671379 0.680596551 1 0.645919359 1

DB05319 1 2.648551585 1.75379047 0.753349628 0.1585137 1 0.12201228 1

DB05327 2 3.049858508 1.75379047 0.753349628 0.628097886 1 0.645919359 1

DB05332 1 2.533939559 1.75379047 0.753349628 0.1585137 1 0.12201228 1

DB05337 1 1.810730995 1.75379047 0.753349628 0.1585137 1 0.12201228 1

DB05339 1 0.856892292 1.75379047 0.753349628 0.1585137 1 0.12201228 1

DB05343 1 3.03536102 1.75379047 0.753349628 0.1585137 1 0.12201228 1

DB05345 1 1.803416739 1.75379047 0.753349628 0.1585137 1 0.12201228 1

DB05351 2 2.725014619 1.757244872 0.464542708 0.699362524 1 0.645919359 1

DB05361 1.5 2.857679594 1.745287633 0.542671379 0.325634379 1 0.346299761 1

DB05362 2 1.637680501 1.75379047 0.753349628 0.628097886 1 0.645919359 1

DB05364 1 0.201034387 1.75379047 0.753349628 0.1585137 1 0.12201228 1

DB05367 1 1.46840622 1.75379047 0.753349628 0.1585137 1 0.12201228 1

DB05369 2 1.705533926 1.75379047 0.753349628 0.628097886 1 0.645919359 1

DB05374 1 1.627307083 1.75379047 0.753349628 0.1585137 1 0.12201228 1

DB05381 1.25 2.562070968 1.753692879 0.367879736 0.085471804 1 0.217648936 1

DB05382 1 2.337530294 1.75379047 0.753349628 0.1585137 1 0.12201228 1

DB05383 1.6666667 2.65835991 1.757244872 0.464542708 0.422702925 1 0.444812957 1

DB05386 1 2.113988685 1.75379047 0.753349628 0.1585137 1 0.12201228 1

DB05387 1 1.860739618 1.757244872 0.464542708 0.051541591 1 0.12201228 1

DB05389 1.5 2.124769578 1.745287633 0.542671379 0.325634379 1 0.346299761 1

DB05390 1 1.985193202 1.75379047 0.753349628 0.1585137 1 0.12201228 1

DB05391 1 1.427028683 1.75379047 0.753349628 0.1585137 1 0.12201228 1

DB05395 1 1.969756469 1.75379047 0.753349628 0.1585137 1 0.12201228 1

DB05398 1 1.212591516 1.75379047 0.753349628 0.1585137 1 0.12201228 1

DB05399 1 2.081780042 1.75379047 0.753349628 0.1585137 1 0.12201228 1

DB05403 1 2.419942051 1.75379047 0.753349628 0.1585137 1 0.12201228 1

DB05404 1 2.741872013 1.75379047 0.753349628 0.1585137 1 0.12201228 1

DB05407 1 1.612140624 1.75379047 0.753349628 0.1585137 1 0.12201228 1

DB05408 1 1.87274651 1.745287633 0.542671379 0.08481895 1 0.12201228 1

DB05412 1 1.171241522 1.753692879 0.367879736 0.020243381 1 0.12201228 1

DB05413 2 2.455235515 1.75379047 0.753349628 0.628097886 1 0.645919359 1

DB05415 1 2.529352155 1.75379047 0.753349628 0.1585137 1 0.12201228 1

DB05416 1 1.047479936 1.745287633 0.542671379 0.08481895 1 0.12201228 1

DB05417 2 1.615943603 1.75379047 0.753349628 0.628097886 1 0.645919359 1

DB05418 1 2.156829943 1.75379047 0.753349628 0.1585137 1 0.12201228 1

DB05419 2 1.674162025 1.75379047 0.753349628 0.628097886 1 0.645919359 1

DB05420 1 2.930687126 1.75379047 0.753349628 0.1585137 1 0.12201228 1

DB05421 1 0.861178255 1.75379047 0.753349628 0.1585137 1 0.12201228 1

DB05422 1 1.859662545 1.75379047 0.753349628 0.1585137 1 0.12201228 1

DB05423 1 2.009189412 1.75379047 0.753349628 0.1585137 1 0.12201228 1

DB05424 1 1.986154561 1.75379047 0.753349628 0.1585137 1 0.12201228 1

DB05428 1.5 1.869514905 1.745287633 0.542671379 0.325634379 1 0.346299761 1

DB05429 1 1.840601595 1.75379047 0.753349628 0.1585137 1 0.12201228 1

DB05431 2 1.643598091 1.75379047 0.753349628 0.628097886 1 0.645919359 1

DB05432 2 1.33931339 1.75379047 0.753349628 0.628097886 1 0.645919359 1

DB05434 1 0.992874818 1.745287633 0.542671379 0.08481895 1 0.12201228 1

DB05442 1 1.305757321 1.75379047 0.753349628 0.1585137 1 0.12201228 1

DB05443 1 3.166087038 1.75379047 0.753349628 0.1585137 1 0.12201228 1

DB05446 1 0.813432446 1.75379047 0.753349628 0.1585137 1 0.12201228 1

DB05447 1 1.599109138 1.75379047 0.753349628 0.1585137 1 0.12201228 1

DB05448 1 2.864677636 1.75379047 0.753349628 0.1585137 1 0.12201228 1

DB05449 1 1.878879572 1.75379047 0.753349628 0.1585137 1 0.12201228 1

DB05451 1 1.698758708 1.745287633 0.542671379 0.08481895 1 0.12201228 1

DB05452 1 3.059592061 1.745287633 0.542671379 0.08481895 1 0.12201228 1

DB05455 1 1.797337982 1.75379047 0.753349628 0.1585137 1 0.12201228 1

DB05457 1 1.624072629 1.75379047 0.753349628 0.1585137 1 0.12201228 1

DB05458 2 0.893463065 1.745287633 0.542671379 0.680596551 1 0.645919359 1

DB05459 1 0.336401655 1.745287633 0.542671379 0.08481895 1 0.12201228 1

DB05460 1 1.479242049 1.75379047 0.753349628 0.1585137 1 0.12201228 1

DB05461 1 2.111221525 1.757244872 0.464542708 0.051541591 1 0.12201228 1

DB05463 1 1.252537864 1.75379047 0.753349628 0.1585137 1 0.12201228 1

DB05464 1 1.825550027 1.745287633 0.542671379 0.08481895 1 0.12201228 1

DB05465 1.5 1.024338172 1.745287633 0.542671379 0.325634379 1 0.346299761 1

DB05466 1 0.866743675 1.75379047 0.753349628 0.1585137 1 0.12201228 1

DB05467 2 1.061426609 1.75379047 0.753349628 0.628097886 1 0.645919359 1

DB05468 -5.5012582 0.081871892 1.75379047 0.753349628 2.98E-22 1.63E-18 2.78E-29 1.52E-25

DB05469 1 0.844026704 1.757244872 0.464542708 0.051541591 1 0.12201228 1

DB05470 1 1.454309149 1.753692879 0.367879736 0.020243381 1 0.12201228 1

DB05471 1 1.561817506 1.745287633 0.542671379 0.08481895 1 0.12201228 1

DB05472 1.5 2.623787534 1.745287633 0.542671379 0.325634379 1 0.346299761 1

DB05475 1 1.748199237 1.753692879 0.367879736 0.020243381 1 0.12201228 1

DB05476 1 2.070090528 1.75379047 0.753349628 0.1585137 1 0.12201228 1

DB05479 1 0.593518199 1.75379047 0.753349628 0.1585137 1 0.12201228 1

DB05481 1 2.717419497 1.75379047 0.753349628 0.1585137 1 0.12201228 1

DB05482 2 2.053538233 1.75379047 0.753349628 0.628097886 1 0.645919359 1

DB05483 1 2.635981928 1.75379047 0.753349628 0.1585137 1 0.12201228 1

DB05484 1 2.711012723 1.75379047 0.753349628 0.1585137 1 0.12201228 1

DB05487 1 1.058499953 1.757244872 0.464542708 0.051541591 1 0.12201228 1

DB05488 2 0.018967335 1.75379047 0.753349628 0.628097886 1 0.645919359 1

DB05490 1 1.923032553 1.75379047 0.753349628 0.1585137 1 0.12201228 1

DB05492 1 1.763974339 1.763093512 0.211303302 0.000152294 0.823912474 0.12201228 1

DB05494 2 2.612256322 1.75379047 0.753349628 0.628097886 1 0.645919359 1

DB05495 1 1.823451687 1.75379047 0.753349628 0.1585137 1 0.12201228 1

DB05501 1 1.948059569 1.745287633 0.542671379 0.08481895 1 0.12201228 1

DB05506 1 2.560074685 1.75379047 0.753349628 0.1585137 1 0.12201228 1

DB05507 1 1.813599995 1.745287633 0.542671379 0.08481895 1 0.12201228 1

DB05509 1 1.974884157 1.745287633 0.542671379 0.08481895 1 0.12201228 1

DB05513 1 1.392618279 1.745287633 0.542671379 0.08481895 1 0.12201228 1

DB05514 2 2.472430134 1.75379047 0.753349628 0.628097886 1 0.645919359 1

DB05518 1 1.724820507 1.75379047 0.753349628 0.1585137 1 0.12201228 1

DB05521 1 2.078038155 1.75379047 0.753349628 0.1585137 1 0.12201228 1

DB05524 1 1.112453591 1.75379047 0.753349628 0.1585137 1 0.12201228 1

DB05530 1 0.754958097 1.75379047 0.753349628 0.1585137 1 0.12201228 1

DB05532 1 1.963415449 1.75379047 0.753349628 0.1585137 1 0.12201228 1

DB05533 2 1.953473769 1.75379047 0.753349628 0.628097886 1 0.645919359 1

DB05540 2 1.788823401 1.745287633 0.542671379 0.680596551 1 0.645919359 1

DB05541 2 1.642777568 1.763093512 0.211303302 0.868891809 1 0.645919359 1

DB05542 2 2.087170562 1.75379047 0.753349628 0.628097886 1 0.645919359 1

DB05552 -2.2485841 3.092055246 1.745287633 0.542671379 9.22E-14 5.04E-10 3.51E-10 1.92E-06

DB05553 1 2.11544696 1.75379047 0.753349628 0.1585137 1 0.12201228 1

DB05559 1 1.899533581 1.745287633 0.542671379 0.08481895 1 0.12201228 1

DB05562 1 1.991414361 1.75379047 0.753349628 0.1585137 1 0.12201228 1

DB05578 1 1.213488696 1.75379047 0.753349628 0.1585137 1 0.12201228 1

DB05590 2 0.811499648 1.75379047 0.753349628 0.628097886 1 0.645919359 1

DB05595 2 0.903740066 1.75379047 0.753349628 0.628097886 1 0.645919359 1

DB05607 1 0.439376933 1.75379047 0.753349628 0.1585137 1 0.12201228 1

DB05624 1 1.665971854 1.75379047 0.753349628 0.1585137 1 0.12201228 1

DB05630 2 2.789514006 1.75379047 0.753349628 0.628097886 1 0.645919359 1

DB05642 1 2.013949174 1.745287633 0.542671379 0.08481895 1 0.12201228 1

DB05662 1 1.089428423 1.75379047 0.753349628 0.1585137 1 0.12201228 1

DB05667 2 -0.084955391 1.75379047 0.753349628 0.628097886 1 0.645919359 1

DB05668 1 0.270741005 1.75379047 0.753349628 0.1585137 1 0.12201228 1

DB05679 1 1.256138962 1.745287633 0.542671379 0.08481895 1 0.12201228 1

DB05685 1 0.934428809 1.75379047 0.753349628 0.1585137 1 0.12201228 1

DB05687 1 2.220093992 1.745287633 0.542671379 0.08481895 1 0.12201228 1

DB05688 1 1.815381678 1.745287633 0.542671379 0.08481895 1 0.12201228 1

DB05695 1 1.39460873 1.75379047 0.753349628 0.1585137 1 0.12201228 1

DB05706 2 0.986439791 1.75379047 0.753349628 0.628097886 1 0.645919359 1

DB05708 2 0.588421385 1.75379047 0.753349628 0.628097886 1 0.645919359 1

DB05710 2 -0.219562234 1.75379047 0.753349628 0.628097886 1 0.645919359 1

DB05712 1 0.740971821 1.75379047 0.753349628 0.1585137 1 0.12201228 1

DB05713 2 1.781240632 1.75379047 0.753349628 0.628097886 1 0.645919359 1

DB05714 1 2.422012717 1.75379047 0.753349628 0.1585137 1 0.12201228 1

DB05736 2 1.868589048 1.75379047 0.753349628 0.628097886 1 0.645919359 1

DB05737 1 0.29406291 1.745287633 0.542671379 0.08481895 1 0.12201228 1

DB05739 1 1.513146354 1.745287633 0.542671379 0.08481895 1 0.12201228 1

DB05740 1.8333333 2.217167401 1.770629523 0.307115021 0.580889804 1 0.546876824 1

DB05744 1 2.387225224 1.753692879 0.367879736 0.020243381 1 0.12201228 1

DB05749 1 1.890929219 1.75379047 0.753349628 0.1585137 1 0.12201228 1

DB05750 1 0.60725177 1.75379047 0.753349628 0.1585137 1 0.12201228 1

DB05752 1 1.547578834 1.757979426 0.337271213 0.012307606 1 0.12201228 1

DB05758 1 2.186625203 1.75379047 0.753349628 0.1585137 1 0.12201228 1

DB05766 1 1.885010919 1.757244872 0.464542708 0.051541591 1 0.12201228 1

DB05767 1 1.392835419 1.757979426 0.337271213 0.012307606 1 0.12201228 1

DB05771 1 1.02350561 1.75379047 0.753349628 0.1585137 1 0.12201228 1

DB05773 1 2.025507171 1.75379047 0.753349628 0.1585137 1 0.12201228 1

DB05777 1 1.357390233 1.745287633 0.542671379 0.08481895 1 0.12201228 1

DB05785 1.5 1.976403338 1.753692879 0.367879736 0.245220308 1 0.346299761 1

DB05786 2 1.509349525 1.75379047 0.753349628 0.628097886 1 0.645919359 1

DB05787 1 1.78364884 1.75379047 0.753349628 0.1585137 1 0.12201228 1

DB05790 1 2.035021494 1.75379047 0.753349628 0.1585137 1 0.12201228 1

DB05793 1 1.966352043 1.75379047 0.753349628 0.1585137 1 0.12201228 1

DB05796 1 1.980855964 1.75379047 0.753349628 0.1585137 1 0.12201228 1

DB05797 1 1.437333984 1.75379047 0.753349628 0.1585137 1 0.12201228 1

DB05798 1.5 1.638485418 1.745287633 0.542671379 0.325634379 1 0.346299761 1

DB05801 2 0.77280134 1.75379047 0.753349628 0.628097886 1 0.645919359 1

DB05806 2 1.72397681 1.75379047 0.753349628 0.628097886 1 0.645919359 1

DB05812 2 1.398675511 1.75379047 0.753349628 0.628097886 1 0.645919359 1

DB05814 2 2.516862166 1.75379047 0.753349628 0.628097886 1 0.645919359 1

DB05824 2 3.654138529 1.75379047 0.753349628 0.628097886 1 0.645919359 1

DB05829 1.5 1.261595735 1.745287633 0.542671379 0.325634379 1 0.346299761 1

DB05831 1 1.799123232 1.75379047 0.753349628 0.1585137 1 0.12201228 1

DB05846 1 1.878390207 1.75379047 0.753349628 0.1585137 1 0.12201228 1

DB05848 1 1.68874311 1.75379047 0.753349628 0.1585137 1 0.12201228 1

DB05849 2 1.573687726 1.75379047 0.753349628 0.628097886 1 0.645919359 1

DB05854 1 1.837174742 1.75379047 0.753349628 0.1585137 1 0.12201228 1

DB05855 1.5 0.851552278 1.745287633 0.542671379 0.325634379 1 0.346299761 1

DB05864 2 1.437494884 1.75379047 0.753349628 0.628097886 1 0.645919359 1

DB05869 1 2.232191599 1.745287633 0.542671379 0.08481895 1 0.12201228 1

DB05879 1 2.84830059 1.75379047 0.753349628 0.1585137 1 0.12201228 1

DB05882 1 1.718915036 1.745287633 0.542671379 0.08481895 1 0.12201228 1

DB05883 1 1.120742101 1.75379047 0.753349628 0.1585137 1 0.12201228 1

DB05885 2 1.463390369 1.753692879 0.367879736 0.748421796 1 0.645919359 1

DB05889 1 0.935192106 1.75379047 0.753349628 0.1585137 1 0.12201228 1

DB05892 1 2.255224009 1.75379047 0.753349628 0.1585137 1 0.12201228 1

DB05895 1 2.358331096 1.75379047 0.753349628 0.1585137 1 0.12201228 1

DB05905 2 0.328459991 1.75379047 0.753349628 0.628097886 1 0.645919359 1

DB05906 1 3.290241575 1.75379047 0.753349628 0.1585137 1 0.12201228 1

DB05913 1 1.277120891 1.745287633 0.542671379 0.08481895 1 0.12201228 1

DB05915 2 1.14475762 1.75379047 0.753349628 0.628097886 1 0.645919359 1

DB05916 1 1.444797925 1.75379047 0.753349628 0.1585137 1 0.12201228 1

DB05920 2 0.980386455 1.75379047 0.753349628 0.628097886 1 0.645919359 1

DB05928 1 0.61172873 1.75379047 0.753349628 0.1585137 1 0.12201228 1

DB05930 1 0.849307313 1.75379047 0.753349628 0.1585137 1 0.12201228 1

DB05931 1 3.186761537 1.75379047 0.753349628 0.1585137 1 0.12201228 1

DB05932 1 0.78836623 1.745287633 0.542671379 0.08481895 1 0.12201228 1

DB05934 1 1.666379805 1.75379047 0.753349628 0.1585137 1 0.12201228 1

DB05936 1 2.367407659 1.75379047 0.753349628 0.1585137 1 0.12201228 1

DB05938 1 0.576442929 1.75379047 0.753349628 0.1585137 1 0.12201228 1

DB05939 1 2.098330098 1.75379047 0.753349628 0.1585137 1 0.12201228 1

DB05941 1 1.150908353 1.75379047 0.753349628 0.1585137 1 0.12201228 1

DB05943 1 1.813811083 1.745287633 0.542671379 0.08481895 1 0.12201228 1

DB05944 1 1.721368769 1.745287633 0.542671379 0.08481895 1 0.12201228 1

DB05945 2 0.724748415 1.745287633 0.542671379 0.680596551 1 0.645919359 1

DB05950 2 0.852234688 1.75379047 0.753349628 0.628097886 1 0.645919359 1

DB05956 2 2.031271842 1.745287633 0.542671379 0.680596551 1 0.645919359 1

DB05959 1 1.900512622 1.757244872 0.464542708 0.051541591 1 0.12201228 1

DB05961 1.3333333 1.892757544 1.757244872 0.464542708 0.180743525 1 0.257238652 1

DB05964 1 2.30256389 1.757244872 0.464542708 0.051541591 1 0.12201228 1

DB05966 1 1.742387211 1.745287633 0.542671379 0.08481895 1 0.12201228 1

DB05967 1 0.222582863 1.75379047 0.753349628 0.1585137 1 0.12201228 1

DB05968 1 1.431455952 1.75379047 0.753349628 0.1585137 1 0.12201228 1

DB05969 2 2.824850223 1.75379047 0.753349628 0.628097886 1 0.645919359 1

DB05971 1 1.846609484 1.75379047 0.753349628 0.1585137 1 0.12201228 1

DB05983 1 2.95379391 1.75379047 0.753349628 0.1585137 1 0.12201228 1

DB05984 1 2.936951939 1.75379047 0.753349628 0.1585137 1 0.12201228 1

DB05990 2 1.992193143 1.75379047 0.753349628 0.628097886 1 0.645919359 1

DB05992 1 0.680569976 1.75379047 0.753349628 0.1585137 1 0.12201228 1

DB05993 2 1.878880228 1.75379047 0.753349628 0.628097886 1 0.645919359 1

DB05996 2 1.521503212 1.75379047 0.753349628 0.628097886 1 0.645919359 1

DB06011 1 2.359948227 1.75379047 0.753349628 0.1585137 1 0.12201228 1

DB06013 2 1.897709314 1.75379047 0.753349628 0.628097886 1 0.645919359 1

DB06016 1 2.079321446 1.770629523 0.307115021 0.006049327 1 0.12201228 1

DB06021 1 2.238453095 1.745287633 0.542671379 0.08481895 1 0.12201228 1

DB06040 2 1.672208265 1.75379047 0.753349628 0.628097886 1 0.645919359 1

DB06042 1.3333333 2.232013401 1.770629523 0.307115021 0.07724 1 0.257238652 1

DB06043 1 1.520179398 1.75379047 0.753349628 0.1585137 1 0.12201228 1

DB06050 1 2.337497089 1.75379047 0.753349628 0.1585137 1 0.12201228 1

DB06061 1 1.774388355 1.75379047 0.753349628 0.1585137 1 0.12201228 1

DB06064 1 2.207088074 1.75379047 0.753349628 0.1585137 1 0.12201228 1

DB06069 2 1.365044786 1.75379047 0.753349628 0.628097886 1 0.645919359 1

DB06070 1 2.700032626 1.745287633 0.542671379 0.08481895 1 0.12201228 1

DB06073 1 1.105831705 1.75379047 0.753349628 0.1585137 1 0.12201228 1

DB06077 1.4 1.886032828 1.757979426 0.337271213 0.14425424 1 0.291413148 1

DB06080 1 1.71947959 1.770629523 0.307115021 0.006049327 1 0.12201228 1

DB06081 1 0.716070608 1.75379047 0.753349628 0.1585137 1 0.12201228 1

DB06082 1 2.334420191 1.75379047 0.753349628 0.1585137 1 0.12201228 1

DB06083 1 2.009378022 1.753692879 0.367879736 0.020243381 1 0.12201228 1

DB06089 1.8 2.128909008 1.757979426 0.337271213 0.549575873 1 0.52649583 1

DB06094 1 2.040068214 1.75379047 0.753349628 0.1585137 1 0.12201228 1

DB06096 1 1.799856814 1.757244872 0.464542708 0.051541591 1 0.12201228 1

DB06097 2 1.357693747 1.75379047 0.753349628 0.628097886 1 0.645919359 1

DB06098 2 2.479794131 1.745287633 0.542671379 0.680596551 1 0.645919359 1

DB06101 1 1.586067129 1.757244872 0.464542708 0.051541591 1 0.12201228 1

DB06103 2 2.979722443 1.745287633 0.542671379 0.680596551 1 0.645919359 1

DB06109 1 1.266323531 1.745287633 0.542671379 0.08481895 1 0.12201228 1

DB06116 1 1.161146596 1.75379047 0.753349628 0.1585137 1 0.12201228 1

DB06124 2 1.255353267 1.75379047 0.753349628 0.628097886 1 0.645919359 1

DB06127 1 -0.165701489 1.75379047 0.753349628 0.1585137 1 0.12201228 1

DB06133 1 3.180869745 1.75379047 0.753349628 0.1585137 1 0.12201228 1

DB06134 1.5 1.770768923 1.745287633 0.542671379 0.325634379 1 0.346299761 1

DB06137 1 1.668233394 1.75379047 0.753349628 0.1585137 1 0.12201228 1

DB06138 1 2.694991862 1.75379047 0.753349628 0.1585137 1 0.12201228 1

DB06140 2 2.027369925 1.75379047 0.753349628 0.628097886 1 0.645919359 1

DB06144 1.125 1.336697856 1.761473636 0.259363203 0.007064127 1 0.165382999 1

DB06148 1.1304348 1.792044601 1.750829326 0.165162414 8.62E-05 0.467121488 0.16747126 1

DB06151 1.6666667 1.913807621 1.753207263 0.264393406 0.371713916 1 0.444812957 1

DB06152 2 1.821516886 1.745287633 0.542671379 0.680596551 1 0.645919359 1

DB06153 1 1.928981807 1.759334798 0.191532594 3.68E-05 0.199524277 0.12201228 1

DB06154 2 2.357625748 1.745287633 0.542671379 0.680596551 1 0.645919359 1

DB06155 1 1.333784198 1.745287633 0.542671379 0.08481895 1 0.12201228 1

DB06156 1 2.130140244 1.757244872 0.464542708 0.051541591 1 0.12201228 1

DB06157 1.5 1.37552519 1.745287633 0.542671379 0.325634379 1 0.346299761 1

DB06159 2 0.769083504 1.75379047 0.753349628 0.628097886 1 0.645919359 1

DB06162 1 2.722798247 1.75379047 0.753349628 0.1585137 1 0.12201228 1

DB06168 1 1.526750294 1.75379047 0.753349628 0.1585137 1 0.12201228 1

DB06174 2 2.689930997 1.75379047 0.753349628 0.628097886 1 0.645919359 1

DB06176 1.4 1.511942247 1.757979426 0.337271213 0.14425424 1 0.291413148 1

DB06184 1 2.864165502 1.75379047 0.753349628 0.1585137 1 0.12201228 1

DB06185 2 0.483940235 1.75379047 0.753349628 0.628097886 1 0.645919359 1

DB06190 1 0.969268451 1.75379047 0.753349628 0.1585137 1 0.12201228 1

DB06191 1 1.564806947 1.75379047 0.753349628 0.1585137 1 0.12201228 1

DB06194 2 1.018706514 1.75379047 0.753349628 0.628097886 1 0.645919359 1

DB06195 1.4285714 2.180684044 1.736866356 0.292454388 0.145903866 1 0.306680831 1

DB06196 1 1.137210674 1.745287633 0.542671379 0.08481895 1 0.12201228 1

DB06199 1 1.599476974 1.75379047 0.753349628 0.1585137 1 0.12201228 1

DB06201 1.5 1.922197908 1.745287633 0.542671379 0.325634379 1 0.346299761 1

DB06202 1 2.407527973 1.757244872 0.464542708 0.051541591 1 0.12201228 1

DB06203 1 2.966871548 1.75379047 0.753349628 0.1585137 1 0.12201228 1

DB06204 1 1.788899623 1.770629523 0.307115021 0.006049327 1 0.12201228 1

DB06207 1 2.095791115 1.757244872 0.464542708 0.051541591 1 0.12201228 1

DB06209 1 1.493772179 1.75379047 0.753349628 0.1585137 1 0.12201228 1

DB06210 1 2.952248117 1.75379047 0.753349628 0.1585137 1 0.12201228 1

DB06212 1 2.638446384 1.75379047 0.753349628 0.1585137 1 0.12201228 1

DB06213 1 1.680722911 1.75379047 0.753349628 0.1585137 1 0.12201228 1

DB06216 1.25 1.935159884 1.758534835 0.16776606 0.001217863 1 0.217648936 1

DB06217 1 1.34496553 1.757244872 0.464542708 0.051541591 1 0.12201228 1

DB06218 2 2.191123292 1.757244872 0.464542708 0.699362524 1 0.645919359 1

DB06226 1 2.0099933 1.75379047 0.753349628 0.1585137 1 0.12201228 1

DB06228 2 1.249602657 1.75379047 0.753349628 0.628097886 1 0.645919359 1

DB06229 1 1.31195833 1.759747174 0.221112233 0.000295158 1 0.12201228 1

DB06230 1 2.401200575 1.757244872 0.464542708 0.051541591 1 0.12201228 1

DB06231 2 3.168148438 1.75379047 0.753349628 0.628097886 1 0.645919359 1

DB06233 1 2.051875228 1.75379047 0.753349628 0.1585137 1 0.12201228 1

DB06237 1 2.878514219 1.75379047 0.753349628 0.1585137 1 0.12201228 1

DB06240 1 1.399279418 1.75379047 0.753349628 0.1585137 1 0.12201228 1

DB06241 1 2.758508274 1.75379047 0.753349628 0.1585137 1 0.12201228 1

DB06243 1 1.486964593 1.75379047 0.753349628 0.1585137 1 0.12201228 1

DB06245 1.25 1.872551397 1.759334798 0.191532594 0.003915635 1 0.217648936 1

DB06246 1.3333333 1.23220025 1.770629523 0.307115021 0.07724 1 0.257238652 1

DB06247 2 2.084812803 1.75379047 0.753349628 0.628097886 1 0.645919359 1

DB06248 1 1.057975514 1.745287633 0.542671379 0.08481895 1 0.12201228 1

DB06249 1 2.616472518 1.745287633 0.542671379 0.08481895 1 0.12201228 1

DB06255 1.3333333 1.302117006 1.757244872 0.464542708 0.180743525 1 0.257238652 1

DB06258 2 3.182853357 1.75379047 0.753349628 0.628097886 1 0.645919359 1

DB06262 1.3 1.880797197 1.759693587 0.247672276 0.031722796 1 0.240967937 1

DB06263 2 1.793711318 1.75379047 0.753349628 0.628097886 1 0.645919359 1

DB06266 1 2.378000485 1.745287633 0.542671379 0.08481895 1 0.12201228 1

DB06267 1 0.17764436 1.75379047 0.753349628 0.1585137 1 0.12201228 1

DB06268 1 1.841699864 1.745287633 0.542671379 0.08481895 1 0.12201228 1

DB06271 1.5 1.927006072 1.745287633 0.542671379 0.325634379 1 0.346299761 1

DB06273 1 1.805112134 1.75379047 0.753349628 0.1585137 1 0.12201228 1

DB06274 1 1.315765266 1.757244872 0.464542708 0.051541591 1 0.12201228 1

DB06281 2 0.911201645 1.75379047 0.753349628 0.628097886 1 0.645919359 1

DB06282 1 1.787702587 1.75379047 0.753349628 0.1585137 1 0.12201228 1

DB06283 2 0.636364168 1.745287633 0.542671379 0.680596551 1 0.645919359 1

DB06285 1 1.447256157 1.75379047 0.753349628 0.1585137 1 0.12201228 1

DB06287 1 2.135231547 1.75379047 0.753349628 0.1585137 1 0.12201228 1

DB06288 1 2.305474849 1.753692879 0.367879736 0.020243381 1 0.12201228 1

DB06292 2 3.05184908 1.75379047 0.753349628 0.628097886 1 0.645919359 1

DB06307 2 1.812406569 1.75379047 0.753349628 0.628097886 1 0.645919359 1

DB06311 1 1.910842738 1.75379047 0.753349628 0.1585137 1 0.12201228 1

DB06314 1 1.923048005 1.75379047 0.753349628 0.1585137 1 0.12201228 1

DB06317 1 2.120947374 1.75379047 0.753349628 0.1585137 1 0.12201228 1

DB06318 1 0.607336313 1.75379047 0.753349628 0.1585137 1 0.12201228 1

DB06321 1 3.039856185 1.75379047 0.753349628 0.1585137 1 0.12201228 1

DB06322 1 2.009356693 1.75379047 0.753349628 0.1585137 1 0.12201228 1

DB06324 1 2.395322647 1.75379047 0.753349628 0.1585137 1 0.12201228 1

DB06325 1 0.580032734 1.75379047 0.753349628 0.1585137 1 0.12201228 1

DB06333 1 1.812431156 1.757244872 0.464542708 0.051541591 1 0.12201228 1

DB06335 1 3.399885781 1.75379047 0.753349628 0.1585137 1 0.12201228 1

DB06342 1 0.970594081 1.75379047 0.753349628 0.1585137 1 0.12201228 1

DB06345 2 2.323537631 1.75379047 0.753349628 0.628097886 1 0.645919359 1

DB06347 1 1.045911914 1.75379047 0.753349628 0.1585137 1 0.12201228 1

DB06350 1 1.920324407 1.75379047 0.753349628 0.1585137 1 0.12201228 1

DB06354 1.5 0.887148714 1.745287633 0.542671379 0.325634379 1 0.346299761 1

DB06356 1 0.665814655 1.75379047 0.753349628 0.1585137 1 0.12201228 1

DB06358 2 1.669058716 1.75379047 0.753349628 0.628097886 1 0.645919359 1

DB06360 1 0.981875073 1.75379047 0.753349628 0.1585137 1 0.12201228 1

DB06362 2 1.371111701 1.745287633 0.542671379 0.680596551 1 0.645919359 1

DB06366 1 0.52598412 1.75379047 0.753349628 0.1585137 1 0.12201228 1

DB06367 2 2.376364847 1.75379047 0.753349628 0.628097886 1 0.645919359 1

DB06371 1 2.683735501 1.75379047 0.753349628 0.1585137 1 0.12201228 1

DB06372 1 2.206164388 1.757244872 0.464542708 0.051541591 1 0.12201228 1

DB06374 1 1.904911142 1.75379047 0.753349628 0.1585137 1 0.12201228 1

DB06376 2 2.591713852 1.75379047 0.753349628 0.628097886 1 0.645919359 1

DB06393 1 1.314276758 1.75379047 0.753349628 0.1585137 1 0.12201228 1

DB06401 1 1.672223281 1.745287633 0.542671379 0.08481895 1 0.12201228 1

DB06403 1 1.569517481 1.745287633 0.542671379 0.08481895 1 0.12201228 1

DB06404 1.7142857 2.434871951 1.736866356 0.292454388 0.4692279 1 0.473897096 1

DB06406 2 1.904105478 1.75379047 0.753349628 0.628097886 1 0.645919359 1

DB06408 2 1.85474912 1.745287633 0.542671379 0.680596551 1 0.645919359 1

DB06409 1 1.657832837 1.757244872 0.464542708 0.051541591 1 0.12201228 1

DB06410 2 2.774069003 1.75379047 0.753349628 0.628097886 1 0.645919359 1

DB06412 1 0.767289528 1.745287633 0.542671379 0.08481895 1 0.12201228 1

DB06413 1 1.273973155 1.75379047 0.753349628 0.1585137 1 0.12201228 1

DB06420 2 1.697810488 1.75379047 0.753349628 0.628097886 1 0.645919359 1

DB06421 2 2.072288024 1.745287633 0.542671379 0.680596551 1 0.645919359 1

DB06422 1.5 1.651126035 1.745287633 0.542671379 0.325634379 1 0.346299761 1

DB06423 1.2 1.654200682 1.757979426 0.337271213 0.049023756 1 0.195688273 1

DB06429 1 3.282298745 1.75379047 0.753349628 0.1585137 1 0.12201228 1

DB06433 1 2.862679647 1.75379047 0.753349628 0.1585137 1 0.12201228 1

DB06436 1 2.03394729 1.75379047 0.753349628 0.1585137 1 0.12201228 1

DB06441 1 1.902368716 1.75379047 0.753349628 0.1585137 1 0.12201228 1

DB06442 2 1.065735191 1.75379047 0.753349628 0.628097886 1 0.645919359 1

DB06444 1 0.898836588 1.75379047 0.753349628 0.1585137 1 0.12201228 1

DB06446 1.25 1.796593404 1.753692879 0.367879736 0.085471804 1 0.217648936 1

DB06447 1 1.503278877 1.75379047 0.753349628 0.1585137 1 0.12201228 1

DB06448 2 1.475165578 1.745287633 0.542671379 0.680596551 1 0.645919359 1

DB06451 1 1.770696048 1.75379047 0.753349628 0.1585137 1 0.12201228 1

DB06454 1 1.154821974 1.757244872 0.464542708 0.051541591 1 0.12201228 1

DB06457 1 1.697051493 1.75379047 0.753349628 0.1585137 1 0.12201228 1

DB06460 1 1.893985037 1.745287633 0.542671379 0.08481895 1 0.12201228 1

DB06468 2 1.295944165 1.75379047 0.753349628 0.628097886 1 0.645919359 1

DB06469 1 0.763865136 1.75379047 0.753349628 0.1585137 1 0.12201228 1

DB06470 2 1.785732791 1.75379047 0.753349628 0.628097886 1 0.645919359 1

DB06471 1 1.55334014 1.75379047 0.753349628 0.1585137 1 0.12201228 1

DB06472 1 1.65978429 1.745287633 0.542671379 0.08481895 1 0.12201228 1

DB06474 2 1.508898751 1.75379047 0.753349628 0.628097886 1 0.645919359 1

DB06475 1 2.043122153 1.75379047 0.753349628 0.1585137 1 0.12201228 1

DB06477 1 1.715706745 1.757244872 0.464542708 0.051541591 1 0.12201228 1

DB06479 1 1.315538194 1.75379047 0.753349628 0.1585137 1 0.12201228 1

DB06480 2 0.092521076 1.75379047 0.753349628 0.628097886 1 0.645919359 1

DB06481 2 1.760663909 1.75379047 0.753349628 0.628097886 1 0.645919359 1

DB06486 1.4285714 1.553736808 1.736866356 0.292454388 0.145903866 1 0.306680831 1

DB06492 1 1.61790088 1.75379047 0.753349628 0.1585137 1 0.12201228 1

DB06494 1 1.227086339 1.75379047 0.753349628 0.1585137 1 0.12201228 1

DB06495 1 0.899853694 1.75379047 0.753349628 0.1585137 1 0.12201228 1

DB06497 1 1.506442748 1.75379047 0.753349628 0.1585137 1 0.12201228 1

DB06503 2 2.07747002 1.75379047 0.753349628 0.628097886 1 0.645919359 1

DB06506 1 1.202821662 1.75379047 0.753349628 0.1585137 1 0.12201228 1

DB06510 1 2.127345375 1.745287633 0.542671379 0.08481895 1 0.12201228 1

DB06511 1 2.32735045 1.75379047 0.753349628 0.1585137 1 0.12201228 1

DB06512 1 1.078084087 1.745287633 0.542671379 0.08481895 1 0.12201228 1

DB06515 1 1.91910854 1.75379047 0.753349628 0.1585137 1 0.12201228 1

DB06518 1 0.349631549 1.75379047 0.753349628 0.1585137 1 0.12201228 1

DB06521 1 1.628742612 1.753692879 0.367879736 0.020243381 1 0.12201228 1

DB06525 2 0.615667867 1.75379047 0.753349628 0.628097886 1 0.645919359 1

DB06530 1 1.378424909 1.745287633 0.542671379 0.08481895 1 0.12201228 1

DB06533 1 1.530369981 1.745287633 0.542671379 0.08481895 1 0.12201228 1

DB06534 1 1.527474283 1.75379047 0.753349628 0.1585137 1 0.12201228 1

DB06536 1 2.813949281 1.745287633 0.542671379 0.08481895 1 0.12201228 1

DB06538 1 1.95330429 1.75379047 0.753349628 0.1585137 1 0.12201228 1

DB06543 1 2.235962359 1.75379047 0.753349628 0.1585137 1 0.12201228 1

DB06548 2 1.799829175 1.745287633 0.542671379 0.680596551 1 0.645919359 1

DB06549 1 2.540935677 1.75379047 0.753349628 0.1585137 1 0.12201228 1

DB06550 1 1.053768675 1.75379047 0.753349628 0.1585137 1 0.12201228 1

DB06552 1.6666667 1.803581464 1.757244872 0.464542708 0.422702925 1 0.444812957 1

DB06558 1 0.863471611 1.745287633 0.542671379 0.08481895 1 0.12201228 1

DB06560 1 1.080022916 1.75379047 0.753349628 0.1585137 1 0.12201228 1

DB06579 2 1.484386451 1.736866356 0.292454388 0.815871351 1 0.645919359 1

DB06584 1 2.577600694 1.75379047 0.753349628 0.1585137 1 0.12201228 1

DB06586 1 1.880868645 1.75379047 0.753349628 0.1585137 1 0.12201228 1

DB06587 1 1.92659119 1.75379047 0.753349628 0.1585137 1 0.12201228 1

DB06589 1.1 2.139630058 1.759693587 0.247672276 0.003865763 1 0.155995064 1

DB06594 1 1.917026995 1.757244872 0.464542708 0.051541591 1 0.12201228 1

DB06595 1 1.903278927 1.770629523 0.307115021 0.006049327 1 0.12201228 1

DB06599 1 1.176528125 1.75379047 0.753349628 0.1585137 1 0.12201228 1

DB06602 1 1.815177478 1.75379047 0.753349628 0.1585137 1 0.12201228 1

DB06603 1.6363636 1.899739256 1.759747174 0.221112233 0.28841764 1 0.426448428 1

DB06605 2 1.443482024 1.75379047 0.753349628 0.628097886 1 0.645919359 1

DB06607 1 1.532576384 1.753692879 0.367879736 0.020243381 1 0.12201228 1

DB06612 1 2.795005296 1.75379047 0.753349628 0.1585137 1 0.12201228 1

DB06616 1.125 1.248400123 1.761473636 0.259363203 0.007064127 1 0.165382999 1

DB06623 1 0.04063608 1.75379047 0.753349628 0.1585137 1 0.12201228 1

DB06626 1 2.655371279 1.757244872 0.464542708 0.051541591 1 0.12201228 1

DB06634 1 2.600068354 1.75379047 0.753349628 0.1585137 1 0.12201228 1

DB06635 2 0.847652249 1.75379047 0.753349628 0.628097886 1 0.645919359 1

DB06637 1.8125 2.041173811 1.759334798 0.191532594 0.609331774 1 0.534149739 1

DB06641 1 2.286473032 1.757244872 0.464542708 0.051541591 1 0.12201228 1

DB06643 1 2.010256932 1.75379047 0.753349628 0.1585137 1 0.12201228 1

DB06650 1 1.317753599 1.75379047 0.753349628 0.1585137 1 0.12201228 1

DB06652 1 0.323018932 1.75379047 0.753349628 0.1585137 1 0.12201228 1

DB06654 1 1.54373207 1.75379047 0.753349628 0.1585137 1 0.12201228 1

DB06655 2 1.934538358 1.75379047 0.753349628 0.628097886 1 0.645919359 1

DB06660 1 2.470908596 1.75379047 0.753349628 0.1585137 1 0.12201228 1

DB06663 1 1.636353304 1.753692879 0.367879736 0.020243381 1 0.12201228 1

DB06670 2 0.329230357 1.75379047 0.753349628 0.628097886 1 0.645919359 1

DB06674 1 3.003523606 1.75379047 0.753349628 0.1585137 1 0.12201228 1

DB06677 1 2.62430195 1.75379047 0.753349628 0.1585137 1 0.12201228 1

DB06678 1 1.575483299 1.736866356 0.292454388 0.005874511 1 0.12201228 1

DB06680 1 0.319341543 1.75379047 0.753349628 0.1585137 1 0.12201228 1

DB06681 1 1.556631293 1.745287633 0.542671379 0.08481895 1 0.12201228 1

DB06684 1 0.674339421 1.745287633 0.542671379 0.08481895 1 0.12201228 1

DB06688 1 2.387945343 1.75379047 0.753349628 0.1585137 1 0.12201228 1

DB06689 2 1.984486379 1.75379047 0.753349628 0.628097886 1 0.645919359 1

DB06691 1 1.243549057 1.75379047 0.753349628 0.1585137 1 0.12201228 1

DB06692 1.75 1.614047676 1.753692879 0.367879736 0.495995374 1 0.495810565 1

DB06693 2 2.224065977 1.75379047 0.753349628 0.628097886 1 0.645919359 1

DB06694 1 1.477334764 1.770629523 0.307115021 0.006049327 1 0.12201228 1

DB06695 1 -0.191031803 1.75379047 0.753349628 0.1585137 1 0.12201228 1

DB06698 1 2.049043912 1.745287633 0.542671379 0.08481895 1 0.12201228 1

DB06699 1 1.198238067 1.75379047 0.753349628 0.1585137 1 0.12201228 1

DB06700 1 1.571232747 1.757244872 0.464542708 0.051541591 1 0.12201228 1

DB06701 1 1.64237084 1.757244872 0.464542708 0.051541591 1 0.12201228 1

DB06702 1 1.042134957 1.757979426 0.337271213 0.012307606 1 0.12201228 1

DB06706 1 0.957208937 1.75379047 0.753349628 0.1585137 1 0.12201228 1

DB06707 1 1.742881966 1.757244872 0.464542708 0.051541591 1 0.12201228 1

DB06709 1 1.242427847 1.75379047 0.753349628 0.1585137 1 0.12201228 1

DB06710 1 1.873313425 1.745287633 0.542671379 0.08481895 1 0.12201228 1

DB06711 1 1.783724767 1.745287633 0.542671379 0.08481895 1 0.12201228 1

DB06712 1.6666667 1.407519035 1.770629523 0.307115021 0.367487831 1 0.444812957 1

DB06713 1 1.048498708 1.757244872 0.464542708 0.051541591 1 0.12201228 1

DB06714 2 1.262016411 1.75379047 0.753349628 0.628097886 1 0.645919359 1

DB06716 2 1.753722975 1.745287633 0.542671379 0.680596551 1 0.645919359 1

DB06718 1 2.011847272 1.75379047 0.753349628 0.1585137 1 0.12201228 1

DB06719 1.5 1.906832083 1.745287633 0.542671379 0.325634379 1 0.346299761 1

DB06720 1 2.079784836 1.75379047 0.753349628 0.1585137 1 0.12201228 1

DB06725 1.5 1.327271021 1.745287633 0.542671379 0.325634379 1 0.346299761 1

DB06726 1.6666667 2.481375651 1.757244872 0.464542708 0.422702925 1 0.444812957 1

DB06728 1.5 3.273676736 1.745287633 0.542671379 0.325634379 1 0.346299761 1

DB06730 1 2.097636153 1.75379047 0.753349628 0.1585137 1 0.12201228 1

DB06732 0.2145493 1.549199021 1.757979426 0.337271213 2.37E-06 0.012875024 0.008797306 1

DB06733 2 1.158589781 1.75379047 0.753349628 0.628097886 1 0.645919359 1

DB06734 2 1.278780651 1.75379047 0.753349628 0.628097886 1 0.645919359 1

DB06736 1.5 2.132683384 1.745287633 0.542671379 0.325634379 1 0.346299761 1

DB06738 1.6 1.675286023 1.759693587 0.247672276 0.259535593 1 0.404623142 1

DB06739 1 2.698116132 1.75379047 0.753349628 0.1585137 1 0.12201228 1

DB06741 1.8571429 1.464835122 1.736866356 0.292454388 0.659561248 1 0.56136264 1

DB06749 1 1.89456913 1.75379047 0.753349628 0.1585137 1 0.12201228 1

DB06750 1 0.531821921 1.75379047 0.753349628 0.1585137 1 0.12201228 1

DB06751 1 1.517289608 1.745287633 0.542671379 0.08481895 1 0.12201228 1

DB06752 2 0.883732175 1.75379047 0.753349628 0.628097886 1 0.645919359 1

DB06754 2 2.349444465 1.75379047 0.753349628 0.628097886 1 0.645919359 1

DB06756 2 2.585523898 1.75379047 0.753349628 0.628097886 1 0.645919359 1

DB06757 1.4 1.656560097 1.757979426 0.337271213 0.14425424 1 0.291413148 1

DB06764 1 1.25586889 1.75379047 0.753349628 0.1585137 1 0.12201228 1

DB06766 1 1.393176263 1.75379047 0.753349628 0.1585137 1 0.12201228 1

DB06772 1.5 2.340213693 1.745287633 0.542671379 0.325634379 1 0.346299761 1

DB06773 1 1.091093105 1.75379047 0.753349628 0.1585137 1 0.12201228 1

DB06774 1.5 2.360657031 1.745287633 0.542671379 0.325634379 1 0.346299761 1

DB06775 2 1.706987222 1.75379047 0.753349628 0.628097886 1 0.645919359 1

DB06777 1.75 2.239291961 1.753692879 0.367879736 0.495995374 1 0.495810565 1

DB06779 1.6666667 1.22771357 1.757244872 0.464542708 0.422702925 1 0.444812957 1

DB06780 2 0.943051963 1.75379047 0.753349628 0.628097886 1 0.645919359 1

DB06781 1 1.464345931 1.75379047 0.753349628 0.1585137 1 0.12201228 1

DB06782 1 2.430836944 1.75379047 0.753349628 0.1585137 1 0.12201228 1

DB06785 1 1.684931655 1.75379047 0.753349628 0.1585137 1 0.12201228 1

DB06786 2 1.306895583 1.75379047 0.753349628 0.628097886 1 0.645919359 1

DB06787 1 2.445214225 1.753692879 0.367879736 0.020243381 1 0.12201228 1

DB06788 1 0.247303486 1.75379047 0.753349628 0.1585137 1 0.12201228 1

DB06789 1 1.682379761 1.75379047 0.753349628 0.1585137 1 0.12201228 1

DB06791 1 1.001438076 1.745287633 0.542671379 0.08481895 1 0.12201228 1

DB06795 2 0.875937651 1.75379047 0.753349628 0.628097886 1 0.645919359 1

DB06800 1 1.635045647 1.745287633 0.542671379 0.08481895 1 0.12201228 1

DB06802 1.5 1.43032172 1.745287633 0.542671379 0.325634379 1 0.346299761 1

DB06809 1 2.489321669 1.75379047 0.753349628 0.1585137 1 0.12201228 1

DB06813 1.5 0.334269884 1.745287633 0.542671379 0.325634379 1 0.346299761 1

DB06814 2 1.422185004 1.75379047 0.753349628 0.628097886 1 0.645919359 1

DB06822 -0.8337527 2.399570636 1.757244872 0.464542708 1.22E-08 6.65E-05 3.34E-05 0.182199783

DB06825 1 1.559170726 1.75379047 0.753349628 0.1585137 1 0.12201228 1

DB06828 2 0.666702789 1.75379047 0.753349628 0.628097886 1 0.645919359 1

DB06829 1 2.471655524 1.75379047 0.753349628 0.1585137 1 0.12201228 1

DB06830 2 2.050844073 1.75379047 0.753349628 0.628097886 1 0.645919359 1

DB06831 -2.2485841 1.809409241 1.745287633 0.542671379 9.22E-14 5.04E-10 3.51E-10 1.92E-06

DB06832 1 1.664899722 1.745287633 0.542671379 0.08481895 1 0.12201228 1

DB06833 2 0.666999031 1.75379047 0.753349628 0.628097886 1 0.645919359 1

DB06834 1 0.341498515 1.75379047 0.753349628 0.1585137 1 0.12201228 1

DB06835 2 1.737529115 1.75379047 0.753349628 0.628097886 1 0.645919359 1

DB06836 1 1.392481814 1.745287633 0.542671379 0.08481895 1 0.12201228 1

DB06837 2 0.646557472 1.75379047 0.753349628 0.628097886 1 0.645919359 1

DB06838 1 2.477373903 1.75379047 0.753349628 0.1585137 1 0.12201228 1

DB06840 2 2.157605282 1.75379047 0.753349628 0.628097886 1 0.645919359 1

DB06841 1 2.302206179 1.75379047 0.753349628 0.1585137 1 0.12201228 1

DB06842 1 0.970999678 1.745287633 0.542671379 0.08481895 1 0.12201228 1

DB06843 1 1.70462688 1.745287633 0.542671379 0.08481895 1 0.12201228 1

DB06844 1 1.039435574 1.745287633 0.542671379 0.08481895 1 0.12201228 1

DB06845 1.5 2.088217906 1.745287633 0.542671379 0.325634379 1 0.346299761 1

DB06848 2 2.620999904 1.75379047 0.753349628 0.628097886 1 0.645919359 1

DB06849 2 2.200928761 1.75379047 0.753349628 0.628097886 1 0.645919359 1

DB06850 1.5 1.08237863 1.745287633 0.542671379 0.325634379 1 0.346299761 1

DB06851 2 1.52949327 1.75379047 0.753349628 0.628097886 1 0.645919359 1

DB06852 2 1.73541309 1.75379047 0.753349628 0.628097886 1 0.645919359 1

DB06853 1.5 1.20731859 1.745287633 0.542671379 0.325634379 1 0.346299761 1

DB06855 1.5 2.214061497 1.745287633 0.542671379 0.325634379 1 0.346299761 1

DB06856 1 1.214813326 1.75379047 0.753349628 0.1585137 1 0.12201228 1

DB06857 1 1.793413514 1.75379047 0.753349628 0.1585137 1 0.12201228 1

DB06858 1.5 1.595446184 1.745287633 0.542671379 0.325634379 1 0.346299761 1

DB06859 1 1.694099093 1.75379047 0.753349628 0.1585137 1 0.12201228 1

DB06861 1 0.931353177 1.75379047 0.753349628 0.1585137 1 0.12201228 1

DB06862 1 1.152679629 1.75379047 0.753349628 0.1585137 1 0.12201228 1

DB06865 1 2.180902351 1.75379047 0.753349628 0.1585137 1 0.12201228 1

DB06866 1 2.544090527 1.75379047 0.753349628 0.1585137 1 0.12201228 1

DB06867 2 2.17965419 1.75379047 0.753349628 0.628097886 1 0.645919359 1

DB06868 1 2.453542478 1.75379047 0.753349628 0.1585137 1 0.12201228 1

DB06869 1 2.190672134 1.75379047 0.753349628 0.1585137 1 0.12201228 1

DB06870 1 1.590696886 1.75379047 0.753349628 0.1585137 1 0.12201228 1

DB06871 1 1.529924681 1.745287633 0.542671379 0.08481895 1 0.12201228 1

DB06875 1 1.841062412 1.745287633 0.542671379 0.08481895 1 0.12201228 1

DB06876 2 1.166889012 1.75379047 0.753349628 0.628097886 1 0.645919359 1

DB06877 1 1.978327988 1.75379047 0.753349628 0.1585137 1 0.12201228 1

DB06878 1 0.681732058 1.75379047 0.753349628 0.1585137 1 0.12201228 1

DB06879 1 3.264969715 1.75379047 0.753349628 0.1585137 1 0.12201228 1

DB06880 1 0.86856349 1.75379047 0.753349628 0.1585137 1 0.12201228 1

DB06882 1 1.729149527 1.745287633 0.542671379 0.08481895 1 0.12201228 1

DB06883 1 2.529204302 1.75379047 0.753349628 0.1585137 1 0.12201228 1

DB06884 1.5 1.739555274 1.745287633 0.542671379 0.325634379 1 0.346299761 1

DB06887 1 2.56891936 1.75379047 0.753349628 0.1585137 1 0.12201228 1

DB06888 1 1.374119385 1.75379047 0.753349628 0.1585137 1 0.12201228 1

DB06891 2 0.539173579 1.75379047 0.753349628 0.628097886 1 0.645919359 1

DB06892 1 1.98120346 1.75379047 0.753349628 0.1585137 1 0.12201228 1

DB06894 1 1.73442381 1.75379047 0.753349628 0.1585137 1 0.12201228 1

DB06896 1 1.785604666 1.75379047 0.753349628 0.1585137 1 0.12201228 1

DB06897 1 1.06516874 1.75379047 0.753349628 0.1585137 1 0.12201228 1

DB06898 1 0.917002073 1.745287633 0.542671379 0.08481895 1 0.12201228 1

DB06899 2 0.684144759 1.75379047 0.753349628 0.628097886 1 0.645919359 1

DB06900 1 2.504197321 1.75379047 0.753349628 0.1585137 1 0.12201228 1

DB06901 1 2.397285117 1.75379047 0.753349628 0.1585137 1 0.12201228 1

DB06902 1 0.597994106 1.75379047 0.753349628 0.1585137 1 0.12201228 1

DB06905 1 3.027767717 1.757244872 0.464542708 0.051541591 1 0.12201228 1

DB06908 1 1.584497247 1.745287633 0.542671379 0.08481895 1 0.12201228 1

DB06909 1 3.092938224 1.75379047 0.753349628 0.1585137 1 0.12201228 1

DB06911 1 1.191387119 1.75379047 0.753349628 0.1585137 1 0.12201228 1

DB06912 1 0.936753832 1.75379047 0.753349628 0.1585137 1 0.12201228 1

DB06914 1 1.561673944 1.75379047 0.753349628 0.1585137 1 0.12201228 1

DB06916 1 1.552157854 1.75379047 0.753349628 0.1585137 1 0.12201228 1

DB06917 2 1.969968759 1.75379047 0.753349628 0.628097886 1 0.645919359 1

DB06918 2 1.817317471 1.75379047 0.753349628 0.628097886 1 0.645919359 1

DB06919 1 0.847757385 1.75379047 0.753349628 0.1585137 1 0.12201228 1

DB06920 2 1.909166145 1.75379047 0.753349628 0.628097886 1 0.645919359 1

DB06921 2 0.504331018 1.75379047 0.753349628 0.628097886 1 0.645919359 1

DB06923 2 0.896844721 1.75379047 0.753349628 0.628097886 1 0.645919359 1

DB06924 2 2.219142985 1.75379047 0.753349628 0.628097886 1 0.645919359 1

DB06925 -6.0684256 1.791749988 1.75379047 0.753349628 1.48E-25 8.11E-22 1.02E-33 5.62E-30

DB06926 1 3.37170193 1.75379047 0.753349628 0.1585137 1 0.12201228 1

DB06927 1 1.12633891 1.757244872 0.464542708 0.051541591 1 0.12201228 1

DB06928 1 1.954991026 1.75379047 0.753349628 0.1585137 1 0.12201228 1

DB06929 1 2.193081066 1.75379047 0.753349628 0.1585137 1 0.12201228 1

DB06930 1 0.715303407 1.75379047 0.753349628 0.1585137 1 0.12201228 1

DB06931 2 2.845732011 1.75379047 0.753349628 0.628097886 1 0.645919359 1

DB06932 1 1.691563829 1.75379047 0.753349628 0.1585137 1 0.12201228 1

DB06936 1 -0.559446241 1.75379047 0.753349628 0.1585137 1 0.12201228 1

DB06937 1 1.601423526 1.75379047 0.753349628 0.1585137 1 0.12201228 1

DB06938 1 1.964611578 1.75379047 0.753349628 0.1585137 1 0.12201228 1

DB06939 1 1.506434937 1.75379047 0.753349628 0.1585137 1 0.12201228 1

DB06940 1 2.16332209 1.75379047 0.753349628 0.1585137 1 0.12201228 1

DB06941 1 1.35972186 1.75379047 0.753349628 0.1585137 1 0.12201228 1

DB06942 1 2.776161355 1.75379047 0.753349628 0.1585137 1 0.12201228 1

DB06943 1 1.285913745 1.75379047 0.753349628 0.1585137 1 0.12201228 1

DB06944 1 2.171342193 1.745287633 0.542671379 0.08481895 1 0.12201228 1

DB06945 2 1.852948331 1.75379047 0.753349628 0.628097886 1 0.645919359 1

DB06947 1 1.390183559 1.75379047 0.753349628 0.1585137 1 0.12201228 1

DB06948 1 2.185415568 1.745287633 0.542671379 0.08481895 1 0.12201228 1

DB06951 2 2.474263503 1.75379047 0.753349628 0.628097886 1 0.645919359 1

DB06953 2 1.900910378 1.745287633 0.542671379 0.680596551 1 0.645919359 1

DB06954 2 1.311134639 1.75379047 0.753349628 0.628097886 1 0.645919359 1

DB06956 1 1.592940316 1.75379047 0.753349628 0.1585137 1 0.12201228 1

DB06957 1 2.188079544 1.75379047 0.753349628 0.1585137 1 0.12201228 1

DB06958 1 1.037662644 1.75379047 0.753349628 0.1585137 1 0.12201228 1

DB06959 1.5 1.388652153 1.745287633 0.542671379 0.325634379 1 0.346299761 1

DB06961 1 0.57066869 1.75379047 0.753349628 0.1585137 1 0.12201228 1

DB06962 2 2.392446213 1.75379047 0.753349628 0.628097886 1 0.645919359 1

DB06963 1 0.071004929 1.75379047 0.753349628 0.1585137 1 0.12201228 1

DB06964 1 1.753785112 1.75379047 0.753349628 0.1585137 1 0.12201228 1

DB06967 2 2.057418766 1.75379047 0.753349628 0.628097886 1 0.645919359 1

DB06969 1 2.627209503 1.75379047 0.753349628 0.1585137 1 0.12201228 1

DB06971 1 1.910703336 1.75379047 0.753349628 0.1585137 1 0.12201228 1

DB06972 1 0.808339653 1.75379047 0.753349628 0.1585137 1 0.12201228 1

DB06973 1 2.313628556 1.75379047 0.753349628 0.1585137 1 0.12201228 1

DB06976 1 1.359353855 1.75379047 0.753349628 0.1585137 1 0.12201228 1

DB06977 1 1.948297207 1.75379047 0.753349628 0.1585137 1 0.12201228 1

DB06979 2 2.238677187 1.75379047 0.753349628 0.628097886 1 0.645919359 1

DB06980 1 0.143241889 1.75379047 0.753349628 0.1585137 1 0.12201228 1

DB06981 1 1.777749186 1.75379047 0.753349628 0.1585137 1 0.12201228 1

DB06982 1 1.64129178 1.75379047 0.753349628 0.1585137 1 0.12201228 1

DB06983 1 2.70358682 1.75379047 0.753349628 0.1585137 1 0.12201228 1

DB06984 2 1.052419839 1.75379047 0.753349628 0.628097886 1 0.645919359 1

DB06986 2 1.601804313 1.75379047 0.753349628 0.628097886 1 0.645919359 1

DB06987 1 1.738741477 1.745287633 0.542671379 0.08481895 1 0.12201228 1

DB06989 1 2.527946415 1.75379047 0.753349628 0.1585137 1 0.12201228 1

DB06990 1 2.207059347 1.75379047 0.753349628 0.1585137 1 0.12201228 1

DB06991 1 2.113357091 1.75379047 0.753349628 0.1585137 1 0.12201228 1

DB06992 2 0.163613095 1.75379047 0.753349628 0.628097886 1 0.645919359 1

DB06993 1 2.151263239 1.75379047 0.753349628 0.1585137 1 0.12201228 1

DB06994 1 1.637135823 1.75379047 0.753349628 0.1585137 1 0.12201228 1

DB06995 1 1.205465705 1.75379047 0.753349628 0.1585137 1 0.12201228 1

DB06996 1 1.277651823 1.75379047 0.753349628 0.1585137 1 0.12201228 1

DB06997 1 1.595755658 1.75379047 0.753349628 0.1585137 1 0.12201228 1

DB07001 1 1.042304167 1.75379047 0.753349628 0.1585137 1 0.12201228 1

DB07002 1 -0.085956686 1.75379047 0.753349628 0.1585137 1 0.12201228 1

DB07003 1 2.055465416 1.75379047 0.753349628 0.1585137 1 0.12201228 1

DB07004 1 0.77662963 1.75379047 0.753349628 0.1585137 1 0.12201228 1

DB07005 1 0.792793549 1.75379047 0.753349628 0.1585137 1 0.12201228 1

DB07006 2 1.748211407 1.75379047 0.753349628 0.628097886 1 0.645919359 1

DB07007 1 0.198061764 1.75379047 0.753349628 0.1585137 1 0.12201228 1

DB07008 1 1.939814058 1.75379047 0.753349628 0.1585137 1 0.12201228 1

DB07009 1 1.167954975 1.745287633 0.542671379 0.08481895 1 0.12201228 1

DB07010 1 2.78355213 1.75379047 0.753349628 0.1585137 1 0.12201228 1

DB07011 1 2.10337912 1.75379047 0.753349628 0.1585137 1 0.12201228 1

DB07013 2 0.750795368 1.75379047 0.753349628 0.628097886 1 0.645919359 1

DB07014 1 1.730526696 1.75379047 0.753349628 0.1585137 1 0.12201228 1

DB07015 1 1.874941105 1.75379047 0.753349628 0.1585137 1 0.12201228 1

DB07016 1 2.806070424 1.75379047 0.753349628 0.1585137 1 0.12201228 1

DB07017 2 1.215684949 1.75379047 0.753349628 0.628097886 1 0.645919359 1

DB07019 1 2.169135025 1.75379047 0.753349628 0.1585137 1 0.12201228 1

DB07020 1 1.960302308 1.75379047 0.753349628 0.1585137 1 0.12201228 1

DB07021 1 1.657506823 1.75379047 0.753349628 0.1585137 1 0.12201228 1

DB07022 2 0.684708819 1.75379047 0.753349628 0.628097886 1 0.645919359 1

DB07023 2 1.835030762 1.75379047 0.753349628 0.628097886 1 0.645919359 1

DB07024 1 2.859726585 1.75379047 0.753349628 0.1585137 1 0.12201228 1

DB07025 2 0.875953116 1.75379047 0.753349628 0.628097886 1 0.645919359 1

DB07026 1 1.651578277 1.75379047 0.753349628 0.1585137 1 0.12201228 1

DB07027 1 1.285802475 1.75379047 0.753349628 0.1585137 1 0.12201228 1

DB07028 2 1.580942076 1.75379047 0.753349628 0.628097886 1 0.645919359 1

DB07029 1 1.613876952 1.75379047 0.753349628 0.1585137 1 0.12201228 1

DB07030 2 1.44380097 1.75379047 0.753349628 0.628097886 1 0.645919359 1

DB07031 2 1.983693484 1.75379047 0.753349628 0.628097886 1 0.645919359 1

DB07032 1 0.540746461 1.75379047 0.753349628 0.1585137 1 0.12201228 1

DB07033 1 0.637814738 1.75379047 0.753349628 0.1585137 1 0.12201228 1

DB07034 2 1.584208723 1.75379047 0.753349628 0.628097886 1 0.645919359 1

DB07036 1 2.023594893 1.75379047 0.753349628 0.1585137 1 0.12201228 1

DB07037 2 2.299767949 1.75379047 0.753349628 0.628097886 1 0.645919359 1

DB07038 2 2.237713497 1.75379047 0.753349628 0.628097886 1 0.645919359 1

DB07039 1 1.121765899 1.75379047 0.753349628 0.1585137 1 0.12201228 1

DB07045 1 1.032378726 1.75379047 0.753349628 0.1585137 1 0.12201228 1

DB07046 1 2.365003929 1.75379047 0.753349628 0.1585137 1 0.12201228 1

DB07048 2 1.604294353 1.75379047 0.753349628 0.628097886 1 0.645919359 1

DB07049 2 3.12131012 1.75379047 0.753349628 0.628097886 1 0.645919359 1

DB07050 2 1.975536605 1.75379047 0.753349628 0.628097886 1 0.645919359 1

DB07051 1 1.831022087 1.75379047 0.753349628 0.1585137 1 0.12201228 1

DB07053 1 2.526606565 1.75379047 0.753349628 0.1585137 1 0.12201228 1

DB07054 1 1.361123189 1.75379047 0.753349628 0.1585137 1 0.12201228 1

DB07056 2 2.170783035 1.75379047 0.753349628 0.628097886 1 0.645919359 1

DB07058 1 1.013786251 1.75379047 0.753349628 0.1585137 1 0.12201228 1

DB07059 2 1.157363701 1.75379047 0.753349628 0.628097886 1 0.645919359 1

DB07060 2 1.91244484 1.75379047 0.753349628 0.628097886 1 0.645919359 1

DB07061 2 3.452901793 1.75379047 0.753349628 0.628097886 1 0.645919359 1

DB07063 2 0.948799416 1.75379047 0.753349628 0.628097886 1 0.645919359 1

DB07064 2 2.629642721 1.75379047 0.753349628 0.628097886 1 0.645919359 1

DB07065 1 -0.362636266 1.75379047 0.753349628 0.1585137 1 0.12201228 1

DB07066 2 1.43373527 1.75379047 0.753349628 0.628097886 1 0.645919359 1

DB07067 1 2.187654118 1.75379047 0.753349628 0.1585137 1 0.12201228 1

DB07068 1 2.642147726 1.75379047 0.753349628 0.1585137 1 0.12201228 1

DB07069 1 1.76418155 1.75379047 0.753349628 0.1585137 1 0.12201228 1

DB07070 1 1.874925981 1.75379047 0.753349628 0.1585137 1 0.12201228 1

DB07071 2 1.522596113 1.75379047 0.753349628 0.628097886 1 0.645919359 1

DB07072 1 1.326594449 1.75379047 0.753349628 0.1585137 1 0.12201228 1

DB07073 1 0.859376228 1.75379047 0.753349628 0.1585137 1 0.12201228 1

DB07074 2 0.799777344 1.75379047 0.753349628 0.628097886 1 0.645919359 1

DB07075 2 1.272331978 1.75379047 0.753349628 0.628097886 1 0.645919359 1

DB07076 1 1.775652065 1.75379047 0.753349628 0.1585137 1 0.12201228 1

DB07077 2 1.970724203 1.75379047 0.753349628 0.628097886 1 0.645919359 1

DB07078 2 2.775602233 1.75379047 0.753349628 0.628097886 1 0.645919359 1

DB07079 1 2.441938588 1.75379047 0.753349628 0.1585137 1 0.12201228 1

DB07080 1.2 2.060759414 1.757979426 0.337271213 0.049023756 1 0.195688273 1

DB07081 1 2.32975738 1.75379047 0.753349628 0.1585137 1 0.12201228 1

DB07082 2 1.69466203 1.75379047 0.753349628 0.628097886 1 0.645919359 1

DB07083 1 1.709765939 1.75379047 0.753349628 0.1585137 1 0.12201228 1

DB07086 1 1.621814634 1.745287633 0.542671379 0.08481895 1 0.12201228 1

DB07087 1 1.730947834 1.745287633 0.542671379 0.08481895 1 0.12201228 1

DB07088 1.5 1.579382868 1.745287633 0.542671379 0.325634379 1 0.346299761 1

DB07089 1 0.994921658 1.75379047 0.753349628 0.1585137 1 0.12201228 1

DB07091 1.5 2.004907998 1.745287633 0.542671379 0.325634379 1 0.346299761 1

DB07092 1 2.133560839 1.75379047 0.753349628 0.1585137 1 0.12201228 1

DB07093 2 0.829921218 1.75379047 0.753349628 0.628097886 1 0.645919359 1

DB07094 2 0.149373038 1.75379047 0.753349628 0.628097886 1 0.645919359 1

DB07095 1.5 1.68845292 1.745287633 0.542671379 0.325634379 1 0.346299761 1

DB07096 2 2.6649866 1.75379047 0.753349628 0.628097886 1 0.645919359 1

DB07099 2 1.338996869 1.75379047 0.753349628 0.628097886 1 0.645919359 1

DB07100 1 0.814325736 1.75379047 0.753349628 0.1585137 1 0.12201228 1

DB07101 1 1.357066631 1.75379047 0.753349628 0.1585137 1 0.12201228 1

DB07102 2 3.027388582 1.75379047 0.753349628 0.628097886 1 0.645919359 1

DB07104 2 2.797884273 1.75379047 0.753349628 0.628097886 1 0.645919359 1

DB07105 1 1.833031891 1.75379047 0.753349628 0.1585137 1 0.12201228 1

DB07106 2 2.681439143 1.75379047 0.753349628 0.628097886 1 0.645919359 1

DB07107 1.5 1.885606837 1.745287633 0.542671379 0.325634379 1 0.346299761 1

DB07108 1 1.486273768 1.75379047 0.753349628 0.1585137 1 0.12201228 1

DB07110 1 1.946009991 1.75379047 0.753349628 0.1585137 1 0.12201228 1

DB07111 1 2.066970942 1.75379047 0.753349628 0.1585137 1 0.12201228 1

DB07112 1 2.304165316 1.75379047 0.753349628 0.1585137 1 0.12201228 1

DB07113 2 0.74286541 1.75379047 0.753349628 0.628097886 1 0.645919359 1

DB07115 2 0.972055928 1.75379047 0.753349628 0.628097886 1 0.645919359 1

DB07116 1 2.556653412 1.75379047 0.753349628 0.1585137 1 0.12201228 1

DB07117 1 1.19855194 1.75379047 0.753349628 0.1585137 1 0.12201228 1

DB07119 1 3.047319073 1.745287633 0.542671379 0.08481895 1 0.12201228 1

DB07120 1 1.453977793 1.75379047 0.753349628 0.1585137 1 0.12201228 1

DB07121 1 2.346463926 1.75379047 0.753349628 0.1585137 1 0.12201228 1

DB07122 1 1.424883447 1.75379047 0.753349628 0.1585137 1 0.12201228 1

DB07124 1 1.960894714 1.75379047 0.753349628 0.1585137 1 0.12201228 1

DB07125 1 2.628193349 1.75379047 0.753349628 0.1585137 1 0.12201228 1

DB07126 1 1.975715264 1.745287633 0.542671379 0.08481895 1 0.12201228 1

DB07127 1 0.91862626 1.75379047 0.753349628 0.1585137 1 0.12201228 1

DB07128 1 2.491713863 1.75379047 0.753349628 0.1585137 1 0.12201228 1

DB07129 1 1.296409535 1.75379047 0.753349628 0.1585137 1 0.12201228 1

DB07130 1 1.494905017 1.75379047 0.753349628 0.1585137 1 0.12201228 1

DB07131 1.5 2.018474741 1.745287633 0.542671379 0.325634379 1 0.346299761 1

DB07132 1 1.980816089 1.75379047 0.753349628 0.1585137 1 0.12201228 1

DB07133 1 2.150751863 1.75379047 0.753349628 0.1585137 1 0.12201228 1

DB07134 1 1.504191866 1.75379047 0.753349628 0.1585137 1 0.12201228 1

DB07135 1 1.97306245 1.75379047 0.753349628 0.1585137 1 0.12201228 1

DB07136 2 1.405640361 1.75379047 0.753349628 0.628097886 1 0.645919359 1

DB07137 1 2.683332168 1.745287633 0.542671379 0.08481895 1 0.12201228 1

DB07138 1 1.708015525 1.75379047 0.753349628 0.1585137 1 0.12201228 1

DB07139 2 1.454212618 1.75379047 0.753349628 0.628097886 1 0.645919359 1

DB07140 2 2.027461088 1.75379047 0.753349628 0.628097886 1 0.645919359 1

DB07141 2 2.992482787 1.75379047 0.753349628 0.628097886 1 0.645919359 1

DB07142 2 1.167278999 1.75379047 0.753349628 0.628097886 1 0.645919359 1

DB07143 1 1.862952096 1.75379047 0.753349628 0.1585137 1 0.12201228 1

DB07144 2 1.411807451 1.75379047 0.753349628 0.628097886 1 0.645919359 1

DB07145 1 0.705444541 1.75379047 0.753349628 0.1585137 1 0.12201228 1

DB07146 -6.0684256 1.215815688 1.75379047 0.753349628 1.48E-25 8.11E-22 1.02E-33 5.62E-30

DB07147 1 1.571127051 1.75379047 0.753349628 0.1585137 1 0.12201228 1

DB07149 1 1.114688565 1.75379047 0.753349628 0.1585137 1 0.12201228 1

DB07150 1 1.244390817 1.745287633 0.542671379 0.08481895 1 0.12201228 1

DB07151 1 2.245755162 1.75379047 0.753349628 0.1585137 1 0.12201228 1

DB07152 2 1.989027612 1.75379047 0.753349628 0.628097886 1 0.645919359 1

DB07154 1 2.300183618 1.75379047 0.753349628 0.1585137 1 0.12201228 1

DB07157 2 1.008696967 1.75379047 0.753349628 0.628097886 1 0.645919359 1

DB07158 2 2.378937332 1.75379047 0.753349628 0.628097886 1 0.645919359 1

DB07159 1 2.351228842 1.75379047 0.753349628 0.1585137 1 0.12201228 1

DB07160 1 2.052251803 1.75379047 0.753349628 0.1585137 1 0.12201228 1

DB07161 1 1.037270175 1.75379047 0.753349628 0.1585137 1 0.12201228 1

DB07162 1 1.902917104 1.75379047 0.753349628 0.1585137 1 0.12201228 1

DB07163 1 1.750965146 1.75379047 0.753349628 0.1585137 1 0.12201228 1

DB07164 1 1.833789709 1.745287633 0.542671379 0.08481895 1 0.12201228 1

DB07165 1 2.321734409 1.75379047 0.753349628 0.1585137 1 0.12201228 1

DB07167 1 1.04814351 1.75379047 0.753349628 0.1585137 1 0.12201228 1

DB07168 2 3.864637913 1.75379047 0.753349628 0.628097886 1 0.645919359 1

DB07171 2 1.172372577 1.75379047 0.753349628 0.628097886 1 0.645919359 1

DB07172 1 1.550387094 1.75379047 0.753349628 0.1585137 1 0.12201228 1

DB07174 2 1.689014011 1.75379047 0.753349628 0.628097886 1 0.645919359 1

DB07175 1 1.650652683 1.75379047 0.753349628 0.1585137 1 0.12201228 1

DB07177 2 1.95243678 1.745287633 0.542671379 0.680596551 1 0.645919359 1

DB07179 1 2.194937684 1.75379047 0.753349628 0.1585137 1 0.12201228 1

DB07180 2 2.551695976 1.75379047 0.753349628 0.628097886 1 0.645919359 1

DB07181 1 2.105753112 1.75379047 0.753349628 0.1585137 1 0.12201228 1

DB07182 2 1.899291685 1.75379047 0.753349628 0.628097886 1 0.645919359 1

DB07183 1 2.705058626 1.75379047 0.753349628 0.1585137 1 0.12201228 1

DB07186 1 2.265624373 1.745287633 0.542671379 0.08481895 1 0.12201228 1

DB07187 2 2.077572403 1.75379047 0.753349628 0.628097886 1 0.645919359 1

DB07189 1 3.024649657 1.75379047 0.753349628 0.1585137 1 0.12201228 1

DB07190 1 1.989829929 1.75379047 0.753349628 0.1585137 1 0.12201228 1

DB07193 1 1.876329339 1.75379047 0.753349628 0.1585137 1 0.12201228 1

DB07194 1 1.795803237 1.75379047 0.753349628 0.1585137 1 0.12201228 1

DB07195 1 1.802897813 1.745287633 0.542671379 0.08481895 1 0.12201228 1

DB07196 2 2.166425065 1.75379047 0.753349628 0.628097886 1 0.645919359 1

DB07197 1 2.119929332 1.75379047 0.753349628 0.1585137 1 0.12201228 1

DB07198 1 1.790455685 1.745287633 0.542671379 0.08481895 1 0.12201228 1

DB07202 1 1.159267749 1.75379047 0.753349628 0.1585137 1 0.12201228 1

DB07203 1 3.466363828 1.745287633 0.542671379 0.08481895 1 0.12201228 1

DB07204 1.5 0.814351701 1.745287633 0.542671379 0.325634379 1 0.346299761 1

DB07205 2 2.699126926 1.75379047 0.753349628 0.628097886 1 0.645919359 1

DB07206 1 3.017709709 1.75379047 0.753349628 0.1585137 1 0.12201228 1

DB07207 1.5 0.824511772 1.745287633 0.542671379 0.325634379 1 0.346299761 1

DB07208 1 2.361047183 1.75379047 0.753349628 0.1585137 1 0.12201228 1

DB07209 1 2.740448013 1.75379047 0.753349628 0.1585137 1 0.12201228 1

DB07210 1 3.449861256 1.75379047 0.753349628 0.1585137 1 0.12201228 1

DB07211 1.5 1.315763671 1.745287633 0.542671379 0.325634379 1 0.346299761 1

DB07212 2 2.012909901 1.75379047 0.753349628 0.628097886 1 0.645919359 1

DB07213 2 0.810423129 1.75379047 0.753349628 0.628097886 1 0.645919359 1

DB07215 1 1.690358709 1.745287633 0.542671379 0.08481895 1 0.12201228 1

DB07216 2 0.858373857 1.745287633 0.542671379 0.680596551 1 0.645919359 1

DB07218 1.5 2.129283061 1.745287633 0.542671379 0.325634379 1 0.346299761 1

DB07219 1 3.207845366 1.75379047 0.753349628 0.1585137 1 0.12201228 1

DB07220 1 2.710998743 1.75379047 0.753349628 0.1585137 1 0.12201228 1

DB07221 2 1.654207962 1.75379047 0.753349628 0.628097886 1 0.645919359 1

DB07223 1 2.542666339 1.75379047 0.753349628 0.1585137 1 0.12201228 1

DB07224 1 1.838657365 1.75379047 0.753349628 0.1585137 1 0.12201228 1

DB07225 1 1.609333345 1.75379047 0.753349628 0.1585137 1 0.12201228 1

DB07226 2 1.646853281 1.75379047 0.753349628 0.628097886 1 0.645919359 1

DB07227 1.6666667 2.158634131 1.757244872 0.464542708 0.422702925 1 0.444812957 1

DB07228 2 2.264910451 1.75379047 0.753349628 0.628097886 1 0.645919359 1

DB07229 2 3.243381609 1.75379047 0.753349628 0.628097886 1 0.645919359 1

DB07230 1 2.317728183 1.745287633 0.542671379 0.08481895 1 0.12201228 1

DB07231 1 1.456959832 1.75379047 0.753349628 0.1585137 1 0.12201228 1

DB07232 2 2.405130464 1.745287633 0.542671379 0.680596551 1 0.645919359 1

DB07233 1 2.147469743 1.75379047 0.753349628 0.1585137 1 0.12201228 1

DB07234 2 2.992199332 1.75379047 0.753349628 0.628097886 1 0.645919359 1

DB07235 1 1.347547523 1.75379047 0.753349628 0.1585137 1 0.12201228 1

DB07236 1 1.076202477 1.745287633 0.542671379 0.08481895 1 0.12201228 1

DB07237 2 1.992218684 1.75379047 0.753349628 0.628097886 1 0.645919359 1

DB07239 1 1.257552518 1.75379047 0.753349628 0.1585137 1 0.12201228 1

DB07242 1 2.055783628 1.745287633 0.542671379 0.08481895 1 0.12201228 1

DB07243 2 1.715222137 1.75379047 0.753349628 0.628097886 1 0.645919359 1

DB07244 1.5 2.146142527 1.745287633 0.542671379 0.325634379 1 0.346299761 1

DB07245 1 2.174488781 1.75379047 0.753349628 0.1585137 1 0.12201228 1

DB07246 1 1.743093898 1.75379047 0.753349628 0.1585137 1 0.12201228 1

DB07247 1.5 1.215840359 1.745287633 0.542671379 0.325634379 1 0.346299761 1

DB07248 1 0.805646127 1.75379047 0.753349628 0.1585137 1 0.12201228 1

DB07249 2 1.672306605 1.75379047 0.753349628 0.628097886 1 0.645919359 1

DB07250 2 2.155004872 1.75379047 0.753349628 0.628097886 1 0.645919359 1

DB07251 2 0.82523555 1.75379047 0.753349628 0.628097886 1 0.645919359 1

DB07252 2 2.370703712 1.75379047 0.753349628 0.628097886 1 0.645919359 1

DB07253 2 1.759338674 1.75379047 0.753349628 0.628097886 1 0.645919359 1

DB07254 2 1.143994107 1.75379047 0.753349628 0.628097886 1 0.645919359 1

DB07255 2 1.230545934 1.75379047 0.753349628 0.628097886 1 0.645919359 1

DB07256 2 2.747920953 1.75379047 0.753349628 0.628097886 1 0.645919359 1

DB07257 2 0.930010554 1.75379047 0.753349628 0.628097886 1 0.645919359 1

DB07258 2 2.11100134 1.75379047 0.753349628 0.628097886 1 0.645919359 1

DB07259 2 1.882079263 1.75379047 0.753349628 0.628097886 1 0.645919359 1

DB07260 2 2.205125119 1.75379047 0.753349628 0.628097886 1 0.645919359 1

DB07261 2 1.898104771 1.75379047 0.753349628 0.628097886 1 0.645919359 1

DB07263 1 2.768357788 1.75379047 0.753349628 0.1585137 1 0.12201228 1

DB07264 1 1.479845408 1.75379047 0.753349628 0.1585137 1 0.12201228 1

DB07265 2 2.477021168 1.75379047 0.753349628 0.628097886 1 0.645919359 1

DB07266 1 0.976199354 1.75379047 0.753349628 0.1585137 1 0.12201228 1

DB07267 2 2.962446532 1.75379047 0.753349628 0.628097886 1 0.645919359 1

DB07268 1 1.872724356 1.75379047 0.753349628 0.1585137 1 0.12201228 1

DB07269 2 2.345570232 1.75379047 0.753349628 0.628097886 1 0.645919359 1

DB07270 1 1.429021313 1.75379047 0.753349628 0.1585137 1 0.12201228 1

DB07271 1 0.708452909 1.75379047 0.753349628 0.1585137 1 0.12201228 1

DB07272 1.5 1.405463907 1.745287633 0.542671379 0.325634379 1 0.346299761 1

DB07274 1 1.479262294 1.75379047 0.753349628 0.1585137 1 0.12201228 1

DB07276 1.5 2.061695287 1.745287633 0.542671379 0.325634379 1 0.346299761 1

DB07277 1.5 1.654824329 1.745287633 0.542671379 0.325634379 1 0.346299761 1

DB07278 1.5 1.862222727 1.745287633 0.542671379 0.325634379 1 0.346299761 1

DB07279 1 1.13070227 1.75379047 0.753349628 0.1585137 1 0.12201228 1

DB07281 1 2.440543832 1.75379047 0.753349628 0.1585137 1 0.12201228 1

DB07284 1 2.105850663 1.75379047 0.753349628 0.1585137 1 0.12201228 1

DB07285 1 2.019181857 1.75379047 0.753349628 0.1585137 1 0.12201228 1

DB07286 1 1.659025723 1.75379047 0.753349628 0.1585137 1 0.12201228 1

DB07288 1 2.654536235 1.75379047 0.753349628 0.1585137 1 0.12201228 1

DB07289 1 0.994322246 1.75379047 0.753349628 0.1585137 1 0.12201228 1

DB07292 2 2.554541229 1.75379047 0.753349628 0.628097886 1 0.645919359 1

DB07294 2 2.250180464 1.75379047 0.753349628 0.628097886 1 0.645919359 1

DB07295 1 2.432790806 1.75379047 0.753349628 0.1585137 1 0.12201228 1

DB07297 -6.0684256 1.233619226 1.75379047 0.753349628 1.48E-25 8.11E-22 1.02E-33 5.62E-30

DB07298 1 2.441860399 1.75379047 0.753349628 0.1585137 1 0.12201228 1

DB07299 2 1.088173865 1.75379047 0.753349628 0.628097886 1 0.645919359 1

DB07300 1 2.332648061 1.75379047 0.753349628 0.1585137 1 0.12201228 1

DB07302 1 1.861840773 1.75379047 0.753349628 0.1585137 1 0.12201228 1

DB07303 1 1.714699171 1.75379047 0.753349628 0.1585137 1 0.12201228 1

DB07306 1 2.331765522 1.75379047 0.753349628 0.1585137 1 0.12201228 1

DB07307 1 1.635530751 1.75379047 0.753349628 0.1585137 1 0.12201228 1

DB07309 2 1.266406841 1.75379047 0.753349628 0.628097886 1 0.645919359 1

DB07310 2 1.793495274 1.75379047 0.753349628 0.628097886 1 0.645919359 1

DB07311 2 1.974111364 1.75379047 0.753349628 0.628097886 1 0.645919359 1

DB07312 1 1.348410696 1.75379047 0.753349628 0.1585137 1 0.12201228 1

DB07313 2 1.552892064 1.75379047 0.753349628 0.628097886 1 0.645919359 1

DB07314 2 1.355312647 1.75379047 0.753349628 0.628097886 1 0.645919359 1

DB07316 2 3.083786655 1.75379047 0.753349628 0.628097886 1 0.645919359 1

DB07317 1 1.238772022 1.75379047 0.753349628 0.1585137 1 0.12201228 1

DB07318 1 1.206985482 1.75379047 0.753349628 0.1585137 1 0.12201228 1

DB07319 1 1.006910435 1.75379047 0.753349628 0.1585137 1 0.12201228 1

DB07320 2 2.61884453 1.75379047 0.753349628 0.628097886 1 0.645919359 1

DB07321 1 2.242942147 1.75379047 0.753349628 0.1585137 1 0.12201228 1

DB07322 2 1.329606982 1.75379047 0.753349628 0.628097886 1 0.645919359 1

DB07323 2 1.637308191 1.75379047 0.753349628 0.628097886 1 0.645919359 1

DB07324 1 3.164045125 1.75379047 0.753349628 0.1585137 1 0.12201228 1

DB07325 1 1.799229364 1.75379047 0.753349628 0.1585137 1 0.12201228 1

DB07326 1 1.379539441 1.75379047 0.753349628 0.1585137 1 0.12201228 1

DB07328 1 1.186600739 1.75379047 0.753349628 0.1585137 1 0.12201228 1

DB07330 2 2.288539514 1.75379047 0.753349628 0.628097886 1 0.645919359 1

DB07333 1 1.358315821 1.75379047 0.753349628 0.1585137 1 0.12201228 1

DB07334 1 2.483807262 1.75379047 0.753349628 0.1585137 1 0.12201228 1

DB07335 1 1.977551802 1.745287633 0.542671379 0.08481895 1 0.12201228 1

DB07336 2 1.709885934 1.75379047 0.753349628 0.628097886 1 0.645919359 1

DB07339 2 1.541217183 1.75379047 0.753349628 0.628097886 1 0.645919359 1

DB07340 1.5 0.931867901 1.745287633 0.542671379 0.325634379 1 0.346299761 1

DB07342 1 1.508545686 1.75379047 0.753349628 0.1585137 1 0.12201228 1

DB07344 1 1.672476151 1.75379047 0.753349628 0.1585137 1 0.12201228 1

DB07345 1 1.772948026 1.75379047 0.753349628 0.1585137 1 0.12201228 1

DB07346 1 3.099046397 1.75379047 0.753349628 0.1585137 1 0.12201228 1

DB07347 1.5 1.160615067 1.745287633 0.542671379 0.325634379 1 0.346299761 1

DB07348 1 2.385837891 1.745287633 0.542671379 0.08481895 1 0.12201228 1

DB07350 2 1.500603706 1.75379047 0.753349628 0.628097886 1 0.645919359 1

DB07351 2 2.052372633 1.75379047 0.753349628 0.628097886 1 0.645919359 1

DB07353 1 1.823571508 1.75379047 0.753349628 0.1585137 1 0.12201228 1

DB07354 2 1.518108096 1.75379047 0.753349628 0.628097886 1 0.645919359 1

DB07356 1 1.520505071 1.75379047 0.753349628 0.1585137 1 0.12201228 1

DB07358 1 0.982869623 1.75379047 0.753349628 0.1585137 1 0.12201228 1

DB07359 1 1.682211333 1.75379047 0.753349628 0.1585137 1 0.12201228 1

DB07360 1 1.506162925 1.75379047 0.753349628 0.1585137 1 0.12201228 1

DB07362 1 0.786989145 1.75379047 0.753349628 0.1585137 1 0.12201228 1

DB07363 2 0.940230773 1.75379047 0.753349628 0.628097886 1 0.645919359 1

DB07364 1.5 0.222753433 1.745287633 0.542671379 0.325634379 1 0.346299761 1

DB07366 1 0.878384617 1.75379047 0.753349628 0.1585137 1 0.12201228 1

DB07368 2 1.502917702 1.75379047 0.753349628 0.628097886 1 0.645919359 1

DB07369 1 2.414471921 1.75379047 0.753349628 0.1585137 1 0.12201228 1

DB07374 2 1.732514125 1.754873752 0.200318376 0.889464136 1 0.645919359 1

DB07376 1.5 2.307945115 1.745287633 0.542671379 0.325634379 1 0.346299761 1

DB07377 2 1.303614698 1.75379047 0.753349628 0.628097886 1 0.645919359 1

DB07379 1 1.812214702 1.75379047 0.753349628 0.1585137 1 0.12201228 1

DB07380 2 1.585160877 1.75379047 0.753349628 0.628097886 1 0.645919359 1

DB07382 2 1.715660971 1.75379047 0.753349628 0.628097886 1 0.645919359 1

DB07383 2 1.545717554 1.75379047 0.753349628 0.628097886 1 0.645919359 1

DB07384 2 1.589503882 1.757244872 0.464542708 0.699362524 1 0.645919359 1

DB07385 1 2.630832957 1.75379047 0.753349628 0.1585137 1 0.12201228 1

DB07387 1 2.158723045 1.75379047 0.753349628 0.1585137 1 0.12201228 1

DB07388 1 2.161307094 1.745287633 0.542671379 0.08481895 1 0.12201228 1

DB07389 1 1.554503648 1.75379047 0.753349628 0.1585137 1 0.12201228 1

DB07390 2 1.604019042 1.75379047 0.753349628 0.628097886 1 0.645919359 1

DB07393 2 1.547107594 1.75379047 0.753349628 0.628097886 1 0.645919359 1

DB07394 2 1.379021187 1.757244872 0.464542708 0.699362524 1 0.645919359 1

DB07397 1 2.23420303 1.75379047 0.753349628 0.1585137 1 0.12201228 1

DB07400 1 2.226937098 1.75379047 0.753349628 0.1585137 1 0.12201228 1

DB07401 1.7 1.848922201 1.759693587 0.247672276 0.40477041 1 0.46515008 1

DB07403 1 2.010132006 1.75379047 0.753349628 0.1585137 1 0.12201228 1

DB07405 1 2.090572916 1.75379047 0.753349628 0.1585137 1 0.12201228 1

DB07406 2 2.142600357 1.75379047 0.753349628 0.628097886 1 0.645919359 1

DB07410 2 1.556263953 1.75379047 0.753349628 0.628097886 1 0.645919359 1

DB07412 1 2.049229668 1.75379047 0.753349628 0.1585137 1 0.12201228 1

DB07415 1 0.89850726 1.75379047 0.753349628 0.1585137 1 0.12201228 1

DB07419 1 2.826886078 1.75379047 0.753349628 0.1585137 1 0.12201228 1

DB07421 1 3.375323166 1.75379047 0.753349628 0.1585137 1 0.12201228 1

DB07422 1 2.186117707 1.75379047 0.753349628 0.1585137 1 0.12201228 1

DB07423 1 2.684636689 1.75379047 0.753349628 0.1585137 1 0.12201228 1

DB07425 1 2.262697638 1.75379047 0.753349628 0.1585137 1 0.12201228 1

DB07427 2 1.663321559 1.745287633 0.542671379 0.680596551 1 0.645919359 1

DB07428 2 1.656105128 1.745287633 0.542671379 0.680596551 1 0.645919359 1

DB07430 2 2.883226189 1.75379047 0.753349628 0.628097886 1 0.645919359 1

DB07431 1.5 1.899506297 1.745287633 0.542671379 0.325634379 1 0.346299761 1

DB07433 2 1.568772664 1.75379047 0.753349628 0.628097886 1 0.645919359 1

DB07435 2 0.680101571 1.75379047 0.753349628 0.628097886 1 0.645919359 1

DB07437 2 1.982192349 1.75379047 0.753349628 0.628097886 1 0.645919359 1

DB07440 1 2.162087959 1.75379047 0.753349628 0.1585137 1 0.12201228 1

DB07443 2 0.271783935 1.75379047 0.753349628 0.628097886 1 0.645919359 1

DB07444 2 2.017600885 1.75379047 0.753349628 0.628097886 1 0.645919359 1

DB07446 1 1.631297368 1.75379047 0.753349628 0.1585137 1 0.12201228 1

DB07447 2 1.653248048 1.75379047 0.753349628 0.628097886 1 0.645919359 1

DB07448 2 2.948059802 1.75379047 0.753349628 0.628097886 1 0.645919359 1

DB07449 1 2.87136046 1.75379047 0.753349628 0.1585137 1 0.12201228 1

DB07450 2 2.702555011 1.75379047 0.753349628 0.628097886 1 0.645919359 1

DB07453 1 0.944019933 1.75379047 0.753349628 0.1585137 1 0.12201228 1

DB07454 1 2.168571804 1.75379047 0.753349628 0.1585137 1 0.12201228 1

DB07455 2 0.957823756 1.75379047 0.753349628 0.628097886 1 0.645919359 1

DB07456 1 0.768844056 1.75379047 0.753349628 0.1585137 1 0.12201228 1

DB07457 1 0.646120899 1.75379047 0.753349628 0.1585137 1 0.12201228 1

DB07458 1 1.961678112 1.75379047 0.753349628 0.1585137 1 0.12201228 1

DB07459 1 0.679622924 1.75379047 0.753349628 0.1585137 1 0.12201228 1

DB07460 1 2.623369941 1.75379047 0.753349628 0.1585137 1 0.12201228 1

DB07461 1 1.603394012 1.75379047 0.753349628 0.1585137 1 0.12201228 1

DB07462 1 0.953448393 1.75379047 0.753349628 0.1585137 1 0.12201228 1

DB07465 1 2.295936502 1.75379047 0.753349628 0.1585137 1 0.12201228 1

DB07467 2 2.348108386 1.75379047 0.753349628 0.628097886 1 0.645919359 1

DB07471 1 1.767081127 1.745287633 0.542671379 0.08481895 1 0.12201228 1

DB07476 2 2.092431885 1.75379047 0.753349628 0.628097886 1 0.645919359 1

DB07477 1 0.274700785 1.75379047 0.753349628 0.1585137 1 0.12201228 1

DB07479 2 1.663642174 1.75379047 0.753349628 0.628097886 1 0.645919359 1

DB07480 1 2.995387989 1.75379047 0.753349628 0.1585137 1 0.12201228 1

DB07482 1 2.401117563 1.75379047 0.753349628 0.1585137 1 0.12201228 1

DB07484 2 3.038630194 1.75379047 0.753349628 0.628097886 1 0.645919359 1

DB07485 1 0.485767467 1.75379047 0.753349628 0.1585137 1 0.12201228 1

DB07486 1 1.825572099 1.75379047 0.753349628 0.1585137 1 0.12201228 1

DB07488 2 2.816589623 1.75379047 0.753349628 0.628097886 1 0.645919359 1

DB07489 2 1.084739853 1.75379047 0.753349628 0.628097886 1 0.645919359 1

DB07491 2 1.30300813 1.75379047 0.753349628 0.628097886 1 0.645919359 1

DB07493 1 2.324720919 1.75379047 0.753349628 0.1585137 1 0.12201228 1

DB07494 2 1.421450449 1.75379047 0.753349628 0.628097886 1 0.645919359 1

DB07495 1 1.580121881 1.75379047 0.753349628 0.1585137 1 0.12201228 1

DB07497 2 2.635854986 1.75379047 0.753349628 0.628097886 1 0.645919359 1

DB07498 2 1.908306282 1.75379047 0.753349628 0.628097886 1 0.645919359 1

DB07500 1 1.502853568 1.75379047 0.753349628 0.1585137 1 0.12201228 1

DB07501 1 2.328468981 1.75379047 0.753349628 0.1585137 1 0.12201228 1

DB07502 1 1.197061309 1.75379047 0.753349628 0.1585137 1 0.12201228 1

DB07503 1 2.923443989 1.75379047 0.753349628 0.1585137 1 0.12201228 1

DB07504 1 1.327273499 1.75379047 0.753349628 0.1585137 1 0.12201228 1

DB07506 2 0.820085414 1.745287633 0.542671379 0.680596551 1 0.645919359 1

DB07508 1 2.769915451 1.75379047 0.753349628 0.1585137 1 0.12201228 1

DB07509 1 2.412781267 1.75379047 0.753349628 0.1585137 1 0.12201228 1

DB07512 1 2.940818656 1.75379047 0.753349628 0.1585137 1 0.12201228 1

DB07513 1 0.815825005 1.75379047 0.753349628 0.1585137 1 0.12201228 1

DB07514 1 1.202796826 1.75379047 0.753349628 0.1585137 1 0.12201228 1

DB07515 1 2.129242089 1.75379047 0.753349628 0.1585137 1 0.12201228 1

DB07519 1 1.695673421 1.75379047 0.753349628 0.1585137 1 0.12201228 1

DB07520 1 1.690575052 1.75379047 0.753349628 0.1585137 1 0.12201228 1

DB07521 1 0.327693094 1.75379047 0.753349628 0.1585137 1 0.12201228 1

DB07522 1 1.511191422 1.75379047 0.753349628 0.1585137 1 0.12201228 1

DB07524 1 0.386378447 1.75379047 0.753349628 0.1585137 1 0.12201228 1

DB07525 1 2.062609875 1.75379047 0.753349628 0.1585137 1 0.12201228 1

DB07527 1 2.23702782 1.75379047 0.753349628 0.1585137 1 0.12201228 1

DB07528 1 2.479821324 1.75379047 0.753349628 0.1585137 1 0.12201228 1

DB07529 1 2.347070798 1.75379047 0.753349628 0.1585137 1 0.12201228 1

DB07530 1.5 2.29254736 1.745287633 0.542671379 0.325634379 1 0.346299761 1

DB07531 1 1.788623684 1.75379047 0.753349628 0.1585137 1 0.12201228 1

DB07533 1 2.708350886 1.745287633 0.542671379 0.08481895 1 0.12201228 1

DB07534 1 2.0796314 1.745287633 0.542671379 0.08481895 1 0.12201228 1

DB07535 1 2.078407919 1.75379047 0.753349628 0.1585137 1 0.12201228 1

DB07537 1 2.623889171 1.75379047 0.753349628 0.1585137 1 0.12201228 1

DB07538 1 2.400454257 1.745287633 0.542671379 0.08481895 1 0.12201228 1

DB07539 1 1.926872485 1.745287633 0.542671379 0.08481895 1 0.12201228 1

DB07540 1 1.984369389 1.75379047 0.753349628 0.1585137 1 0.12201228 1

DB07542 1 2.40967198 1.75379047 0.753349628 0.1585137 1 0.12201228 1

DB07543 2 1.057127691 1.75379047 0.753349628 0.628097886 1 0.645919359 1

DB07544 2 1.546625704 1.75379047 0.753349628 0.628097886 1 0.645919359 1

DB07545 1 2.166328773 1.75379047 0.753349628 0.1585137 1 0.12201228 1

DB07546 2 1.246674811 1.75379047 0.753349628 0.628097886 1 0.645919359 1

DB07548 1 2.910687984 1.75379047 0.753349628 0.1585137 1 0.12201228 1

DB07549 1 2.80915791 1.75379047 0.753349628 0.1585137 1 0.12201228 1

DB07550 1 2.430496648 1.75379047 0.753349628 0.1585137 1 0.12201228 1

DB07555 2 1.60196229 1.75379047 0.753349628 0.628097886 1 0.645919359 1

DB07556 1 0.750576082 1.745287633 0.542671379 0.08481895 1 0.12201228 1

DB07557 1.5 1.395340736 1.753692879 0.367879736 0.245220308 1 0.346299761 1

DB07558 1 1.473677164 1.75379047 0.753349628 0.1585137 1 0.12201228 1

DB07559 2 1.031138497 1.75379047 0.753349628 0.628097886 1 0.645919359 1

DB07561 2 0.903007928 1.75379047 0.753349628 0.628097886 1 0.645919359 1

DB07562 1 2.436576753 1.745287633 0.542671379 0.08481895 1 0.12201228 1

DB07563 2 2.849236633 1.75379047 0.753349628 0.628097886 1 0.645919359 1

DB07567 1 3.252919486 1.75379047 0.753349628 0.1585137 1 0.12201228 1

DB07573 1 0.819764441 1.75379047 0.753349628 0.1585137 1 0.12201228 1

DB07574 2 1.739439893 1.757979426 0.337271213 0.763493223 1 0.645919359 1

DB07577 1 0.985344386 1.75379047 0.753349628 0.1585137 1 0.12201228 1

DB07579 2 1.667547118 1.75379047 0.753349628 0.628097886 1 0.645919359 1

DB07580 2 1.390418838 1.75379047 0.753349628 0.628097886 1 0.645919359 1

DB07583 1.5 1.352848768 1.745287633 0.542671379 0.325634379 1 0.346299761 1

DB07584 1 1.894139539 1.745287633 0.542671379 0.08481895 1 0.12201228 1

DB07585 1 1.31821692 1.745287633 0.542671379 0.08481895 1 0.12201228 1

DB07586 2 2.419431815 1.75379047 0.753349628 0.628097886 1 0.645919359 1

DB07587 1 1.769549591 1.75379047 0.753349628 0.1585137 1 0.12201228 1

DB07589 1 0.229680011 1.75379047 0.753349628 0.1585137 1 0.12201228 1

DB07592 2 1.990515306 1.75379047 0.753349628 0.628097886 1 0.645919359 1

DB07593 2 1.620976622 1.75379047 0.753349628 0.628097886 1 0.645919359 1

DB07594 1 1.139720728 1.745287633 0.542671379 0.08481895 1 0.12201228 1

DB07595 1 1.310686181 1.75379047 0.753349628 0.1585137 1 0.12201228 1

DB07596 2 0.826503496 1.75379047 0.753349628 0.628097886 1 0.645919359 1

DB07597 2 2.816872952 1.75379047 0.753349628 0.628097886 1 0.645919359 1

DB07598 2 2.207513292 1.75379047 0.753349628 0.628097886 1 0.645919359 1

DB07601 1 1.900916273 1.75379047 0.753349628 0.1585137 1 0.12201228 1

DB07602 1 0.843926425 1.75379047 0.753349628 0.1585137 1 0.12201228 1

DB07604 1 1.729292939 1.75379047 0.753349628 0.1585137 1 0.12201228 1

DB07605 2 0.747470526 1.75379047 0.753349628 0.628097886 1 0.645919359 1

DB07606 1 1.836129892 1.75379047 0.753349628 0.1585137 1 0.12201228 1

DB07607 1 1.629825108 1.75379047 0.753349628 0.1585137 1 0.12201228 1

DB07608 1 1.33269495 1.75379047 0.753349628 0.1585137 1 0.12201228 1

DB07609 2 0.149589685 1.75379047 0.753349628 0.628097886 1 0.645919359 1

DB07612 1 2.359395755 1.75379047 0.753349628 0.1585137 1 0.12201228 1

DB07613 1 1.546372926 1.75379047 0.753349628 0.1585137 1 0.12201228 1

DB07614 1 2.473199732 1.75379047 0.753349628 0.1585137 1 0.12201228 1

DB07615 1 1.833943224 1.75379047 0.753349628 0.1585137 1 0.12201228 1

DB07616 1 2.700387084 1.75379047 0.753349628 0.1585137 1 0.12201228 1

DB07617 2 2.42826785 1.75379047 0.753349628 0.628097886 1 0.645919359 1

DB07618 1 0.247513061 1.75379047 0.753349628 0.1585137 1 0.12201228 1

DB07619 2 1.074797682 1.75379047 0.753349628 0.628097886 1 0.645919359 1

DB07621 2 0.928675849 1.75379047 0.753349628 0.628097886 1 0.645919359 1

DB07622 1 0.60913001 1.75379047 0.753349628 0.1585137 1 0.12201228 1

DB07623 2 1.742787862 1.75379047 0.753349628 0.628097886 1 0.645919359 1

DB07624 2 0.623746816 1.75379047 0.753349628 0.628097886 1 0.645919359 1

DB07625 1 1.182140139 1.75379047 0.753349628 0.1585137 1 0.12201228 1

DB07626 1 1.880435306 1.75379047 0.753349628 0.1585137 1 0.12201228 1

DB07627 2 1.279258025 1.75379047 0.753349628 0.628097886 1 0.645919359 1

DB07628 1 1.320519315 1.75379047 0.753349628 0.1585137 1 0.12201228 1

DB07629 2 1.303060257 1.75379047 0.753349628 0.628097886 1 0.645919359 1

DB07630 2 0.948431458 1.75379047 0.753349628 0.628097886 1 0.645919359 1

DB07632 2 1.888278262 1.75379047 0.753349628 0.628097886 1 0.645919359 1

DB07636 1.6 2.167510736 1.757979426 0.337271213 0.319747599 1 0.404623142 1

DB07637 1 1.520008832 1.75379047 0.753349628 0.1585137 1 0.12201228 1

DB07638 1 0.740540888 1.745287633 0.542671379 0.08481895 1 0.12201228 1

DB07639 1 2.101022827 1.75379047 0.753349628 0.1585137 1 0.12201228 1

DB07641 1 1.458041376 1.75379047 0.753349628 0.1585137 1 0.12201228 1

DB07642 2 2.794297324 1.75379047 0.753349628 0.628097886 1 0.645919359 1

DB07643 2 2.392076247 1.75379047 0.753349628 0.628097886 1 0.645919359 1

DB07644 2 2.462652148 1.75379047 0.753349628 0.628097886 1 0.645919359 1

DB07645 2 1.373263454 1.745287633 0.542671379 0.680596551 1 0.645919359 1

DB07646 2 1.613734781 1.75379047 0.753349628 0.628097886 1 0.645919359 1

DB07647 2 2.321613763 1.75379047 0.753349628 0.628097886 1 0.645919359 1

DB07648 2 0.084876973 1.75379047 0.753349628 0.628097886 1 0.645919359 1

DB07651 1 2.264275704 1.75379047 0.753349628 0.1585137 1 0.12201228 1

DB07652 2 2.230988003 1.75379047 0.753349628 0.628097886 1 0.645919359 1

DB07653 2 1.966615678 1.75379047 0.753349628 0.628097886 1 0.645919359 1

DB07654 2 1.875617473 1.75379047 0.753349628 0.628097886 1 0.645919359 1

DB07655 2 0.950224712 1.75379047 0.753349628 0.628097886 1 0.645919359 1

DB07657 1 2.835654346 1.75379047 0.753349628 0.1585137 1 0.12201228 1

DB07658 1 1.917717557 1.75379047 0.753349628 0.1585137 1 0.12201228 1

DB07659 1 1.524226547 1.75379047 0.753349628 0.1585137 1 0.12201228 1

DB07660 1 1.485836831 1.75379047 0.753349628 0.1585137 1 0.12201228 1

DB07662 1 1.842615411 1.745287633 0.542671379 0.08481895 1 0.12201228 1

DB07664 1.8 1.753305216 1.757979426 0.337271213 0.549575873 1 0.52649583 1

DB07665 1 0.874648609 1.75379047 0.753349628 0.1585137 1 0.12201228 1

DB07666 1 1.283546282 1.75379047 0.753349628 0.1585137 1 0.12201228 1

DB07668 2 1.857431939 1.75379047 0.753349628 0.628097886 1 0.645919359 1

DB07670 2 1.265518777 1.75379047 0.753349628 0.628097886 1 0.645919359 1

DB07675 1 2.485082691 1.75379047 0.753349628 0.1585137 1 0.12201228 1

DB07676 1.5 2.088150365 1.745287633 0.542671379 0.325634379 1 0.346299761 1

DB07677 2 3.332442188 1.75379047 0.753349628 0.628097886 1 0.645919359 1

DB07678 1 1.052089718 1.745287633 0.542671379 0.08481895 1 0.12201228 1

DB07680 1 1.986447112 1.75379047 0.753349628 0.1585137 1 0.12201228 1

DB07681 2 1.700362436 1.75379047 0.753349628 0.628097886 1 0.645919359 1

DB07683 1 0.736286396 1.75379047 0.753349628 0.1585137 1 0.12201228 1

DB07684 2 2.363571276 1.75379047 0.753349628 0.628097886 1 0.645919359 1

DB07685 1 0.762651738 1.75379047 0.753349628 0.1585137 1 0.12201228 1

DB07686 1 2.340405943 1.75379047 0.753349628 0.1585137 1 0.12201228 1

DB07687 1 1.993078731 1.75379047 0.753349628 0.1585137 1 0.12201228 1

DB07688 1 1.056800719 1.745287633 0.542671379 0.08481895 1 0.12201228 1

DB07691 1 1.101397349 1.75379047 0.753349628 0.1585137 1 0.12201228 1

DB07692 1 1.226991962 1.75379047 0.753349628 0.1585137 1 0.12201228 1

DB07696 1 1.004253188 1.75379047 0.753349628 0.1585137 1 0.12201228 1

DB07697 1 2.624614271 1.75379047 0.753349628 0.1585137 1 0.12201228 1

DB07698 2 2.514179744 1.75379047 0.753349628 0.628097886 1 0.645919359 1

DB07700 1 0.426563305 1.75379047 0.753349628 0.1585137 1 0.12201228 1

DB07701 2 3.082216235 1.75379047 0.753349628 0.628097886 1 0.645919359 1

DB07702 1.5 1.002815596 1.745287633 0.542671379 0.325634379 1 0.346299761 1

DB07707 1 2.098940753 1.745287633 0.542671379 0.08481895 1 0.12201228 1

DB07708 1 1.350517362 1.745287633 0.542671379 0.08481895 1 0.12201228 1

DB07710 2 1.679379849 1.75379047 0.753349628 0.628097886 1 0.645919359 1

DB07711 1 2.562489215 1.75379047 0.753349628 0.1585137 1 0.12201228 1

DB07712 1 2.179609255 1.745287633 0.542671379 0.08481895 1 0.12201228 1

DB07713 1 2.967088589 1.75379047 0.753349628 0.1585137 1 0.12201228 1

DB07714 2 3.399387383 1.75379047 0.753349628 0.628097886 1 0.645919359 1

DB07715 1.5 1.512489687 1.745287633 0.542671379 0.325634379 1 0.346299761 1

DB07717 1 1.555604137 1.75379047 0.753349628 0.1585137 1 0.12201228 1

DB07718 1.5 1.604099914 1.745287633 0.542671379 0.325634379 1 0.346299761 1

DB07719 1 0.834779905 1.75379047 0.753349628 0.1585137 1 0.12201228 1

DB07720 1.8571429 1.884183731 1.736866356 0.292454388 0.659561248 1 0.56136264 1

DB07723 1 2.387897517 1.75379047 0.753349628 0.1585137 1 0.12201228 1

DB07724 1 2.100690365 1.753692879 0.367879736 0.020243381 1 0.12201228 1

DB07728 2 1.580995264 1.745287633 0.542671379 0.680596551 1 0.645919359 1

DB07730 1 0.97234054 1.75379047 0.753349628 0.1585137 1 0.12201228 1

DB07731 1 2.09417642 1.75379047 0.753349628 0.1585137 1 0.12201228 1

DB07733 1 2.221618204 1.75379047 0.753349628 0.1585137 1 0.12201228 1

DB07734 1 0.856276815 1.75379047 0.753349628 0.1585137 1 0.12201228 1

DB07735 1 1.385589155 1.75379047 0.753349628 0.1585137 1 0.12201228 1

DB07736 1 2.024974081 1.75379047 0.753349628 0.1585137 1 0.12201228 1

DB07737 1 2.635834081 1.75379047 0.753349628 0.1585137 1 0.12201228 1

DB07738 1 0.441675367 1.75379047 0.753349628 0.1585137 1 0.12201228 1

DB07739 2 2.293186994 1.75379047 0.753349628 0.628097886 1 0.645919359 1

DB07740 2 1.544051525 1.75379047 0.753349628 0.628097886 1 0.645919359 1

DB07741 1 1.849139584 1.75379047 0.753349628 0.1585137 1 0.12201228 1

DB07742 2 1.850142426 1.75379047 0.753349628 0.628097886 1 0.645919359 1

DB07744 1 1.399649863 1.75379047 0.753349628 0.1585137 1 0.12201228 1

DB07746 2 0.790548927 1.75379047 0.753349628 0.628097886 1 0.645919359 1

DB07747 2 1.503333872 1.75379047 0.753349628 0.628097886 1 0.645919359 1

DB07749 2 1.786118647 1.75379047 0.753349628 0.628097886 1 0.645919359 1

DB07750 1 0.982822669 1.75379047 0.753349628 0.1585137 1 0.12201228 1

DB07751 1 1.356970664 1.75379047 0.753349628 0.1585137 1 0.12201228 1

DB07754 1 0.252174992 1.75379047 0.753349628 0.1585137 1 0.12201228 1

DB07755 1 2.039761843 1.75379047 0.753349628 0.1585137 1 0.12201228 1

DB07756 2 0.069663838 1.75379047 0.753349628 0.628097886 1 0.645919359 1

DB07757 1 0.708281407 1.75379047 0.753349628 0.1585137 1 0.12201228 1

DB07760 1 3.429330887 1.75379047 0.753349628 0.1585137 1 0.12201228 1

DB07761 1 2.441319737 1.75379047 0.753349628 0.1585137 1 0.12201228 1

DB07763 1.7 2.060080131 1.759693587 0.247672276 0.40477041 1 0.46515008 1

DB07766 2 1.611592749 1.75379047 0.753349628 0.628097886 1 0.645919359 1

DB07768 1.5 2.404147301 1.745287633 0.542671379 0.325634379 1 0.346299761 1

DB07769 1 1.344158866 1.75379047 0.753349628 0.1585137 1 0.12201228 1

DB07771 1.6666667 2.289682925 1.757244872 0.464542708 0.422702925 1 0.444812957 1

DB07772 1 2.078407597 1.75379047 0.753349628 0.1585137 1 0.12201228 1

DB07776 -0.6424179 1.798978942 1.757244872 0.464542708 1.20E-07 0.000652461 0.000110733 0.604712798

DB07778 1.7 1.398470817 1.759693587 0.247672276 0.40477041 1 0.46515008 1

DB07779 1 0.753632364 1.75379047 0.753349628 0.1585137 1 0.12201228 1

DB07780 1.8571429 1.597210961 1.736866356 0.292454388 0.659561248 1 0.56136264 1

DB07783 1 1.548888291 1.75379047 0.753349628 0.1585137 1 0.12201228 1

DB07785 1 1.350206168 1.75379047 0.753349628 0.1585137 1 0.12201228 1

DB07786 1 1.861687568 1.75379047 0.753349628 0.1585137 1 0.12201228 1

DB07787 2 1.106881624 1.75379047 0.753349628 0.628097886 1 0.645919359 1

DB07788 1 1.958894313 1.75379047 0.753349628 0.1585137 1 0.12201228 1

DB07789 1 1.87177409 1.75379047 0.753349628 0.1585137 1 0.12201228 1

DB07790 1 1.568669565 1.75379047 0.753349628 0.1585137 1 0.12201228 1

DB07791 1 1.997016022 1.75379047 0.753349628 0.1585137 1 0.12201228 1

DB07792 2 1.919153936 1.75379047 0.753349628 0.628097886 1 0.645919359 1

DB07793 2 2.574250737 1.75379047 0.753349628 0.628097886 1 0.645919359 1

DB07794 1 2.214620655 1.75379047 0.753349628 0.1585137 1 0.12201228 1

DB07795 1 1.328530983 1.75379047 0.753349628 0.1585137 1 0.12201228 1

DB07796 1 1.561705083 1.75379047 0.753349628 0.1585137 1 0.12201228 1

DB07798 2 1.973403947 1.75379047 0.753349628 0.628097886 1 0.645919359 1

DB07800 2 2.841113783 1.75379047 0.753349628 0.628097886 1 0.645919359 1

DB07801 1 1.468593495 1.75379047 0.753349628 0.1585137 1 0.12201228 1

DB07802 2 2.357078127 1.75379047 0.753349628 0.628097886 1 0.645919359 1

DB07804 2 2.460421509 1.75379047 0.753349628 0.628097886 1 0.645919359 1

DB07807 2 2.63886366 1.75379047 0.753349628 0.628097886 1 0.645919359 1

DB07809 1 2.015500183 1.75379047 0.753349628 0.1585137 1 0.12201228 1

DB07811 1 2.973858974 1.75379047 0.753349628 0.1585137 1 0.12201228 1

DB07812 1 1.496720722 1.745287633 0.542671379 0.08481895 1 0.12201228 1

DB07814 2 2.669164716 1.75379047 0.753349628 0.628097886 1 0.645919359 1

DB07815 2 2.052776764 1.75379047 0.753349628 0.628097886 1 0.645919359 1

DB07821 1.6666667 1.939866914 1.757244872 0.464542708 0.422702925 1 0.444812957 1

DB07827 2 1.550155333 1.75379047 0.753349628 0.628097886 1 0.645919359 1

DB07829 1 2.405658168 1.75379047 0.753349628 0.1585137 1 0.12201228 1

DB07830 1 2.489995896 1.75379047 0.753349628 0.1585137 1 0.12201228 1

DB07831 1 1.784290434 1.75379047 0.753349628 0.1585137 1 0.12201228 1

DB07832 1 0.49017131 1.75379047 0.753349628 0.1585137 1 0.12201228 1

DB07833 1 2.989849208 1.75379047 0.753349628 0.1585137 1 0.12201228 1

DB07834 1 2.480336776 1.75379047 0.753349628 0.1585137 1 0.12201228 1

DB07835 1 1.684264998 1.75379047 0.753349628 0.1585137 1 0.12201228 1

DB07836 1 1.710454625 1.75379047 0.753349628 0.1585137 1 0.12201228 1

DB07837 1 1.728230332 1.75379047 0.753349628 0.1585137 1 0.12201228 1

DB07838 2 3.342640541 1.75379047 0.753349628 0.628097886 1 0.645919359 1

DB07839 1 1.620050947 1.75379047 0.753349628 0.1585137 1 0.12201228 1

DB07840 1 0.870881554 1.75379047 0.753349628 0.1585137 1 0.12201228 1

DB07841 1.8571429 2.398280293 1.736866356 0.292454388 0.659561248 1 0.56136264 1

DB07842 1 3.278094123 1.75379047 0.753349628 0.1585137 1 0.12201228 1

DB07843 2 1.833878676 1.75379047 0.753349628 0.628097886 1 0.645919359 1

DB07844 2 1.347152539 1.75379047 0.753349628 0.628097886 1 0.645919359 1

DB07845 1 1.471260694 1.75379047 0.753349628 0.1585137 1 0.12201228 1

DB07846 2 1.652100758 1.75379047 0.753349628 0.628097886 1 0.645919359 1

DB07847 2 1.974218116 1.75379047 0.753349628 0.628097886 1 0.645919359 1

DB07848 2 0.72737944 1.75379047 0.753349628 0.628097886 1 0.645919359 1

DB07849 1 2.824649357 1.75379047 0.753349628 0.1585137 1 0.12201228 1

DB07851 1 2.490268011 1.75379047 0.753349628 0.1585137 1 0.12201228 1

DB07852 1 0.950614307 1.745287633 0.542671379 0.08481895 1 0.12201228 1

DB07853 1.6666667 2.174811833 1.757244872 0.464542708 0.422702925 1 0.444812957 1

DB07854 1.5 1.279053371 1.745287633 0.542671379 0.325634379 1 0.346299761 1

DB07855 1.5 2.165411698 1.745287633 0.542671379 0.325634379 1 0.346299761 1

DB07856 1.5 2.351996186 1.745287633 0.542671379 0.325634379 1 0.346299761 1

DB07857 1.5 1.505660737 1.745287633 0.542671379 0.325634379 1 0.346299761 1

DB07858 1.5 0.853756525 1.745287633 0.542671379 0.325634379 1 0.346299761 1

DB07859 1.25 1.399067276 1.753692879 0.367879736 0.085471804 1 0.217648936 1

DB07860 1.5 2.254662194 1.745287633 0.542671379 0.325634379 1 0.346299761 1

DB07862 2 0.808179746 1.75379047 0.753349628 0.628097886 1 0.645919359 1

DB07863 1.3333333 1.687209036 1.757244872 0.464542708 0.180743525 1 0.257238652 1

DB07866 2 0.565684025 1.75379047 0.753349628 0.628097886 1 0.645919359 1

DB07870 2 1.505798428 1.75379047 0.753349628 0.628097886 1 0.645919359 1

DB07872 2 2.246235715 1.75379047 0.753349628 0.628097886 1 0.645919359 1

DB07873 2 0.963288561 1.75379047 0.753349628 0.628097886 1 0.645919359 1

DB07874 1 2.927484351 1.75379047 0.753349628 0.1585137 1 0.12201228 1

DB07875 2 2.420026376 1.75379047 0.753349628 0.628097886 1 0.645919359 1

DB07876 1.6666667 1.63783323 1.757244872 0.464542708 0.422702925 1 0.444812957 1

DB07877 1 3.228092076 1.745287633 0.542671379 0.08481895 1 0.12201228 1

DB07878 1 2.812075363 1.75379047 0.753349628 0.1585137 1 0.12201228 1

DB07879 1 0.64759891 1.75379047 0.753349628 0.1585137 1 0.12201228 1

DB07887 2 2.968321599 1.75379047 0.753349628 0.628097886 1 0.645919359 1

DB07888 2 2.69642649 1.75379047 0.753349628 0.628097886 1 0.645919359 1

DB07889 1 2.403923657 1.75379047 0.753349628 0.1585137 1 0.12201228 1

DB07895 2 1.477792008 1.745287633 0.542671379 0.680596551 1 0.645919359 1

DB07897 1 1.805396145 1.75379047 0.753349628 0.1585137 1 0.12201228 1

DB07899 2 0.505467433 1.75379047 0.753349628 0.628097886 1 0.645919359 1

DB07900 1 1.472350801 1.75379047 0.753349628 0.1585137 1 0.12201228 1

DB07901 2 2.946028454 1.75379047 0.753349628 0.628097886 1 0.645919359 1

DB07902 2 1.390866256 1.75379047 0.753349628 0.628097886 1 0.645919359 1

DB07903 2 0.614058322 1.75379047 0.753349628 0.628097886 1 0.645919359 1

DB07905 1 1.976228039 1.75379047 0.753349628 0.1585137 1 0.12201228 1

DB07906 2 3.675213983 1.75379047 0.753349628 0.628097886 1 0.645919359 1

DB07907 2 2.278996607 1.75379047 0.753349628 0.628097886 1 0.645919359 1

DB07908 1 1.039301457 1.75379047 0.753349628 0.1585137 1 0.12201228 1

DB07912 2 2.087152471 1.75379047 0.753349628 0.628097886 1 0.645919359 1

DB07913 2 1.272678746 1.75379047 0.753349628 0.628097886 1 0.645919359 1

DB07916 1 0.63359963 1.75379047 0.753349628 0.1585137 1 0.12201228 1

DB07917 1 1.374901279 1.75379047 0.753349628 0.1585137 1 0.12201228 1

DB07919 1 2.102471599 1.75379047 0.753349628 0.1585137 1 0.12201228 1

DB07920 1 1.711273052 1.75379047 0.753349628 0.1585137 1 0.12201228 1

DB07921 1 1.620304021 1.75379047 0.753349628 0.1585137 1 0.12201228 1

DB07922 1 2.913679009 1.75379047 0.753349628 0.1585137 1 0.12201228 1

DB07926 1 1.32766735 1.75379047 0.753349628 0.1585137 1 0.12201228 1

DB07929 1 0.244658842 1.75379047 0.753349628 0.1585137 1 0.12201228 1

DB07930 1 0.961972229 1.75379047 0.753349628 0.1585137 1 0.12201228 1

DB07931 1 2.045080199 1.753692879 0.367879736 0.020243381 1 0.12201228 1

DB07932 1 0.808858229 1.745287633 0.542671379 0.08481895 1 0.12201228 1

DB07933 1 1.205603806 1.745287633 0.542671379 0.08481895 1 0.12201228 1

DB07934 1 2.28071902 1.75379047 0.753349628 0.1585137 1 0.12201228 1

DB07936 1 1.789052246 1.75379047 0.753349628 0.1585137 1 0.12201228 1

DB07940 2 0.795110414 1.75379047 0.753349628 0.628097886 1 0.645919359 1

DB07941 1 1.061664297 1.75379047 0.753349628 0.1585137 1 0.12201228 1

DB07942 1 2.040888096 1.75379047 0.753349628 0.1585137 1 0.12201228 1

DB07943 1 2.50321988 1.75379047 0.753349628 0.1585137 1 0.12201228 1

DB07944 1 1.225441401 1.75379047 0.753349628 0.1585137 1 0.12201228 1

DB07946 1 1.890522886 1.75379047 0.753349628 0.1585137 1 0.12201228 1

DB07947 1.25 2.017320601 1.753692879 0.367879736 0.085471804 1 0.217648936 1

DB07949 2 1.989383456 1.75379047 0.753349628 0.628097886 1 0.645919359 1

DB07950 1 1.999364372 1.745287633 0.542671379 0.08481895 1 0.12201228 1

DB07954 1 1.279389395 1.761473636 0.259363203 0.001662717 1 0.12201228 1

DB07955 2 2.075123962 1.75379047 0.753349628 0.628097886 1 0.645919359 1

DB07956 2 -0.128656959 1.75379047 0.753349628 0.628097886 1 0.645919359 1

DB07957 2 2.560710827 1.75379047 0.753349628 0.628097886 1 0.645919359 1

DB07958 1 2.090446854 1.75379047 0.753349628 0.1585137 1 0.12201228 1

DB07959 2 2.002731044 1.75379047 0.753349628 0.628097886 1 0.645919359 1

DB07960 2 0.553167159 1.75379047 0.753349628 0.628097886 1 0.645919359 1

DB07964 1 2.314628876 1.75379047 0.753349628 0.1585137 1 0.12201228 1

DB07965 2 0.999131461 1.75379047 0.753349628 0.628097886 1 0.645919359 1

DB07966 1 3.506608427 1.75379047 0.753349628 0.1585137 1 0.12201228 1

DB07967 2 1.64164728 1.75379047 0.753349628 0.628097886 1 0.645919359 1

DB07969 1 3.003444989 1.75379047 0.753349628 0.1585137 1 0.12201228 1

DB07970 1 1.130867177 1.75379047 0.753349628 0.1585137 1 0.12201228 1

DB07973 2 2.857604732 1.75379047 0.753349628 0.628097886 1 0.645919359 1

DB07974 2 2.282423382 1.75379047 0.753349628 0.628097886 1 0.645919359 1

DB07975 2 0.120984464 1.75379047 0.753349628 0.628097886 1 0.645919359 1

DB07976 2 1.506039092 1.75379047 0.753349628 0.628097886 1 0.645919359 1

DB07977 2 1.715584991 1.75379047 0.753349628 0.628097886 1 0.645919359 1

DB07978 2 1.435199063 1.75379047 0.753349628 0.628097886 1 0.645919359 1

DB07979 2 2.566457992 1.75379047 0.753349628 0.628097886 1 0.645919359 1

DB07981 2 0.909101066 1.75379047 0.753349628 0.628097886 1 0.645919359 1

DB07982 1 3.984029247 1.75379047 0.753349628 0.1585137 1 0.12201228 1

DB07983 2 1.786456088 1.75379047 0.753349628 0.628097886 1 0.645919359 1

DB07984 2 1.707380217 1.75379047 0.753349628 0.628097886 1 0.645919359 1

DB07985 2 1.275647009 1.75379047 0.753349628 0.628097886 1 0.645919359 1

DB07986 2 1.584374208 1.75379047 0.753349628 0.628097886 1 0.645919359 1

DB07987 2 1.741372324 1.75379047 0.753349628 0.628097886 1 0.645919359 1

DB07988 2 1.142351524 1.75379047 0.753349628 0.628097886 1 0.645919359 1

DB07991 1 1.997707017 1.75379047 0.753349628 0.1585137 1 0.12201228 1

DB07993 1 1.934590597 1.75379047 0.753349628 0.1585137 1 0.12201228 1

DB07994 1 1.22911883 1.75379047 0.753349628 0.1585137 1 0.12201228 1

DB07995 1.6666667 2.475954424 1.757244872 0.464542708 0.422702925 1 0.444812957 1

DB07996 1.5 1.869097117 1.745287633 0.542671379 0.325634379 1 0.346299761 1

DB07997 1.5 1.647217976 1.745287633 0.542671379 0.325634379 1 0.346299761 1

DB07999 2 0.286124654 1.75379047 0.753349628 0.628097886 1 0.645919359 1

DB08000 2 1.44494159 1.75379047 0.753349628 0.628097886 1 0.645919359 1

DB08001 1 2.517665943 1.75379047 0.753349628 0.1585137 1 0.12201228 1

DB08003 1 2.3762458 1.75379047 0.753349628 0.1585137 1 0.12201228 1

DB08004 2 0.756113112 1.75379047 0.753349628 0.628097886 1 0.645919359 1

DB08006 2 2.029261633 1.75379047 0.753349628 0.628097886 1 0.645919359 1

DB08007 2 1.739638403 1.75379047 0.753349628 0.628097886 1 0.645919359 1

DB08008 2 2.288915845 1.75379047 0.753349628 0.628097886 1 0.645919359 1

DB08009 1 2.85421385 1.75379047 0.753349628 0.1585137 1 0.12201228 1

DB08018 1 2.968286442 1.745287633 0.542671379 0.08481895 1 0.12201228 1

DB08019 1 1.380630858 1.745287633 0.542671379 0.08481895 1 0.12201228 1

DB08020 1 1.162087251 1.745287633 0.542671379 0.08481895 1 0.12201228 1

DB08022 1 1.904580998 1.75379047 0.753349628 0.1585137 1 0.12201228 1

DB08024 1 3.0669172 1.75379047 0.753349628 0.1585137 1 0.12201228 1

DB08028 1 1.55702356 1.75379047 0.753349628 0.1585137 1 0.12201228 1

DB08029 2 2.858281464 1.75379047 0.753349628 0.628097886 1 0.645919359 1

DB08030 2 2.820870137 1.75379047 0.753349628 0.628097886 1 0.645919359 1

DB08032 2 0.992101613 1.75379047 0.753349628 0.628097886 1 0.645919359 1

DB08033 2 0.120332951 1.75379047 0.753349628 0.628097886 1 0.645919359 1

DB08035 1 1.835364963 1.75379047 0.753349628 0.1585137 1 0.12201228 1

DB08037 2 3.040349096 1.75379047 0.753349628 0.628097886 1 0.645919359 1

DB08038 1 0.851915417 1.75379047 0.753349628 0.1585137 1 0.12201228 1

DB08039 1.5 1.110200334 1.745287633 0.542671379 0.325634379 1 0.346299761 1

DB08040 2 0.505977767 1.75379047 0.753349628 0.628097886 1 0.645919359 1

DB08042 1 1.361587199 1.75379047 0.753349628 0.1585137 1 0.12201228 1

DB08043 1 2.028097175 1.75379047 0.753349628 0.1585137 1 0.12201228 1

DB08044 1 1.289003749 1.75379047 0.753349628 0.1585137 1 0.12201228 1

DB08045 1 3.324147893 1.75379047 0.753349628 0.1585137 1 0.12201228 1

DB08046 2 0.856285119 1.75379047 0.753349628 0.628097886 1 0.645919359 1

DB08047 1 1.10844195 1.745287633 0.542671379 0.08481895 1 0.12201228 1

DB08048 1 2.468524705 1.745287633 0.542671379 0.08481895 1 0.12201228 1

DB08049 1 2.094683476 1.75379047 0.753349628 0.1585137 1 0.12201228 1

DB08051 1 1.316526824 1.75379047 0.753349628 0.1585137 1 0.12201228 1

DB08052 1 1.666878534 1.75379047 0.753349628 0.1585137 1 0.12201228 1

DB08053 1 1.113244167 1.75379047 0.753349628 0.1585137 1 0.12201228 1

DB08054 1 0.971555482 1.75379047 0.753349628 0.1585137 1 0.12201228 1

DB08055 -6.0684256 1.841586044 1.75379047 0.753349628 1.48E-25 8.11E-22 1.02E-33 5.62E-30

DB08056 -6.0684256 1.507565476 1.75379047 0.753349628 1.48E-25 8.11E-22 1.02E-33 5.62E-30

DB08057 -6.0684256 1.40086413 1.75379047 0.753349628 1.48E-25 8.11E-22 1.02E-33 5.62E-30

DB08058 2 0.140895559 1.75379047 0.753349628 0.628097886 1 0.645919359 1

DB08059 1 1.341786795 1.753692879 0.367879736 0.020243381 1 0.12201228 1

DB08060 1 2.027131442 1.75379047 0.753349628 0.1585137 1 0.12201228 1

DB08061 1 2.136843373 1.75379047 0.753349628 0.1585137 1 0.12201228 1

DB08062 1 2.405395752 1.75379047 0.753349628 0.1585137 1 0.12201228 1

DB08063 2 2.347620745 1.75379047 0.753349628 0.628097886 1 0.645919359 1

DB08064 1 1.756172402 1.75379047 0.753349628 0.1585137 1 0.12201228 1

DB08065 1 0.55688581 1.75379047 0.753349628 0.1585137 1 0.12201228 1

DB08066 1 2.054963993 1.745287633 0.542671379 0.08481895 1 0.12201228 1

DB08067 1 1.013377914 1.75379047 0.753349628 0.1585137 1 0.12201228 1

DB08068 1 0.911003644 1.75379047 0.753349628 0.1585137 1 0.12201228 1

DB08070 1.5 0.928650397 1.745287633 0.542671379 0.325634379 1 0.346299761 1

DB08072 1 2.064419982 1.75379047 0.753349628 0.1585137 1 0.12201228 1

DB08073 1.25 1.913013614 1.753692879 0.367879736 0.085471804 1 0.217648936 1

DB08077 2 1.619892122 1.745287633 0.542671379 0.680596551 1 0.645919359 1

DB08078 1 2.697061358 1.75379047 0.753349628 0.1585137 1 0.12201228 1

DB08079 1 1.440603982 1.75379047 0.753349628 0.1585137 1 0.12201228 1

DB08080 1.5 0.913935019 1.745287633 0.542671379 0.325634379 1 0.346299761 1

DB08082 1 0.660777333 1.75379047 0.753349628 0.1585137 1 0.12201228 1

DB08083 2 1.778981621 1.75379047 0.753349628 0.628097886 1 0.645919359 1

DB08084 2 0.49930258 1.75379047 0.753349628 0.628097886 1 0.645919359 1

DB08085 1 1.759605755 1.75379047 0.753349628 0.1585137 1 0.12201228 1

DB08087 1 0.994661798 1.75379047 0.753349628 0.1585137 1 0.12201228 1

DB08088 1 2.413356689 1.75379047 0.753349628 0.1585137 1 0.12201228 1

DB08089 1 2.048008773 1.75379047 0.753349628 0.1585137 1 0.12201228 1

DB08091 1 1.117984867 1.75379047 0.753349628 0.1585137 1 0.12201228 1

DB08092 1 1.798119326 1.75379047 0.753349628 0.1585137 1 0.12201228 1

DB08093 1 1.152070242 1.75379047 0.753349628 0.1585137 1 0.12201228 1

DB08094 1 1.127292528 1.75379047 0.753349628 0.1585137 1 0.12201228 1

DB08095 1 1.797695469 1.75379047 0.753349628 0.1585137 1 0.12201228 1

DB08096 1 1.009051881 1.75379047 0.753349628 0.1585137 1 0.12201228 1

DB08097 1 2.586977858 1.75379047 0.753349628 0.1585137 1 0.12201228 1

DB08098 2 1.591275193 1.75379047 0.753349628 0.628097886 1 0.645919359 1

DB08099 2 1.86778285 1.75379047 0.753349628 0.628097886 1 0.645919359 1

DB08113 1.5 3.099229505 1.745287633 0.542671379 0.325634379 1 0.346299761 1

DB08114 1.5 2.194581982 1.745287633 0.542671379 0.325634379 1 0.346299761 1

DB08118 1 1.266260106 1.75379047 0.753349628 0.1585137 1 0.12201228 1

DB08119 2 1.295979868 1.75379047 0.753349628 0.628097886 1 0.645919359 1

DB08121 1 1.173500059 1.75379047 0.753349628 0.1585137 1 0.12201228 1

DB08122 1 1.299617018 1.75379047 0.753349628 0.1585137 1 0.12201228 1

DB08123 1 2.110395181 1.75379047 0.753349628 0.1585137 1 0.12201228 1

DB08124 1 0.984052192 1.75379047 0.753349628 0.1585137 1 0.12201228 1

DB08125 1 0.630290655 1.75379047 0.753349628 0.1585137 1 0.12201228 1

DB08126 1 1.466788207 1.75379047 0.753349628 0.1585137 1 0.12201228 1

DB08128 2 1.835390082 1.75379047 0.753349628 0.628097886 1 0.645919359 1

DB08129 2 1.108255376 1.75379047 0.753349628 0.628097886 1 0.645919359 1

DB08130 1 2.229682232 1.75379047 0.753349628 0.1585137 1 0.12201228 1

DB08132 1 1.273565245 1.75379047 0.753349628 0.1585137 1 0.12201228 1

DB08133 1 3.266077108 1.75379047 0.753349628 0.1585137 1 0.12201228 1

DB08134 1 0.646495861 1.75379047 0.753349628 0.1585137 1 0.12201228 1

DB08135 1 1.298463029 1.75379047 0.753349628 0.1585137 1 0.12201228 1

DB08136 1 2.777641747 1.75379047 0.753349628 0.1585137 1 0.12201228 1

DB08137 1 1.817833737 1.75379047 0.753349628 0.1585137 1 0.12201228 1

DB08138 1 1.265085729 1.75379047 0.753349628 0.1585137 1 0.12201228 1

DB08139 1 2.419176966 1.75379047 0.753349628 0.1585137 1 0.12201228 1

DB08140 1 1.519321899 1.75379047 0.753349628 0.1585137 1 0.12201228 1

DB08141 1 1.965378547 1.75379047 0.753349628 0.1585137 1 0.12201228 1

DB08142 1 1.776110476 1.745287633 0.542671379 0.08481895 1 0.12201228 1

DB08143 2 2.601455227 1.75379047 0.753349628 0.628097886 1 0.645919359 1

DB08147 1 1.537925809 1.75379047 0.753349628 0.1585137 1 0.12201228 1

DB08148 1.5 1.555158689 1.745287633 0.542671379 0.325634379 1 0.346299761 1

DB08149 1.5 1.823792716 1.745287633 0.542671379 0.325634379 1 0.346299761 1

DB08150 1.5 1.861293969 1.745287633 0.542671379 0.325634379 1 0.346299761 1

DB08151 2 2.33396278 1.75379047 0.753349628 0.628097886 1 0.645919359 1

DB08152 1 3.919837733 1.75379047 0.753349628 0.1585137 1 0.12201228 1

DB08153 1 0.841092484 1.75379047 0.753349628 0.1585137 1 0.12201228 1

DB08155 2 1.224841163 1.745287633 0.542671379 0.680596551 1 0.645919359 1

DB08156 2 1.232092461 1.745287633 0.542671379 0.680596551 1 0.645919359 1

DB08157 2 2.310882326 1.745287633 0.542671379 0.680596551 1 0.645919359 1

DB08159 2 1.610912288 1.75379047 0.753349628 0.628097886 1 0.645919359 1

DB08162 1.5 1.935012373 1.753692879 0.367879736 0.245220308 1 0.346299761 1

DB08163 2 3.129984458 1.75379047 0.753349628 0.628097886 1 0.645919359 1

DB08164 1 0.783137057 1.75379047 0.753349628 0.1585137 1 0.12201228 1

DB08165 2 2.189342165 1.75379047 0.753349628 0.628097886 1 0.645919359 1

DB08166 1 2.342895873 1.75379047 0.753349628 0.1585137 1 0.12201228 1

DB08167 2 1.910296712 1.75379047 0.753349628 0.628097886 1 0.645919359 1

DB08168 2 1.473877194 1.745287633 0.542671379 0.680596551 1 0.645919359 1

DB08169 2 0.395761512 1.75379047 0.753349628 0.628097886 1 0.645919359 1

DB08170 1 1.287074008 1.75379047 0.753349628 0.1585137 1 0.12201228 1

DB08172 2 2.658455537 1.75379047 0.753349628 0.628097886 1 0.645919359 1

DB08173 2 1.87768324 1.75379047 0.753349628 0.628097886 1 0.645919359 1

DB08174 2 -0.016748149 1.75379047 0.753349628 0.628097886 1 0.645919359 1

DB08175 1 2.18448985 1.745287633 0.542671379 0.08481895 1 0.12201228 1

DB08176 1 2.648932265 1.75379047 0.753349628 0.1585137 1 0.12201228 1

DB08178 1 2.214046724 1.745287633 0.542671379 0.08481895 1 0.12201228 1

DB08179 2 1.411660338 1.75379047 0.753349628 0.628097886 1 0.645919359 1

DB08180 1.5 1.591047353 1.745287633 0.542671379 0.325634379 1 0.346299761 1

DB08182 1 2.918263378 1.745287633 0.542671379 0.08481895 1 0.12201228 1

DB08184 2 0.556526699 1.75379047 0.753349628 0.628097886 1 0.645919359 1

DB08187 1 0.440988248 1.75379047 0.753349628 0.1585137 1 0.12201228 1

DB08190 2 0.928549407 1.75379047 0.753349628 0.628097886 1 0.645919359 1

DB08191 1 2.143431479 1.75379047 0.753349628 0.1585137 1 0.12201228 1

DB08192 1 0.638020377 1.75379047 0.753349628 0.1585137 1 0.12201228 1

DB08194 1 1.634043748 1.75379047 0.753349628 0.1585137 1 0.12201228 1

DB08195 1 1.908883703 1.75379047 0.753349628 0.1585137 1 0.12201228 1

DB08197 1 1.680035001 1.75379047 0.753349628 0.1585137 1 0.12201228 1

DB08198 2 2.151609318 1.75379047 0.753349628 0.628097886 1 0.645919359 1

DB08200 2 2.882797897 1.75379047 0.753349628 0.628097886 1 0.645919359 1

DB08201 2 2.430279358 1.75379047 0.753349628 0.628097886 1 0.645919359 1

DB08202 2 2.196236864 1.75379047 0.753349628 0.628097886 1 0.645919359 1

DB08203 2 1.224441288 1.75379047 0.753349628 0.628097886 1 0.645919359 1

DB08208 1 2.722073032 1.75379047 0.753349628 0.1585137 1 0.12201228 1

DB08210 1 1.122734414 1.75379047 0.753349628 0.1585137 1 0.12201228 1

DB08213 1 2.516808661 1.75379047 0.753349628 0.1585137 1 0.12201228 1

DB08214 1 1.565765149 1.75379047 0.753349628 0.1585137 1 0.12201228 1

DB08217 -2.0964784 2.850235575 1.745287633 0.542671379 7.24E-13 3.96E-09 1.50E-09 8.21E-06

DB08218 1 2.213029515 1.745287633 0.542671379 0.08481895 1 0.12201228 1

DB08219 1 0.969991656 1.745287633 0.542671379 0.08481895 1 0.12201228 1

DB08220 1.5 1.558813444 1.745287633 0.542671379 0.325634379 1 0.346299761 1

DB08221 1 2.279440197 1.75379047 0.753349628 0.1585137 1 0.12201228 1

DB08222 1 2.065193938 1.745287633 0.542671379 0.08481895 1 0.12201228 1

DB08224 2 2.106036448 1.75379047 0.753349628 0.628097886 1 0.645919359 1

DB08228 2 1.899492069 1.75379047 0.753349628 0.628097886 1 0.645919359 1

DB08229 1 3.03509567 1.75379047 0.753349628 0.1585137 1 0.12201228 1

DB08230 1 0.374960994 1.75379047 0.753349628 0.1585137 1 0.12201228 1

DB08231 1.2777778 2.055584916 1.755399642 0.180590294 0.004087186 1 0.230439784 1

DB08232 1.5 1.858623381 1.745287633 0.542671379 0.325634379 1 0.346299761 1

DB08233 1 0.975550129 1.745287633 0.542671379 0.08481895 1 0.12201228 1

DB08234 2 1.583881787 1.75379047 0.753349628 0.628097886 1 0.645919359 1

DB08235 1 2.01892825 1.736866356 0.292454388 0.005874511 1 0.12201228 1

DB08236 1 1.834927822 1.736866356 0.292454388 0.005874511 1 0.12201228 1

DB08237 2 2.493973608 1.75379047 0.753349628 0.628097886 1 0.645919359 1

DB08238 2 0.97453813 1.75379047 0.753349628 0.628097886 1 0.645919359 1

DB08239 2 0.780288952 1.75379047 0.753349628 0.628097886 1 0.645919359 1

DB08241 1 2.054509932 1.745287633 0.542671379 0.08481895 1 0.12201228 1

DB08242 1 1.80093572 1.75379047 0.753349628 0.1585137 1 0.12201228 1

DB08244 2 2.125793511 1.75379047 0.753349628 0.628097886 1 0.645919359 1

DB08246 2 2.486312562 1.75379047 0.753349628 0.628097886 1 0.645919359 1

DB08247 1 1.866024982 1.75379047 0.753349628 0.1585137 1 0.12201228 1

DB08248 1 1.438695847 1.745287633 0.542671379 0.08481895 1 0.12201228 1

DB08249 2 0.628321901 1.75379047 0.753349628 0.628097886 1 0.645919359 1

DB08250 2 1.958116453 1.75379047 0.753349628 0.628097886 1 0.645919359 1

DB08251 1 2.407678253 1.75379047 0.753349628 0.1585137 1 0.12201228 1

DB08254 1.5 1.386793919 1.745287633 0.542671379 0.325634379 1 0.346299761 1

DB08256 2 2.409206139 1.75379047 0.753349628 0.628097886 1 0.645919359 1

DB08257 2 -0.010085214 1.75379047 0.753349628 0.628097886 1 0.645919359 1

DB08258 2 0.140968009 1.75379047 0.753349628 0.628097886 1 0.645919359 1

DB08259 2 1.12726107 1.75379047 0.753349628 0.628097886 1 0.645919359 1

DB08262 2 0.773306818 1.745287633 0.542671379 0.680596551 1 0.645919359 1

DB08263 2 2.573791932 1.75379047 0.753349628 0.628097886 1 0.645919359 1

DB08270 2 2.317611417 1.75379047 0.753349628 0.628097886 1 0.645919359 1

DB08271 1.75 1.642220635 1.753692879 0.367879736 0.495995374 1 0.495810565 1

DB08277 2 2.426602633 1.75379047 0.753349628 0.628097886 1 0.645919359 1

DB08280 2 1.015575759 1.75379047 0.753349628 0.628097886 1 0.645919359 1

DB08283 1 0.476240113 1.75379047 0.753349628 0.1585137 1 0.12201228 1

DB08285 1 2.290274322 1.745287633 0.542671379 0.08481895 1 0.12201228 1

DB08287 2 1.304252734 1.75379047 0.753349628 0.628097886 1 0.645919359 1

DB08292 1 2.537078322 1.75379047 0.753349628 0.1585137 1 0.12201228 1

DB08293 1 2.134357336 1.75379047 0.753349628 0.1585137 1 0.12201228 1

DB08299 1 1.85173984 1.757244872 0.464542708 0.051541591 1 0.12201228 1

DB08300 1 0.72468431 1.75379047 0.753349628 0.1585137 1 0.12201228 1

DB08301 2 1.420380166 1.75379047 0.753349628 0.628097886 1 0.645919359 1

DB08302 1 1.732079464 1.75379047 0.753349628 0.1585137 1 0.12201228 1

DB08303 2 1.670025255 1.75379047 0.753349628 0.628097886 1 0.645919359 1

DB08304 2 2.983914709 1.75379047 0.753349628 0.628097886 1 0.645919359 1

DB08305 2 1.704705106 1.75379047 0.753349628 0.628097886 1 0.645919359 1

DB08309 1 2.604391129 1.745287633 0.542671379 0.08481895 1 0.12201228 1

DB08312 1 2.071523423 1.75379047 0.753349628 0.1585137 1 0.12201228 1

DB08313 1 0.352013197 1.75379047 0.753349628 0.1585137 1 0.12201228 1

DB08320 1 1.495036215 1.745287633 0.542671379 0.08481895 1 0.12201228 1

DB08321 2 2.005619187 1.75379047 0.753349628 0.628097886 1 0.645919359 1

DB08322 2 0.963601138 1.75379047 0.753349628 0.628097886 1 0.645919359 1

DB08325 2 1.900056152 1.745287633 0.542671379 0.680596551 1 0.645919359 1

DB08326 2 2.291515391 1.75379047 0.753349628 0.628097886 1 0.645919359 1

DB08329 2 1.72755661 1.75379047 0.753349628 0.628097886 1 0.645919359 1

DB08330 1.7 1.721552192 1.759693587 0.247672276 0.40477041 1 0.46515008 1

DB08333 2 1.967317294 1.75379047 0.753349628 0.628097886 1 0.645919359 1

DB08334 2 2.768162804 1.75379047 0.753349628 0.628097886 1 0.645919359 1

DB08335 2 2.276459347 1.75379047 0.753349628 0.628097886 1 0.645919359 1

DB08338 2 1.568056437 1.75379047 0.753349628 0.628097886 1 0.645919359 1

DB08339 1 2.065562642 1.75379047 0.753349628 0.1585137 1 0.12201228 1

DB08340 2 1.4478062 1.75379047 0.753349628 0.628097886 1 0.645919359 1

DB08341 1 2.574981918 1.75379047 0.753349628 0.1585137 1 0.12201228 1

DB08342 2 2.079023616 1.757979426 0.337271213 0.763493223 1 0.645919359 1

DB08345 2 1.917625468 1.75379047 0.753349628 0.628097886 1 0.645919359 1

DB08346 1 1.236110116 1.75379047 0.753349628 0.1585137 1 0.12201228 1

DB08347 2 2.307999084 1.75379047 0.753349628 0.628097886 1 0.645919359 1

DB08348 2 1.541297115 1.757244872 0.464542708 0.699362524 1 0.645919359 1

DB08349 1 1.197891992 1.75379047 0.753349628 0.1585137 1 0.12201228 1

DB08350 1 3.678577776 1.75379047 0.753349628 0.1585137 1 0.12201228 1

DB08351 1 0.304672553 1.75379047 0.753349628 0.1585137 1 0.12201228 1

DB08352 1 2.748822984 1.75379047 0.753349628 0.1585137 1 0.12201228 1

DB08353 2 1.662164931 1.75379047 0.753349628 0.628097886 1 0.645919359 1

DB08354 2 0.849338235 1.75379047 0.753349628 0.628097886 1 0.645919359 1

DB08355 1 1.905424922 1.745287633 0.542671379 0.08481895 1 0.12201228 1

DB08356 1 2.087614912 1.75379047 0.753349628 0.1585137 1 0.12201228 1

DB08357 2 2.039764321 1.75379047 0.753349628 0.628097886 1 0.645919359 1

DB08358 2 2.295085176 1.745287633 0.542671379 0.680596551 1 0.645919359 1

DB08360 2 1.844653722 1.75379047 0.753349628 0.628097886 1 0.645919359 1

DB08361 1 0.360739166 1.75379047 0.753349628 0.1585137 1 0.12201228 1

DB08362 2 0.872010372 1.75379047 0.753349628 0.628097886 1 0.645919359 1

DB08363 1 1.608038457 1.75379047 0.753349628 0.1585137 1 0.12201228 1

DB08365 2 2.82076563 1.75379047 0.753349628 0.628097886 1 0.645919359 1

DB08368 2 1.304689105 1.75379047 0.753349628 0.628097886 1 0.645919359 1

DB08369 1 0.912777276 1.75379047 0.753349628 0.1585137 1 0.12201228 1

DB08370 1 0.39503481 1.75379047 0.753349628 0.1585137 1 0.12201228 1

DB08371 1.5 0.23011673 1.745287633 0.542671379 0.325634379 1 0.346299761 1

DB08373 1 2.128074893 1.75379047 0.753349628 0.1585137 1 0.12201228 1

DB08374 2 0.672773467 1.75379047 0.753349628 0.628097886 1 0.645919359 1

DB08378 1.6666667 2.113955318 1.757244872 0.464542708 0.422702925 1 0.444812957 1

DB08382 1 1.379042025 1.75379047 0.753349628 0.1585137 1 0.12201228 1

DB08385 1 2.472693643 1.75379047 0.753349628 0.1585137 1 0.12201228 1

DB08388 2 2.694531317 1.75379047 0.753349628 0.628097886 1 0.645919359 1

DB08392 2 0.919655563 1.75379047 0.753349628 0.628097886 1 0.645919359 1

DB08393 2 1.897444106 1.75379047 0.753349628 0.628097886 1 0.645919359 1

DB08395 1 1.726881151 1.75379047 0.753349628 0.1585137 1 0.12201228 1

DB08397 1 2.609703939 1.75379047 0.753349628 0.1585137 1 0.12201228 1

DB08398 1 1.582287015 1.745287633 0.542671379 0.08481895 1 0.12201228 1

DB08399 2 2.210075621 1.757244872 0.464542708 0.699362524 1 0.645919359 1

DB08400 1 1.447149186 1.75379047 0.753349628 0.1585137 1 0.12201228 1

DB08402 1.3333333 2.619956234 1.757244872 0.464542708 0.180743525 1 0.257238652 1

DB08403 1 2.248776759 1.745287633 0.542671379 0.08481895 1 0.12201228 1

DB08406 2 1.724506916 1.75379047 0.753349628 0.628097886 1 0.645919359 1

DB08413 2 2.231388889 1.75379047 0.753349628 0.628097886 1 0.645919359 1

DB08416 2 2.815920991 1.75379047 0.753349628 0.628097886 1 0.645919359 1

DB08418 2 2.188903042 1.75379047 0.753349628 0.628097886 1 0.645919359 1

DB08420 2 2.648409935 1.75379047 0.753349628 0.628097886 1 0.645919359 1

DB08422 1 2.007861624 1.75379047 0.753349628 0.1585137 1 0.12201228 1

DB08423 1 1.890037917 1.75379047 0.753349628 0.1585137 1 0.12201228 1

DB08424 1 2.847109803 1.75379047 0.753349628 0.1585137 1 0.12201228 1

DB08426 2 2.043266854 1.75379047 0.753349628 0.628097886 1 0.645919359 1

DB08429 1 2.459835021 1.75379047 0.753349628 0.1585137 1 0.12201228 1

DB08430 2 0.268699826 1.75379047 0.753349628 0.628097886 1 0.645919359 1

DB08435 1 0.96938889 1.75379047 0.753349628 0.1585137 1 0.12201228 1

DB08436 1 2.915764512 1.75379047 0.753349628 0.1585137 1 0.12201228 1

DB08437 2 1.944390701 1.763093512 0.211303302 0.868891809 1 0.645919359 1

DB08439 1 1.834679253 1.745287633 0.542671379 0.08481895 1 0.12201228 1

DB08441 1 1.314871385 1.75379047 0.753349628 0.1585137 1 0.12201228 1

DB08442 1 2.443295607 1.75379047 0.753349628 0.1585137 1 0.12201228 1

DB08443 1 1.837180925 1.75379047 0.753349628 0.1585137 1 0.12201228 1

DB08445 1 1.745906944 1.75379047 0.753349628 0.1585137 1 0.12201228 1

DB08447 1 1.138377795 1.75379047 0.753349628 0.1585137 1 0.12201228 1

DB08448 2 1.67255017 1.75379047 0.753349628 0.628097886 1 0.645919359 1

DB08449 2 2.874363894 1.75379047 0.753349628 0.628097886 1 0.645919359 1

DB08450 2 1.393015129 1.75379047 0.753349628 0.628097886 1 0.645919359 1

DB08453 1.7 1.981791949 1.759693587 0.247672276 0.40477041 1 0.46515008 1

DB08454 1 1.750420869 1.75379047 0.753349628 0.1585137 1 0.12201228 1

DB08461 1 2.744780949 1.75379047 0.753349628 0.1585137 1 0.12201228 1

DB08462 1 1.260608837 1.75379047 0.753349628 0.1585137 1 0.12201228 1

DB08463 1 1.696847628 1.745287633 0.542671379 0.08481895 1 0.12201228 1

DB08464 1 1.03697542 1.745287633 0.542671379 0.08481895 1 0.12201228 1

DB08465 1 1.485928744 1.745287633 0.542671379 0.08481895 1 0.12201228 1

DB08466 2 2.689608028 1.75379047 0.753349628 0.628097886 1 0.645919359 1

DB08473 2 0.753772651 1.75379047 0.753349628 0.628097886 1 0.645919359 1

DB08476 1 1.305305308 1.75379047 0.753349628 0.1585137 1 0.12201228 1

DB08478 1 1.390255027 1.75379047 0.753349628 0.1585137 1 0.12201228 1

DB08479 1 1.330377831 1.75379047 0.753349628 0.1585137 1 0.12201228 1

DB08480 1 0.826041227 1.75379047 0.753349628 0.1585137 1 0.12201228 1

DB08482 1 1.351871856 1.75379047 0.753349628 0.1585137 1 0.12201228 1

DB08484 1 1.333299191 1.75379047 0.753349628 0.1585137 1 0.12201228 1

DB08486 2 1.366284352 1.745287633 0.542671379 0.680596551 1 0.645919359 1

DB08487 2 1.125308394 1.75379047 0.753349628 0.628097886 1 0.645919359 1

DB08488 2 2.426769899 1.75379047 0.753349628 0.628097886 1 0.645919359 1

DB08489 1 0.370302508 1.75379047 0.753349628 0.1585137 1 0.12201228 1

DB08490 2 1.865218459 1.75379047 0.753349628 0.628097886 1 0.645919359 1

DB08491 1 1.116627607 1.75379047 0.753349628 0.1585137 1 0.12201228 1

DB08492 2 2.069475578 1.75379047 0.753349628 0.628097886 1 0.645919359 1

DB08493 1 1.011602083 1.75379047 0.753349628 0.1585137 1 0.12201228 1

DB08495 2 1.999920037 1.75379047 0.753349628 0.628097886 1 0.645919359 1

DB08497 1 2.404144507 1.75379047 0.753349628 0.1585137 1 0.12201228 1

DB08498 1 1.444604432 1.75379047 0.753349628 0.1585137 1 0.12201228 1

DB08499 1 -0.051637723 1.75379047 0.753349628 0.1585137 1 0.12201228 1

DB08500 2 1.500190976 1.75379047 0.753349628 0.628097886 1 0.645919359 1

DB08503 2 1.476293531 1.75379047 0.753349628 0.628097886 1 0.645919359 1

DB08504 1 1.684456744 1.75379047 0.753349628 0.1585137 1 0.12201228 1

DB08507 2 1.954831672 1.75379047 0.753349628 0.628097886 1 0.645919359 1

DB08513 1 1.18608637 1.757244872 0.464542708 0.051541591 1 0.12201228 1

DB08515 1 1.897607844 1.773663076 0.187305714 1.81E-05 0.098319689 0.12201228 1

DB08516 1 3.683516182 1.75379047 0.753349628 0.1585137 1 0.12201228 1

DB08519 1 1.635549906 1.75379047 0.753349628 0.1585137 1 0.12201228 1

DB08520 2 2.444597346 1.75379047 0.753349628 0.628097886 1 0.645919359 1

DB08521 1 1.14878492 1.745287633 0.542671379 0.08481895 1 0.12201228 1

DB08522 1 2.063243972 1.75379047 0.753349628 0.1585137 1 0.12201228 1

DB08526 2 2.251858287 1.75379047 0.753349628 0.628097886 1 0.645919359 1

DB08527 1 1.528601531 1.745287633 0.542671379 0.08481895 1 0.12201228 1

DB08529 2 2.301401154 1.75379047 0.753349628 0.628097886 1 0.645919359 1

DB08530 1 1.345918367 1.75379047 0.753349628 0.1585137 1 0.12201228 1

DB08531 1 2.23149744 1.75379047 0.753349628 0.1585137 1 0.12201228 1

DB08532 1 1.500808611 1.75379047 0.753349628 0.1585137 1 0.12201228 1

DB08533 1 1.943110885 1.75379047 0.753349628 0.1585137 1 0.12201228 1

DB08534 1 1.04616115 1.75379047 0.753349628 0.1585137 1 0.12201228 1

DB08535 1 1.576730215 1.75379047 0.753349628 0.1585137 1 0.12201228 1

DB08536 1 1.969852575 1.75379047 0.753349628 0.1585137 1 0.12201228 1

DB08537 1 2.037421578 1.75379047 0.753349628 0.1585137 1 0.12201228 1

DB08538 1 1.955577882 1.75379047 0.753349628 0.1585137 1 0.12201228 1

DB08539 1 2.43112631 1.75379047 0.753349628 0.1585137 1 0.12201228 1

DB08546 1 1.202511399 1.75379047 0.753349628 0.1585137 1 0.12201228 1

DB08549 1 2.194500596 1.75379047 0.753349628 0.1585137 1 0.12201228 1

DB08550 2 3.35881972 1.75379047 0.753349628 0.628097886 1 0.645919359 1

DB08557 1 1.928188957 1.75379047 0.753349628 0.1585137 1 0.12201228 1

DB08558 2 2.242330383 1.745287633 0.542671379 0.680596551 1 0.645919359 1

DB08560 1 1.404035887 1.75379047 0.753349628 0.1585137 1 0.12201228 1

DB08561 2 3.072325201 1.75379047 0.753349628 0.628097886 1 0.645919359 1

DB08564 1 1.179464618 1.75379047 0.753349628 0.1585137 1 0.12201228 1

DB08565 2 0.814135135 1.75379047 0.753349628 0.628097886 1 0.645919359 1

DB08566 2 1.656303046 1.75379047 0.753349628 0.628097886 1 0.645919359 1

DB08568 1.5 2.000857245 1.745287633 0.542671379 0.325634379 1 0.346299761 1

DB08569 1.5 1.172747726 1.745287633 0.542671379 0.325634379 1 0.346299761 1

DB08572 1 1.418803766 1.745287633 0.542671379 0.08481895 1 0.12201228 1

DB08574 1 1.103691174 1.75379047 0.753349628 0.1585137 1 0.12201228 1

DB08575 1 2.266395027 1.75379047 0.753349628 0.1585137 1 0.12201228 1

DB08577 1 3.002944024 1.75379047 0.753349628 0.1585137 1 0.12201228 1

DB08583 1 0.587053966 1.75379047 0.753349628 0.1585137 1 0.12201228 1

DB08588 1 3.092005688 1.75379047 0.753349628 0.1585137 1 0.12201228 1

DB08591 1 1.925857172 1.75379047 0.753349628 0.1585137 1 0.12201228 1

DB08593 1 1.794916825 1.75379047 0.753349628 0.1585137 1 0.12201228 1

DB08594 2 2.27523072 1.75379047 0.753349628 0.628097886 1 0.645919359 1

DB08595 1 2.295245225 1.745287633 0.542671379 0.08481895 1 0.12201228 1

DB08596 2 1.089526584 1.75379047 0.753349628 0.628097886 1 0.645919359 1

DB08597 2 1.973133856 1.745287633 0.542671379 0.680596551 1 0.645919359 1

DB08599 1 1.685707487 1.75379047 0.753349628 0.1585137 1 0.12201228 1

DB08601 1.5 1.837943847 1.745287633 0.542671379 0.325634379 1 0.346299761 1

DB08602 1 3.823537175 1.75379047 0.753349628 0.1585137 1 0.12201228 1

DB08604 1 2.930431401 1.757979426 0.337271213 0.012307606 1 0.12201228 1

DB08605 1 2.396364407 1.75379047 0.753349628 0.1585137 1 0.12201228 1

DB08607 1 2.079858857 1.75379047 0.753349628 0.1585137 1 0.12201228 1

DB08608 1 1.876660413 1.75379047 0.753349628 0.1585137 1 0.12201228 1

DB08609 1 3.348208144 1.75379047 0.753349628 0.1585137 1 0.12201228 1

DB08610 1 1.88236339 1.75379047 0.753349628 0.1585137 1 0.12201228 1

DB08611 1 2.095556664 1.75379047 0.753349628 0.1585137 1 0.12201228 1

DB08613 1 1.640798665 1.75379047 0.753349628 0.1585137 1 0.12201228 1

DB08614 2 2.2475699 1.75379047 0.753349628 0.628097886 1 0.645919359 1

DB08615 2 3.054707058 1.75379047 0.753349628 0.628097886 1 0.645919359 1

DB08617 2 1.526239274 1.75379047 0.753349628 0.628097886 1 0.645919359 1

DB08620 2 2.50843741 1.75379047 0.753349628 0.628097886 1 0.645919359 1

DB08624 1 2.026380967 1.745287633 0.542671379 0.08481895 1 0.12201228 1

DB08626 1 2.357821439 1.75379047 0.753349628 0.1585137 1 0.12201228 1

DB08629 2 1.096951332 1.757244872 0.464542708 0.699362524 1 0.645919359 1

DB08631 2 2.459920584 1.75379047 0.753349628 0.628097886 1 0.645919359 1

DB08632 2 2.099020183 1.745287633 0.542671379 0.680596551 1 0.645919359 1

DB08633 2 0.706652394 1.75379047 0.753349628 0.628097886 1 0.645919359 1

DB08640 2 1.598608059 1.75379047 0.753349628 0.628097886 1 0.645919359 1

DB08641 2 1.659075767 1.75379047 0.753349628 0.628097886 1 0.645919359 1

DB08642 2 1.568707064 1.75379047 0.753349628 0.628097886 1 0.645919359 1

DB08643 2 1.789835619 1.75379047 0.753349628 0.628097886 1 0.645919359 1

DB08645 2 1.586839645 1.75379047 0.753349628 0.628097886 1 0.645919359 1

DB08651 2 3.583831857 1.75379047 0.753349628 0.628097886 1 0.645919359 1

DB08654 2 2.801014703 1.75379047 0.753349628 0.628097886 1 0.645919359 1

DB08655 1 2.676986563 1.75379047 0.753349628 0.1585137 1 0.12201228 1

DB08658 2 1.543281633 1.75379047 0.753349628 0.628097886 1 0.645919359 1

DB08659 2 0.554545739 1.75379047 0.753349628 0.628097886 1 0.645919359 1

DB08660 2 2.496508931 1.75379047 0.753349628 0.628097886 1 0.645919359 1

DB08661 2 2.131980214 1.75379047 0.753349628 0.628097886 1 0.645919359 1

DB08672 1 1.648693875 1.75379047 0.753349628 0.1585137 1 0.12201228 1

DB08673 1 2.617215315 1.75379047 0.753349628 0.1585137 1 0.12201228 1

DB08674 2 1.497106202 1.745287633 0.542671379 0.680596551 1 0.645919359 1

DB08675 2 2.179291418 1.75379047 0.753349628 0.628097886 1 0.645919359 1

DB08676 2 1.970044235 1.745287633 0.542671379 0.680596551 1 0.645919359 1

DB08677 1 2.434801895 1.75379047 0.753349628 0.1585137 1 0.12201228 1

DB08678 1 0.856383271 1.75379047 0.753349628 0.1585137 1 0.12201228 1

DB08683 2 1.656722472 1.75379047 0.753349628 0.628097886 1 0.645919359 1

DB08687 1 1.241412174 1.745287633 0.542671379 0.08481895 1 0.12201228 1

DB08688 2 2.642662952 1.75379047 0.753349628 0.628097886 1 0.645919359 1

DB08689 1.5 1.754402655 1.753692879 0.367879736 0.245220308 1 0.346299761 1

DB08690 1.7 1.903817411 1.759693587 0.247672276 0.40477041 1 0.46515008 1

DB08691 2 2.308593846 1.745287633 0.542671379 0.680596551 1 0.645919359 1

DB08692 2 2.384056712 1.75379047 0.753349628 0.628097886 1 0.645919359 1

DB08693 2 1.265101475 1.75379047 0.753349628 0.628097886 1 0.645919359 1

DB08694 1 1.720441034 1.745287633 0.542671379 0.08481895 1 0.12201228 1

DB08695 1 1.907618144 1.75379047 0.753349628 0.1585137 1 0.12201228 1

DB08697 1 1.531631035 1.75379047 0.753349628 0.1585137 1 0.12201228 1

DB08703 2 0.388787607 1.75379047 0.753349628 0.628097886 1 0.645919359 1

DB08705 1 2.409539993 1.75379047 0.753349628 0.1585137 1 0.12201228 1

DB08707 1 1.883872306 1.75379047 0.753349628 0.1585137 1 0.12201228 1

DB08708 1 2.471393111 1.75379047 0.753349628 0.1585137 1 0.12201228 1

DB08709 1 2.190635185 1.75379047 0.753349628 0.1585137 1 0.12201228 1

DB08729 1 2.631085123 1.75379047 0.753349628 0.1585137 1 0.12201228 1

DB08730 1 2.356261313 1.75379047 0.753349628 0.1585137 1 0.12201228 1

DB08733 1 3.174396444 1.75379047 0.753349628 0.1585137 1 0.12201228 1

DB08734 1 2.329817194 1.75379047 0.753349628 0.1585137 1 0.12201228 1

DB08737 1 2.11752821 1.75379047 0.753349628 0.1585137 1 0.12201228 1

DB08740 1 1.593778188 1.75379047 0.753349628 0.1585137 1 0.12201228 1

DB08742 1.5 1.334728684 1.745287633 0.542671379 0.325634379 1 0.346299761 1

DB08743 1 1.201299643 1.75379047 0.753349628 0.1585137 1 0.12201228 1

DB08744 2 1.945382169 1.75379047 0.753349628 0.628097886 1 0.645919359 1

DB08745 2 1.017131102 1.75379047 0.753349628 0.628097886 1 0.645919359 1

DB08746 2 2.231449709 1.75379047 0.753349628 0.628097886 1 0.645919359 1

DB08749 1 2.080720529 1.75379047 0.753349628 0.1585137 1 0.12201228 1

DB08750 1 1.591841844 1.75379047 0.753349628 0.1585137 1 0.12201228 1

DB08751 1 -0.050231302 1.75379047 0.753349628 0.1585137 1 0.12201228 1

DB08752 1 1.686205945 1.75379047 0.753349628 0.1585137 1 0.12201228 1

DB08755 1 1.798410489 1.75379047 0.753349628 0.1585137 1 0.12201228 1

DB08756 1.5 1.78973833 1.753692879 0.367879736 0.245220308 1 0.346299761 1

DB08760 1 1.108824289 1.75379047 0.753349628 0.1585137 1 0.12201228 1

DB08762 2 2.044569097 1.75379047 0.753349628 0.628097886 1 0.645919359 1

DB08763 2 1.268180302 1.75379047 0.753349628 0.628097886 1 0.645919359 1

DB08765 2 2.383018351 1.745287633 0.542671379 0.680596551 1 0.645919359 1

DB08766 2 2.554820367 1.75379047 0.753349628 0.628097886 1 0.645919359 1

DB08768 1 1.892846141 1.75379047 0.753349628 0.1585137 1 0.12201228 1

DB08770 1 1.481109242 1.75379047 0.753349628 0.1585137 1 0.12201228 1

DB08771 2 1.773722761 1.75379047 0.753349628 0.628097886 1 0.645919359 1

DB08772 2 1.770870284 1.745287633 0.542671379 0.680596551 1 0.645919359 1

DB08773 1 3.121108193 1.745287633 0.542671379 0.08481895 1 0.12201228 1

DB08774 2 1.862209093 1.75379047 0.753349628 0.628097886 1 0.645919359 1

DB08775 2 2.080601164 1.75379047 0.753349628 0.628097886 1 0.645919359 1

DB08776 2 1.481084814 1.75379047 0.753349628 0.628097886 1 0.645919359 1

DB08777 2 2.205024085 1.75379047 0.753349628 0.628097886 1 0.645919359 1

DB08778 2 1.42862532 1.75379047 0.753349628 0.628097886 1 0.645919359 1

DB08779 2 0.624770152 1.75379047 0.753349628 0.628097886 1 0.645919359 1

DB08780 2 2.410814717 1.75379047 0.753349628 0.628097886 1 0.645919359 1

DB08781 2 2.789934404 1.75379047 0.753349628 0.628097886 1 0.645919359 1

DB08782 2 2.352829764 1.75379047 0.753349628 0.628097886 1 0.645919359 1

DB08783 1 3.353663999 1.75379047 0.753349628 0.1585137 1 0.12201228 1

DB08784 2 1.477080765 1.75379047 0.753349628 0.628097886 1 0.645919359 1

DB08786 1 0.187217907 1.75379047 0.753349628 0.1585137 1 0.12201228 1

DB08787 1 1.45321964 1.75379047 0.753349628 0.1585137 1 0.12201228 1

DB08788 1 1.741182924 1.75379047 0.753349628 0.1585137 1 0.12201228 1

DB08789 1 1.732682526 1.75379047 0.753349628 0.1585137 1 0.12201228 1

DB08790 1 3.057174669 1.75379047 0.753349628 0.1585137 1 0.12201228 1

DB08791 1 1.696578187 1.75379047 0.753349628 0.1585137 1 0.12201228 1

DB08799 1 1.105710536 1.75379047 0.753349628 0.1585137 1 0.12201228 1

DB08800 1 2.92747105 1.75379047 0.753349628 0.1585137 1 0.12201228 1

DB08801 1 0.923096115 1.745287633 0.542671379 0.08481895 1 0.12201228 1

DB08802 1 0.386807855 1.75379047 0.753349628 0.1585137 1 0.12201228 1

DB08804 1 3.72090964 1.75379047 0.753349628 0.1585137 1 0.12201228 1

DB08805 2 1.358650364 1.75379047 0.753349628 0.628097886 1 0.645919359 1

DB08806 2 2.140421861 1.75379047 0.753349628 0.628097886 1 0.645919359 1

DB08807 1.4 1.574112316 1.757979426 0.337271213 0.14425424 1 0.291413148 1

DB08808 1.6666667 1.82851796 1.757244872 0.464542708 0.422702925 1 0.444812957 1

DB08809 1 1.813584662 1.75379047 0.753349628 0.1585137 1 0.12201228 1

DB08810 1.3333333 1.442028709 1.757244872 0.464542708 0.180743525 1 0.257238652 1

DB08811 1 1.028373749 1.757244872 0.464542708 0.051541591 1 0.12201228 1

DB08813 1.3333333 1.607013684 1.757244872 0.464542708 0.180743525 1 0.257238652 1

DB08814 1.3333333 1.260715343 1.757244872 0.464542708 0.180743525 1 0.257238652 1

DB08815 1 1.367400619 1.770629523 0.307115021 0.006049327 1 0.12201228 1

DB08816 1 1.687960753 1.75379047 0.753349628 0.1585137 1 0.12201228 1

DB08818 1.6153846 1.808786886 1.754873752 0.200318376 0.243108818 1 0.413824896 1

DB08819 1 2.217540601 1.75379047 0.753349628 0.1585137 1 0.12201228 1

DB08820 1 0.469190038 1.75379047 0.753349628 0.1585137 1 0.12201228 1

DB08822 1 1.766605714 1.75379047 0.753349628 0.1585137 1 0.12201228 1

DB08828 2 1.203552731 1.75379047 0.753349628 0.628097886 1 0.645919359 1

DB08835 1 1.996321489 1.75379047 0.753349628 0.1585137 1 0.12201228 1

DB08836 1 2.081165716 1.75379047 0.753349628 0.1585137 1 0.12201228 1

DB08837 1.5 1.615206995 1.745287633 0.542671379 0.325634379 1 0.346299761 1

DB08838 1.5714286 2.02183001 1.736866356 0.292454388 0.285803159 1 0.387676968 1

DB08839 1.25 1.985188413 1.753692879 0.367879736 0.085471804 1 0.217648936 1

DB08846 1.7333333 1.690063281 1.760312929 0.204781867 0.447591756 1 0.48557866 1

DB08848 2 2.065076566 1.753692879 0.367879736 0.748421796 1 0.645919359 1

DB08855 1.3333333 1.442806687 1.757244872 0.464542708 0.180743525 1 0.257238652 1

DB08860 1.5 1.76915687 1.745287633 0.542671379 0.325634379 1 0.346299761 1

DB08862 1 1.896364018 1.757979426 0.337271213 0.012307606 1 0.12201228 1

DB08864 1 1.34629357 1.75379047 0.753349628 0.1585137 1 0.12201228 1

DB08865 1 1.938169824 1.745287633 0.542671379 0.08481895 1 0.12201228 1

DB08867 1 1.617757039 1.757244872 0.464542708 0.051541591 1 0.12201228 1

DB08868 1 1.749511214 1.757979426 0.337271213 0.012307606 1 0.12201228 1

DB08869 2 2.199153301 1.75379047 0.753349628 0.628097886 1 0.645919359 1

DB08870 1 1.624068309 1.75379047 0.753349628 0.1585137 1 0.12201228 1

DB08871 1.5 1.257432881 1.745287633 0.542671379 0.325634379 1 0.346299761 1

DB08872 2 1.897576031 1.745287633 0.542671379 0.680596551 1 0.645919359 1

DB08875 1 1.9567487 1.757244872 0.464542708 0.051541591 1 0.12201228 1

DB08877 1 1.360272949 1.745287633 0.542671379 0.08481895 1 0.12201228 1

DB08879 1 2.253125955 1.75379047 0.753349628 0.1585137 1 0.12201228 1

DB08880 2 1.863557734 1.75379047 0.753349628 0.628097886 1 0.645919359 1

DB08882 1 0.785022739 1.75379047 0.753349628 0.1585137 1 0.12201228 1

DB08883 2 2.787993449 1.75379047 0.753349628 0.628097886 1 0.645919359 1

DB08885 1.5 2.236217758 1.745287633 0.542671379 0.325634379 1 0.346299761 1

DB08888 1 2.039421368 1.757244872 0.464542708 0.051541591 1 0.12201228 1

DB08889 -0.1254288 1.689926912 1.770629523 0.307115021 3.33E-10 1.82E-06 0.001881508 1

DB08890 -5.1357984 0.536497776 1.75379047 0.753349628 2.97E-20 1.63E-16 1.34E-26 7.34E-23

DB08891 1 2.005888612 1.745287633 0.542671379 0.08481895 1 0.12201228 1

DB08892 1 1.730757016 1.745287633 0.542671379 0.08481895 1 0.12201228 1

DB08893 1 -0.175717588 1.75379047 0.753349628 0.1585137 1 0.12201228 1

DB08894 1 2.866972382 1.75379047 0.753349628 0.1585137 1 0.12201228 1

DB08895 1 1.577119913 1.753692879 0.367879736 0.020243381 1 0.12201228 1

DB08896 1.0588235 1.741421338 1.748450254 0.194078018 0.00019018 1 0.141313103 1

DB08897 1 1.713148812 1.757979426 0.337271213 0.012307606 1 0.12201228 1

DB08899 1 1.553865057 1.75379047 0.753349628 0.1585137 1 0.12201228 1

DB08900 2 1.302774066 1.75379047 0.753349628 0.628097886 1 0.645919359 1

DB08901 0.5287716 1.8028316 1.760312929 0.204781867 9.05E-10 4.94E-06 0.029355301 1

DB08904 1 2.668989873 1.75379047 0.753349628 0.1585137 1 0.12201228 1

DB08906 1.3333333 1.755037471 1.757244872 0.464542708 0.180743525 1 0.257238652 1

DB08907 2 1.980367315 1.75379047 0.753349628 0.628097886 1 0.645919359 1

DB08908 1 1.702460917 1.745287633 0.542671379 0.08481895 1 0.12201228 1

DB08910 1 1.598814748 1.757244872 0.464542708 0.051541591 1 0.12201228 1

DB08911 1 1.470423047 1.745287633 0.542671379 0.08481895 1 0.12201228 1

DB08912 1.5 1.397231091 1.753692879 0.367879736 0.245220308 1 0.346299761 1

DB08915 1 2.210394734 1.757244872 0.464542708 0.051541591 1 0.12201228 1

DB08916 1 2.128510415 1.757244872 0.464542708 0.051541591 1 0.12201228 1

DB08918 1 1.086188732 1.745287633 0.542671379 0.08481895 1 0.12201228 1

DB08922 1 2.104169652 1.761473636 0.259363203 0.001662717 1 0.12201228 1

DB08927 1 1.474797213 1.75379047 0.753349628 0.1585137 1 0.12201228 1

DB08931 1 2.137108004 1.75379047 0.753349628 0.1585137 1 0.12201228 1

DB08932 1 1.755012423 1.745287633 0.542671379 0.08481895 1 0.12201228 1

DB08935 1 1.453403321 1.75379047 0.753349628 0.1585137 1 0.12201228 1

DB08936 1 1.794549855 1.75379047 0.753349628 0.1585137 1 0.12201228 1

DB08949 1 2.015245907 1.745287633 0.542671379 0.08481895 1 0.12201228 1

DB08950 1 1.464836456 1.75379047 0.753349628 0.1585137 1 0.12201228 1

DB08954 2 1.847540493 1.745287633 0.542671379 0.680596551 1 0.645919359 1

DB08960 2 1.393763784 1.75379047 0.753349628 0.628097886 1 0.645919359 1

DB08964 1.5 1.216386101 1.745287633 0.542671379 0.325634379 1 0.346299761 1

DB08995 1 2.22364247 1.75379047 0.753349628 0.1585137 1 0.12201228 1

DB08996 2 2.476550062 1.75379047 0.753349628 0.628097886 1 0.645919359 1

DB09006 1 2.305521668 1.757244872 0.464542708 0.051541591 1 0.12201228 1

DB09013 2 2.625650044 1.745287633 0.542671379 0.680596551 1 0.645919359 1

DB09014 1.3333333 1.135549011 1.757244872 0.464542708 0.180743525 1 0.257238652 1

DB09016 1 2.050131137 1.757244872 0.464542708 0.051541591 1 0.12201228 1

DB09017 2 1.876046845 1.759334798 0.191532594 0.895536882 1 0.645919359 1

DB09018 1 2.745937788 1.75379047 0.753349628 0.1585137 1 0.12201228 1

DB09026 2 1.723746909 1.75379047 0.753349628 0.628097886 1 0.645919359 1

DB09028 2 1.488510309 1.757979426 0.337271213 0.763493223 1 0.645919359 1

DB09030 1 2.4418609 1.75379047 0.753349628 0.1585137 1 0.12201228 1

DB09031 1 1.364051262 1.75379047 0.753349628 0.1585137 1 0.12201228 1

DB09033 -2.2506291 2.201121336 1.745287633 0.542671379 8.96E-14 4.90E-10 3.44E-10 1.88E-06

DB09034 1 2.240451934 1.745287633 0.542671379 0.08481895 1 0.12201228 1

DB09035 1 1.970464643 1.75379047 0.753349628 0.1585137 1 0.12201228 1

DB09036 1 1.929609831 1.75379047 0.753349628 0.1585137 1 0.12201228 1

DB09037 1 2.394482396 1.75379047 0.753349628 0.1585137 1 0.12201228 1

DB09038 2 1.370858351 1.75379047 0.753349628 0.628097886 1 0.645919359 1

DB09039 1 2.710653732 1.75379047 0.753349628 0.1585137 1 0.12201228 1

DB09043 2 2.154421605 1.75379047 0.753349628 0.628097886 1 0.645919359 1

DB09045 2 2.089003964 1.75379047 0.753349628 0.628097886 1 0.645919359 1

DB09046 1 2.864015769 1.75379047 0.753349628 0.1585137 1 0.12201228 1

DB09047 2 0.860069199 1.75379047 0.753349628 0.628097886 1 0.645919359 1

DB09048 1 1.716479933 1.75379047 0.753349628 0.1585137 1 0.12201228 1

DB09049 1 1.580628537 1.75379047 0.753349628 0.1585137 1 0.12201228 1

DB09052 1 3.203415289 1.745287633 0.542671379 0.08481895 1 0.12201228 1

DB09053 1 2.393833543 1.75379047 0.753349628 0.1585137 1 0.12201228 1

DB09054 -5.4971682 0.496389069 1.75379047 0.753349628 3.14E-22 1.72E-18 2.99E-29 1.64E-25

DB09059 1 2.72197587 1.757244872 0.464542708 0.051541591 1 0.12201228 1

DB09061 1.1646277 1.85412077 1.760376084 0.119854867 3.34E-07 0.001816666 0.180996849 1

DB09063 1 3.641293639 1.75379047 0.753349628 0.1585137 1 0.12201228 1

DB09064 1 2.773675885 1.75379047 0.753349628 0.1585137 1 0.12201228 1

DB09067 2 2.121512673 1.75379047 0.753349628 0.628097886 1 0.645919359 1

DB09068 1.1428571 1.646455925 1.736866356 0.292454388 0.021121533 1 0.172307877 1

DB09069 1 1.623142036 1.75379047 0.753349628 0.1585137 1 0.12201228 1

DB09070 1 1.598682287 1.75379047 0.753349628 0.1585137 1 0.12201228 1

DB09071 1 2.116804946 1.745287633 0.542671379 0.08481895 1 0.12201228 1

DB09073 1 1.245131414 1.745287633 0.542671379 0.08481895 1 0.12201228 1

DB09074 2 1.184297231 1.757244872 0.464542708 0.699362524 1 0.645919359 1

DB09075 2 1.937879992 1.75379047 0.753349628 0.628097886 1 0.645919359 1

DB09076 1 0.848160218 1.757979426 0.337271213 0.012307606 1 0.12201228 1

DB09078 1 1.600871913 1.759693587 0.247672276 0.001079887 1 0.12201228 1

DB09079 0.4109645 1.641690759 1.763093512 0.211303302 7.82E-11 4.27E-07 0.01914689 1

DB09080 2 1.559723853 1.75379047 0.753349628 0.628097886 1 0.645919359 1

DB09082 2 2.248947588 1.75379047 0.753349628 0.628097886 1 0.645919359 1

DB09083 2 1.852187402 1.75379047 0.753349628 0.628097886 1 0.645919359 1

DB09085 1.7777778 1.702101502 1.753207263 0.264393406 0.537021102 1 0.512866521 1

DB09086 1.25 1.887940218 1.753692879 0.367879736 0.085471804 1 0.217648936 1

DB09088 2 1.425173536 1.736866356 0.292454388 0.815871351 1 0.645919359 1

DB09089 1.6521739 1.558964081 1.750829326 0.165162414 0.275145637 1 0.436012625 1

DB09090 1 1.009005695 1.75379047 0.753349628 0.1585137 1 0.12201228 1

DB09091 1 1.71924332 1.745287633 0.542671379 0.08481895 1 0.12201228 1

DB09092 1.8333333 1.991718395 1.770629523 0.307115021 0.580889804 1 0.546876824 1

DB09095 1 1.354248088 1.745287633 0.542671379 0.08481895 1 0.12201228 1

DB09097 1.6666667 1.423703267 1.757244872 0.464542708 0.422702925 1 0.444812957 1

DB09099 1 2.076298172 1.757979426 0.337271213 0.012307606 1 0.12201228 1

DB09100 1 1.574398662 1.745287633 0.542671379 0.08481895 1 0.12201228 1

DB09103 1 2.103424534 1.75379047 0.753349628 0.1585137 1 0.12201228 1

DB09105 1 1.802810032 1.75379047 0.753349628 0.1585137 1 0.12201228 1

DB09107 1 2.26405079 1.75379047 0.753349628 0.1585137 1 0.12201228 1

DB09108 1 1.505024207 1.75379047 0.753349628 0.1585137 1 0.12201228 1

DB09109 1.3333333 1.526438526 1.757244872 0.464542708 0.180743525 1 0.257238652 1

DB09112 1.6666667 1.459162297 1.757244872 0.464542708 0.422702925 1 0.444812957 1

DB09116 2 1.253419875 1.75379047 0.753349628 0.628097886 1 0.645919359 1

DB09118 2 1.784643729 1.755399642 0.180590294 0.912203427 1 0.645919359 1

DB09119 1 1.438083248 1.75379047 0.753349628 0.1585137 1 0.12201228 1

DB09120 1 0.186041117 1.75379047 0.753349628 0.1585137 1 0.12201228 1

DB09121 1 1.725283743 1.757244872 0.464542708 0.051541591 1 0.12201228 1

DB09123 1 2.534722307 1.745287633 0.542671379 0.08481895 1 0.12201228 1

DB09124 1 0.853331438 1.75379047 0.753349628 0.1585137 1 0.12201228 1

DB09126 2 2.044297201 1.75379047 0.753349628 0.628097886 1 0.645919359 1

DB09128 1 2.531751593 1.757979426 0.337271213 0.012307606 1 0.12201228 1

DB09129 1 2.089182542 1.75379047 0.753349628 0.1585137 1 0.12201228 1

DB09130 1.4406432 1.719089996 1.757659223 0.065149043 5.69E-07 0.003097335 0.313236014 1

DB09131 1.5 2.376477694 1.745287633 0.542671379 0.325634379 1 0.346299761 1

DB09140 1.5 1.608381099 1.745287633 0.542671379 0.325634379 1 0.346299761 1

DB09141 2 1.606783474 1.745287633 0.542671379 0.680596551 1 0.645919359 1

DB09143 2 1.833674312 1.75379047 0.753349628 0.628097886 1 0.645919359 1

DB09147 2 1.897118778 1.757244872 0.464542708 0.699362524 1 0.645919359 1

DB09148 1 2.584891712 1.75379047 0.753349628 0.1585137 1 0.12201228 1

DB09149 1 1.133709082 1.75379047 0.753349628 0.1585137 1 0.12201228 1

DB09151 1 2.909001735 1.75379047 0.753349628 0.1585137 1 0.12201228 1

DB09166 1.9411765 1.612932601 1.748450254 0.194078018 0.839653527 1 0.611710091 1

DB09167 1 1.556941953 1.759334798 0.191532594 3.68E-05 0.199524277 0.12201228 1

DB09173 1 1.85361647 1.757244872 0.464542708 0.051541591 1 0.12201228 1

DB09185 1 0.555488045 1.75379047 0.753349628 0.1585137 1 0.12201228 1

DB09186 1 1.901152567 1.75379047 0.753349628 0.1585137 1 0.12201228 1

DB09194 1 1.978256965 1.773663076 0.187305714 1.81E-05 0.098319689 0.12201228 1

DB09195 1 1.358870296 1.759693587 0.247672276 0.001079887 1 0.12201228 1

DB09198 1 1.124700776 1.75379047 0.753349628 0.1585137 1 0.12201228 1

DB09201 1 2.401927848 1.75379047 0.753349628 0.1585137 1 0.12201228 1

DB09202 1 1.575787289 1.770629523 0.307115021 0.006049327 1 0.12201228 1

DB09203 1 2.246043942 1.757244872 0.464542708 0.051541591 1 0.12201228 1

DB09204 1.4 1.683472286 1.757979426 0.337271213 0.14425424 1 0.291413148 1

DB09205 1 1.92035782 1.770629523 0.307115021 0.006049327 1 0.12201228 1

DB09207 1 2.690787168 1.745287633 0.542671379 0.08481895 1 0.12201228 1

DB09209 1 1.959953167 1.757244872 0.464542708 0.051541591 1 0.12201228 1

DB09211 1.25 1.403983043 1.753692879 0.367879736 0.085471804 1 0.217648936 1

DB09212 1.5 1.206066222 1.745287633 0.542671379 0.325634379 1 0.346299761 1

DB09213 1.4166667 1.891782275 1.763093512 0.211303302 0.050557023 1 0.30027637 1

DB09214 1.5 1.436363721 1.745287633 0.542671379 0.325634379 1 0.346299761 1

DB09215 1.5 2.260419268 1.745287633 0.542671379 0.325634379 1 0.346299761 1

DB09216 1.5 2.362662156 1.745287633 0.542671379 0.325634379 1 0.346299761 1

DB09217 1 1.406714095 1.75379047 0.753349628 0.1585137 1 0.12201228 1

DB09220 2 2.076318813 1.75379047 0.753349628 0.628097886 1 0.645919359 1

DB09221 1 1.741904517 1.761473636 0.259363203 0.001662717 1 0.12201228 1

DB09223 1 2.533041293 1.757244872 0.464542708 0.051541591 1 0.12201228 1

DB09224 1 2.775768368 1.75379047 0.753349628 0.1585137 1 0.12201228 1

DB09225 1.375 1.460454018 1.761473636 0.259363203 0.068100732 1 0.278352103 1

DB09227 1 0.476367912 1.75379047 0.753349628 0.1585137 1 0.12201228 1

DB09228 1.7142857 1.615316084 1.736866356 0.292454388 0.4692279 1 0.473897096 1

DB09229 1.2 1.671080152 1.759693587 0.247672276 0.01191636 1 0.195688273 1

DB09230 2 0.860990856 1.75379047 0.753349628 0.628097886 1 0.645919359 1

DB09231 1.8461538 1.599796133 1.754873752 0.200318376 0.675688099 1 0.554685855 1

DB09232 1.8 2.079015924 1.759693587 0.247672276 0.564638785 1 0.52649583 1

DB09235 1.5 1.782866317 1.745287633 0.542671379 0.325634379 1 0.346299761 1

DB09236 1.7777778 1.554869522 1.753207263 0.264393406 0.537021102 1 0.512866521 1

DB09237 1.25 2.122515868 1.753692879 0.367879736 0.085471804 1 0.217648936 1

DB09238 1.8333333 1.980526289 1.763093512 0.211303302 0.630210974 1 0.546876824 1

DB09239 1 1.766997766 1.75379047 0.753349628 0.1585137 1 0.12201228 1

DB09241 1 1.834546601 1.745287633 0.542671379 0.08481895 1 0.12201228 1

DB09242 1.5 1.514465358 1.745287633 0.542671379 0.325634379 1 0.346299761 1

DB09244 1 1.968086485 1.757244872 0.464542708 0.051541591 1 0.12201228 1

DB09245 1 1.178797469 1.75379047 0.753349628 0.1585137 1 0.12201228 1

DB09256 1 1.08436766 1.75379047 0.753349628 0.1585137 1 0.12201228 1

DB09257 2 2.087008554 1.75379047 0.753349628 0.628097886 1 0.645919359 1

DB09258 1.6666667 1.22115543 1.757244872 0.464542708 0.422702925 1 0.444812957 1

DB09262 1 1.012263676 1.757244872 0.464542708 0.051541591 1 0.12201228 1

DB09265 2 1.836607464 1.75379047 0.753349628 0.628097886 1 0.645919359 1

DB09266 1 3.047637134 1.75379047 0.753349628 0.1585137 1 0.12201228 1

DB09270 1.5 1.953242375 1.745287633 0.542671379 0.325634379 1 0.346299761 1

DB09272 1 1.201231543 1.757244872 0.464542708 0.051541591 1 0.12201228 1

DB09273 1.5 1.524419698 1.745287633 0.542671379 0.325634379 1 0.346299761 1

DB09279 1 2.509080001 1.75379047 0.753349628 0.1585137 1 0.12201228 1

DB09280 1 1.10088671 1.75379047 0.753349628 0.1585137 1 0.12201228 1

DB09282 1 2.03994959 1.75379047 0.753349628 0.1585137 1 0.12201228 1

DB09283 1.0526316 1.80933457 1.755240506 0.172383815 2.29E-05 0.124455671 0.139189107 1

DB09285 1.25 1.899047745 1.753692879 0.367879736 0.085471804 1 0.217648936 1

DB09286 1.1 2.244735683 1.759693587 0.247672276 0.003865763 1 0.155995064 1

DB09288 1.25 1.780777666 1.753692879 0.367879736 0.085471804 1 0.217648936 1

DB09289 1.25 1.786008173 1.753692879 0.367879736 0.085471804 1 0.217648936 1

DB09290 1 2.263731551 1.75379047 0.753349628 0.1585137 1 0.12201228 1

DB09291 1 2.796101824 1.75379047 0.753349628 0.1585137 1 0.12201228 1

DB09292 1 3.195167477 1.75379047 0.753349628 0.1585137 1 0.12201228 1

DB09295 1.6666667 1.383846332 1.757244872 0.464542708 0.422702925 1 0.444812957 1

DB09300 1 2.199524602 1.745287633 0.542671379 0.08481895 1 0.12201228 1

DB09301 1 2.450030977 1.757244872 0.464542708 0.051541591 1 0.12201228 1

DB09302 2 1.37341092 1.75379047 0.753349628 0.628097886 1 0.645919359 1

DB09304 1.3157895 1.789172772 1.755240506 0.172383815 0.005397603 1 0.248604609 1

DB09310 1 2.42838565 1.75379047 0.753349628 0.1585137 1 0.12201228 1

DB09317 1 2.862676032 1.75379047 0.753349628 0.1585137 1 0.12201228 1

DB09318 1 1.05424203 1.75379047 0.753349628 0.1585137 1 0.12201228 1

DB09327 1.5 2.580231428 1.745287633 0.542671379 0.325634379 1 0.346299761 1

DB09329 1 2.110115525 1.75379047 0.753349628 0.1585137 1 0.12201228 1

DB09330 1 2.009756542 1.75379047 0.753349628 0.1585137 1 0.12201228 1

DB09331 1 1.706936201 1.75379047 0.753349628 0.1585137 1 0.12201228 1

DB09332 1.4285714 1.503986854 1.736866356 0.292454388 0.145903866 1 0.306680831 1

DB09333 1 3.078989801 1.75379047 0.753349628 0.1585137 1 0.12201228 1

DB09334 2 0.858739769 1.75379047 0.753349628 0.628097886 1 0.645919359 1

DB09336 1 1.746666551 1.745287633 0.542671379 0.08481895 1 0.12201228 1

DB09338 1.3333333 2.341588108 1.757244872 0.464542708 0.180743525 1 0.257238652 1

DB09342 2 1.681698882 1.736866356 0.292454388 0.815871351 1 0.645919359 1

DB09343 2 1.4875967 1.75379047 0.753349628 0.628097886 1 0.645919359 1

DB09345 2 1.748230247 1.736866356 0.292454388 0.815871351 1 0.645919359 1

DB09351 2 -0.061161156 1.75379047 0.753349628 0.628097886 1 0.645919359 1

DB09369 1 1.730717466 1.75379047 0.753349628 0.1585137 1 0.12201228 1

DB09371 1 1.192347312 1.757244872 0.464542708 0.051541591 1 0.12201228 1

DB09389 1.3333333 2.048276478 1.757244872 0.464542708 0.180743525 1 0.257238652 1

DB09401 1.3333333 1.669238934 1.757244872 0.464542708 0.180743525 1 0.257238652 1

DB09409 1.8571429 1.846692854 1.736866356 0.292454388 0.659561248 1 0.56136264 1

DB09418 1 3.094910771 1.75379047 0.753349628 0.1585137 1 0.12201228 1

DB09421 1 2.188416388 1.75379047 0.753349628 0.1585137 1 0.12201228 1

DB09422 1 1.896706248 1.75379047 0.753349628 0.1585137 1 0.12201228 1

DB09460 2 1.3730647 1.753692879 0.367879736 0.748421796 1 0.645919359 1

DB09462 1.3 1.745114241 1.759693587 0.247672276 0.031722796 1 0.240967937 1

DB09472 2 1.825075159 1.745287633 0.542671379 0.680596551 1 0.645919359 1

DB09477 1 2.133592808 1.745287633 0.542671379 0.08481895 1 0.12201228 1

DB09481 1.8571429 2.074276814 1.736866356 0.292454388 0.659561248 1 0.56136264 1

DB09488 1 1.55416617 1.75379047 0.753349628 0.1585137 1 0.12201228 1

DB09517 2 2.51604431 1.757244872 0.464542708 0.699362524 1 0.645919359 1

DB09526 1 1.53135924 1.75379047 0.753349628 0.1585137 1 0.12201228 1

DB09532 2 2.222041449 1.75379047 0.753349628 0.628097886 1 0.645919359 1

DB09535 1 1.353780206 1.745287633 0.542671379 0.08481895 1 0.12201228 1

DB09536 1 1.278892067 1.75379047 0.753349628 0.1585137 1 0.12201228 1

DB09539 1 2.263594554 1.75379047 0.753349628 0.1585137 1 0.12201228 1

DB09552 1 0.565061786 1.75379047 0.753349628 0.1585137 1 0.12201228 1

DB09555 1 1.791264729 1.75379047 0.753349628 0.1585137 1 0.12201228 1

DB09559 1 0.819200806 1.75379047 0.753349628 0.1585137 1 0.12201228 1

DB09564 1 1.656703453 1.75379047 0.753349628 0.1585137 1 0.12201228 1

DB09568 1.8 1.632999028 1.757979426 0.337271213 0.549575873 1 0.52649583 1

DB10770 1.1 1.654255961 1.759693587 0.247672276 0.003865763 1 0.155995064 1

DB10772 1.1 1.483726859 1.759693587 0.247672276 0.003865763 1 0.155995064 1

DB11059 1 1.868484517 1.75379047 0.753349628 0.1585137 1 0.12201228 1

DB11064 1 0.824640483 1.757244872 0.464542708 0.051541591 1 0.12201228 1

DB11071 1.5 1.768693574 1.745287633 0.542671379 0.325634379 1 0.346299761 1

DB11075 1 2.094268908 1.75379047 0.753349628 0.1585137 1 0.12201228 1

DB11077 2 1.566211905 1.75379047 0.753349628 0.628097886 1 0.645919359 1

DB11079 1.5 1.096524091 1.745287633 0.542671379 0.325634379 1 0.346299761 1

DB11081 2 2.747763773 1.75379047 0.753349628 0.628097886 1 0.645919359 1

DB11085 1 2.333078876 1.75379047 0.753349628 0.1585137 1 0.12201228 1

DB11087 1 2.330189023 1.75379047 0.753349628 0.1585137 1 0.12201228 1

DB11090 1 1.989091364 1.75379047 0.753349628 0.1585137 1 0.12201228 1

DB11093 1.6 1.696357375 1.760507724 0.1309174 0.110095039 1 0.404623142 1

DB11094 1.5 1.78799352 1.745287633 0.542671379 0.325634379 1 0.346299761 1

DB11113 1 1.860858036 1.745287633 0.542671379 0.08481895 1 0.12201228 1

DB11120 1 0.920751634 1.75379047 0.753349628 0.1585137 1 0.12201228 1

DB11124 1.2222222 2.047324323 1.753207263 0.264393406 0.022304951 1 0.205277671 1

DB11130 1 1.224092821 1.757244872 0.464542708 0.051541591 1 0.12201228 1

DB11131 1 2.161438843 1.75379047 0.753349628 0.1585137 1 0.12201228 1

DB11132 1 0.78526296 1.75379047 0.753349628 0.1585137 1 0.12201228 1

DB11133 1 2.15638207 1.757244872 0.464542708 0.051541591 1 0.12201228 1

DB11136 1 0.779583982 1.75379047 0.753349628 0.1585137 1 0.12201228 1

DB11148 1.9090909 1.978396617 1.751829593 0.13569543 0.876757344 1 0.592658552 1

DB11156 1 1.801412894 1.745287633 0.542671379 0.08481895 1 0.12201228 1

DB11157 2 2.059021366 1.757244872 0.464542708 0.699362524 1 0.645919359 1

DB11166 1.5 0.911183841 1.745287633 0.542671379 0.325634379 1 0.346299761 1

DB11181 1 1.837286016 1.757979426 0.337271213 0.012307606 1 0.12201228 1

DB11186 1.7272727 1.945587976 1.759747174 0.221112233 0.441617815 1 0.481859977 1

DB11193 1 1.785644331 1.75379047 0.753349628 0.1585137 1 0.12201228 1

DB11201 1.5 2.362984001 1.745287633 0.542671379 0.325634379 1 0.346299761 1

DB11217 1 2.042458754 1.75379047 0.753349628 0.1585137 1 0.12201228 1

DB11219 1 2.115576992 1.753692879 0.367879736 0.020243381 1 0.12201228 1

DB11221 1 2.362351618 1.75379047 0.753349628 0.1585137 1 0.12201228 1

DB11235 1 1.932331389 1.770629523 0.307115021 0.006049327 1 0.12201228 1

DB11254 1.3333333 1.0459528 1.757244872 0.464542708 0.180743525 1 0.257238652 1

DB11273 1.32 1.572001041 1.76622404 0.151326267 0.001595286 1 0.250662597 1

DB11274 1.5 1.475967997 1.753692879 0.367879736 0.245220308 1 0.346299761 1

DB11275 1.4 1.364619316 1.757979426 0.337271213 0.14425424 1 0.291413148 1

DB11278 1.2222222 1.341915334 1.753207263 0.264393406 0.022304951 1 0.205277671 1

DB11294 1 1.567376022 1.757979426 0.337271213 0.012307606 1 0.12201228 1

DB11300 1.1111111 1.527655663 1.753207263 0.264393406 0.007579386 1 0.160123241 1

DB11311 1 1.628884862 1.757244872 0.464542708 0.051541591 1 0.12201228 1

DB11312 1 1.770794072 1.745287633 0.542671379 0.08481895 1 0.12201228 1

DB11323 1.5 1.752323233 1.745287633 0.542671379 0.325634379 1 0.346299761 1

DB11324 1 1.910287726 1.75379047 0.753349628 0.1585137 1 0.12201228 1

DB11327 1 2.796509664 1.745287633 0.542671379 0.08481895 1 0.12201228 1

DB11328 1 0.878774778 1.75379047 0.753349628 0.1585137 1 0.12201228 1

DB11338 1 1.527161135 1.753692879 0.367879736 0.020243381 1 0.12201228 1

DB11345 1.75 1.665588314 1.753692879 0.367879736 0.495995374 1 0.495810565 1

DB11348 1.6 1.642964697 1.760507724 0.1309174 0.110095039 1 0.404623142 1

DB11359 1 2.024794689 1.75379047 0.753349628 0.1585137 1 0.12201228 1

DB11362 2 2.178887756 1.75379047 0.753349628 0.628097886 1 0.645919359 1

DB11363 1 2.147924568 1.75379047 0.753349628 0.1585137 1 0.12201228 1

DB11365 1 2.426109517 1.75379047 0.753349628 0.1585137 1 0.12201228 1

DB11386 1 0.184749234 1.75379047 0.753349628 0.1585137 1 0.12201228 1

DB11397 1.5 1.893334336 1.745287633 0.542671379 0.325634379 1 0.346299761 1

DB11421 2 1.552499898 1.75379047 0.753349628 0.628097886 1 0.645919359 1

DB11429 1 1.375805764 1.75379047 0.753349628 0.1585137 1 0.12201228 1

DB11447 1 0.808315192 1.75379047 0.753349628 0.1585137 1 0.12201228 1

DB11477 1 1.510991619 1.757244872 0.464542708 0.051541591 1 0.12201228 1

DB11478 1 1.546773737 1.745287633 0.542671379 0.08481895 1 0.12201228 1

DB11496 1 2.941360931 1.75379047 0.753349628 0.1585137 1 0.12201228 1

DB11541 1 2.344965148 1.75379047 0.753349628 0.1585137 1 0.12201228 1

DB11560 2 0.266406477 1.745287633 0.542671379 0.680596551 1 0.645919359 1

DB11571 1.1428571 1.666858173 1.736866356 0.292454388 0.021121533 1 0.172307877 1

DB11572 1 1.964826527 1.753692879 0.367879736 0.020243381 1 0.12201228 1

DB11577 1 0.978538064 1.736866356 0.292454388 0.005874511 1 0.12201228 1

DB11580 1 1.970064643 1.75379047 0.753349628 0.1585137 1 0.12201228 1

DB11581 2 1.081467774 1.75379047 0.753349628 0.628097886 1 0.645919359 1

DB11582 1.9444444 1.876303902 1.755399642 0.180590294 0.85240771 1 0.613636316 1

DB11584 2 1.962383105 1.745287633 0.542671379 0.680596551 1 0.645919359 1

DB11587 2 1.343610153 1.75379047 0.753349628 0.628097886 1 0.645919359 1

DB11588 1 1.191323026 1.75379047 0.753349628 0.1585137 1 0.12201228 1

DB11590 1.6666667 2.061667908 1.757244872 0.464542708 0.422702925 1 0.444812957 1

DB11591 1 1.397304855 1.75379047 0.753349628 0.1585137 1 0.12201228 1

DB11595 1 1.08350894 1.75379047 0.753349628 0.1585137 1 0.12201228 1

DB11598 2 2.767359779 1.75379047 0.753349628 0.628097886 1 0.645919359 1

DB11601 1 0.607689235 1.75379047 0.753349628 0.1585137 1 0.12201228 1

DB11605 1.3333333 1.306549103 1.757244872 0.464542708 0.180743525 1 0.257238652 1

DB11606 1 2.823585049 1.75379047 0.753349628 0.1585137 1 0.12201228 1

DB11607 1 1.144332312 1.75379047 0.753349628 0.1585137 1 0.12201228 1

DB11611 1 4.172252134 1.75379047 0.753349628 0.1585137 1 0.12201228 1

DB11614 1 2.273403634 1.745287633 0.542671379 0.08481895 1 0.12201228 1

DB11619 1 1.307286606 1.770629523 0.307115021 0.006049327 1 0.12201228 1

DB11623 1 -0.171115455 1.75379047 0.753349628 0.1585137 1 0.12201228 1

DB11626 1 1.762503596 1.745287633 0.542671379 0.08481895 1 0.12201228 1

DB11632 1 1.037243631 1.75379047 0.753349628 0.1585137 1 0.12201228 1

DB11633 1.25 2.024485988 1.761473636 0.259363203 0.024302735 1 0.217648936 1

DB11635 1 1.632166749 1.75379047 0.753349628 0.1585137 1 0.12201228 1

DB11638 1.6 1.860419648 1.75858521 0.088890791 0.037208141 1 0.404623142 1

DB11639 2 2.5371328 1.745287633 0.542671379 0.680596551 1 0.645919359 1

DB11640 2 1.909850406 1.75379047 0.753349628 0.628097886 1 0.645919359 1

DB11641 1 1.479932063 1.75379047 0.753349628 0.1585137 1 0.12201228 1

DB11642 1 2.341166834 1.745287633 0.542671379 0.08481895 1 0.12201228 1

DB11644 2 3.080346607 1.75379047 0.753349628 0.628097886 1 0.645919359 1

DB11652 1 1.754738756 1.745287633 0.542671379 0.08481895 1 0.12201228 1

DB11653 1.4 2.103046748 1.757979426 0.337271213 0.14425424 1 0.291413148 1

DB11660 1 1.182020496 1.75379047 0.753349628 0.1585137 1 0.12201228 1

DB11672 1.2 1.528999864 1.757979426 0.337271213 0.049023756 1 0.195688273 1

DB11674 1 1.942302807 1.745287633 0.542671379 0.08481895 1 0.12201228 1

DB11689 1 1.892170517 1.745287633 0.542671379 0.08481895 1 0.12201228 1

DB11691 1 2.624815319 1.757244872 0.464542708 0.051541591 1 0.12201228 1

DB11692 1 2.047230981 1.75379047 0.753349628 0.1585137 1 0.12201228 1

DB11693 1.5 2.068910551 1.745287633 0.542671379 0.325634379 1 0.346299761 1

DB11699 1 1.340591661 1.75379047 0.753349628 0.1585137 1 0.12201228 1

DB11703 1 1.320629086 1.75379047 0.753349628 0.1585137 1 0.12201228 1

DB11712 1 1.508704937 1.75379047 0.753349628 0.1585137 1 0.12201228 1

DB11714 1 1.526964823 1.75379047 0.753349628 0.1585137 1 0.12201228 1

DB11718 1 2.448556285 1.75379047 0.753349628 0.1585137 1 0.12201228 1

DB11723 1 2.257439755 1.75379047 0.753349628 0.1585137 1 0.12201228 1

DB11726 2 3.654902616 1.75379047 0.753349628 0.628097886 1 0.645919359 1

DB11730 1 1.424755988 1.745287633 0.542671379 0.08481895 1 0.12201228 1

DB11731 1 2.390677733 1.745287633 0.542671379 0.08481895 1 0.12201228 1

DB11732 1 1.350464156 1.75379047 0.753349628 0.1585137 1 0.12201228 1

DB11737 1 1.862782258 1.75379047 0.753349628 0.1585137 1 0.12201228 1

DB11738 1 0.920041659 1.75379047 0.753349628 0.1585137 1 0.12201228 1

DB11740 2 2.472535684 1.75379047 0.753349628 0.628097886 1 0.645919359 1

DB11745 1 2.207113677 1.75379047 0.753349628 0.1585137 1 0.12201228 1

DB11752 1.125 2.041892272 1.761473636 0.259363203 0.007064127 1 0.165382999 1

DB11755 1.4285714 1.552694374 1.736866356 0.292454388 0.145903866 1 0.306680831 1

DB11757 1 2.44976721 1.745287633 0.542671379 0.08481895 1 0.12201228 1

DB11760 2 2.111283147 1.745287633 0.542671379 0.680596551 1 0.645919359 1

DB11761 1 1.37037528 1.75379047 0.753349628 0.1585137 1 0.12201228 1

DB11767 1 2.034930603 1.753692879 0.367879736 0.020243381 1 0.12201228 1

DB11772 1 0.519127134 1.75379047 0.753349628 0.1585137 1 0.12201228 1

DB11781 2 1.236550456 1.745287633 0.542671379 0.680596551 1 0.645919359 1

DB11791 1 1.648793109 1.75379047 0.753349628 0.1585137 1 0.12201228 1

DB11793 2 2.454936216 1.745287633 0.542671379 0.680596551 1 0.645919359 1

DB11811 1 1.219726597 1.75379047 0.753349628 0.1585137 1 0.12201228 1

DB11817 1 1.374740091 1.753692879 0.367879736 0.020243381 1 0.12201228 1

DB11823 1.8 2.090281348 1.759693587 0.247672276 0.564638785 1 0.52649583 1

DB11827 2 1.62539203 1.75379047 0.753349628 0.628097886 1 0.645919359 1

DB11828 1 1.050025883 1.75379047 0.753349628 0.1585137 1 0.12201228 1

DB11830 1.3333333 1.328598414 1.757244872 0.464542708 0.180743525 1 0.257238652 1

DB11831 1 1.373179267 1.75379047 0.753349628 0.1585137 1 0.12201228 1

DB11834 1 -0.311415258 1.75379047 0.753349628 0.1585137 1 0.12201228 1

DB11837 2 2.463781342 1.745287633 0.542671379 0.680596551 1 0.645919359 1

DB11842 1 1.877327101 1.75379047 0.753349628 0.1585137 1 0.12201228 1

DB11855 1 1.913664693 1.757979426 0.337271213 0.012307606 1 0.12201228 1

DB11858 1 1.434907916 1.745287633 0.542671379 0.08481895 1 0.12201228 1

DB11859 2 1.709102934 1.759334798 0.191532594 0.895536882 1 0.645919359 1

DB11866 2 1.180052491 1.75379047 0.753349628 0.628097886 1 0.645919359 1

DB11901 1.9411765 1.738374956 1.748450254 0.194078018 0.839653527 1 0.611710091 1

DB11914 1 2.474283661 1.75379047 0.753349628 0.1585137 1 0.12201228 1

DB11921 1 1.153361539 1.75379047 0.753349628 0.1585137 1 0.12201228 1

DB11936 2 1.366772749 1.75379047 0.753349628 0.628097886 1 0.645919359 1

DB11937 1 1.746316833 1.745287633 0.542671379 0.08481895 1 0.12201228 1

DB11942 1 2.130914593 1.75379047 0.753349628 0.1585137 1 0.12201228 1

DB11945 1 2.551972486 1.75379047 0.753349628 0.1585137 1 0.12201228 1

DB11948 2 1.020231836 1.75379047 0.753349628 0.628097886 1 0.645919359 1

DB11949 1 2.173912285 1.75379047 0.753349628 0.1585137 1 0.12201228 1

DB11951 1 1.463325544 1.745287633 0.542671379 0.08481895 1 0.12201228 1

DB11952 -2.2485841 1.269025327 1.745287633 0.542671379 9.22E-14 5.04E-10 3.51E-10 1.92E-06

DB11963 1 2.345321887 1.75379047 0.753349628 0.1585137 1 0.12201228 1

DB11967 1 2.070971623 1.757979426 0.337271213 0.012307606 1 0.12201228 1

DB11973 1.3333333 1.883062083 1.757244872 0.464542708 0.180743525 1 0.257238652 1

DB11978 2 1.358095766 1.75379047 0.753349628 0.628097886 1 0.645919359 1

DB11979 1 0.861785281 1.75379047 0.753349628 0.1585137 1 0.12201228 1

DB11986 1.6 1.842424516 1.757979426 0.337271213 0.319747599 1 0.404623142 1

DB11988 1 3.371060686 1.75379047 0.753349628 0.1585137 1 0.12201228 1

DB11994 1.7142857 1.512916837 1.736866356 0.292454388 0.4692279 1 0.473897096 1

DB11995 1.3333333 1.727501815 1.757244872 0.464542708 0.180743525 1 0.257238652 1

DB12001 1 1.932689777 1.745287633 0.542671379 0.08481895 1 0.12201228 1

DB12007 1 0.944328556 1.745287633 0.542671379 0.08481895 1 0.12201228 1

DB12010 1.4571645 1.818617585 1.755930518 0.045260353 2.04E-11 1.11E-07 0.322303521 1

DB12015 1 3.10086184 1.75379047 0.753349628 0.1585137 1 0.12201228 1

DB12023 1 0.840822165 1.75379047 0.753349628 0.1585137 1 0.12201228 1

DB12061 1 1.612989791 1.753692879 0.367879736 0.020243381 1 0.12201228 1

DB12077 1 0.955726599 1.75379047 0.753349628 0.1585137 1 0.12201228 1

DB12093 1.5 1.772510356 1.745287633 0.542671379 0.325634379 1 0.346299761 1

DB12095 1 2.193131402 1.745287633 0.542671379 0.08481895 1 0.12201228 1

DB12110 1 3.26562361 1.75379047 0.753349628 0.1585137 1 0.12201228 1

DB12116 1.3333333 2.28433831 1.757244872 0.464542708 0.180743525 1 0.257238652 1

DB12118 2 2.129382191 1.75379047 0.753349628 0.628097886 1 0.645919359 1

DB12119 1 1.101346737 1.75379047 0.753349628 0.1585137 1 0.12201228 1

DB12128 1 1.960226586 1.75379047 0.753349628 0.1585137 1 0.12201228 1

DB12129 1 0.74045784 1.75379047 0.753349628 0.1585137 1 0.12201228 1

DB12130 1 2.184845976 1.75379047 0.753349628 0.1585137 1 0.12201228 1

DB12140 1 1.573321574 1.757244872 0.464542708 0.051541591 1 0.12201228 1

DB12141 1.3684211 1.60280188 1.755240506 0.172383815 0.012418044 1 0.274963044 1

DB12147 1 2.096265208 1.753207263 0.264393406 0.002194136 1 0.12201228 1

DB12159 1 2.034033944 1.75379047 0.753349628 0.1585137 1 0.12201228 1

DB12163 1 0.762668259 1.745287633 0.542671379 0.08481895 1 0.12201228 1

DB12177 1 2.196520846 1.745287633 0.542671379 0.08481895 1 0.12201228 1

DB12191 2 0.849083782 1.75379047 0.753349628 0.628097886 1 0.645919359 1

DB12200 1 2.103397896 1.75379047 0.753349628 0.1585137 1 0.12201228 1

DB12202 1 1.368606318 1.75379047 0.753349628 0.1585137 1 0.12201228 1

DB12228 2 1.12122901 1.745287633 0.542671379 0.680596551 1 0.645919359 1

DB12240 -5.1416636 2.475729433 1.75379047 0.753349628 2.77E-20 1.51E-16 1.22E-26 6.66E-23

DB12248 2 1.204942852 1.75379047 0.753349628 0.628097886 1 0.645919359 1

DB12267 1 1.79082246 1.761473636 0.259363203 0.001662717 1 0.12201228 1

DB12271 2 1.227106904 1.745287633 0.542671379 0.680596551 1 0.645919359 1

DB12278 1 1.971226389 1.736866356 0.292454388 0.005874511 1 0.12201228 1

DB12285 1 1.079199245 1.75379047 0.753349628 0.1585137 1 0.12201228 1

DB12305 1 2.439710913 1.757244872 0.464542708 0.051541591 1 0.12201228 1

DB12307 1 1.953770669 1.745287633 0.542671379 0.08481895 1 0.12201228 1

DB12319 2 2.593288326 1.745287633 0.542671379 0.680596551 1 0.645919359 1

DB12323 1 0.917769202 1.75379047 0.753349628 0.1585137 1 0.12201228 1

DB12328 1 1.201404695 1.75379047 0.753349628 0.1585137 1 0.12201228 1

DB12332 2 1.967406905 1.757244872 0.464542708 0.699362524 1 0.645919359 1

DB12334 1 1.625175045 1.745287633 0.542671379 0.08481895 1 0.12201228 1

DB12339 2 2.949346587 1.745287633 0.542671379 0.680596551 1 0.645919359 1

DB12340 1.3333333 1.551883831 1.757244872 0.464542708 0.180743525 1 0.257238652 1

DB12364 2 1.165803255 1.75379047 0.753349628 0.628097886 1 0.645919359 1

DB12371 1 0.789158091 1.745287633 0.542671379 0.08481895 1 0.12201228 1

DB12379 1 1.73524794 1.745287633 0.542671379 0.08481895 1 0.12201228 1

DB12418 2 1.10694931 1.75379047 0.753349628 0.628097886 1 0.645919359 1

DB12436 1 2.973298826 1.75379047 0.753349628 0.1585137 1 0.12201228 1

DB12442 1 2.128014563 1.75379047 0.753349628 0.1585137 1 0.12201228 1

DB12445 2 1.56126679 1.75379047 0.753349628 0.628097886 1 0.645919359 1

DB12450 1 1.787293463 1.75379047 0.753349628 0.1585137 1 0.12201228 1

DB12457 2 1.293164537 1.75379047 0.753349628 0.628097886 1 0.645919359 1

DB12458 2 2.200987255 1.757244872 0.464542708 0.699362524 1 0.645919359 1

DB12465 1 -1.190279894 1.75379047 0.753349628 0.1585137 1 0.12201228 1

DB12478 1 0.719945722 1.745287633 0.542671379 0.08481895 1 0.12201228 1

DB12483 -2.2485841 1.880260583 1.745287633 0.542671379 9.22E-14 5.04E-10 3.51E-10 1.92E-06

DB12498 1 2.084849086 1.75379047 0.753349628 0.1585137 1 0.12201228 1

DB12500 1 1.549562785 1.757244872 0.464542708 0.051541591 1 0.12201228 1

DB12518 1 3.655174668 1.75379047 0.753349628 0.1585137 1 0.12201228 1

DB12520 1 1.157114554 1.75379047 0.753349628 0.1585137 1 0.12201228 1

DB12530 1 1.233132426 1.75379047 0.753349628 0.1585137 1 0.12201228 1

DB12532 1 1.772117848 1.75379047 0.753349628 0.1585137 1 0.12201228 1

DB12537 2 1.955571426 1.759334798 0.191532594 0.895536882 1 0.645919359 1

DB12548 1 1.422619301 1.745287633 0.542671379 0.08481895 1 0.12201228 1

DB12555 1 2.714921123 1.75379047 0.753349628 0.1585137 1 0.12201228 1

DB12565 1 0.93204126 1.75379047 0.753349628 0.1585137 1 0.12201228 1

DB12569 1 1.424520231 1.75379047 0.753349628 0.1585137 1 0.12201228 1

DB12579 1 0.843562542 1.75379047 0.753349628 0.1585137 1 0.12201228 1

DB12582 1 1.008094151 1.75379047 0.753349628 0.1585137 1 0.12201228 1

DB12589 1 1.131157352 1.75379047 0.753349628 0.1585137 1 0.12201228 1

DB12598 1.6666667 1.219147266 1.770629523 0.307115021 0.367487831 1 0.444812957 1

DB12610 2 1.938342164 1.75379047 0.753349628 0.628097886 1 0.645919359 1

DB12612 1 1.589256983 1.745287633 0.542671379 0.08481895 1 0.12201228 1

DB12637 1.5 1.911605793 1.745287633 0.542671379 0.325634379 1 0.346299761 1

DB12651 1 1.138121169 1.75379047 0.753349628 0.1585137 1 0.12201228 1

DB12662 1 2.383132766 1.75379047 0.753349628 0.1585137 1 0.12201228 1

DB12668 1 2.513138443 1.745287633 0.542671379 0.08481895 1 0.12201228 1

DB12670 1 1.436050601 1.75379047 0.753349628 0.1585137 1 0.12201228 1

DB12688 1.5 1.79430962 1.745287633 0.542671379 0.325634379 1 0.346299761 1

DB12693 1 1.629295211 1.75379047 0.753349628 0.1585137 1 0.12201228 1

DB12695 1.3589744 1.826047079 1.758692928 0.118260807 0.000362463 1 0.270132499 1

DB12698 1 1.563803599 1.757244872 0.464542708 0.051541591 1 0.12201228 1

DB12702 2 2.622057548 1.75379047 0.753349628 0.628097886 1 0.645919359 1

DB12731 1 0.646558245 1.75379047 0.753349628 0.1585137 1 0.12201228 1

DB12733 1 0.658112742 1.75379047 0.753349628 0.1585137 1 0.12201228 1

DB12742 1.1666667 1.601649748 1.770629523 0.307115021 0.024616471 1 0.181824474 1

DB12747 1 0.669071402 1.75379047 0.753349628 0.1585137 1 0.12201228 1

DB12773 1 1.67717578 1.75379047 0.753349628 0.1585137 1 0.12201228 1

DB12783 1 2.33536397 1.75379047 0.753349628 0.1585137 1 0.12201228 1

DB12789 1 2.285107745 1.75379047 0.753349628 0.1585137 1 0.12201228 1

DB12808 1.6666667 2.33420508 1.757244872 0.464542708 0.422702925 1 0.444812957 1

DB12831 1.3333333 1.689164674 1.757244872 0.464542708 0.180743525 1 0.257238652 1

DB12836 1 0.436979296 1.75379047 0.753349628 0.1585137 1 0.12201228 1

DB12845 1 1.084104794 1.75379047 0.753349628 0.1585137 1 0.12201228 1

DB12865 1 1.91314814 1.75379047 0.753349628 0.1585137 1 0.12201228 1

DB12872 1 2.179252407 1.745287633 0.542671379 0.08481895 1 0.12201228 1

DB12874 1 1.359166242 1.75379047 0.753349628 0.1585137 1 0.12201228 1

DB12887 1.5 1.323144455 1.745287633 0.542671379 0.325634379 1 0.346299761 1

DB12890 2 1.743268938 1.75379047 0.753349628 0.628097886 1 0.645919359 1

DB12893 2 1.135749464 1.757244872 0.464542708 0.699362524 1 0.645919359 1

DB12941 1 1.499072781 1.745287633 0.542671379 0.08481895 1 0.12201228 1

DB12960 1 1.562507171 1.75379047 0.753349628 0.1585137 1 0.12201228 1

DB12961 1 1.111066757 1.757244872 0.464542708 0.051541591 1 0.12201228 1

DB12965 1.6875 1.574685973 1.759334798 0.191532594 0.353810673 1 0.457510077 1

DB12978 1 2.470596771 1.753692879 0.367879736 0.020243381 1 0.12201228 1

DB13003 1 2.617977512 1.75379047 0.753349628 0.1585137 1 0.12201228 1

DB13007 1 0.623729973 1.75379047 0.753349628 0.1585137 1 0.12201228 1

DB13014 1 1.767402503 1.745287633 0.542671379 0.08481895 1 0.12201228 1

DB13025 1.25 1.78764675 1.751434425 0.152818387 0.000516751 1 0.217648936 1

DB13036 1 1.910230337 1.75379047 0.753349628 0.1585137 1 0.12201228 1

DB13044 1 2.098452034 1.75379047 0.753349628 0.1585137 1 0.12201228 1

DB13061 1 1.926529985 1.75379047 0.753349628 0.1585137 1 0.12201228 1

DB13074 1 1.158930464 1.75379047 0.753349628 0.1585137 1 0.12201228 1

DB13083 2 1.628709052 1.75379047 0.753349628 0.628097886 1 0.645919359 1

DB13125 1 1.89616071 1.75379047 0.753349628 0.1585137 1 0.12201228 1

DB13133 1 2.010294815 1.745287633 0.542671379 0.08481895 1 0.12201228 1

DB13139 2 1.664231974 1.75379047 0.753349628 0.628097886 1 0.645919359 1

DB13144 1 2.001234321 1.75379047 0.753349628 0.1585137 1 0.12201228 1

DB13146 1.9285714 1.687801515 1.773663076 0.187305714 0.795891301 1 0.604254789 1

DB13149 1.3333333 1.849604244 1.757244872 0.464542708 0.180743525 1 0.257238652 1

DB13150 1.3333333 2.775220259 1.757244872 0.464542708 0.180743525 1 0.257238652 1

DB13151 1.2857143 1.721607412 1.736866356 0.292454388 0.061458983 1 0.234170029 1

DB13152 1.7142857 1.88455706 1.736866356 0.292454388 0.4692279 1 0.473897096 1

DB13154 2 1.783830052 1.75379047 0.753349628 0.628097886 1 0.645919359 1

DB13155 1 0.774670587 1.75379047 0.753349628 0.1585137 1 0.12201228 1

DB13158 1 0.57516615 1.75379047 0.753349628 0.1585137 1 0.12201228 1

DB13164 1 1.054964286 1.75379047 0.753349628 0.1585137 1 0.12201228 1

DB13165 1.5 1.976398512 1.745287633 0.542671379 0.325634379 1 0.346299761 1

DB13166 1 1.050700597 1.75379047 0.753349628 0.1585137 1 0.12201228 1

DB13167 1 0.936047322 1.75379047 0.753349628 0.1585137 1 0.12201228 1

DB13170 1 2.388911565 1.75379047 0.753349628 0.1585137 1 0.12201228 1

DB13173 1 2.766207473 1.75379047 0.753349628 0.1585137 1 0.12201228 1

DB13174 2 1.875634333 1.745287633 0.542671379 0.680596551 1 0.645919359 1

DB13179 1 1.664428227 1.75379047 0.753349628 0.1585137 1 0.12201228 1

DB13191 2 1.754124129 1.770629523 0.307115021 0.772424541 1 0.645919359 1

DB13192 1.5 1.439580108 1.745287633 0.542671379 0.325634379 1 0.346299761 1

DB13200 1 2.625797706 1.75379047 0.753349628 0.1585137 1 0.12201228 1

DB13257 1.6666667 2.716621711 1.757244872 0.464542708 0.422702925 1 0.444812957 1

DB13269 2 1.84381682 1.759747174 0.221112233 0.861385463 1 0.645919359 1

DB13335 2 2.166293102 1.759334798 0.191532594 0.895536882 1 0.645919359 1

DB13345 1.3333333 1.694637516 1.760507724 0.1309174 0.000551332 1 0.257238652 1

DB13346 1.75 1.998320358 1.753692879 0.367879736 0.495995374 1 0.495810565 1

DB13437 2 2.054437998 1.759334798 0.191532594 0.895536882 1 0.645919359 1

DB13501 1.5 1.535092287 1.745287633 0.542671379 0.325634379 1 0.346299761 1

DB13503 2 1.240198247 1.75379047 0.753349628 0.628097886 1 0.645919359 1

DB13520 2 2.180159999 1.75379047 0.753349628 0.628097886 1 0.645919359 1

DB13581 1 1.444754621 1.757979426 0.337271213 0.012307606 1 0.12201228 1

DB13615 1 2.106190997 1.745287633 0.542671379 0.08481895 1 0.12201228 1

DB13643 2 1.627484685 1.759334798 0.191532594 0.895536882 1 0.645919359 1

DB13720 1 1.00676975 1.75379047 0.753349628 0.1585137 1 0.12201228 1

DB13729 1.5 1.166277729 1.753692879 0.367879736 0.245220308 1 0.346299761 1

DB13746 1.9375 1.849784894 1.752088368 0.130643051 0.922083004 1 0.609539735 1

DB13751 1.2 1.718200668 1.757979426 0.337271213 0.049023756 1 0.195688273 1

DB13781 2 3.022359017 1.75379047 0.753349628 0.628097886 1 0.645919359 1

DB13783 1.5 1.42258781 1.745287633 0.542671379 0.325634379 1 0.346299761 1

DB13800 1 3.212986955 1.757244872 0.464542708 0.051541591 1 0.12201228 1

DB13837 2 1.987319734 1.759334798 0.191532594 0.895536882 1 0.645919359 1

DB13857 1 1.410771805 1.75379047 0.753349628 0.1585137 1 0.12201228 1

DB13867 1.25 1.235688029 1.753692879 0.367879736 0.085471804 1 0.217648936 1

DB13869 1 1.791353518 1.745287633 0.542671379 0.08481895 1 0.12201228 1

DB13872 2 1.878448285 1.759334798 0.191532594 0.895536882 1 0.645919359 1

DB13873 1 2.763932644 1.757979426 0.337271213 0.012307606 1 0.12201228 1

DB13874 2 1.167700268 1.75379047 0.753349628 0.628097886 1 0.645919359 1

DB13875 2 1.66267362 1.75379047 0.753349628 0.628097886 1 0.645919359 1

DB13876 1 0.195135763 1.75379047 0.753349628 0.1585137 1 0.12201228 1

DB13877 2 2.896819291 1.75379047 0.753349628 0.628097886 1 0.645919359 1

DB13881 1 1.970486594 1.75379047 0.753349628 0.1585137 1 0.12201228 1

DB13884 2 1.762513398 1.75379047 0.753349628 0.628097886 1 0.645919359 1

DB13893 1 3.151286843 1.75379047 0.753349628 0.1585137 1 0.12201228 1

DB13894 1 2.39703119 1.75379047 0.753349628 0.1585137 1 0.12201228 1

DB13908 2 2.327470589 1.75379047 0.753349628 0.628097886 1 0.645919359 1

DB13914 2 2.283613214 1.75379047 0.753349628 0.628097886 1 0.645919359 1

DB13915 1 1.665038209 1.75379047 0.753349628 0.1585137 1 0.12201228 1

DB13919 1 1.490385649 1.75379047 0.753349628 0.1585137 1 0.12201228 1

DB13923 1.5 1.151159671 1.745287633 0.542671379 0.325634379 1 0.346299761 1

DB13925 1 1.372408357 1.75379047 0.753349628 0.1585137 1 0.12201228 1

DB13926 1 2.063478463 1.75379047 0.753349628 0.1585137 1 0.12201228 1

DB13928 2 2.003052046 1.75379047 0.753349628 0.628097886 1 0.645919359 1

DB13929 1 2.553891323 1.745287633 0.542671379 0.08481895 1 0.12201228 1

DB13930 1 1.78901983 1.745287633 0.542671379 0.08481895 1 0.12201228 1

DB13931 1.3333333 2.170533318 1.757244872 0.464542708 0.180743525 1 0.257238652 1

DB13932 2 2.38341974 1.75379047 0.753349628 0.628097886 1 0.645919359 1

DB13933 1.6666667 2.059857371 1.757244872 0.464542708 0.422702925 1 0.444812957 1

DB13934 1 0.543267397 1.75379047 0.753349628 0.1585137 1 0.12201228 1

DB13940 1 1.247067107 1.745287633 0.542671379 0.08481895 1 0.12201228 1

DB13943 1.3333333 1.310279063 1.757244872 0.464542708 0.180743525 1 0.257238652 1

DB13944 1.3333333 1.717471951 1.757244872 0.464542708 0.180743525 1 0.257238652 1

DB13946 1.3333333 1.712141111 1.757244872 0.464542708 0.180743525 1 0.257238652 1

DB13948 1 2.105334578 1.75379047 0.753349628 0.1585137 1 0.12201228 1

DB13949 1 2.980481301 1.75379047 0.753349628 0.1585137 1 0.12201228 1

DB13951 1.25 1.812440869 1.753692879 0.367879736 0.085471804 1 0.217648936 1

DB13952 1.2 1.535786547 1.759693587 0.247672276 0.01191636 1 0.195688273 1

DB13953 1.2 1.810764598 1.759693587 0.247672276 0.01191636 1 0.195688273 1

DB13954 1.2 1.818376698 1.759693587 0.247672276 0.01191636 1 0.195688273 1

DB13955 1.2 1.752238137 1.759693587 0.247672276 0.01191636 1 0.195688273 1

DB13956 1.2 1.783485069 1.759693587 0.247672276 0.01191636 1 0.195688273 1

DB13961 1.6428571 1.982015323 1.757145597 0.141561363 0.209734608 1 0.43037167 1

DB13967 1 2.751853981 1.75379047 0.753349628 0.1585137 1 0.12201228 1

DB13978 1 1.069849583 1.75379047 0.753349628 0.1585137 1 0.12201228 1

DB13983 2 2.008656417 1.736866356 0.292454388 0.815871351 1 0.645919359 1

DB13985 1 2.042285835 1.757979426 0.337271213 0.012307606 1 0.12201228 1

DB13988 1 2.112003256 1.75379047 0.753349628 0.1585137 1 0.12201228 1

DB13994 2 2.210619281 1.745287633 0.542671379 0.680596551 1 0.645919359 1

DB13995 2 2.125298801 1.753692879 0.367879736 0.748421796 1 0.645919359 1

DB13996 2 1.491226466 1.75379047 0.753349628 0.628097886 1 0.645919359 1

DB13998 1.5454545 2.064021599 1.759747174 0.221112233 0.166233199 1 0.372453903 1

DB13999 1.5454545 1.740171888 1.759747174 0.221112233 0.166233199 1 0.372453903 1

DB14001 1.7333333 1.493492242 1.760312929 0.204781867 0.447591756 1 0.48557866 1

DB14002 1.6 1.68364863 1.757979426 0.337271213 0.319747599 1 0.404623142 1

DB14004 1 1.985546732 1.745287633 0.542671379 0.08481895 1 0.12201228 1

DB14006 1.5714286 1.5610943 1.736866356 0.292454388 0.285803159 1 0.387676968 1

DB14009 1.3169248 1.621707231 1.755244111 0.11282555 5.12E-05 0.277496803 0.249158628 1

DB14010 1 1.530918887 1.745287633 0.542671379 0.08481895 1 0.12201228 1

DB14011 1.1646277 1.905808223 1.760376084 0.119854867 3.34E-07 0.001816666 0.180996849 1

DB14013 2 2.48755089 1.745287633 0.542671379 0.680596551 1 0.645919359 1

DB14014 2 2.413406758 1.745287633 0.542671379 0.680596551 1 0.645919359 1

DB14017 2 1.937880119 1.75379047 0.753349628 0.628097886 1 0.645919359 1

DB14019 1 1.627886926 1.75379047 0.753349628 0.1585137 1 0.12201228 1

DB14028 2 1.800672523 1.759334798 0.191532594 0.895536882 1 0.645919359 1

DB14034 1 2.216701341 1.75379047 0.753349628 0.1585137 1 0.12201228 1

DB14039 2 2.489902204 1.75379047 0.753349628 0.628097886 1 0.645919359 1

DB14040 2 2.151613353 1.75379047 0.753349628 0.628097886 1 0.645919359 1

DB14041 2 1.773064497 1.75379047 0.753349628 0.628097886 1 0.645919359 1

DB14042 2 1.636625506 1.75379047 0.753349628 0.628097886 1 0.645919359 1

DB14050 1.6666667 1.960681264 1.757244872 0.464542708 0.422702925 1 0.444812957 1

DB14059 1 1.406714253 1.757244872 0.464542708 0.051541591 1 0.12201228 1

DB14061 1 1.319509944 1.75379047 0.753349628 0.1585137 1 0.12201228 1

DB14062 1 1.671496396 1.75379047 0.753349628 0.1585137 1 0.12201228 1

DB14063 1 1.640045244 1.75379047 0.753349628 0.1585137 1 0.12201228 1

DB14064 1 0.557044488 1.75379047 0.753349628 0.1585137 1 0.12201228 1

DB14065 1 2.559378077 1.75379047 0.753349628 0.1585137 1 0.12201228 1

DB14066 1 1.077095711 1.75379047 0.753349628 0.1585137 1 0.12201228 1

DB14067 1.5 1.827645101 1.745287633 0.542671379 0.325634379 1 0.346299761 1

DB14068 1 1.349414454 1.75379047 0.753349628 0.1585137 1 0.12201228 1

DB14069 1 0.256214719 1.75379047 0.753349628 0.1585137 1 0.12201228 1

DB14070 1 1.524994328 1.75379047 0.753349628 0.1585137 1 0.12201228 1

DB14071 1 2.768043006 1.75379047 0.753349628 0.1585137 1 0.12201228 1

DB14072 1 3.462821544 1.75379047 0.753349628 0.1585137 1 0.12201228 1

DB14132 1 0.980344229 1.75379047 0.753349628 0.1585137 1 0.12201228 1

DB14146 1 1.495681591 1.757244872 0.464542708 0.051541591 1 0.12201228 1

DB14185 1.12 1.825388471 1.76622404 0.151326267 9.76E-06 0.05301429 0.163476751 1

DB14481 1.6 1.418494229 1.760507724 0.1309174 0.110095039 1 0.404623142 1

DB14487 1.3905527 1.759166949 1.75792302 0.071843204 1.58E-07 0.000860968 0.28644396 1

DB14488 1.6666667 1.975671049 1.763093512 0.211303302 0.324071624 1 0.444812957 1

DB14489 1.6666667 1.958095798 1.763093512 0.211303302 0.324071624 1 0.444812957 1

DB14490 1.6666667 1.853097974 1.763093512 0.211303302 0.324071624 1 0.444812957 1

DB14491 1.6666667 1.972688537 1.763093512 0.211303302 0.324071624 1 0.444812957 1

DB14498 2 1.57127098 1.75379047 0.753349628 0.628097886 1 0.645919359 1

DB14499 2 1.882218007 1.75379047 0.753349628 0.628097886 1 0.645919359 1
[truncated: 10,177 more chars]
